# Assessment of waterlogging tolerance in tea genotypes through morpho-physiological and biochemical profiling

**DOI:** 10.1371/journal.pone.0354144

**Published:** 2026-07-20

**Authors:** Md. Riyadh Arefin, Shitosri Mondal, Md. Fazle Rabbi, Hafsa Tasnim, Md. Alamgir Hossain, Md. Sabibul Haque, A. K. M. Golam Sarwar

**Affiliations:** 1 Botany Division, Bangladesh Tea Research Institute, Sreemangal, Moulvibazar, Bangladesh; 2 Department of Crop Botany, Bangladesh Agricultural University, Mymensingh, Bangladesh; Mustafa Kemal University: Hatay Mustafa Kemal Universitesi, TÜRKIYE

## Abstract

Tea is one of the major economic crops in Bangladesh, however, its productivity is severely constrained by waterlogging stress arising from erratic climatic changes. A significant knowledge gap persists regarding the morpho-physiological and biochemical determinants of waterlogging tolerance and post-stress recovery in tea, hindering the development of tolerant genotypes. Therefore, the present study aimed to investigate the integrated morpho-physiological and biochemical responses of tea genotypes under waterlogging stress to identify tolerant genotype(s) based on stress tolerance indices (STIs). Eight pre-screened tea genotypes—P/LAL/08/23, P/LAL/08/62, P/LAL/09/116, P/AFN/11/35, P/AFN/11/46, P/OTI/31, P/AFN/13/90, P/AFN/11/31, with two control clones, BT2 (popular variety) and TV9 (waterlogging-tolerant variety), were evaluated using 23 morpho-physiological and biochemical parameters maintaining a two-factorial completely randomized design experiment. One set of tea genotypes was exposed to a 14-day waterlogging phase, followed by a 14-day recovery phase (total 28 days of stress), while another set of plants was maintained under control (non-stress) conditions, for the same period, to calculate STIs. Root fresh weight (RFW), leaf number (NL), net photosynthesis (*Pn*), transpiration rate (*E*), stomatal conductance (*g*_*s*_), relative leaf water content (RWC), and absolute growth rate (AGR) were significantly decreased under waterlogging condition compared to the control. Leaf chlorophyll *a* (CHA), chlorophyll *b* (CHB), and total carotenoids (CRTN), were reduced under stress, whereas proline content (of leaf: PRLF and root: PRRT), total antioxidant activity (of leaf: TACL and root: TACR), and lipid peroxidation (of leaf: LOPL and root: LPOR) increased as a part of adaptive responses. The traits such as RFW, NL, *Pn*, *E*, *g*_*s*_, RWC, AGR, CHA, CHB, CRTN, and LPOL, were detected as most influential at both phases of stress conditions from principal component analysis. Ultimately, the P/AFN/13/90 was identified as the most waterlogging-tolerant genotype, exhibiting higher STIs for maximum traits during both the waterlogging and recovery phases.

## Introduction

Plants face various types of biotic and abiotic stresses throughout their life cycles [1,2]. Among abiotic stresses, waterlogging is one of the major stressors affecting plant growth and development [3]. Nearly 10–16% of the world’s arable land is affected by waterlogging, which is increasingly threatening agricultural productivity and sustainability [4]. Waterlogging is the state of soil when it is partially or completely saturated with water, displacing air spaces of soil for a period of time [5]. There are several causes of waterlogging stress, but most of these are due to the impact of erratic climate change. Natural climatic factors causing waterlogging include excessive and unevenly distributed rainfall, increasing sea levels, natural calamities, elevation of groundwater table, topography, soil properties, etc. [6]. At the same time, anthropogenic factors include ineffective drainage systems, compacted soil, over-irrigation, improper cultural practices, etc. [7]. Under waterlogged conditions, plants suffer hypoxia or anoxia and are affected anatomically, morphologically, physiologically, and biochemically, hindering plants’ growth and development [8]. Waterlogging can be categorized either based on available oxygen (O₂) in soil, *i.e.,* hypoxia (decreased O₂) and anoxia (no available O₂) [9] or based on occurrence pattern or duration of waterlogging, *i.e.*, transient (intermittent) and continuous (long-term) [10]. In waterlogging stress, roots suffer primarily from insufficient O₂. Plants accumulate reactive oxygen species (ROS) and some toxic metabolites resulting from anaerobic respiration, which disrupts gas exchange capacity by stomatal closure with reduced photosynthesis and ultimately reduces crop yield in majority of crops, including tea [11–13].

Tea (*Camellia sinensis* (L.) O. Kuntze) is one of the major beverage crops that has therapeutic, socio-economic, and cultural value worldwide [14]. Currently, tea is grown in 64 countries across diverse topographical, climatic, and ecological zones, such as hilly areas [15], plain lands [16], and tropical to temperate regions [17]. Water is essential for tea-plant growth, but it must not be stagnant. Normally, tea is grown in hilly areas, but the areas between two adjacent hills, which are termed locally as ‘*kunchi*’ [18], also suffer from waterlogging due to percolation of water beneath. Waterlogging caused by heavy rainfall or floods is responsible for 15–25% annual loss in North-East India [19], 10% reduction in production in West Bengal and Assam [20], and a significant reduction in tea yield in Sri Lanka [21]. Tea is the most widely consumed drink in Bangladesh, contributing 1% to the national gross domestic product (GDP) [22], and the tea-growing regions also suffer from waterlogging, particularly in greater Sylhet, Chattogram, and northern tea-growing areas of Bangladesh [23].

Tea plants are affected drastically by waterlogging, morphologically through deformation of roots, leaf yellowing (chlorosis), leaf dropping, decreased plant height, low root-shoot weights or dry matter; physiologically through reduced photosynthesis, stomatal conductance, transpiration, and leaf water potential; and biochemically by decreased leaf pigments, phenylalanine ammonia-lyase (PAL) activity, wax content, flavonoids and polyphenols [13,19,24,25]. Excess stagnant water in tea soil reduces organic matter content and nutrient levels, ultimately decreasing crop yield and quality [26]. Waterlogging-tolerant tea genotypes exhibit some changes, such as developing longer roots and aerenchyma tissues, increasing proline content, wax content, polyphenols, flavonoids, synthesizing antioxidants, etc. [13,19,24,25]. Waterlogging and other environmental stresses disrupt the metabolic pathways of tea plant, thereby weakening the bushes. Such weakened plants become highly susceptible to numerous diseases, including red rust, dieback, as well as pests like tea mosquito bug, red spider mites, etc. [27]. A proper drainage system helps to minimize the waterlogging effect in tea estates. Common practices to improve drainage include constructing deep main drains and lateral sub-drains, implementing contour drainage systems, and so on [26]. Despite these technologies, waterlog-tolerant plants are considered a more effective and sustainable long-term strategy [27].

Although waterlogging has become a serious constraint to tea cultivation, in-depth research on waterlogging in tea remains limited. Moreover, most studies focused on general responses of waterlogging and were largely concentrated in countries like India, Sri Lanka, where several tolerant varieties, including TV9, SNT-10, MNPR/51/P2, GNGA/31/P3, GNGA/31/P4, DFLGR/34/P8, and TRI 2023 have already been developed [21,28,29]. Countries like China have emphasized the importance of soil status and nutrient accumulation in plants under waterlogging conditions [26,30]. In contrast, no systemic study on waterlogging tolerance in tea has yet been conducted in Bangladesh; no tolerant tea variety is currently available for commercial cultivation [23]. In addition, the key adaptive traits have not been systematically identified yet, as well as their relative importance and combined role in conferring tolerance are still poorly understood. Furthermore, previous studies have predominantly focused on waterlogging stress alone, overlooking the important post-stress recovery response in tea. To address this gaps, the current study was conducted with three primary objectives: (i) to investigate the effect of waterlogging stress along with post-stress recovery on tea plant growth, (ii) to identify key traits contributing to tolerance, and (iii) to screen the tolerant tea genotype(s) effectively through integrated morphological, physiological, and biochemical profiling under waterlogging stress, for potential use in future breeding programs.

## Materials and methods

### Genotypes and experimental conditions

The experiment was conducted at Bangladesh Tea Research Institute (BTRI), Moulvibazar (24.352° N, 91.736° E) from August to December 2024. This experiment was conducted using a two-factorial completely randomized design with five replications, in which five plants of each genotype constituted five replicates. In this study, factor one was considered as ‘genotypes’, while factor two represented the ‘experimental condition’.

Screening at the sapling stage enables rapid, cost-effective, and large-scale evaluation of breeding materials before field trials. Therefore, a total of ten genotypes (factor-1) of 12-month-old saplings were used in the current study. Among these, eight genotypes, such as P/LAL/08/23 (V1), P/LAL/08/62 (V2), P/LAL/09/116 (V3), P/AFN/11/35 (V4), P/AFN/11/46 (V5), P/OTI/31 (V6), P/AFN/13/90 (V7), P/AFN/11/31 (V8), were selected from a previous study [23]. These genotypes represent advanced breeding lines maintained at BTRI and were originally collected from different waterlogging-prone tea estates of Bangladesh through individual selection. Owing to their origin from waterlogging-affected areas, these genotypes were presumed to have waterlogging tolerance abilities. However, their tolerance ability has not yet been systematically evaluated. Therefore, these eight genotypes, together with two check varieties, were selected for systemic assessment of waterlogging tolerance. The two check varieties included BT2, the most widely cultivated tea variety in Bangladesh [31], and TV9, a renowned waterlogging-tolerant variety from India [28,29].

The saplings for this experiment were obtained from the vegetative cuttings of selected genotypes. Cuttings were collected from fresh, mature, pest-disease free shoots of mother bushes and treated with CUPRAVIT® OB 50 WP (K&N Efthymiadis, Sindos, Greece) @ 2.24 g per liter of water for disinfection. The disinfected cuttings were then placed in polytubes (height: 22 cm, width: 15 cm, thickness: 0.004 cm). Each polybag contained 2.5 kg cow dung mixed with sandy-loam soil (soil: cow dung ratio = 3:1) and triple superphosphate (TSP) at 500 gm for 1 m³ soil [32,33]. Watering was done at each one-day interval. Recommended pesticides were applied as needed, but no additional hormones or fertilizers were applied to support sapling development. Finally, pest-disease free, healthy, uniform saplings of 12 months were selected for the current experiment.

**Fig 1:**
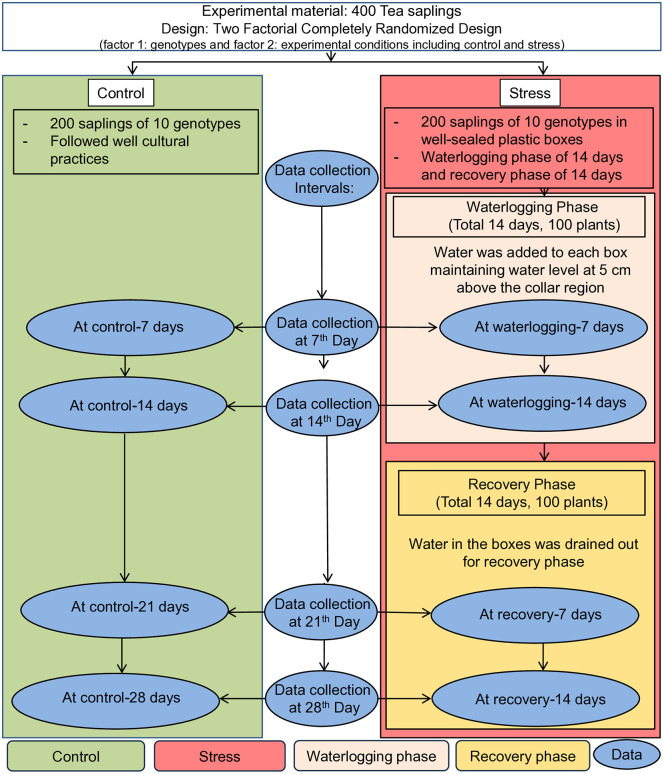
Schematic representation of the experimental methodology for assessing waterlogging tolerance in selected tea genotypes.

The rooted saplings (within polybags) of each genotype were placed under two conditions to assess the waterlogging effect, such as ‘without stress’ as control and ‘waterlogging’ as stress, which both were considered as ‘factor-2’. The waterlogging stress was induced by keeping plants of each genotype in separate plastic boxes (length: 45.72 cm, width: 30.48 cm, and depth: 30.48 cm) and sealing the boxes with polyethylene sheets to retain water. Then, fresh water (pH 7.15–7.2) was added to each box, maintaining the water level at 5 cm above the collar region of the plants for simulating waterlogging conditions and kept for 14 days (waterlogging phase). Then water of the boxes was drained out facilitating normal growth conditions for a recovery period of 14 days. Under control conditions (without stress), regular watering was applied at two-day intervals (one day of watering followed by one day without irrigation). A total of 200 saplings were maintained under control conditions, while another 200 saplings were subjected to stress conditions. Among the stress-conditioned plants, 100 saplings were used for the waterlogging phase, and remaining 100 saplings for the recovery phase. In total, 400 saplings representing 10 genotypes were maintained throughout the experimental period. Data were collected at 7-day intervals across 23 morphological, physiological, and biochemical traits for both conditions, resulting in a total of 8 data sets, where for each set or interval had 50 plants (10 genotypes × 5 replications = 50 plants). Therefore, the entire datasets were categorized into control (control-7, control-14, control-21, control-28) and stress (waterlog-7, waterlog-14, recovery-7, recovery-14). Destructive sampling methods for leaf and root collection were performed on the same day at each sampling interval. The detailed data collection timeline at different phases (Control, waterlogging and recovery) is presented in Fig 1.

The whole experiment was established in a ‘Vegetatively Propagated Nursery (VP Nursery)’ fully covered by high-density polyethylene (HDPE) agronet (Shreeji Agronet Industries, Gujarat, India) to ensure a uniform growth environment by minimizing the effects of external UV radiation, wind, fluctuations in temperature, and humidity. During the 28-day experiment-establishment period (1 to 28 August 2024), the average daily temperature (°C) and relative humidity (%) inside the controlled VP nursery ranged from 26.8° C to 28.9° C and 85.25% to 89.5%, respectively (S1 Fig). Considering the homogenous environmental conditions and the absence of noticeable environmental changes within the VP nursery, a two-factor factorial Completely Randomized Design (CRD) was adopted for current experiment.

### Morphological traits

The morphological traits such as plant height, shoot fresh weight, root fresh weight, shoot dry weight, root dry weight, total dry matter, vertical root length, and number of leaves, were determined throughout the experimental period. Plant height (PH) was measured in centimeters (cm) by a meter scale from the soil surface of the polybag to the stem apex [34]. Shoot fresh weight (SFW) and root fresh weight (RFW) were recorded by a digital weight scale (KERN ABJ-NM, Kern & Sohn GmbH, Germany) in grams (g). In contrast, shoot dry weight (SDW) and root dry weight (RDW) were measured by oven-drying shoots and roots, respectively, in a GENLAB MINI/30 oven (Genlab Limited, Widnes, UK) at 80 ± 2 °C for 72 hours [35]. Total dry matter (TDM) was calculated by summing the SDW and RDW of respective genotypes [34]. Roots of each plant were carefully washed, and vertical root length (VTRL) was recorded in centimeters (cm) by measuring the longest root. The number of leaves on each plant was also recorded and denoted as NL.

### Physiological traits

The fifth leaf (5^th^) from the top was tagged for measuring physiological and biochemical traits. The gas exchange parameters such as net photosynthesis (*Pn*, µmol CO₂ m ⁻ ² s ⁻ ¹), transpiration rate (*E*, mol H₂O m ⁻ ² s ⁻ ¹), and stomatal conductance (*g*_*s*_, mmol m ⁻ ² s ⁻ ¹) were measured by a portable photosynthesis system (LCi-SD Photosynthetic system, ADC BioScientific Ltd., Herts, UK). The gas exchange parameters were recorded at a photosynthetic photon flux density (PPFD) of nearly 850 µmol m ⁻ ² s ⁻ ¹ with 400 ppm CO_2_ and ambient temperature. The relative leaf water content (RWC) was determined by taking 0.5 g (FW, fresh weight) mature and disease-free fresh leaves of all genotypes and soaking them in water for 24 hours to obtain turgid weight (TW). Leaves were then oven dried at 80 ± 2° C for 72 hours (GENLAB MINI/30 oven, Genlab Limited, Widnes, UK), and finally, dry weight (DW) was measured. The RWC (as percentage) was calculated as RWC (%) = {(FW – DW) ÷ (TW – DW)} × 100 [36]. The absolute growth rate (AGR) was calculated by AGR = (W₂ – W_1_) ÷ (T₂ – T₁), where (W₂ – W₁) is the difference between the final dry weights (W₂) measured at final time (T₂) and initial dry weight (W₁) at initial time (T₁) [37]. The weights were measured by KERN ABJ-NM weight machine (Kern & Sohn GmbH, Germany). The greenness of leaves (SPAD) was measured by a portable SPAD meter (SPAD-502Plus, Konica Minolta, Osaka, Japan) by averaging three values (at three parts of each leaf), avoiding the midrib region.

### Biochemical properties

Leaf pigments (chlorophyll *a*, chlorophyll *b*, and total carotenoids) were determined by taking 50 mg fresh leaf samples and putting them into 10 mL 80% acetone (Supelco, Sigma-Aldrich, Massachusetts, USA) in the amber glass vial under the dark condition for seven days for complete extraction of pigments into acetone mixture [36,38]. The absorbance readings of the acetone solutions were taken at three different wavelengths like 470 nm (*A*₄₇₀), 646.8 nm (*A*₆₄₆.₈), and 663.2 nm (*A*₆₆₃.₂) by an ultraviolet-visible (*UV-Vis*) spectrophotometer (LAMBDA 1050 + , PerkinElmer, Springfield, United States), and pigments were calculated using the following equations and expressed as mg g ⁻ ¹ fresh weight (mg g ⁻ ¹ FW).

Chlorophyll *a* (CHA) = 12.25 × *A*₆₆₃.₂ – 2.79 × *A*₆₄₆.₈

Chlorophyll *b* (CHB) = 21.50 × *A*₆₄₆.₈ – 5.10 × *A*₆₆₃.₂

Total carotenoids (CRTN) = (1000 × *A*₄₇₀ – 1.82 × CHA – 85.02 × CHB) ÷ 198

The proline contents of leaf (PRLF) and root (PRRT) were measured by ninhydrin colorimetric method [39] with some slight change [40]. About 50 mg of fresh sample (leaf and root) were grounded in 2.5 mL methanol (Supelco, Sigma-Aldrich, Massachusetts, USA) and centrifuging (MicroCL 21R centrifuge, Thermo Fisher Scientific India Pvt Ltd., India) the sample at 12000 rpm for 15 minutes to obtain supernatant. The ninhydrin mixture was made by mixing 1% ninhydrin with 60% acetic acid and 20% ethanol (Supelco, Sigma-Aldrich, Massachusetts, USA). Then, 1 ml of the ninhydrin mixture and 500 µL of the methanolic plant extract were mixed again and incubated in a hot-water bath (Niive NB20 Unstirred Water bath, Crown Healthcare, Nairobi, Kenya) at 90° C for 30 minutes. Finally, after cooling, the absorbances were recorded at 520 nm by a similar spectrophotometer, and proline concentrations were expressed as mg g ⁻ ¹ FW, which were prepared from the standard curve of L-proline (y = 0.0453x ‒ 0.0146, r² = 0.996). The phosphomolybdenum assay method [41], with minor modifications [40], was used to measure the total antioxidant capacity of leaves (TACL) and roots (TACR). The 100 µl methanolic extracts were mixed with 1 ml phosphomolybdenum assay consisted of 0.6 M sulfuric acid, 28 mM sodium phosphate, and 4 mm ammonium molybdate. The reaction mixtures were incubated in a hot water bath (95 °C) for 90 minutes, and the absorbance of the cooled mixtures was measured at 695 nm. The concentrations were obtained from a standard reference (y = 0.00458493x + 0.07094, r² = 0.997) and expressed as ascorbic acid equivalent (mg eq. AA g ⁻ ¹ FW). Lipid peroxidations in leaf (LPOL) and root (LPOR) were measured by thiobarbituric acid reactive substance (TBARS) assay expressed as malondialdehyde (MDA) content (nmol g ⁻ ¹ FW) [42,43]. About 100 mg of fresh leaf and root samples were ground with 0.1% trichloroacetic acid, and the mixtures were then centrifuged at 12000 rpm at 4° C for 15 minutes. The 500 µL supernatants were mixed with 1 ml mixture of 0.5% thiobarbituric acid and 20% trichloroacetic acid. The reaction mixtures were then incubated in hot water bath for 15 minutes at 95° C and immediately placed in icebox for 10 minutes. The mixtures were centrifuged again for 5 minutes at 10000 rpm at 4° C. The absorbance of prepared samples was measured at 532 nm and 600 nm wavelengths by the same spectrophotometer. The differences between the readings of 532 nm and 600 nm (*A*₅₃₂ – *A*₆₀₀) were estimated to calculate the lipid peroxidation as MDA content with the help of the extinction coefficient of 155 mM ⁻ ¹ cm ⁻ ¹.

### Statistical analysis of data

After confirming normality of all datasets using the Shapiro–Wilk test, the two-factorial CRD analysis of variance (ANOVA) regarding the parameters was computed by the Minitab software (version 21). The boxplot and correlation matrix were constructed using the *ggplot2* package, and Principal Component Analysis (PCA) was carried out using the *fviz_pca* and *corrplot* packages [44] in RStudio (version 2025.05.1 + 513, released 05-06-2025) and R (version 4.5.1) programs. The graphs of the percent increase or decrease of each trait in stress condition relative to control were made by SigmaPlot software (Version: 14) developed by Grafiti LLC. The two-way clustering heatmaps with dendrograms and radar plots were generated using OriginPro (Version 2025) from OriginLab. The PCA, correlation matrix, and clustering heatmaps were prepared from the stress tolerance indices (STI) of each trait, STI (%) = (Tₛ ÷ T󠇎ₒ) × 100, where Tₛ was the trait value at the end of waterlogging or recovery phase, Tₒ is the trait value under corresponding optimum control condition [45].

## Results

### Analysis of variance

Significant variations were observed among maximum measured traits (except AGR) in genotypic factor (factor 1) throughout the experiment (S1 Table). On the other hand, all traits across experimental conditions (factor 2) were observed to be significant. The maximum number of traits performed significant interaction effects (Genotype × Condition) except RDW, VRTL, and NL. But the interaction effects were always less than the two main factors (genotype and experimental condition).

### Waterlogging *vs.* control

**Fig 2:**
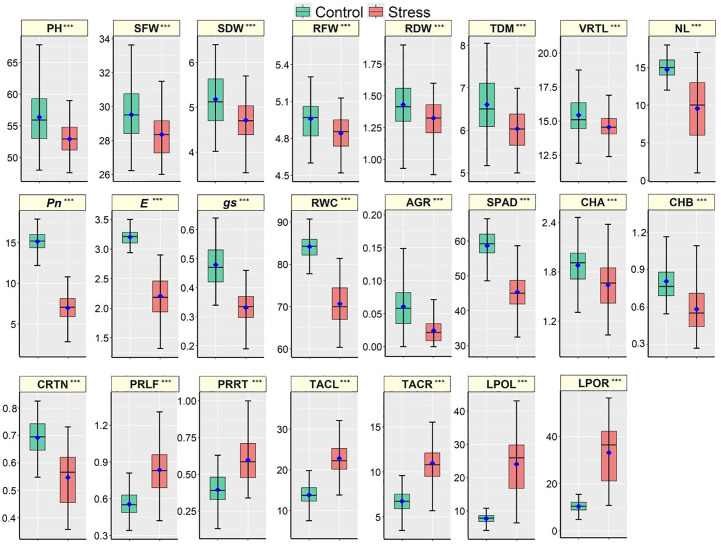
Responses of ten genotypes for 23 traits under control and stress conditions. PH: Plant height (cm), SFW: Shoot fresh weight (g), SDW: Shoot dry weight (g), RFW: Root fresh weight (g), RDW: Root dry weight (g), TDM: Total dry matter (g), VRTL: Vertical root length (cm), NL: Number of leaves per plant*, Pn*: Net photosynthesis (µmol CO₂ m ⁻ ² s ⁻ ¹), *E*: Transpiration rate (mol H₂O m ⁻ ² s ⁻ ¹)*, g*_*s*_: Stomatal conductance (mmol m ⁻ ² s ⁻ ¹), RWC: Percent relative leaf water content, AGR: Absolute growth rate, SPAD: SPAD value of leaves, CHA: Chlorophyll *a* (mg g ⁻ ¹ FW), CHB: Chlorophyll *b* (mg g ⁻ ¹ FW), CRTN: Total carotenoids (mg g ⁻ ¹ FW), PRLF: Proline content in leaf (mg g ⁻ ¹ FW), PRRT: Proline content in root (mg g ⁻ ¹ FW), TACL: Total antioxidant capacity of leaf (mg eq. AA g ⁻ ¹ FW), TACR: Total antioxidant capacity of root (mg eq. AA g ⁻ ¹ FW), LPOL: Lipid peroxidation of leaf (MDA content nmol g ⁻ ¹ FW) and LPOR: Lipid peroxidation of root (MDA content nmol g ⁻ ¹ FW). The ‘***’ stands for the level of significance at p ≤ ‘0.1%’.

The overall response of the tea plant under waterlogging stress showed a remarkable variation in morphological performance, physiological functioning, and biochemical stability compared to the control condition (Fig 2). Morphological traits (PH, NL, SFW, SDW, RFW, RDW, VRTL, TDM) and physiological traits (AGR, *Pn*, *E*, *g*_*s*_, RLWC, SPAD) were notably decreased in waterlogged conditions compared to control for all genotypes. Some biochemical traits (CHA, CHB, and CRTN) also decreased, whereas most biochemical traits, such as PRLF, PRRT, TACL, TACR, LPOL, and LPOR, substantially increased under stress compared to the control environment.

### Interaction effects of genotypes and experimental conditions

#### Morphological traits.

**Fig 3:**
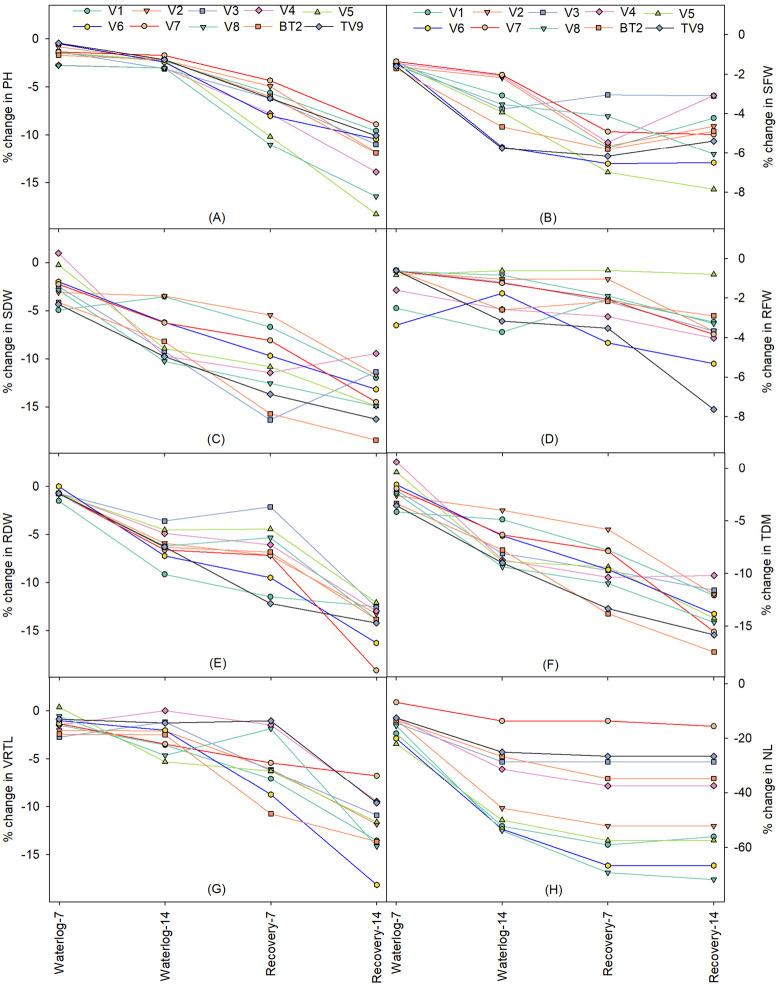
Relative changes in morphological traits in stress (waterlogging and recovery phases) than control conditions. (A) PH: Plant height, (B) SFW: Shoot fresh weight, (C) SDW: Shoot dry weight, (D) RFW: Root fresh weight, (E) RDW: Root dry weight, (F) TDM: Total dry matter, (G) VRTL: Vertical root length, and (H) NL: Number of leaves per plant.

All genotypes showed a substantial reduction in morphological traits compared to the control, and the extent of decline increased with stress duration. The decline became more pronounced during the recovery phases due to stunted growth under stress conditions compared to the profuse growth in the control (Fig 3). The interaction responses (genotypes x experimental conditions) across all the observational intervals were provided in S2-S9 Figs.

Among the evaluated genotypes, V7 exhibited the lowest reduction in plant height (PH) with decreases of only 1.73% and 8.90% at Waterlog-14 days and Recovery-14 days, respectively, compared to the control (Fig 3A). A comparable trend was observed for shoot fresh weight (SFW), where V7 showed the least decline (2.04%) under Waterlog-14 days, and V4 performed best during Recovery-14 days (3.08% reduction) (Fig 3B). For shoot dry weight (SDW), V2 exhibited greater stability at Waterlog-14 days with 3.48% reduction, whereas V4 maintained the minimum decrease of 9.45% under Recovery-14 days (Fig 3C). The root fresh weight (RFW) was least affected in V5 with declines of 0.62% and 0.80% at Waterlog-14 days and Recovery-14 days, respectively (Fig 3D). The smallest decrease in RDW was detected in V3 under Waterlog-14 days (3.60%) and V5 (12.08%) at Recovery-14 days (Fig 3E). Regarding TDM, V2 (4.01%) showed a smaller decline at Waterlog-14 days, but V4 (10.20%) exhibited the least decrease at Recovery-14 days (Fig 3F). Again, the slightest decrease in VRTL was observed in V4 at Waterlog-14 days (1.18%) and in V7 at Recovery-14 days (6.77%) (Fig 3G). In terms of number of leaves (NL), V7 consistently showed superior tolerance, with declines of 13.64% during Waterlog-14 days and 15.56% during Recovery-14 days compared with the control condition (Fig 3H).

#### Physiological traits.

**Fig 4:**
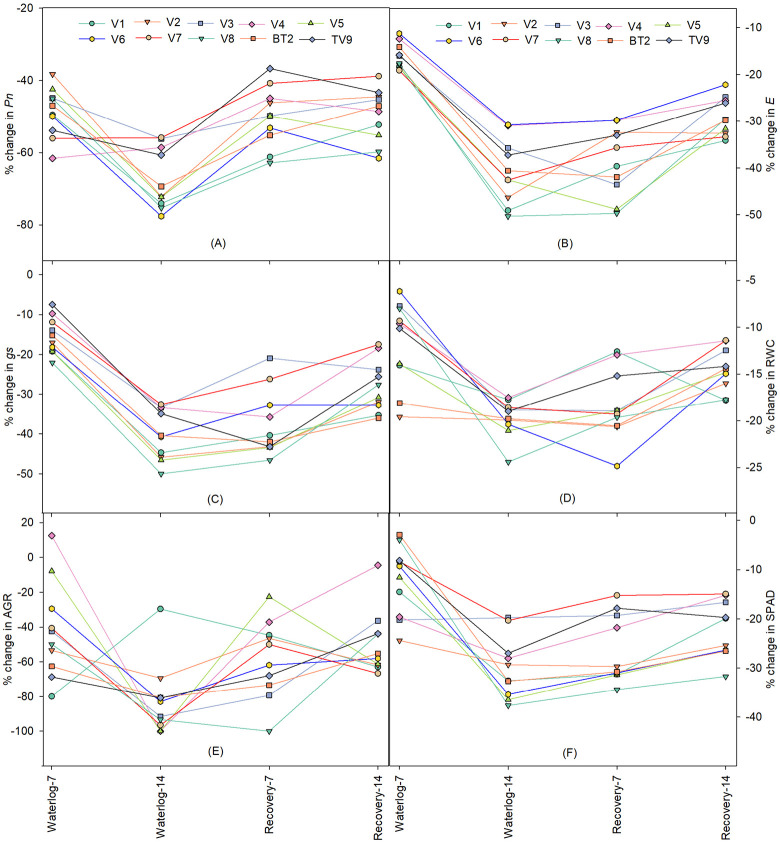
Percent changes in physiological parameters- (A) *Pn*: net photosynthesis, (B) *E*: Transpiration rate, (C) *g*_*s*_: Stomatal conductance, (D) RWC: Percent relative leaf water content, (E) AGR: Absolute growth rate, and (F) SPAD: SPAD value of leaves of 10 genotypes under stress conditions relative to control.

Waterlogging stress exerted a pronounced effect on the physiological attributes in tea saplings (S10-S15 Figs). At Waterlog-14 days, net photosynthesis (*Pn*) decreased to a minimum of 55.89% and 38.80% at Waterlogging-14 days and Recovery-14 days, respectively, in V7 (Fig 4A). Likely *Pn*, the transpiration rate (*E*) was also impaired by stress conditions, where the least decreases compared to control during Waterlog-14 days and Recovery-14 days were noticed in V6 by 30.77% and 22.19%, respectively (Fig 4B). Again, the least declines in stomatal conductance (*g*_*s*_) were observed in V7 at both Waterlog-14 days (32.56%) and Recovery-14 days (17.50%) (Fig 4C). The minimum reduction in relative leaf water content (RWC) was observed in V4 (17.53%) at Waterlog-14 days and in V7 (11.41%) at Recovery-14 days, compared to control (Fig 4D). The more stable absolute growth rate (AGR) was noticed in V1 during Waterlog-14 days with a minimum reduction of 29.62% and in V4 during Recovery-14 days (4.56%) (Fig 4E). In terms of SPAD value, V3 demonstrated the lowest decrease of 19.78% at Waterlog-14 days; however, V7 showed comparatively higher tolerance with a minimum decline of 14.96% at Recovery-14 days compared to the control condition (Fig 4F).

#### Biochemical traits.

**Fig 5:**
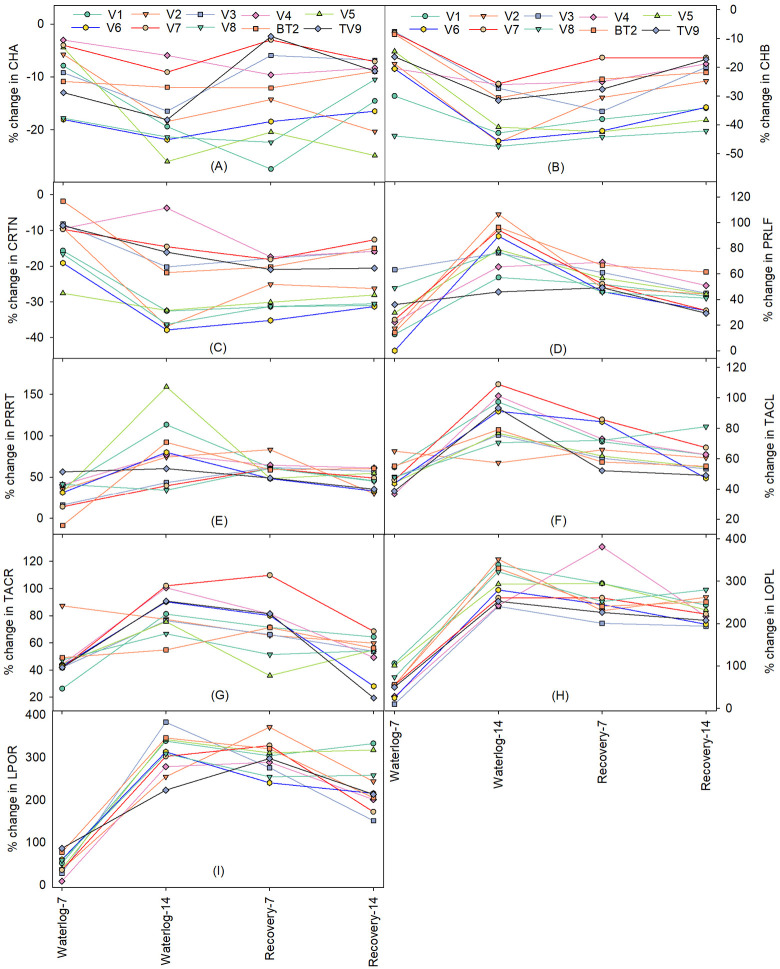
Biochemical changes (%) in- (A) CHA: Chlorophyll *a*, (B) CHB: Chlorophyll *b*, (C) CRTN: Total carotenoids, (D) PRLF: Proline content in leaf, (E) PRRT: Proline content in root, (F) TACL: Total antioxidant capacity of leaf, (G) TACR: Total antioxidant capacity of root, (H) LPOL: Lipid peroxidation of leaf, and (I) LPOR: Lipid peroxidation of root in ten genotypes under stress conditions compared to control condition.

Distinct biochemical variations were recorded throughout the study period (S16-S24 Figs). At waterlog-14 days, chlorophyll *a* (CHA) declined least in V4 (5.95%), followed by V7 (9.12%), whereas Recovery-14 days, V3 (6.85%) and V7 (7.12%) exhibited lower reductions compared to control (Fig 5A). For chlorophyll *b* (CHB), V7 consistently showed the least decrease at both Waterlog-14 days (25.77%) and Recovery-14 days (16.67%) (Fig 5B). Similarly, total carotenoids (CRTN) decreased minimally in V4 (3.75%) at Waterlog-14 days and in V7 (12.63%) at Recovery-14 days (Fig 5C).

Leaf proline concentration (PRLF) increased most in V2 at Waterlog-14 days (106.38%) and in BT2 at Recovery-14 days (61.40%) (Fig 5D), while the amount of root proline (PRRT) showed maximum elevation in V5 Waterlog-14 days (159.09%) and in V4 at Recovery-14 days (61.22%) (Fig 5E). Notably, V7 and V4 accumulated the highest leaf proline (1.32 mg g ⁻ ¹ FW) and root proline (0.94 mg g ⁻ ¹ FW) during Waterlogg-14 days (S19 and S20 Figs). Total antioxidant capacity in leaf (TACL) increased most in V7 at Waterlog-14 days (108.83%) and V8 at Recovery-14 days (81.05%) (Fig 5F). The highest antioxidant capacity in roots (TACR) was observed in V7 at both Waterlog-14 days (101.94%) and Recovery-14 days (68.44%) (Fig 5G). Under severe waterlogging stress, V7 demonstrated the highest antioxidants in both leaf (34.54 mg eq. AA g ⁻ ¹ FW) and root (28.84 mg eq. AA g ⁻ ¹ FW) (S21 and S22 Figs). Elevated lipid peroxidation with disrupted cellular integrity was also detected under stress conditions. But the lowest increase in lipid peroxidation in leaves (LPOL) was observed in V3 at Waterlog-14 days (240.65%) and at Recovery-14 days (193.68%) (Fig 5H). Again, the minimum decline in lipid peroxidation in the root (LPOR) was noticed in TV9 (223.25%) and V3 (151.08%) at Waterlog-14 days and Recovery-14 days, respectively, with the comparison to the control condition (Fig 5I).

### Principal Component Analysis (PCA)

**Fig 6:**
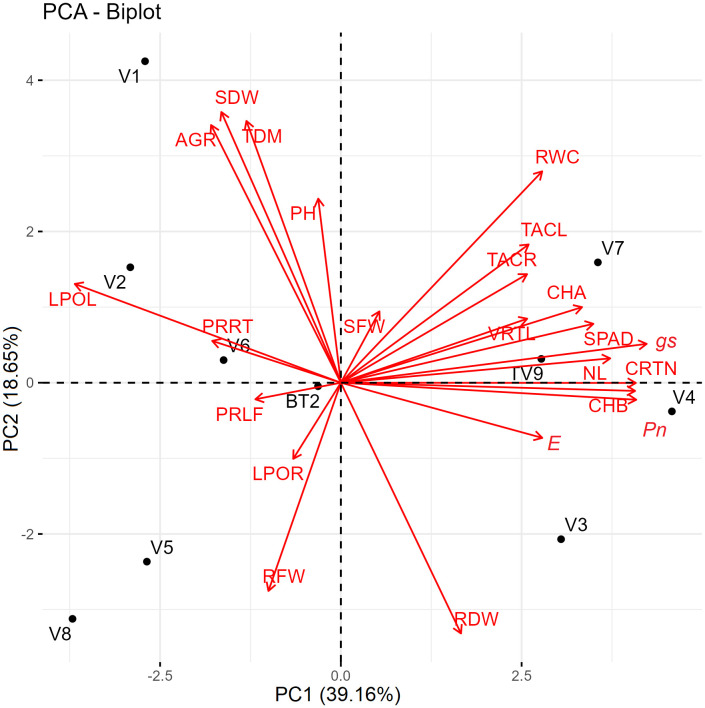
Principal component analysis (PCA)-biplot exhibits the interrelation among the traits and genotypes after the waterlogging phase. PH: Plant height (cm), SFW: Shoot fresh weight (g), SDW: Shoot dry weight (g), RFW: Root fresh weight (g), RDW: Root dry weight (g), TDM: Total dry matter (g), VRTL: Vertical root length (cm), NL: Number of leaves per plant*, Pn*: Net photosynthesis (µmol CO₂ m ⁻ ² s ⁻ ¹), *E*: Transpiration rate (mol H₂O m ⁻ ² s ⁻ ¹)*, g*_*s*_: Stomatal conductance (mmol m ⁻ ² s ⁻ ¹), RWC: Percent relative leaf water content, AGR: Absolute growth rate, SPAD: SPAD value of leaves, CHA: Chlorophyll *a* (mg g ⁻ ¹ FW), CHB: Chlorophyll *b* (mg g ⁻ ¹ FW), CRTN: Total carotenoids (mg g ⁻ ¹ FW), PRLF: Proline content in leaf (mg g ⁻ ¹ FW), PRRT: Proline content in root (mg g ⁻ ¹ FW), TACL: Total antioxidant capacity of leaf (mg eq. AA g ⁻ ¹ FW), TACR: Total antioxidant capacity of root (mg eq. AA g ⁻ ¹ FW), LPOL: Lipid peroxidation of leaf (MDA content nmol g ⁻ ¹ FW), and LPOR: Lipid peroxidation of root (MDA content nmol g ⁻ ¹ FW).

Principal component analysis (PCA) was performed for both the waterlogging and recovery phases on the 14th day to determine the traits most responsible for tolerance. At the end of waterlogging phase, PC1 and PC2 were responsible for 39.16% and 18.65% of total variance, respectively, while cumulatively designated for 57.81% of total variance (S2 Table, Fig 6). Some degree of relationship between the traits can also be predicted from the direction of arrows in the biplot (Fig 6). Generally, the same direction in a biplot implies positive relationship, like RWC and TACL, TACL and TACR, CHA and SPAD, *g*_*s*_ and SPAD, *Pn* and CHB, LPOL and PRRT, AGR and TDM, PH and TDM, etc. (Fig 6). Opposite directions of arrows generally indicate a negative relationship, such as RDW and TDM, RDW and SDW, AGR and RDW, *E* and LPOL, *g*_*s*_ and LPOL, *Pn* and LPOL, PRLF and TACL, PRLF and TACR, SPAD and PRRT, LPOL and CRTN (Fig 6). The genotypes V7, TV9, and V3, V4 were placed in the 1^st^ and 4^th^ quadrants of the PCA-biplot at Waterlog-14 days, respectively (Fig 6). Traits such as *g*_*s*_, *Pn*, CRTN, CHB, NL, LPOL, SPAD, CHA, *E,* and RWC strongly influenced PC1, while SDW, TDM, AGR, RDW, RWC, RFW, and PH mainly contributed to PC2 at Waterlog-14 days (Figs S25 and S26).

**Fig 7:**
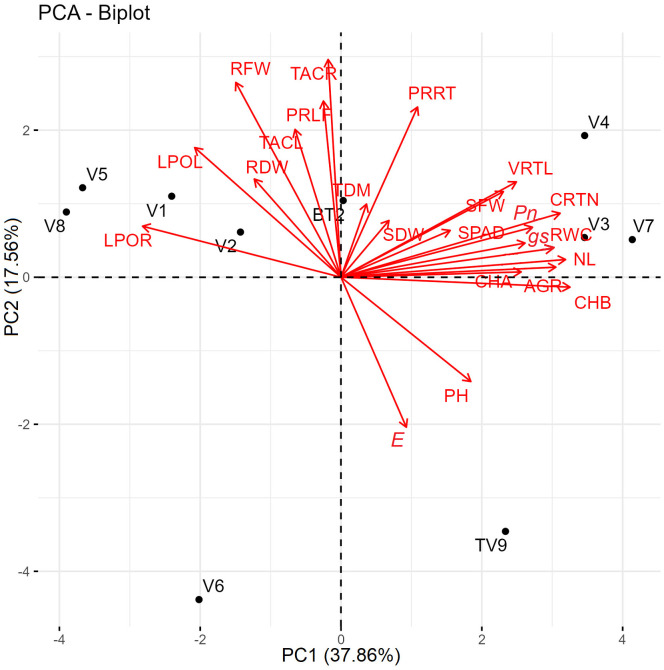
Principal component analysis (PCA)-biplot exhibits the interrelation among the traits and genotypes under recovery phase. PH: Plant height (cm), SFW: Shoot fresh weight (g), SDW: Shoot dry weight (g), RFW: Root fresh weight (g), RDW: Root dry weight (g), TDM: Total dry matter (g), VRTL: Vertical root length (cm), NL: Number of leaves per plant*, Pn*: Net photosynthesis (µmol CO₂ m ⁻ ² s ⁻ ¹), *E*: Transpiration rate (mol H₂O m ⁻ ² s ⁻ ¹)*, g*_*s*_: Stomatal conductance (mmol m ⁻ ² s ⁻ ¹), RWC: Percent relative leaf water content, AGR: Absolute growth rate, SPAD: SPAD value of leaves, CHA: Chlorophyll *a* (mg g ⁻ ¹ FW), CHB: Chlorophyll *b* (mg g ⁻ ¹ FW), CRTN: Total carotenoids (mg g ⁻ ¹ FW), PRLF: Proline content in leaf (mg g ⁻ ¹ FW), PRRT: Proline content in root (mg g ⁻ ¹ FW), TACL: Total antioxidant capacity of leaf (mg eq. AA g ⁻ ¹ FW), TACR: Total antioxidant capacity of root (mg eq. AA g ⁻ ¹ FW), LPOL: Lipid peroxidation of leaf (MDA content nmol g ⁻ ¹ FW), and LPOR: Lipid peroxidation of root (MDA content nmol g ⁻ ¹ FW).

Again, PC1 contributed 37.86%, meanwhile PC2 contributed 17.56% of the total variation under the recovery phase (S3 Table, Fig 7). Positive correlations were observed in VRTL and SFW, CRTN and *Pn*, *g*_*s*_ and *Pn*, RWC and NL, NL and AGR, AGR and RWC, TACR and PRLF, TACR and TACL, LPOL and LPOR, RFW and LPOL, LPOR and RFW (Fig 7), etc. On the contrary, negative associations were detected between LPOL and PH, LPOR and PH, *E* and RFW, *g*_*s*_ and RDW, NL and LPOR, NL and LPOL, RWC and LPOL, RWC and LPOR, CHA and LPOL, CHB and LPOL, CRTN and LPOL, CHA and LPOR, CHB and LPOR, CRTN and LPOR*,* etc. (Fig 7). The first quadrant possessed three genotypes like V3, V4, and V7, whereas the fourth quadrant had only one genotype, TV9 (Fig 7). In Recovery-14 days, several traits, such as CHB, NL, CRTN, AGR, RWC, LPOR, *Pn*, *g*_*s*_, CHA, VRTL, and SFW affected PC1 mostly, whereas TACR, RFW, PRLF, PRRT, *E*, TACL, and LPOL were the main contributing characters for PC2 (S27 and S28 Figs).

### Correlation analysis of the measured traits

**Fig 8:**
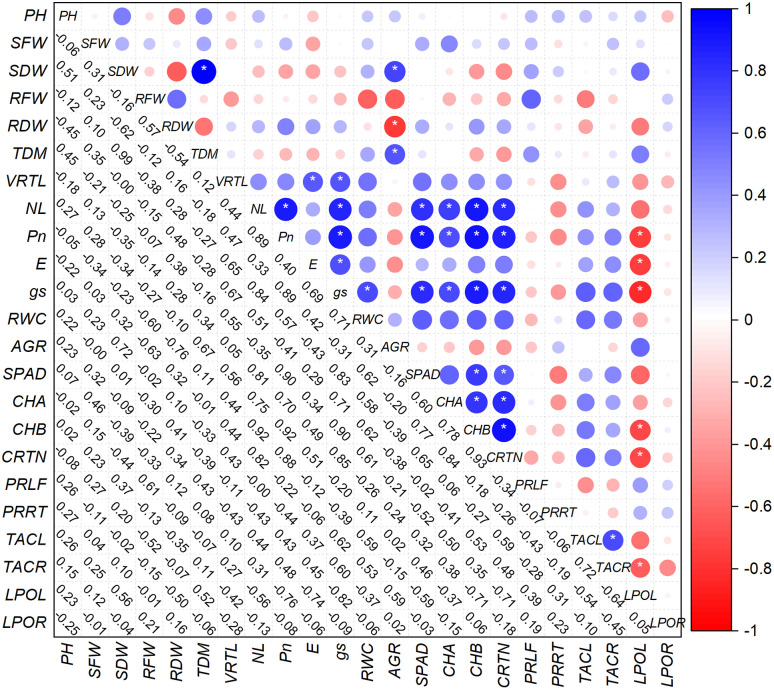
Correlation matrix showing the degree of relationship among the 23 measured traits during the waterlogging phase. The degree of relationship is explained by the color gradient of Pearson’s correlation scale of −1 (deep red color) to 1 (deep blue). PH: Plant height (cm), SFW: Shoot fresh weight (g), SDW: Shoot dry weight (g), RFW: Root fresh weight (g), RDW: Root dry weight (g), TDM: Total dry matter (g), VRTL: Vertical root length (cm), NL: Number of leaves per plant*, Pn*: Net photosynthesis (µmol CO₂ m ⁻ ² s ⁻ ¹), *E*: Transpiration rate (mol H₂O m ⁻ ² s ⁻ ¹)*, g*_*s*_: Stomatal conductance (mmol m ⁻ ² s ⁻ ¹), RWC: Percent relative leaf water content, AGR: Absolute growth rate, SPAD: SPAD value of leaves, CHA: Chlorophyll *a* (mg g ⁻ ¹ FW), CHB: Chlorophyll *b* (mg g ⁻ ¹ FW), CRTN: Total carotenoids (mg g ⁻ ¹ FW), PRLF: Proline content in leaf (mg g ⁻ ¹ FW), PRRT: Proline content in root (mg g ⁻ ¹ FW), TACL: Total antioxidant capacity of leaf (mg eq. AA g ⁻ ¹ FW), TACR: Total antioxidant capacity of root (mg eq. AA g ⁻ ¹ FW), LPOL: Lipid peroxidation of leaf (MDA content nmol g ⁻ ¹ FW), and LPOR: Lipid peroxidation of root (MDA content nmol g ⁻ ¹ FW). The ‘*’ stands for the level of significance at p ≤ ‘5%’.

Several positive and negative correlations were noticed within the traits under waterlogging phase (Fig 8). Diversified significant, relationship such as, SDW positively with TDM and AGR; RDW negatively with AGR; TDM positively with AGR; VRTL positively with *E* and *g*_*s*_; NL positively with physiological parameters (*Pn*, *g*_*s*_, SPAD) and plant pigments (CHA, CHB and CRTN) but negatively with LPOL; *Pn* positively with *g*_*s*_ and plant pigments but negatively with LPOL; *E* positively with *g*_*s*_ but negatively with LPOL; *g*_*s*_ positively with RWC and plant pigments but negatively with LPOL; SPAD positively with plant pigments; plant pigments positively with each other; and TACL positively with TACR but negatively with LPOL; TACR negatively with both LPOL were observed after waterlogging phase.

**Fig 9:**
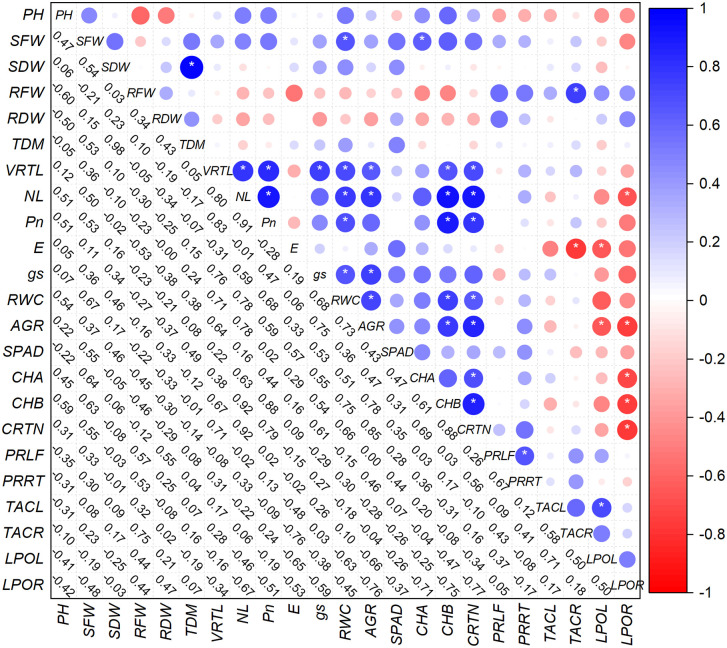
Correlation matrix showing the degree of relationship among the 23 measured traits during the recovery phase. The degree of relationship is explained by the color gradient of Pearson’s correlation scale of −1 (deep red color) to 1 (deep blue). PH: Plant height (cm), SFW: Shoot fresh weight (g), SDW: Shoot dry weight (g), RFW: Root fresh weight (g), RDW: Root dry weight (g), TDM: Total dry matter (g), VRTL: Vertical root length (cm), NL: Number of leaves per plant*, Pn*: Net photosynthesis (µmol CO₂ m ⁻ ² s ⁻ ¹), *E*: Transpiration rate (mol H₂O m ⁻ ² s ⁻ ¹)*, g*_*s*_: Stomatal conductance (mmol m ⁻ ² s ⁻ ¹), RWC: Percent relative leaf water content, AGR: Absolute growth rate, SPAD: SPAD value of leaves, CHA: Chlorophyll *a* (mg g ⁻ ¹ FW), CHB: Chlorophyll *b* (mg g ⁻ ¹ FW), CRTN: Total carotenoids (mg g ⁻ ¹ FW), PRLF: Proline content in leaf (mg g ⁻ ¹ FW), PRRT: Proline content in root (mg g ⁻ ¹ FW), TACL: Total antioxidant capacity of leaf (mg eq. AA g ⁻ ¹ FW), TACR: Total antioxidant capacity of root (mg eq. AA g ⁻ ¹ FW), LPOL: Lipid peroxidation of leaf (MDA content nmol g ⁻ ¹ FW), and LPOR: Lipid peroxidation of root (MDA content nmol g ⁻ ¹ FW). The ‘*’ stands for the level of significance at p ≤ ‘5%’.

In contrast, various sorts of significant associations were also observed among the described traits under the recovery phase (Fig 9). SFW is notably associated with RWC and CHA; SDW strongly positively with TDM; RFW highly relied on TACR; VRTL positively relied on most of the parameters except *E*, LPOL, and LPOR; NL positively associated with VRTL, several physiological parameters (*Pn*, RWC, AGR), and leaf pigments, while negatively with LPOR; *Pn* positively correlated with RWC and leaf pigments; *g*_*s*_, RWC, and AGR positively with each other; leaf pigments correlated with each other but negatively with LPOR under recovery phase.

### Classification of traits and genotypes

**Fig 10:**
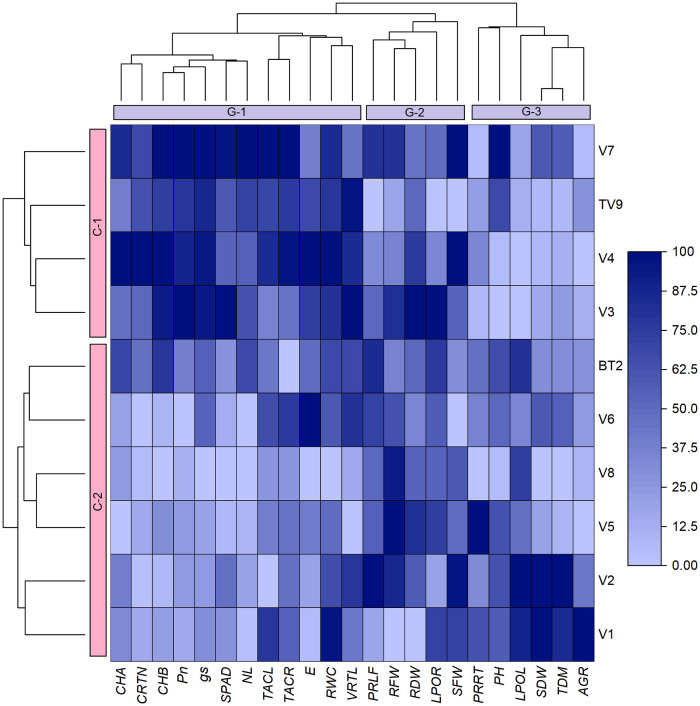
The hierarchical clustering heatmap (two-way) with dendrogram showing the clustering of 23 measured traits and 10 genotypes under the waterlogging phase. The right and left sides of the heatmap illustrate the clusters of the genotypes (C-1 and C-2), while the upper and bottom sides of the heatmap provide the groups of the measured traits (G-1, G-2, and G-3). The STI values are demonstrated by a normalized value on a scale of 0 to 100, with a color gradient from light blue to deep blue. PH: Plant height (cm), SFW: Shoot fresh weight (g), SDW: Shoot dry weight (g), RFW: Root fresh weight (g), RDW: Root dry weight (g), TDM: Total dry matter (g), VRTL: Vertical root length (cm), NL: Number of leaves per plant*, Pn*: Net photosynthesis (µmol CO₂ m ⁻ ² s ⁻ ¹), *E*: Transpiration rate (mol H₂O m ⁻ ² s ⁻ ¹)*, g*_*s*_: Stomatal conductance (mmol m ⁻ ² s ⁻ ¹), RWC: Percent relative leaf water content, AGR: Absolute growth rate, SPAD: SPAD value of leaves, CHA: Chlorophyll *a* (mg g ⁻ ¹ FW), CHB: Chlorophyll *b* (mg g ⁻ ¹ FW), CRTN: Total carotenoids (mg g ⁻ ¹ FW), PRLF: Proline content in leaf (mg g ⁻ ¹ FW), PRRT: Proline content in root (mg g ⁻ ¹ FW), TACL: Total antioxidant capacity of leaf (mg eq. AA g ⁻ ¹ FW), TACR: Total antioxidant capacity of root (mg eq. AA g ⁻ ¹ FW), LPOL: Lipid peroxidation of leaf (MDA content nmol g ⁻ ¹ FW), and LPOR: Lipid peroxidation of root (MDA content nmol g ⁻ ¹ FW).

**Fig 11:**
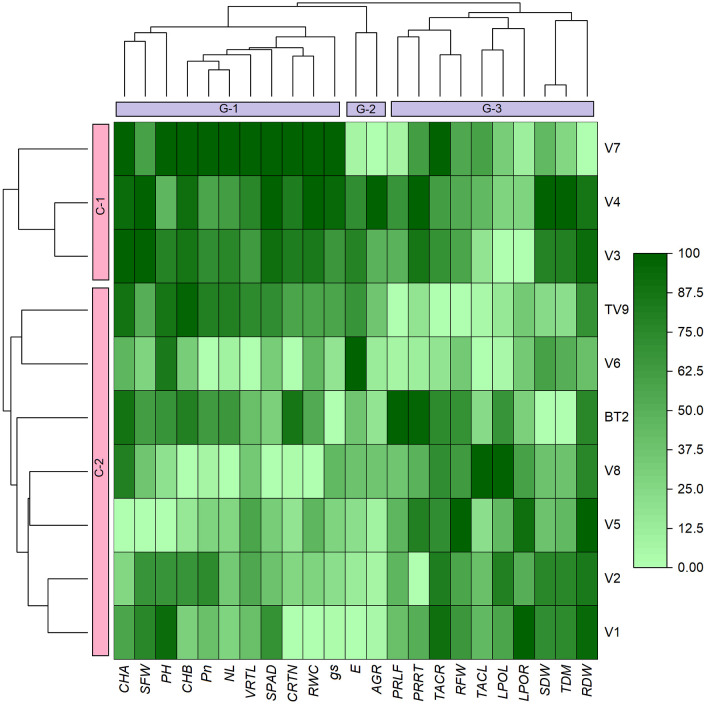
The hierarchical clustering heatmap (two-way) with dendrogram showing the clustering of 23 measured traits and 10 genotypes under the recovery phase. The right and left sides of the heatmap illustrate the clusters of the genotypes (C-1 and C-2), while the upper and bottom sides of the heatmap provide the groups of the measured traits (G-1, G-2, and G-3). The STI values are represented by a normalized value on a scale of 0 to 100, with a color gradient from light green to deep green. PH: Plant height (cm), SFW: Shoot fresh weight (g), SDW: Shoot dry weight (g), RFW: Root fresh weight (g), RDW: Root dry weight (g), TDM: Total dry matter (g), VRTL: Vertical root length (cm), NL: Number of leaves per plant*, Pn*: Net photosynthesis (µmol CO₂ m ⁻ ² s ⁻ ¹), *E*: Transpiration rate (mol H₂O m ⁻ ² s ⁻ ¹)*, g*_*s*_: Stomatal conductance (mmol m ⁻ ² s ⁻ ¹), RWC: Percent relative leaf water content, AGR: Absolute growth rate, SPAD: SPAD value of leaves, CHA: Chlorophyll *a* (mg g ⁻ ¹ FW), CHB: Chlorophyll *b* (mg g ⁻ ¹ FW), CRTN: Total carotenoids (mg g ⁻ ¹ FW), PRLF: Proline content in leaf (mg g ⁻ ¹ FW), PRRT: Proline content in root (mg g ⁻ ¹ FW), TACL: Total antioxidant capacity of leaf (mg eq. AA g ⁻ ¹ FW), TACR: Total antioxidant capacity of root (mg eq. AA g ⁻ ¹ FW), LPOL: Lipid peroxidation of leaf (MDA content nmol g ⁻ ¹ FW), and LPOR: Lipid peroxidation of root (MDA content nmol g ⁻ ¹ FW).

All the measured traits and genotypes in the current study were classified into several clusters and groups by performing a two-way hierarchical clustering heatmap with a dendrogram for both the waterlogging phase (Fig 10) and the recovery phase (Fig 11) using stress tolerance index (STI) values. After the end of the waterlogging phase, the measured traits were grouped into three groups (G-1, G-2, and G-3) (Fig 10). G-1 consisted of CHA, CRTN, CHB, *Pn*, *g*_*s*_, SPAD, NL, TACL, TACR, *E*, RWC, and VRTL, while G-2 comprised of PRLF, RFW, RDW, LPOR, SFW, and the rest of the traits placed in G-3. The genotypes were placed into two clusters (C-1 and C-2), C-1 included the genotypes V7, TV9, V4 and V3; meanwhile, rest of the genotypes (BT2, V6, V8, V5, V2, V1) were kept in C-2 cluster in waterlogging phase (Fig 10).

All the described characters were grouped into three groups at the recovery phase (Fig 11). The highest number (11) of characters were placed into G-1, like CHA, SFW, PH, CHB, *Pn*, NL, VRTL, SPAD, CRTN, RWC, and *g*_*s*_. G-2 consisted of only two physiological traits: *E* and AGR, whereas the remaining ten characters were allocated in the G-3 group. The genotypes were also classified into two clusters (C-1 and C-2) during the recovery phase. C-1 consisted of the genotypes V7, V4 and V3, while remaining seven genotypes (TV9, V6, BT2, V8, V5, V2, V1) were placed in C-2 (Fig 11).

## Discussion

### Morphological changes during waterlogging stress

Plants undergo various morphological changes under waterlogged conditions. Generally, the effects of waterlogging start in the root zone, including limited root growth, varying root length, root discoloration and decay, etc. Frequent adaptive responses are observed in some cases, like formation of aerenchyma tissue, development of adventitious roots, anaerobic respiration due to limited oxygen, accumulation of hormones and osmoprotectants, etc. [46,47]. In the current investigation, the insignificant interaction effect (genotype × experimental conditions) of root dry weight (RDW) was observed (S1 Table), similar findings were also noticed in soybean under waterlogging stress [48], as well as many other crops [49]. This insignificant effect could result from simultaneous changes in waterlogged roots, including root decay, varying root length, and the formation of adventitious roots. Waterlogging also affects root length and plasticity in many crops [50,51]. In the current study, V4 and V7 showed less reduction in vertical root length during the waterlogging and recovery phases, respectively, compared to the control condition (Figs 3G, 10, and 11). Roots provide nutrients, minerals, etc. to the whole plant, and studies about root systems are very important during waterlogging stress because changes in root morphology have a direct impact on shoot growth parameters, *i.e.,* plant height, biomass accumulation, leaf area, leaf number, etc. [52]. Under waterlogging, plants suffer from inadequate photosynthates, which disrupts cell growth, fresh weights, and dry matter accumulation [53]. In the current study, PH, SFW, SDW, RFW, RDW, and TDM decreased with increasing stress duration (Figs 2, 3A-F, 10, and 11), and this declining trend was consistent with many studies [54,55]. Leaf pigment degradation reduced physiological activities, and a decline in the leaf number was observed under waterlogging stress [56]. Maximum leaf numbers were observed in TV9 throughout this experiment, although V7 exhibited the lowest reduction [Fig S9, Figs 3H, 10, and 11], and reduced leaf numbers were observed in other studies [57]. The reduction in (morphological) growth of tea under different stresses has been reported in many studies [58]. Prolonged waterlogging reduced organic matter content and the availability of other nutrients in tea soil [26]. However, during the post-stress condition, plants regained their capacity to absorb nutrients more efficiently than under the waterlogged condition, which subsequently promoted the restoration of vegetative growth [30].

### Fluctuations in physiological traits during waterlogging stress

Waterlogging stress impairs root respiration due to oxygen deficiency, causing osmotic imbalance [59]. This situation stimulates stomatal closure (lower stomatal conductance), thereby restricting water loss and, in turn, preventing the movement of carbon dioxide (CO₂). This limited CO₂ reduces photosynthetic activity and transpiration as well [60]. Reduced net photosynthesis, transpiration rate, and stomatal conductance were also observed as immediate responses in various experiments under waterlogging stress in tea [13,19,24] like the current findings (Figs 4A-C, 10, and 11). Waterlogging-tolerant tea cultivar SNT-10 exhibited the least reductions in *Pn*, *E,* and *g*_*s*_ by 53.6%, 43.8%, and 57.9%, respectively, under prolonged waterlogging compared to the other cultivars [19]. Excess moisture stress also reduces cell expansion, which ultimately lowers relative water content (RWC) [61]. The lowest degree of reduction in RWC was noticed in V7 at recovery phase (Figs 4D, 10, and 11) which was consistent with many previous findings [62]. Although the absolute growth rate (AGR) had an insignificant effect on genotypes, the conditions and interaction effects were significant, indicating that AGR varied significantly across experimental conditions (S1 Table). The growth rate depends on dry matter accumulation, which decreases under waterlogging stress [63]. Leaf greenness (SPAD indices) was also reduced during stress in the current study (Fig 2), but the minimum decrease was observed in V3 (at the waterlogging phase) and V7 (at the recovery phase) (Figs 4F, 10, and 11). A similar trend was also reported in many studies, and the decreased SPAD value was attributed to oxidative stress-induced damage of chloroplasts under stress conditions [61]. Plant experiences reoxygenation by removing excess water from the root zone during recovery phases [30]. The resupply of oxygen to the root zone improves gas exchange and photosynthetic efficiency, thereby restoring aerobic respiration and other physiological activities. Here, the restoration of physiological activities was also detected during recovery, which is consistent with previous studies [30,64].

### Waterlogging stress and alterations of biochemical parameters

Photosynthetic pigments, like chlorophylls, are essential pigments that facilitate photosynthesis by utilizing atmospheric CO₂ and water molecules to convert them into glucose and O₂ in the presence of sunlight [65]. Chlorophyll *a* and *b* are the major photosynthetic pigments, where chlorophyll *a* is responsible for the majority of photosynthesis and chlorophyll *b* acts as a supporting pigment [66]. Carotenoids are another important group of leaf pigments that protect plants from oxidative stress caused by ROS [67]. A Decline in leaf pigments under waterlogging has also been reported in many crops, including tea [13,24,68,69] as similar with this investigation (Figs 5A-C, 10, and 11). An Indian tea cultivar, SNT-10, demonstrated superior waterlogging tolerance compared to other cultivars, showing the least decline in chlorophyll *a* (31.4%) and chlorophyll *b* (8.9%) under prolonged and excess soil moisture conditions [19].

Proline, an essential amino acid, acts as an osmolyte that increases in response to waterlogging stress to maintain water balance in plants [70]. Tolerant plants synthesize more proline as a defense mechanism to neutralize the ROS [71]. The highest proline accumulation (3.78 µmol g ⁻ ¹ FW) was recorded among 29 tea cultivars after prolonged waterlogging [24]. Interestingly, the proline content decreases under recovery phase, which may be the removal of stress condition, *i.e.*, resupply of oxygen. The presence of oxygen interacts with glutamate pathway and pyrroline-5-carboxylate synthetase (*P5CS*) enzyme to reduce the proline content. The decreasing trend in proline under recovery phase was detected here (Figs 5D, 5E, 10, and 11) which was also reported in other crops [70]. Antioxidants play vital roles in counteracting oxidative stress caused by ROS and free radicals under excess moisture stress [72]. In the current investigation, V7 showed the greatest percentage increase in total antioxidant capacity under waterlogging compared to the control in both leaves and roots (Figs 5F, 5G, 10, and 11). Higher total antioxidant activity in tolerant species was also noticed in different stress conditions across various experiments [36,40,71]. Under stress conditions, cell membrane degrades, disrupting cellular integrity and making the cell more permeable, thereby unbalancing water-nutrient transport [73]. The level of malondialdehyde (MDA) increases in waterlogging stress, a key indicator of cellular damage, and the genotype with the least increase of MDA appears to be more tolerant [62,73]. Significant changes in MDA contents were observed in *Musa acuminata,* ranging from 23 to 25 nmol g^-1^ FW after 14 days of waterlogging phase [74]. As with proline content, the concentration of MDA in this experiment also decreased under recovery, but the reduction followed a steady trend (Figs 5H, 5I, 10, and 11). A similarly slower decrease in MDA with resupply of oxygen was also reported in previous investigations [75].

### Degree of relationship and identification of major traits under waterlogging stress

**Fig 12:**
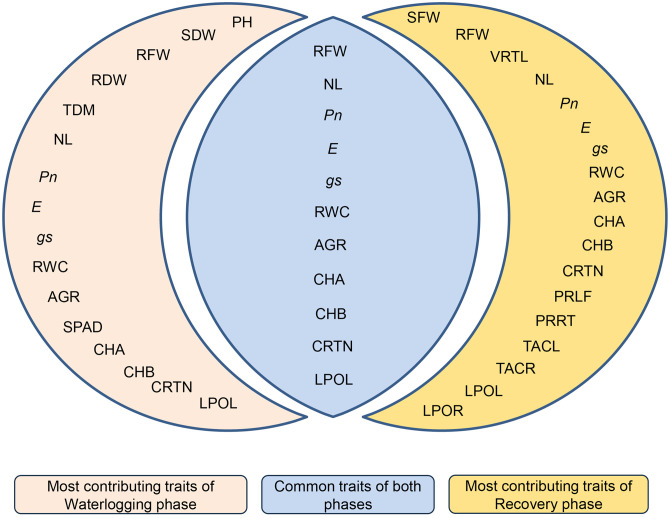
Venn diagram showing the most contributing traits under waterlogging and recovery phases. The left and right fragments of the diagram indicated the traits mostly related to waterlogging and recovery phases, respectively, while the overlapping (middle) fragment in the center represented the common contributors under both phases. PH: Plant height (cm), SFW: Shoot fresh weight (g), SDW: Shoot dry weight (g), RFW: Root fresh weight (g), RDW: Root dry weight (g), TDM: Total dry matter (g), VRTL: Vertical root length (cm), NL: Number of leaves per plant*, Pn*: Net photosynthesis (µmol CO₂ m ⁻ ² s ⁻ ¹), *E*: Transpiration rate (mol H₂O m ⁻ ² s ⁻ ¹)*, g*_*s*_: Stomatal conductance (mmol m ⁻ ² s ⁻ ¹), RWC: Percent relative leaf water content, AGR: Absolute growth rate, SPAD: SPAD value of leaves, CHA: Chlorophyll *a* (mg g ⁻ ¹ FW), CHB: Chlorophyll *b* (mg g ⁻ ¹ FW), CRTN: Total carotenoids (mg g ⁻ ¹ FW), PRLF: Proline content in leaf (mg g ⁻ ¹ FW), PRRT: Proline content in root (mg g ⁻ ¹ FW), TACL: Total antioxidant capacity of leaf (mg eq. AA g ⁻ ¹ FW), TACR: Total antioxidant capacity of root (mg eq. AA g ⁻ ¹ FW), LPOL: Lipid peroxidation of leaf (MDA content nmol g ⁻ ¹ FW), and LPOR: Lipid peroxidation of root (MDA content nmol g ⁻ ¹ FW).

The types and degrees of relationship among the measured traits were explained by PC analysis (Figs 6 and 7) and a correlation matrix (Figs 8 and 9). PH was positively correlated with SFW and SDW but negatively correlated with RFW and RDW, reflecting the contrasting growth patterns between the shoot and root systems, as supported by previous studies [76,77]. Under waterlogging phase, PH positively associated with PRLF, PRRT, TACL, TACR, LPOL, whereas the relationship became negative during recovery conditions. This trend indicates that the accumulation of proline, antioxidants, and MDA increased under waterlogged conditions but declined upon recovery, while PH continued to increase over time. Under waterlogged conditions, TDM negatively correlated with *Pn*, *E*, *g*_*s*_ but showed a positive association during recovery, as reported by others [8,55]. During the waterlogging phase, PH, SDW, RFW, RDW, TDM, NL, *Pn*, *E*, *g*_*s*_, RWC, AGR, SPAD, CHA, CHB, CRTN, and LPOL were main influential traits (S25 and S26 Figs), whereas SFW, RFW, VRTL, NL, *Pn*, *E*, *g*_*s*_, RWC, AGR, CHA, CHB, CRTN, PRLF, PRRT, TACL, TACR, LPOL, and LPOR were the main contributing traits in the recovery phase (S27 and S28 Figs). Notably, 11 traits, *i.e.,* RFW, NL, *Pn*, *E*, *g*_*s*_, RWC, AGR, CHA, CHB, CRTN, and LPOL, were common to both phases, suggesting that these parameters are the most influential indicators of waterlogging response in tea (Fig 12). Physiological traits like *Pn*, *E*, and *g*_*s*_*,* have also been recommended by previous studies for assessing stress tolerance in tea [13,19,24,78], while proline and MDA contents were similarly important for waterlogging tolerance assessment [13,19,24,72–74]. Again, a consistent number of leaves (NL) and leaf pigments (CHA and CHB) are directly related to adaptive responses, which help accumulate photosynthates and maintain better physiological growth under waterlogging stress [3].

### Screening of tolerant tea genotypes for waterlogging stress

A waterlogging-induced stress condition, coupled with the post-waterlogging recovery phase, provides a comprehensive approach to understanding a plant’s resilience and adaptive capabilities during waterlogging. Under an excess moisture situation, plants trigger their tolerance mechanisms to withstand the stress, but in recovery phase, they focus on restoring their original normal state once the stress is alleviated [79]. Recovery performance studies across different periods after eliminating waterlogging stress were also conducted in various crops, such as barley [80], coffee [81], avocado [82], and banana [83]. Tea plants exhibit immediate morphological, physiological, and biochemical responses to waterlogging, but recovery varies by genotype, and the effects generally persist for longer [27,84]. In the present experiment, waterlogging-tolerant tea genotypes were identified using PC analysis (Figs 6 and 7) and hierarchical clustering heatmaps (Figs 10 and 11) using STI values of each measured trait under both waterlogging and recovery phases. PCA helps to identify promising genotypes by reducing magnitude of the variables [85,86], whereas a heatmap visualizes the responses of genotypes and groups them accordingly [73,85].

**Fig 13:**
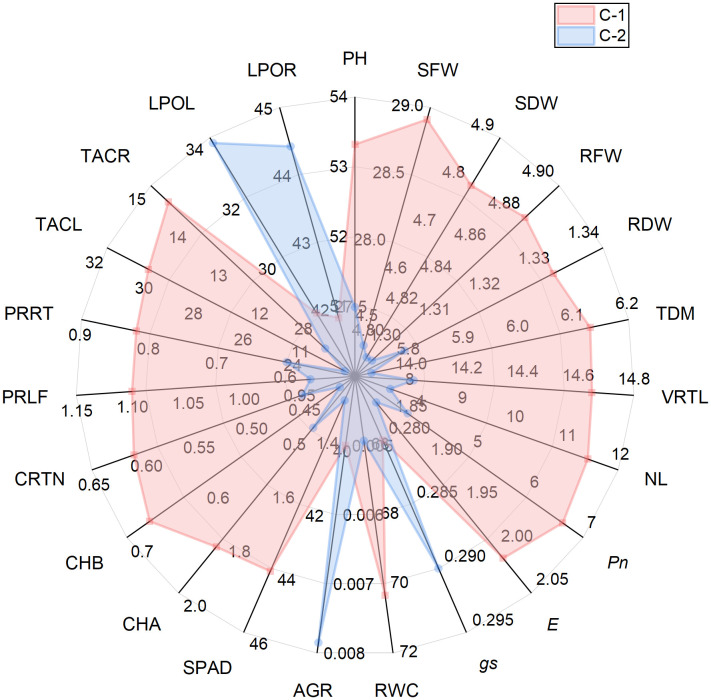
Comparative responses of two different clusters (C-1 and C-2) during the waterlogging phase. The radar plot demonstrated variation in morphological, physiological and biochemical traits, where the pink and blue shaded regions represented the responses of clusters C-1 and C-2, respectively. PH: Plant height (cm), SFW: Shoot fresh weight (g), SDW: Shoot dry weight (g), RFW: Root fresh weight (g), RDW: Root dry weight (g), TDM: Total dry matter (g), VRTL: Vertical root length (cm), NL: Number of leaves per plant*, Pn*: Net photosynthesis (µmol CO₂ m ⁻ ² s ⁻ ¹), *E*: Transpiration rate (mol H₂O m ⁻ ² s ⁻ ¹)*, g*_*s*_: Stomatal conductance (mmol m ⁻ ² s ⁻ ¹), RWC: Percent relative leaf water content, AGR: Absolute growth rate, SPAD: SPAD value of leaves, CHA: Chlorophyll *a* (mg g ⁻ ¹ FW), CHB: Chlorophyll *b* (mg g ⁻ ¹ FW), CRTN: Total carotenoids (mg g ⁻ ¹ FW), PRLF: Proline content in leaf (mg g ⁻ ¹ FW), PRRT: Proline content in root (mg g ⁻ ¹ FW), TACL: Total antioxidant capacity of leaf (mg eq. AA g ⁻ ¹ FW), TACR: Total antioxidant capacity of root (mg eq. AA g ⁻ ¹ FW), LPOL: Lipid peroxidation of leaf (MDA content nmol g ⁻ ¹ FW), and LPOR: Lipid peroxidation of root (MDA content nmol g ⁻ ¹ FW).

In the waterlogging phase, the genotypes were grouped into two clusters (C-1 and C-2) (Fig 10). It can be easily noticed that the C-1 cluster, which comprised of V7, TV9, V4, and V3 genotypes, exhibited superior responses in the waterlogging phase compared to C-2 for maximum traits (Fig 13). Based on the clustering dendrogram (Fig 10), the performance hierarchy within this cluster was V7 > TV9 > V4 > V3. The same grouping pattern is also observed in PCA-biplot (Fig 6), where V7, TV9, V4, and V3 genotypes were kept on the same right side of the biplot (1^st^ and 4^th^ quadrants). Although V4 and V3 genotypes performed well, they did not surpass the control variety, TV9. On the other hand, the genotypes in C-2 showed a weaker response to waterlogging with a sequence of (BT2 > V6 > V8 > V5 > V2 > V1 which was also placed in a similar left side (2^nd^ and 3^rd^ quadrants) of PCA-biplot (Fig 6). Therefore, it was observed that only one genotype (V7) was identified as most superior, whereas V1 was the weakest one in the waterlogging phase.

**Fig 14:**
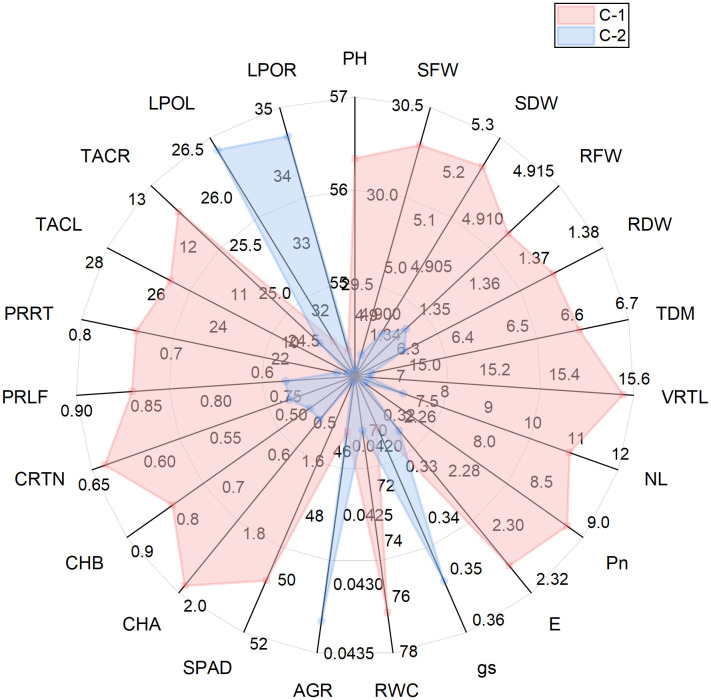
Comparative responses of tea genotype clusters (C-1 and C-2) during the recovery phase. The pink and blue areas indicated the responses of C-1 and C-2, respectively. PH: Plant height (cm), SFW: Shoot fresh weight (g), SDW: Shoot dry weight (g), RFW: Root fresh weight (g), RDW: Root dry weight (g), TDM: Total dry matter (g), VRTL: Vertical root length (cm), NL: Number of leaves per plant*, Pn*: Net photosynthesis (µmol CO₂ m ⁻ ² s ⁻ ¹), *E*: Transpiration rate (mol H₂O m ⁻ ² s ⁻ ¹)*, g*_*s*_: Stomatal conductance (mmol m ⁻ ² s ⁻ ¹), RWC: Percent relative leaf water content, AGR: Absolute growth rate, SPAD: SPAD value of leaves, CHA: Chlorophyll *a* (mg g ⁻ ¹ FW), CHB: Chlorophyll *b* (mg g ⁻ ¹ FW), CRTN: Total carotenoids (mg g ⁻ ¹ FW), PRLF: Proline content in leaf (mg g ⁻ ¹ FW), PRRT: Proline content in root (mg g ⁻ ¹ FW), TACL: Total antioxidant capacity of leaf (mg eq. AA g ⁻ ¹ FW), TACR: Total antioxidant capacity of root (mg eq. AA g ⁻ ¹ FW), LPOL: Lipid peroxidation of leaf (MDA content nmol g ⁻ ¹ FW), and LPOR: Lipid peroxidation of root (MDA content nmol g ⁻ ¹ FW).

Again, in the recovery phase, genotypes were also categorized into cluster-1 (C-1) and cluster-2 (C-2) (Fig 11). C-1 comprised the genotypes that recovered most effectively than C-2 (Fig 14). The order of recovery performance was V7 > V4 > V3 (Fig 11), which was also located in the same (1^st^) quadrant of PCA-biplot (Fig 7). Cluster C-2 consisted of seven genotypes with the sequence TV9 > V6 > BT2 > V8 > V5 > V2 > V1, which exhibited relatively steady recovery compared to control conditions. Interestingly, TV9, which was one of the best performers in waterlogging phase (Fig 10), shifted to C-2 in recovery (Fig 11), suggesting a lower recovery potential. Consistently, V1 remained the most susceptible genotype in both waterlogging and recovery phases. Considering the overall responses under both the waterlogging and recovery phases, V7 (P/AFN/13/90) was identified as the most tolerant, demonstrating superior stress endurance and rapid recovery than other genotypes.

**Fig 15:**
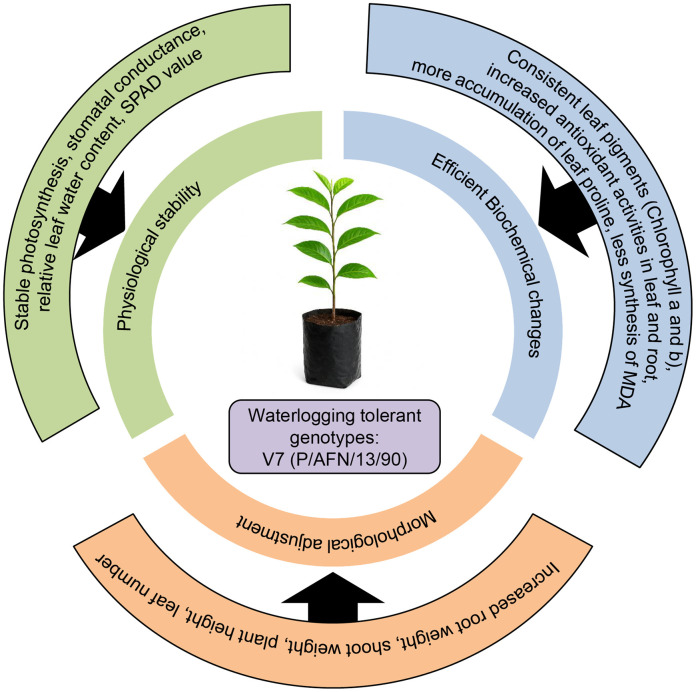
Generalized tolerance pathways of waterlogging-tolerant tea genotype V7 (P/AFN/13/90).

The genotype V7 (P/AFN/13/90) exhibited superior tolerance against waterlogging through coordinated morpho-physiological and biochemical adjustments. It maintained relatively higher and more stable photosynthesis, transpiration, stomatal conductance, and chlorophyll content than other genotypes, thereby providing greater energy under stress conditions. Increased proline and antioxidant activity helped to maintain osmotic balance and neutralize ROS as an adaptive response, while lower MDA levels indicated higher membrane integrity. Moreover, sustained and greater root growth, relative leaf water content, and leaf number, along with an overall higher growth rate, indicated the superior tolerance of V7. During the recovery phase, V7 also demonstrated a comparatively faster restoration of morphological growth traits, indicating lower stress-induced damage. The rapid recovery of photosynthetic efficiency, gaseous exchange capacity, and biochemical fixation also confirmed its stronger resilience after removal of excess water. Collectively, these responses contributed to increased tolerance of V7 to waterlogging through better adaptation with faster recovery potential; therefore, a hypothetical tolerance mechanism pathway is illustrated in Fig 15. This waterlogging tolerance pathway is in line with the findings of previous several studies [27,87].

## Conclusion

The tea plant showed a significant response to waterlogging stress, and evaluating tea genotypes for tolerance was more effective when both the waterlogging and recovery phases were considered together. All morpho-physiological traits and some biochemical parameters decreased, whereas proline content, total antioxidant activity, and lipid peroxidation increased notably under stress conditions. Among the measured traits, root fresh weight, number of leaves, net photosynthesis, transpiration rate, stomatal conductance, percent relative leaf water content, absolute growth rate, leaf pigments contents, and leaf lipid peroxidation were identified as the most effective stress indicator traits for the assessment of waterlogging tolerance of tea. Finally, among the tested genotypes, V7 (P/AFN/13/90) was identified as the most promising and potentially effective against waterlogging stress. However, further genetic characterization and extensive field trials are required to validate the results under variable environmental conditions.

## Supporting information

S1 Table Analysis of variances (ANOVA) of 23 measured traits in 10 tea genotypes for waterlogging tolerance. (DOC)

S2 Table Eigenvalue, percent (%) variance, and cumulative percent (%) variance of corresponding principal components (PCs) of 10 genotypes for 23 traits under waterlogging phase. (DOC)

S3 Table Eigenvalue, percent (%) variance, and cumulative percent (%) variance of corresponding principal components (PCs) of 10 genotypes for 23 traits under recovery phase. (DOC)

S1 Fig Mean temperature (° C) and relative humidity (%) of experimental conditions. (TIF)

S2 Fig Variations in plant height (cm) of 10 genotypes in different experimental conditions. (TIF)

S3 Fig Variations in shoot fresh weight (g) of 10 genotypes in experimental conditions. (TIF)

S4 Fig Variations in shoot dry weight (g) of 10 genotypes in both stress and control conditions. (TIF)

S5 Fig Variations in root fresh weight (g) of 10 genotypes in different experimental conditions. (TIF)

S6 Fig Variations in root dry weight (g) of 10 genotypes in both stress and control conditions. (TIF)

S7 Fig Variations in total dry matter (g) of 10 genotypes in different experimental conditions. (TIF)

S8 Fig Variations in vertical root length (cm) of 10 genotypes in both stress and control conditions. (TIF)

S9 Fig Variations in number of leaves per plant of 10 genotypes in different experimental conditions. (TIF)

S10 Fig Variations in net photosynthesis (µmol CO_2_ m⁻ ^⁻ 2^ s⁻ ^⁻1^) of 10 genotypes in different experimental conditions. (TIF)

S11 Fig Variations in transpiration rate (mol H_2_O m⁻ ^⁻ 2^ s⁻ ^⁻ 1^) of 10 genotypes in different experimental conditions. (TIF)

S12 Fig Variations in stomatal conductance (mmol m⁻ ^⁻ 2^ s⁻ ^⁻ 1^) of 10 genotypes in both stress and control conditions. (TIF)

S13 Fig Variations in relative leaf water content (%) of 10 genotypes in different experimental conditions. (TIF)

S14 Fig Variations in absolute growth rate of 10 genotypes in both stress and control conditions. (TIF)

S15 Fig Variations in SPAD value of leaves of 10 genotypes in different experimental conditions. (TIF)

S16 Fig Variations in chlorophyll a (mg g^⁻ 1^ FW) of 10 genotypes in both stress and control conditions. (TIF)

S17 Fig Variations in chlorophyll b (mg g^⁻ 1^ FW) of 10 genotypes in different experimental conditions. (TIF)

S18 Fig Variations in total carotenoids (mg g^⁻ 1^ FW) of 10 genotypes in both stress and control conditions. (TIF)

S19 Fig Variations in proline content in leaf (mg g⁻ ^1^ FW) of 10 genotypes in different experimental conditions. (TIF)

S20 Fig Variations in proline content in root (mg g^⁻ 1^ FW) of 10 genotypes in both stress and control conditions. (TIF)

S21 Fig Variations in total antioxidant capacity of leaf (mg eq. AA g ⁻ ^1^ FW) of 10 genotypes in different experimental conditions. (TIF)

S22 Fig Variations in total antioxidant capacity of root (mg eq. AA g ⁻ ^1^ FW) of 10 genotypes in both stress and control conditions. (TIF)

S23 Fig Variations in lipid peroxidation of leaf (MDA content nmol g⁻ ^⁻ 1^ FW) of 10 genotypes in different experimental conditions. (TIF)

S24 Fig Variations in lipid peroxidation of root (MDA content nmol g^⁻ 1^ FW) of 10 genotypes in different experimental conditions. (TIF)

S25 Fig Contribution of the variables to PC1 under waterlogging phase. (TIF)

S26 Fig Contribution of the variables to PC2 under waterlogging phase. (TIF)

S27 Fig Contribution of the variables to PC1 under recovery phase. (TIF)

S28 Fig Contribution of the variables to PC2 under recovery phase. (TIF)
