# Supplementary material for: Assessment of waterlogging tolerance in tea genotypes through morpho-physiological and biochemical profiling
Source: PLoS One. 2026 Jul 20;21(7):e0354144. doi: 10.1371/journal.pone.0354144 (PMC13384526; doi:10.1371/journal.pone.0354144)
Supplement: S1 Table — (DOC) [file pone.0354144.s001.doc]

S1 Table. Analysis of variances (ANOVA) of 23 measured traits in 10 tea genotypes for waterlogging tolerance

| **Parameters** | **Genotypes**  **(factor-1)** | **Experimental conditions**  **(factor-2)** | **Genotypes x Experimental conditions** | **Residuals** | **LSD**  **values** |
| --- | --- | --- | --- | --- | --- |
|  | **9** | **7** | **63** | **160** | **-** |
| **PH** | 93.67*** | 387.96*** | 1.9* | 1.33 | 4.05 |
| **SFW** | 24.62*** | 37.12*** | 0.37* | 0.25 | 1.76 |
| **SDW** | 4.36*** | 5.35*** | 0.04* | 0.03 | 0.57 |
| **RFW** | 0.44*** | 0.29*** | 0.01* | 0.01 | 0.26 |
| **RDW** | 0.09*** | 0.25*** | 0.002^ns^ | 0.03 | 0.59 |
| **TDM** | 5.31*** | 7.75*** | 0.04* | 0.02 | 0.55 |
| **VRTL** | 6.17*** | 27.6*** | 0.23 ^ns^ | 1.20 | 3.84 |
| **NL** | 61.04*** | 282.94*** | 4.51 ^ns^ | 4.49 | 7.44 |
| ***Pn*** | 11.86*** | 594.41*** | 2.49*** | 1.21 | 3.85 |
| ***E*** | 0.07** | 10.1*** | 0.05*** | 0.02 | 0.55 |
| ***g_s_*** | 0.0431*** | 0.2224*** | 0.0026** | 0.0016 | 0.138 |
| **RWC** | 140.67*** | 1696.33*** | 11.47*** | 3.27 | 6.35 |
| **AGR** | 0.0005^ns^ | 0.0200*** | 0.0009*** | 0.0003 | 0.064 |
| **SPAD** | 307.59*** | 1765.42*** | 19.96*** | 4.86 | 7.74 |
| **CHA** | 1.89*** | 0.55^***^ | 0.02*** | 0.01 | 0.31 |
| **CHB** | 0.64*** | 0.49*** | 0.01*** | 0.001 | 0.16 |
| **CRTN** | 0.11*** | 0.20*** | 0.002*** | 0.001 | 0.14 |
| **PRLF** | 0.18*** | 0.9*** | 0.01* | 0.01 | 0.301 |
| **PRRT** | 0.25*** | 0.43*** | 0.01* | 0.004 | 0.225 |
| **TACL** | 218.55*** | 761.53*** | 5.95* | 3.76 | 6.81 |
| **TACR** | 50.1*** | 168.78*** | 1.78*** | 0.96 | 3.45 |
| **LPOL** | 80.57*** | 3292.39*** | 11.6*** | 2.65 | 5.71 |
| **LPOR** | 76.16*** | 6520.01*** | 20.56*** | 9.69 | 10.93 |

LSD values: Least Significance Difference at 5% level of probability. PH: Plant height, SFW: Shoot fresh weight, SDW: Shoot dry weight, RFW: Root fresh weight, RDW: Root dry weight, TDM: Total dry matter, VRTL: Vertical root length, NL: Number of leaves per plant*, Pn*: net photosynthesis, *E*: Transpiration rate*, g_s_*: Stomatal conductance, RWC: Percent relative leaf water content, AGR: Absolute growth rate, SPAD: SPAD value of leaves, CHA: Chlorophyll *a*, CHB: Chlorophyll *b*, CRTN: Total carotenoids, PRLF: Proline content in leaf, PRRT: Proline content in root, TACL: Total antioxidant capacity of leaf, TACR: Total antioxidant capacity of root, LPOL: Lipid peroxidation of leaf and LPOR: Lipid peroxidation of root. The ‘*’, ‘**’ and ‘***’ stands for level of significance at p≤ ‘5%’, ‘1%’ and ‘0.1%’ respectively, whereas ‘^ns^’ for ‘non-significant’.
