# Supplementary material for: Assessment of waterlogging tolerance in tea genotypes through morpho-physiological and biochemical profiling
Source: PLoS One. 2026 Jul 20;21(7):e0354144. doi: 10.1371/journal.pone.0354144 (PMC13384526; doi:10.1371/journal.pone.0354144)
Supplement: S2 Table — (DOC) [file pone.0354144.s002.doc]

S2 Table. Eigenvalue, percent (%) variance, and cumulative percent (%) variance of corresponding principal components (PCs) of 10 genotypes for 23 traits under waterlogging phase.

| **Principal components (PCs)** | **Eigenvalue** | **Variance (%)** | **Cumulative variance (%)** |
| --- | --- | --- | --- |
| **PC1** | 9.01 | 39.16 | 39.16 |
| **PC2** | 4.29 | 18.65 | 57.81 |
| **PC3** | 2.64 | 11.49 | 69.30 |
| **PC4** | 1.56 | 6.78 | 76.08 |
| **PC5** | 1.43 | 6.24 | 82.32 |
| **PC6** | 1.35 | 5.86 | 88.18 |
| **PC7** | 1.15 | 5.02 | 93.20 |
| **PC8** | 0.82 | 3.53 | 96.73 |
| **PC9** | 0.75 | 3.27 | 100 |
