# Supplementary material for: Assessment of waterlogging tolerance in tea genotypes through morpho-physiological and biochemical profiling
Source: PLoS One. 2026 Jul 20;21(7):e0354144. doi: 10.1371/journal.pone.0354144 (PMC13384526; doi:10.1371/journal.pone.0354144)
Supplement: S3 Table — (DOC) [file pone.0354144.s003.doc]

S3 Table. Eigenvalue, percent (%) variance, and cumulative percent (%) variance of corresponding principal components (PCs) of 10 genotypes for 23 traits under recovery phase.

| **Principal components (PCs)** | **Eigenvalue** | **Variance (%)** | **Cumulative**  **variance (%)** |
| --- | --- | --- | --- |
| **PC1** | 8.71 | 37.86 | 37.86 |
| **PC2** | 4.04 | 17.56 | 55.42 |
| **PC3** | 3.26 | 14.18 | 69.6 |
| **PC4** | 2.19 | 9.53 | 79.13 |
| **PC5** | 1.66 | 7.2 | 86.33 |
| **PC6** | 1.47 | 6.4 | 92.73 |
| **PC7** | 0.95 | 4.13 | 96.86 |
| **PC8** | 0.54 | 2.34 | 99.2 |
| **PC9** | 0.19 | 0.8 | 100 |
