# Supplementary figures and images for: Assessment of waterlogging tolerance in tea genotypes through morpho-physiological and biochemical profiling
Source: PLoS One. 2026 Jul 20;21(7):e0354144. doi: 10.1371/journal.pone.0354144 (PMC13384526; doi:10.1371/journal.pone.0354144)

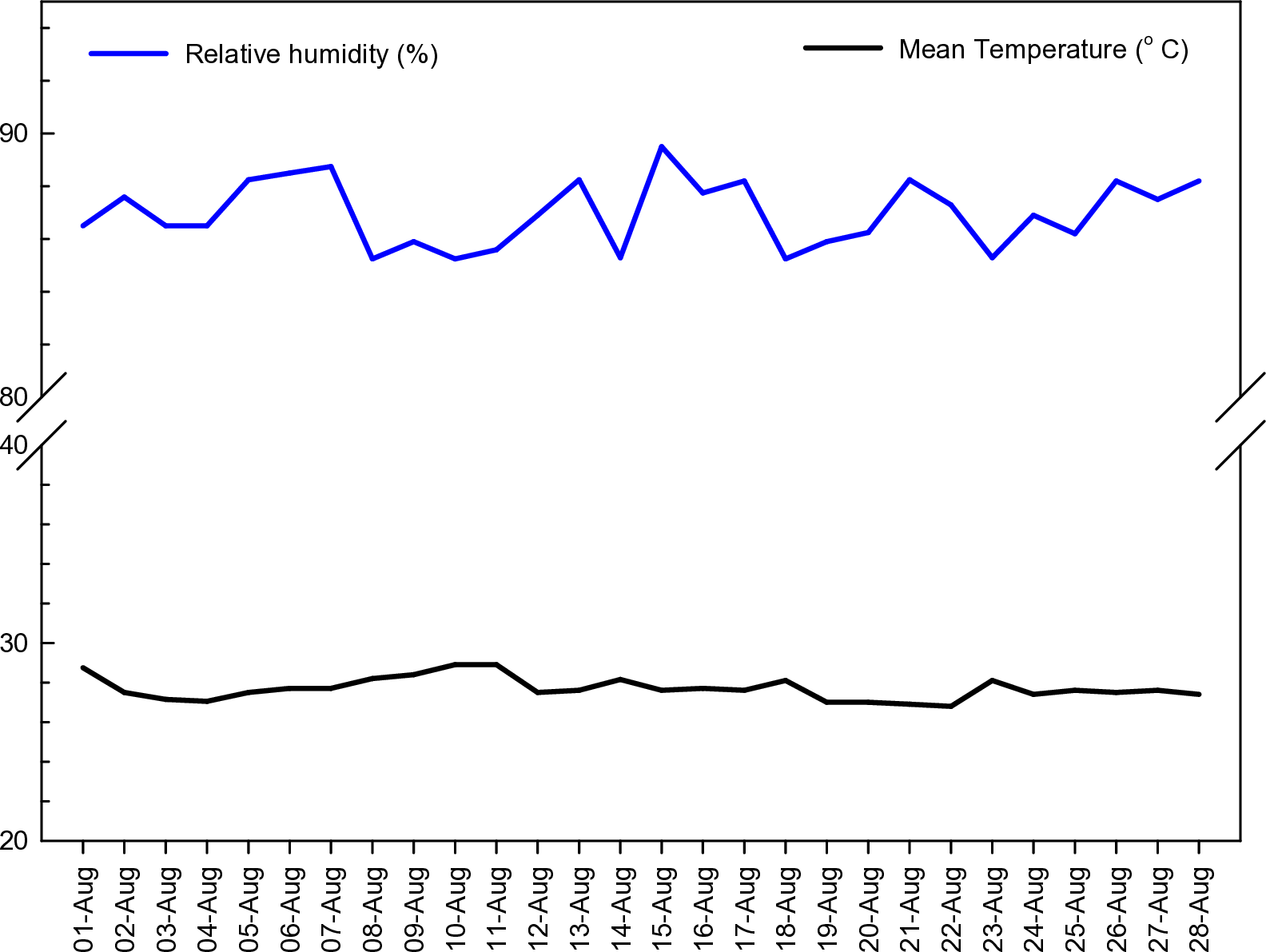

Supplement: S1 Fig — (TIF) [file pone.0354144.s004.tif]

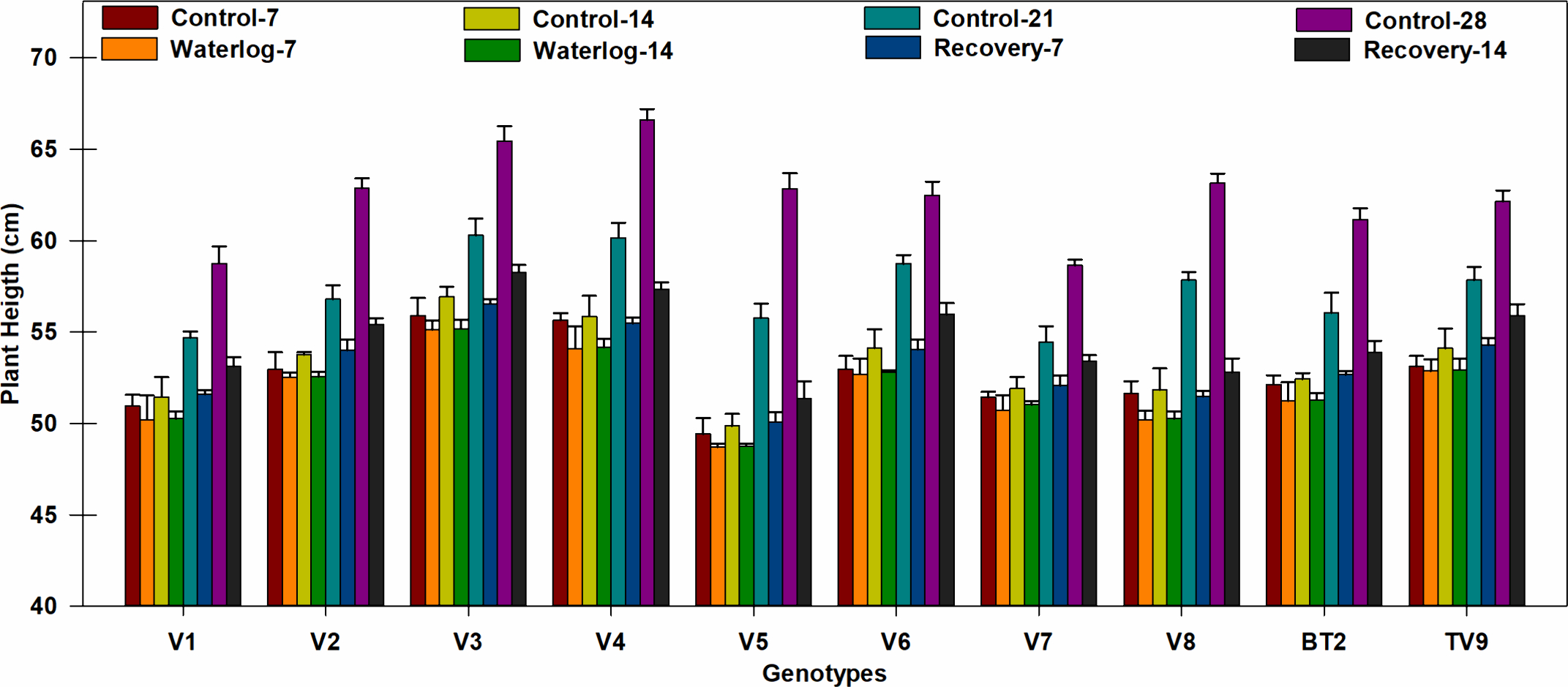

Supplement: S2 Fig — (TIF) [file pone.0354144.s005.tif]

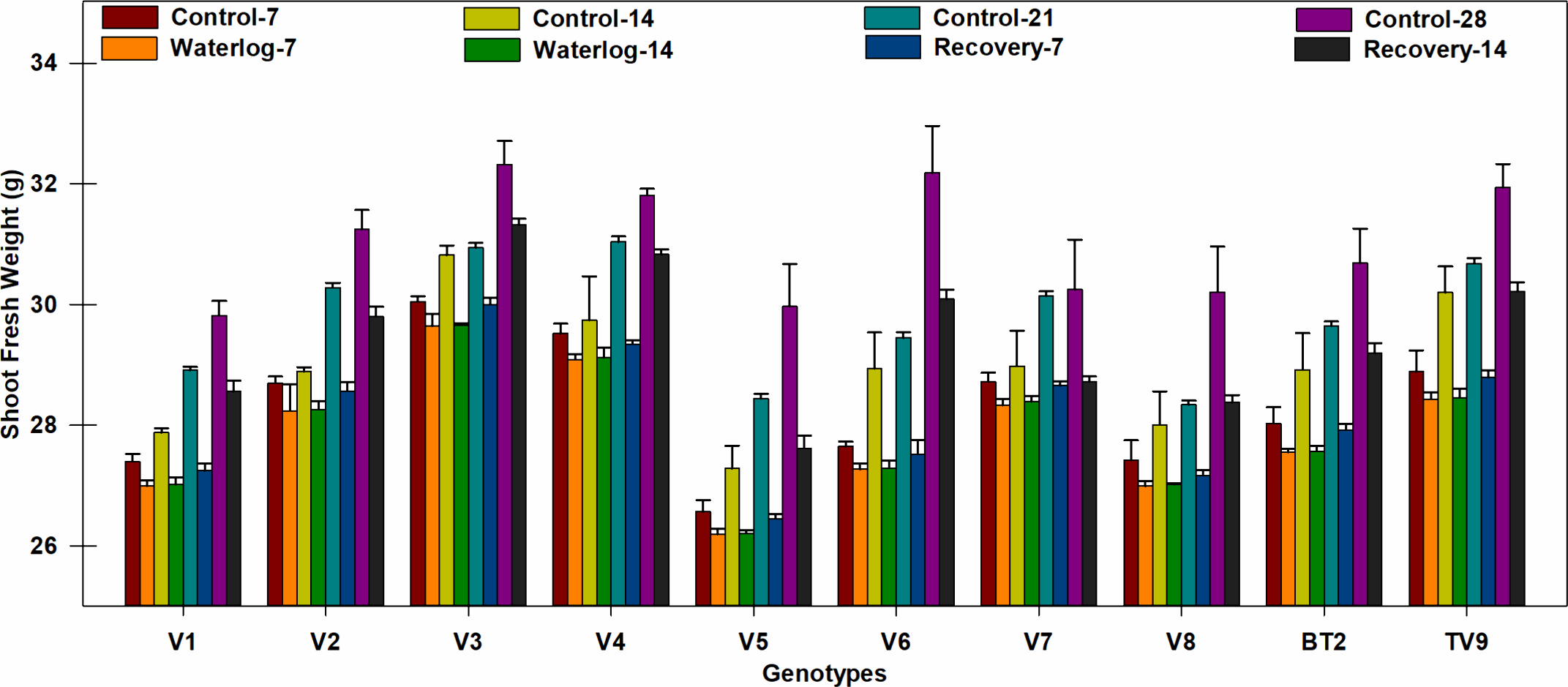

Supplement: S3 Fig — (TIF) [file pone.0354144.s006.tif]

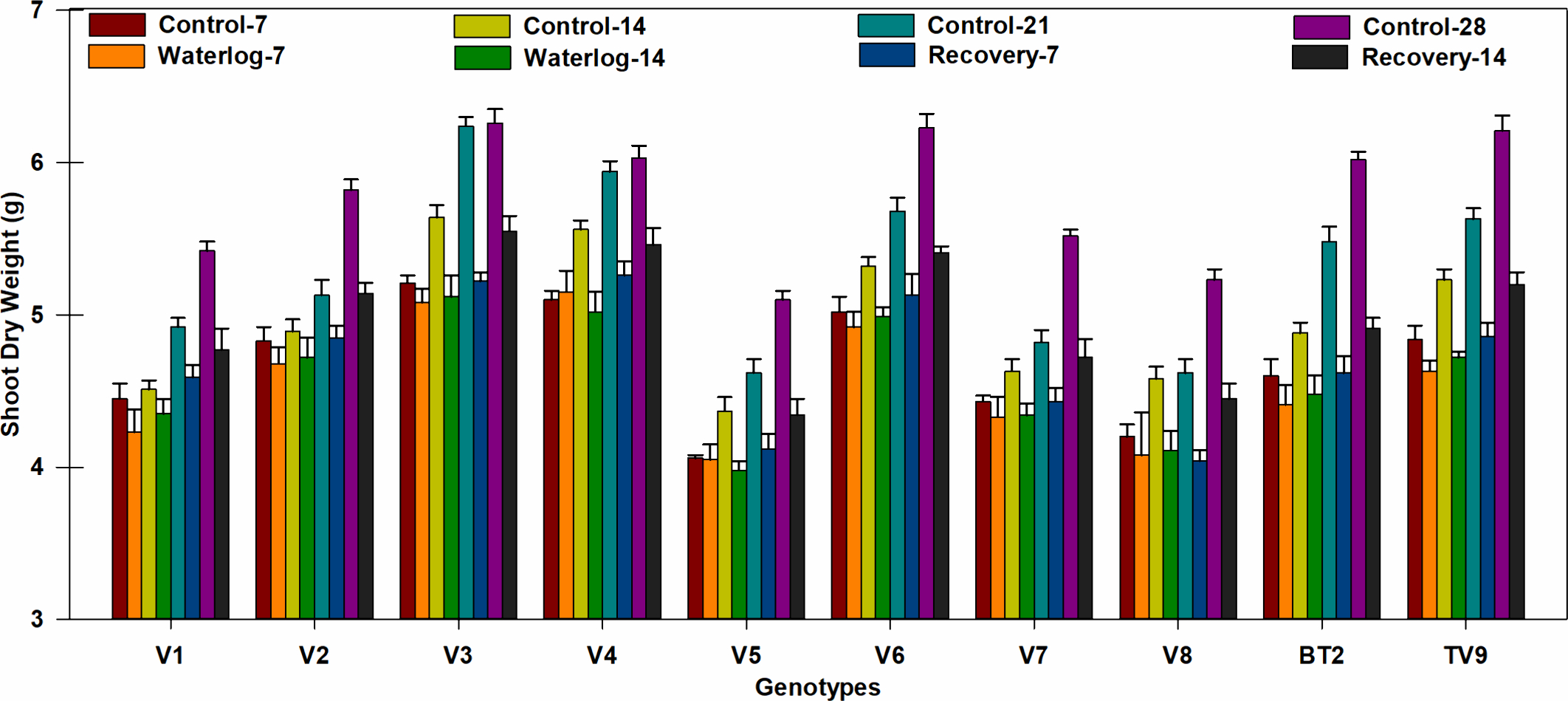

Supplement: S4 Fig — (TIF) [file pone.0354144.s007.tif]

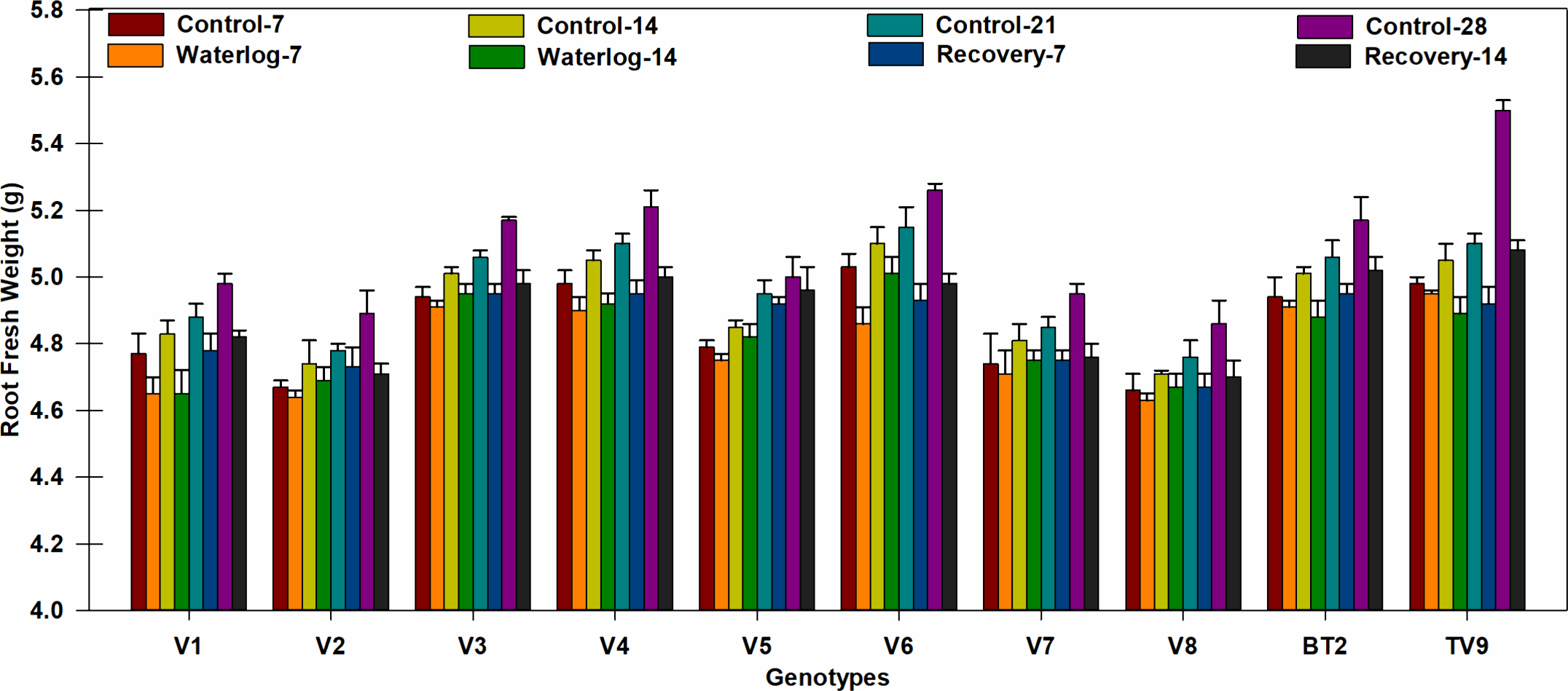

Supplement: S5 Fig — (TIF) [file pone.0354144.s008.tif]

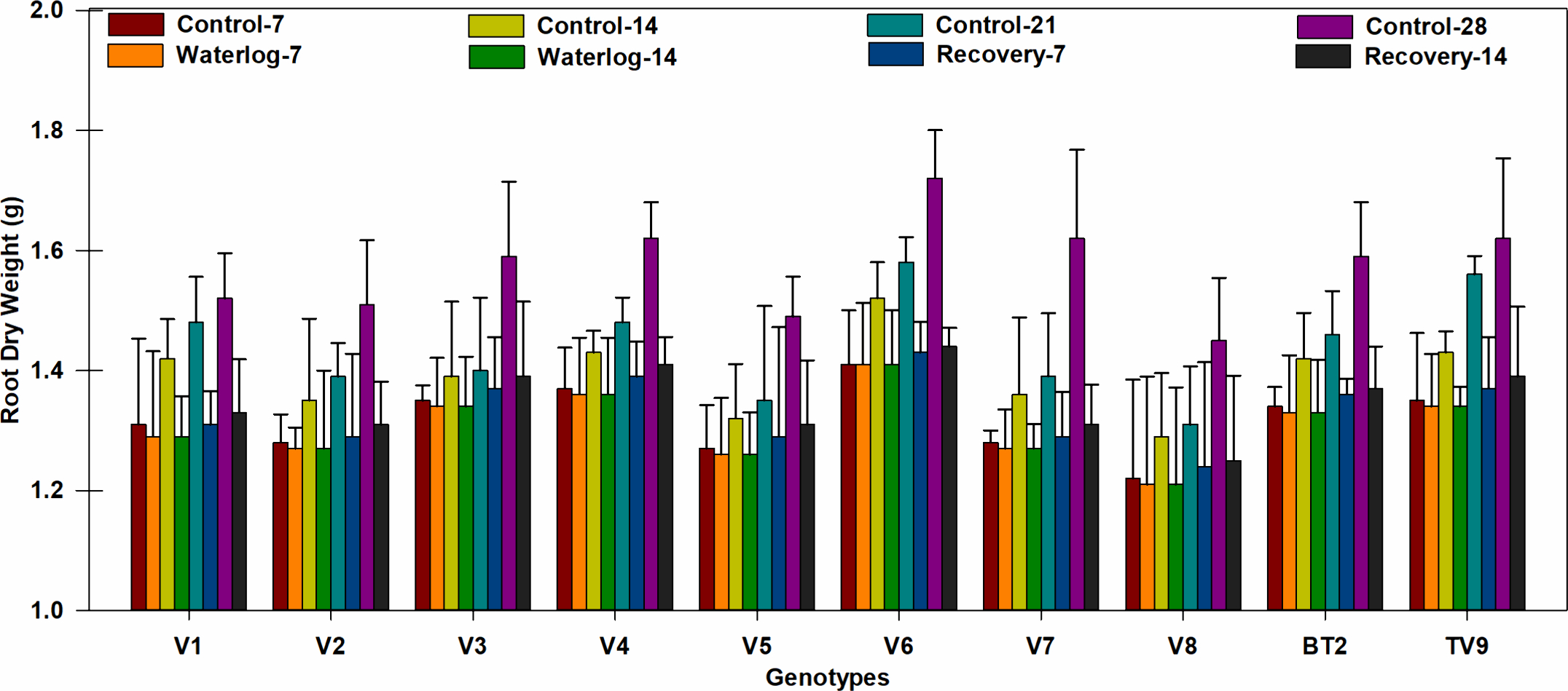

Supplement: S6 Fig — (TIF) [file pone.0354144.s009.tif]

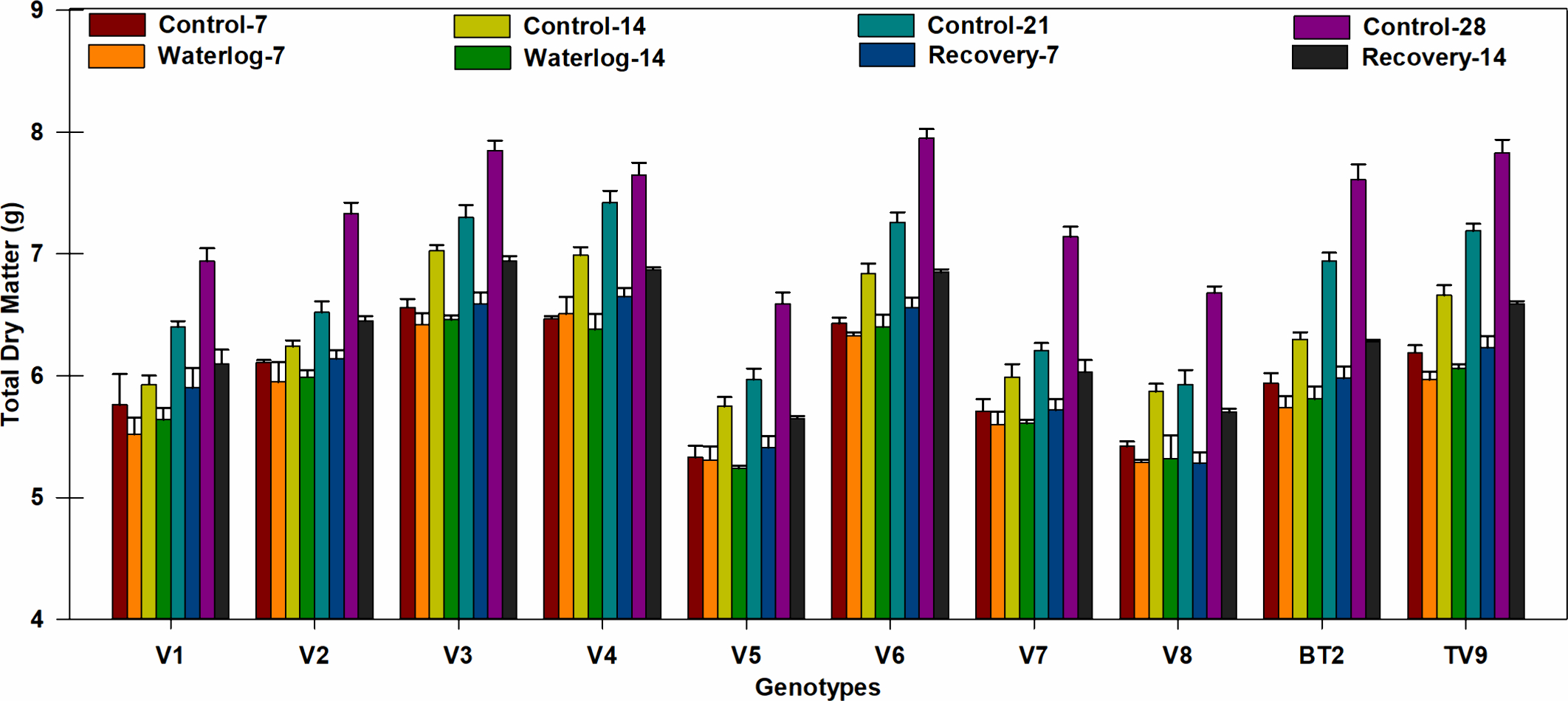

Supplement: S7 Fig — (TIF) [file pone.0354144.s010.tif]

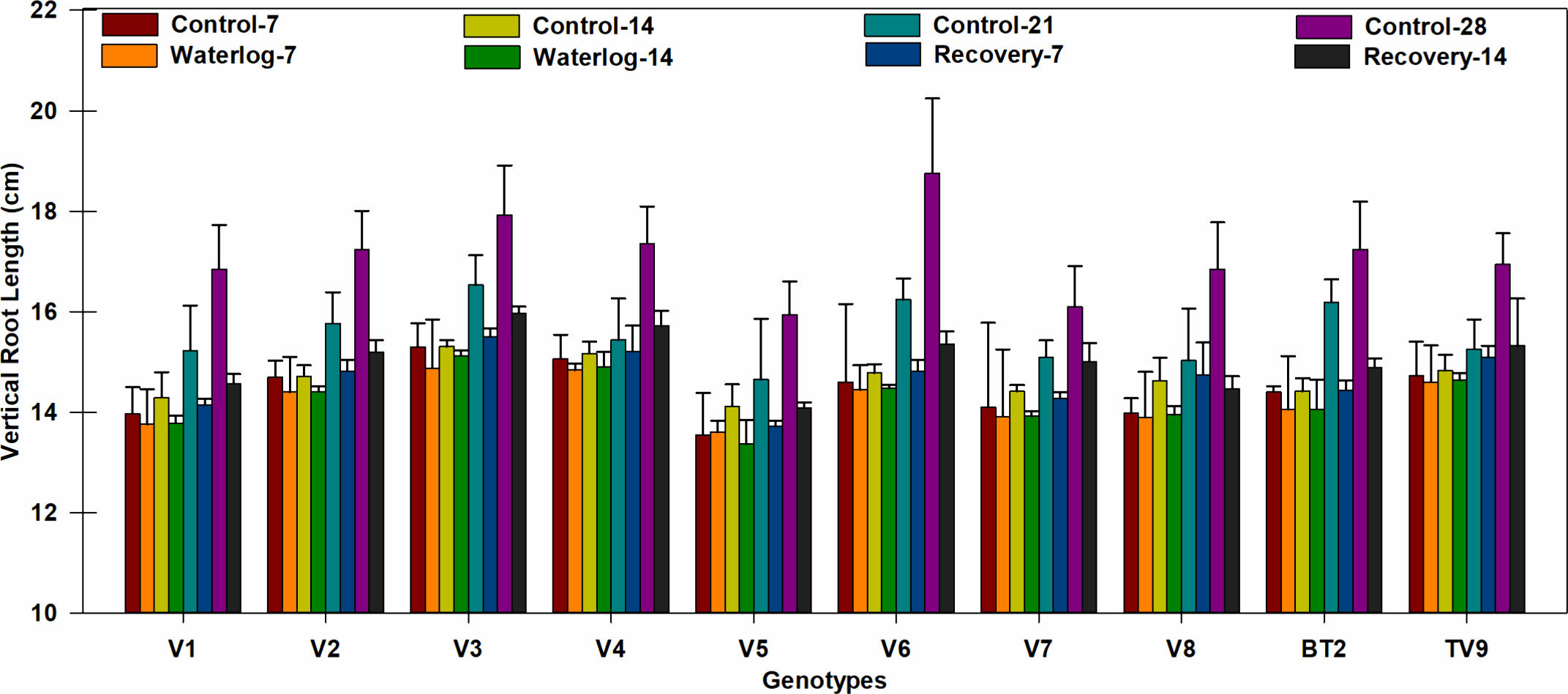

Supplement: S8 Fig — (TIF) [file pone.0354144.s011.tif]

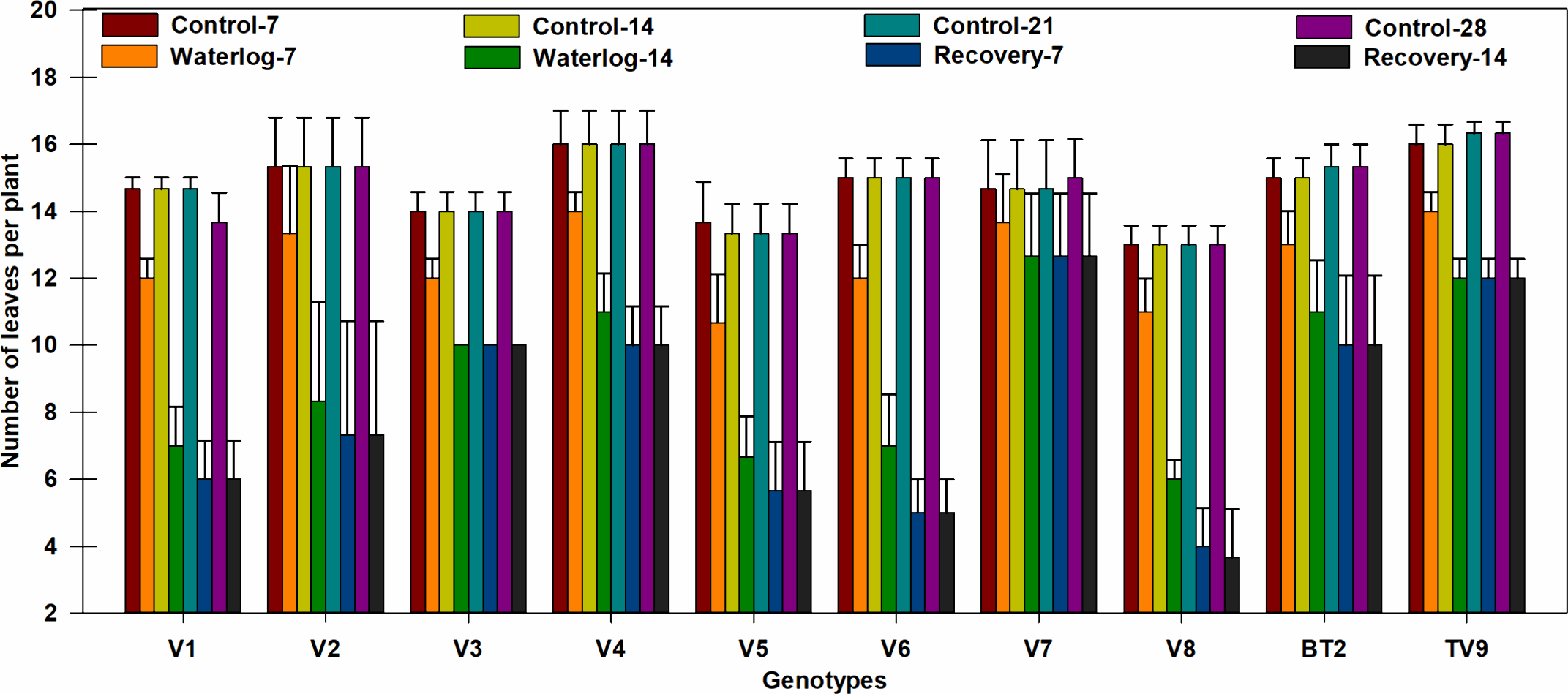

Supplement: S9 Fig — (TIF) [file pone.0354144.s012.tif]

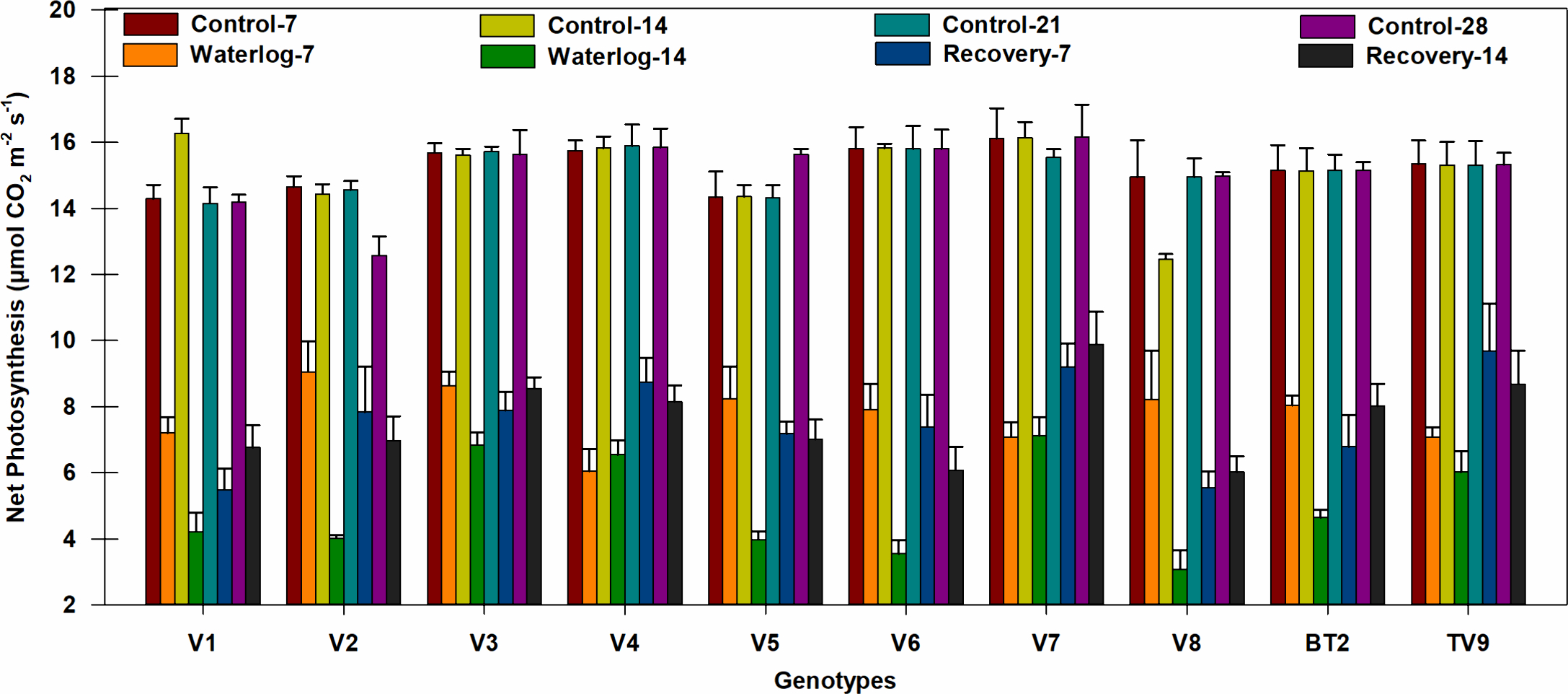

Supplement: S10 Fig — (TIF) [file pone.0354144.s013.tif]

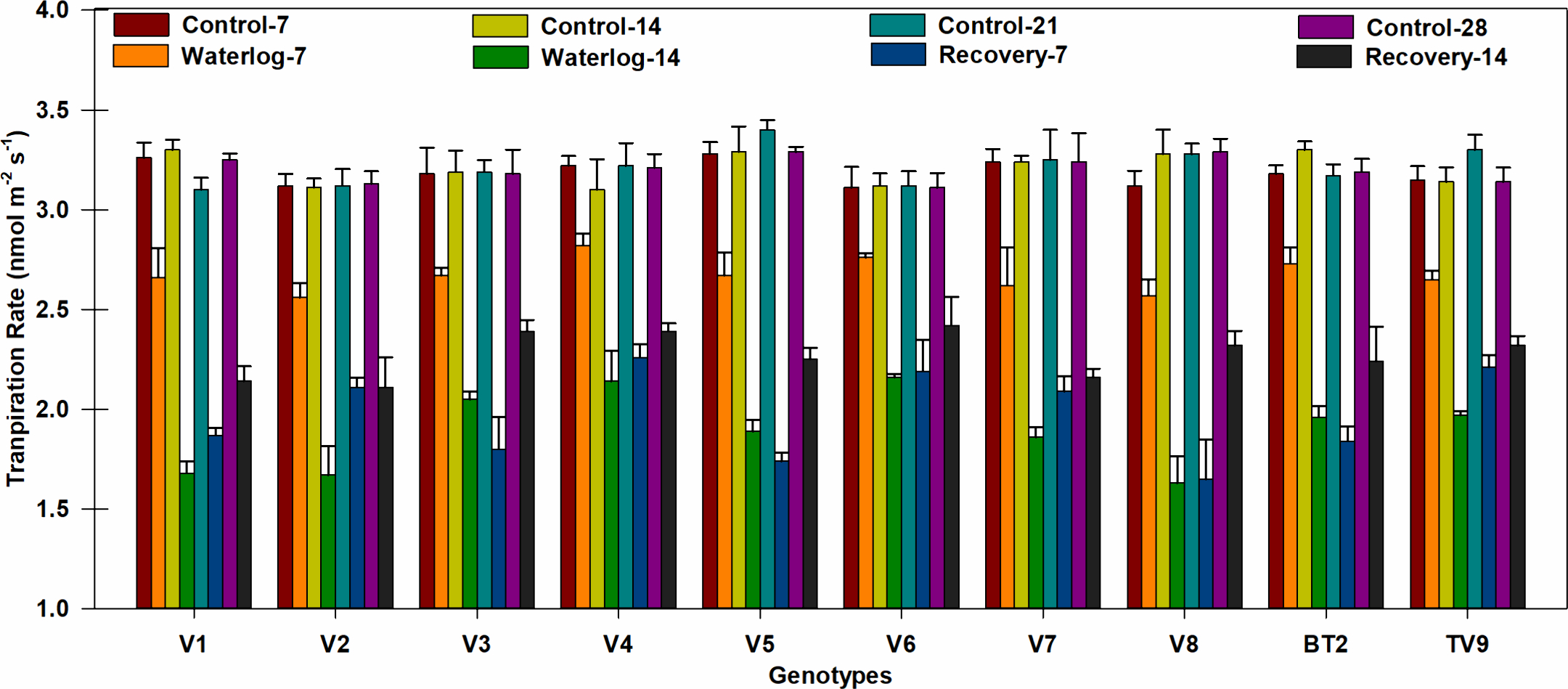

Supplement: S11 Fig — (TIF) [file pone.0354144.s014.tif]

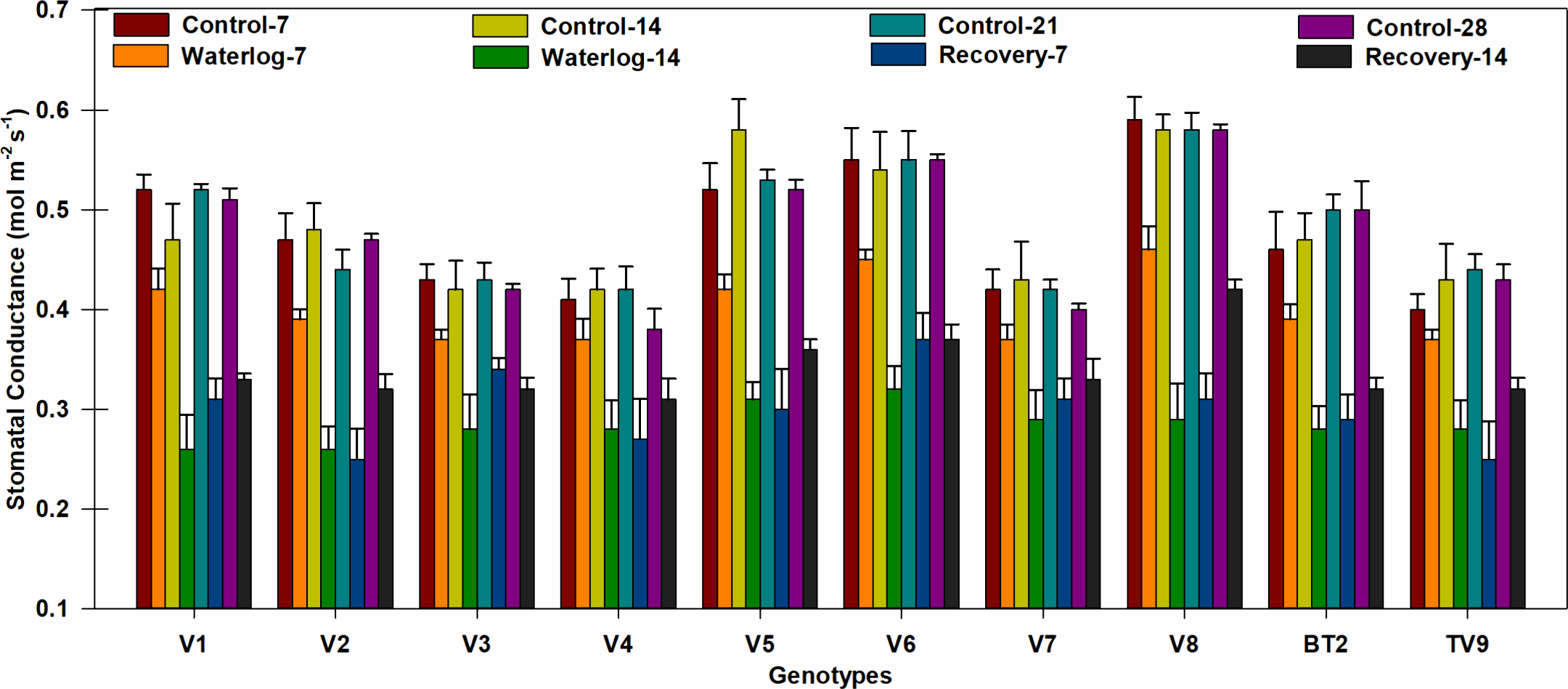

Supplement: S12 Fig — (TIF) [file pone.0354144.s015.tif]

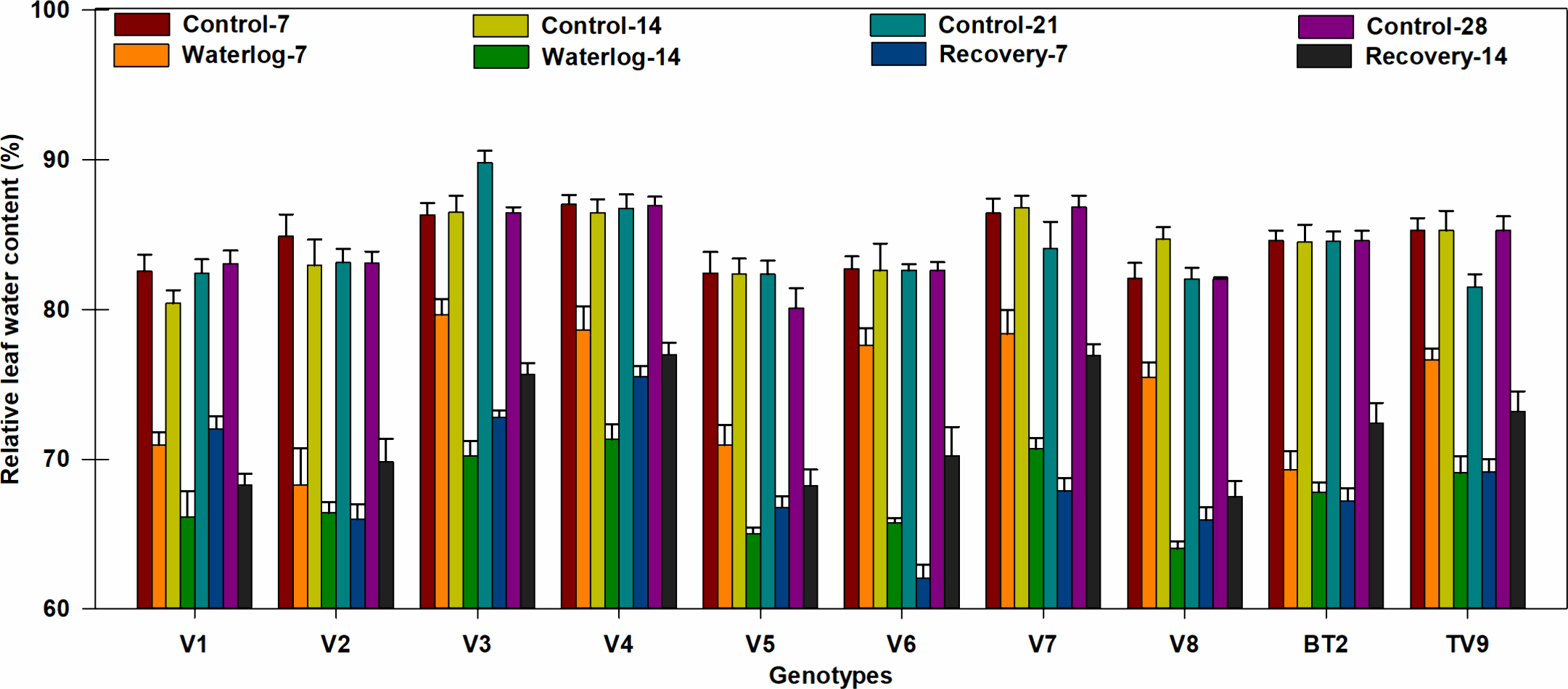

Supplement: S13 Fig — (TIF) [file pone.0354144.s016.tif]

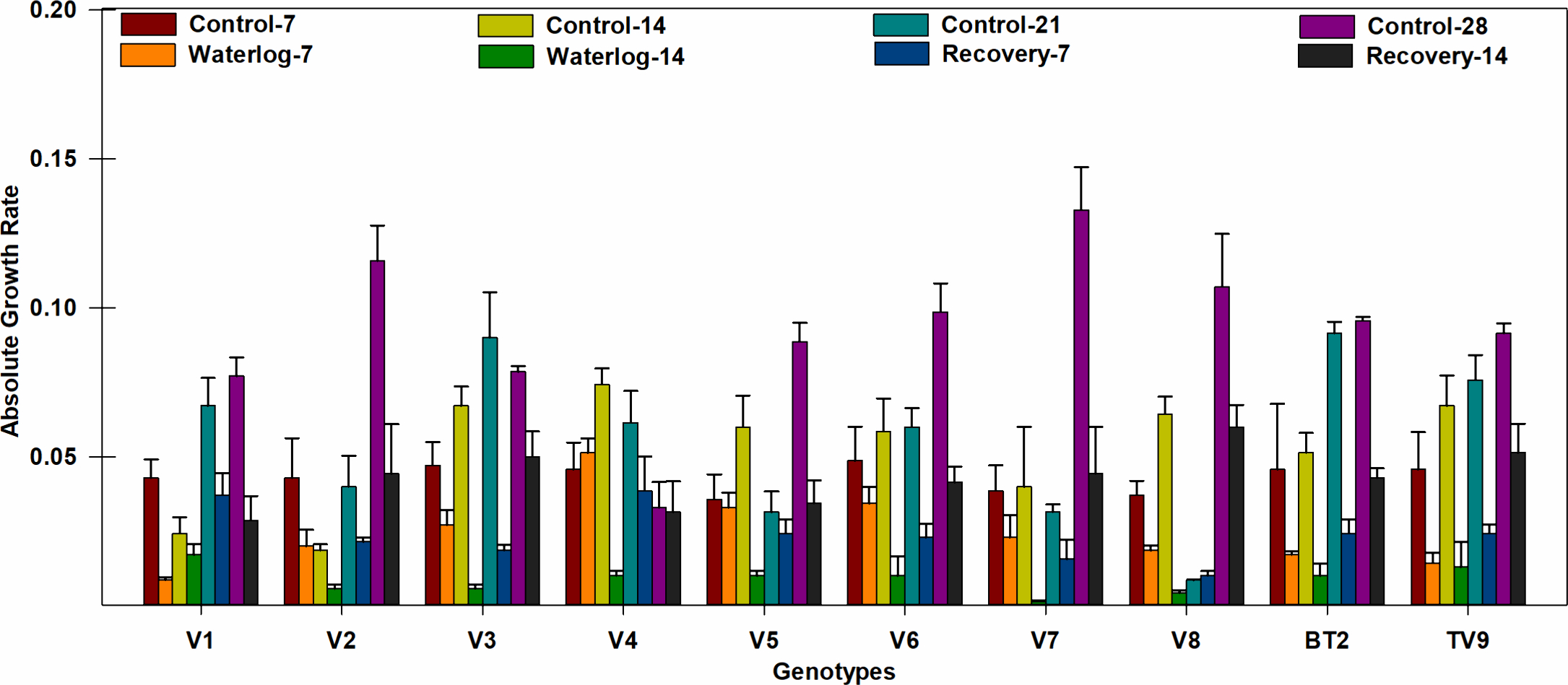

Supplement: S14 Fig — (TIF) [file pone.0354144.s017.tif]

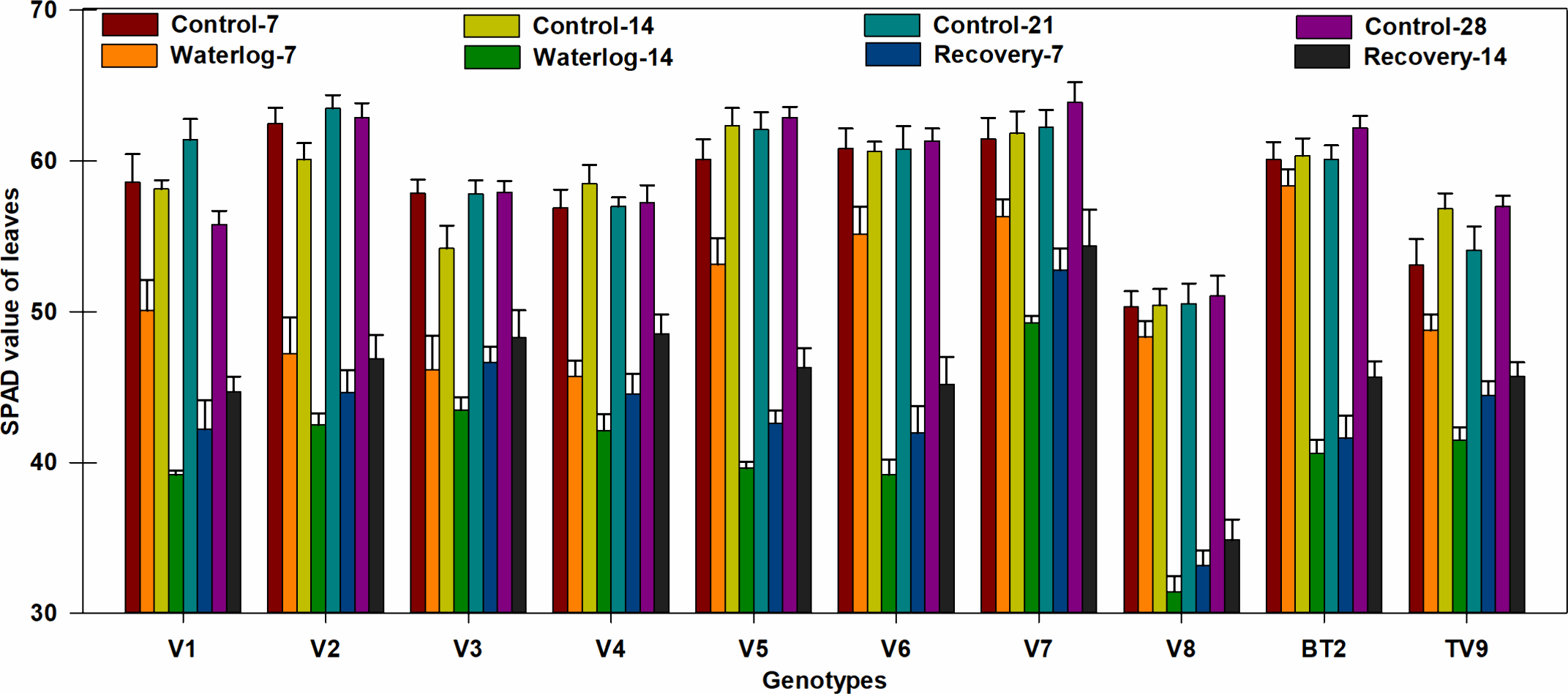

Supplement: S15 Fig — (TIF) [file pone.0354144.s018.tif]

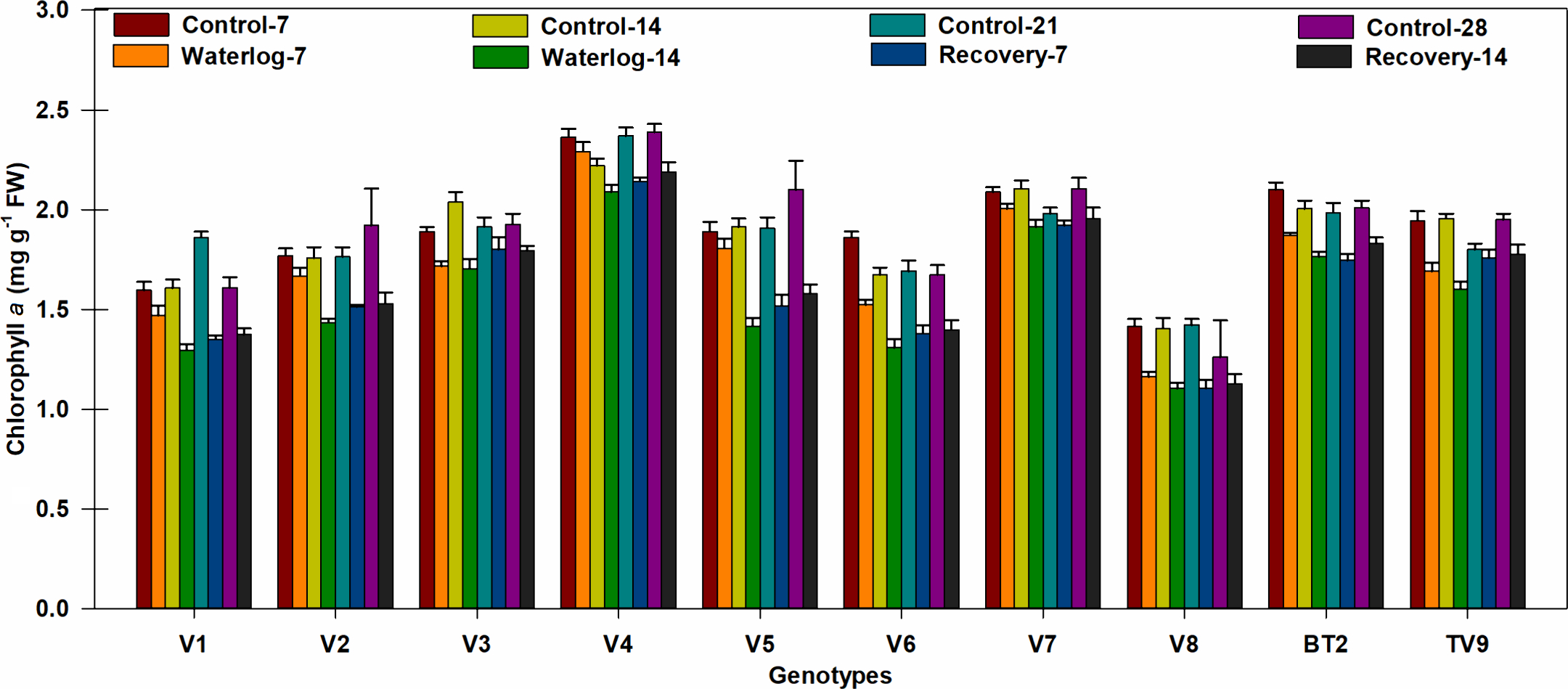

Supplement: S16 Fig — (TIF) [file pone.0354144.s019.tif]

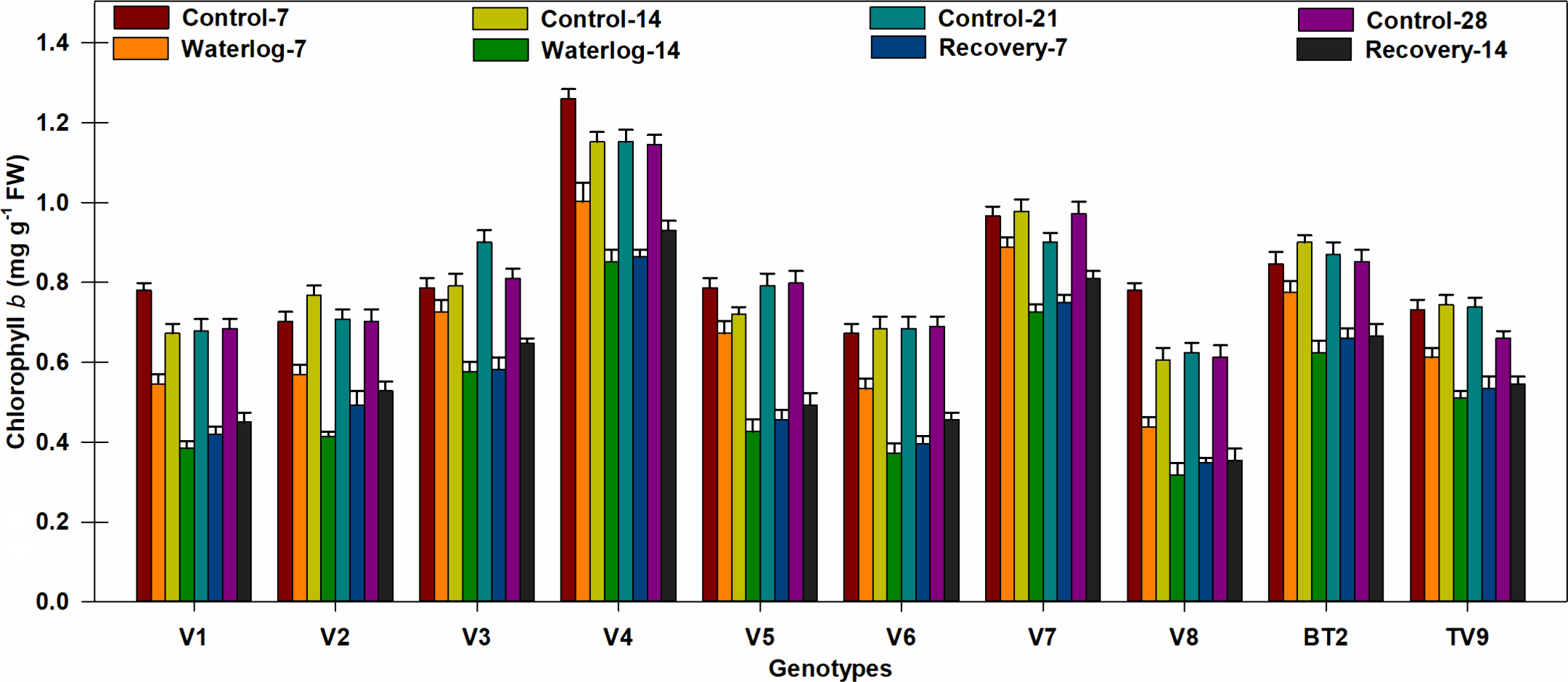

Supplement: S17 Fig — (TIF) [file pone.0354144.s020.tif]

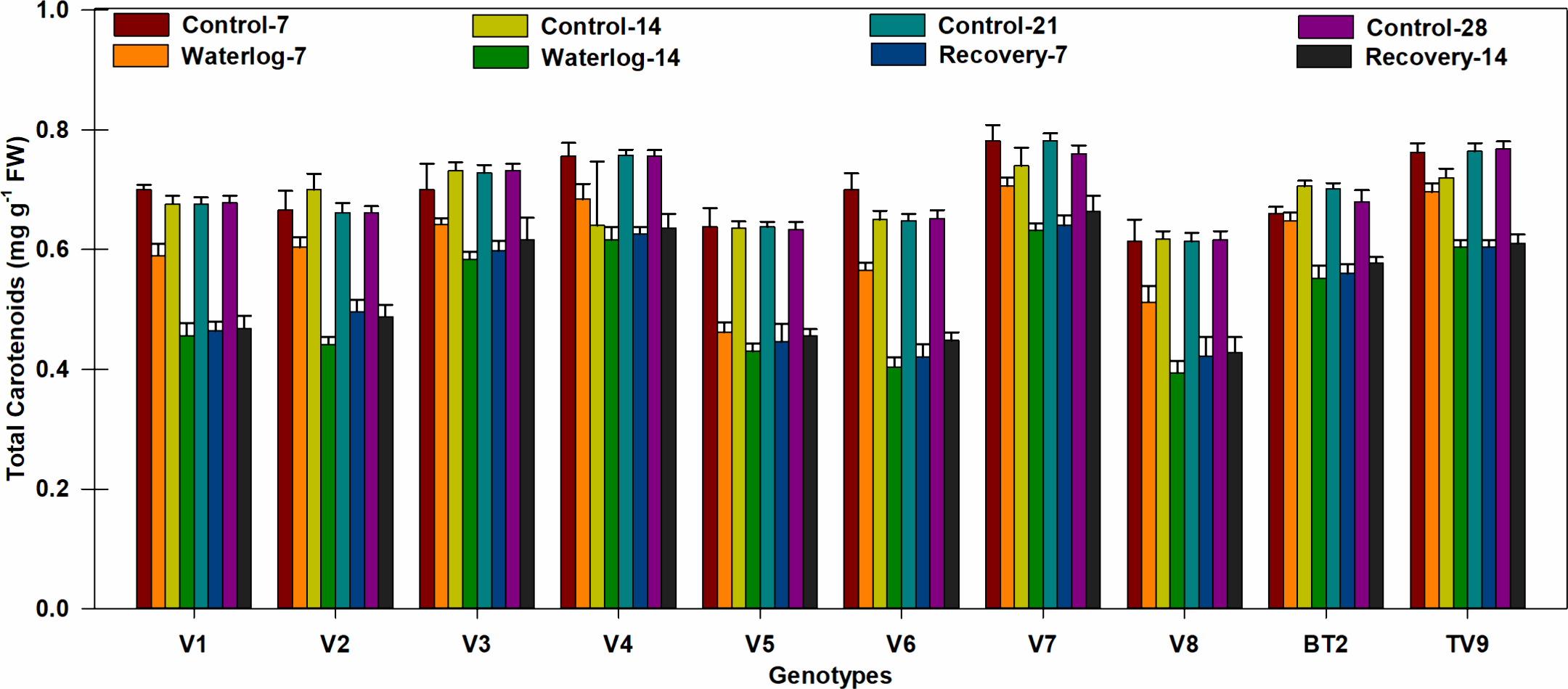

Supplement: S18 Fig — (TIF) [file pone.0354144.s021.tif]

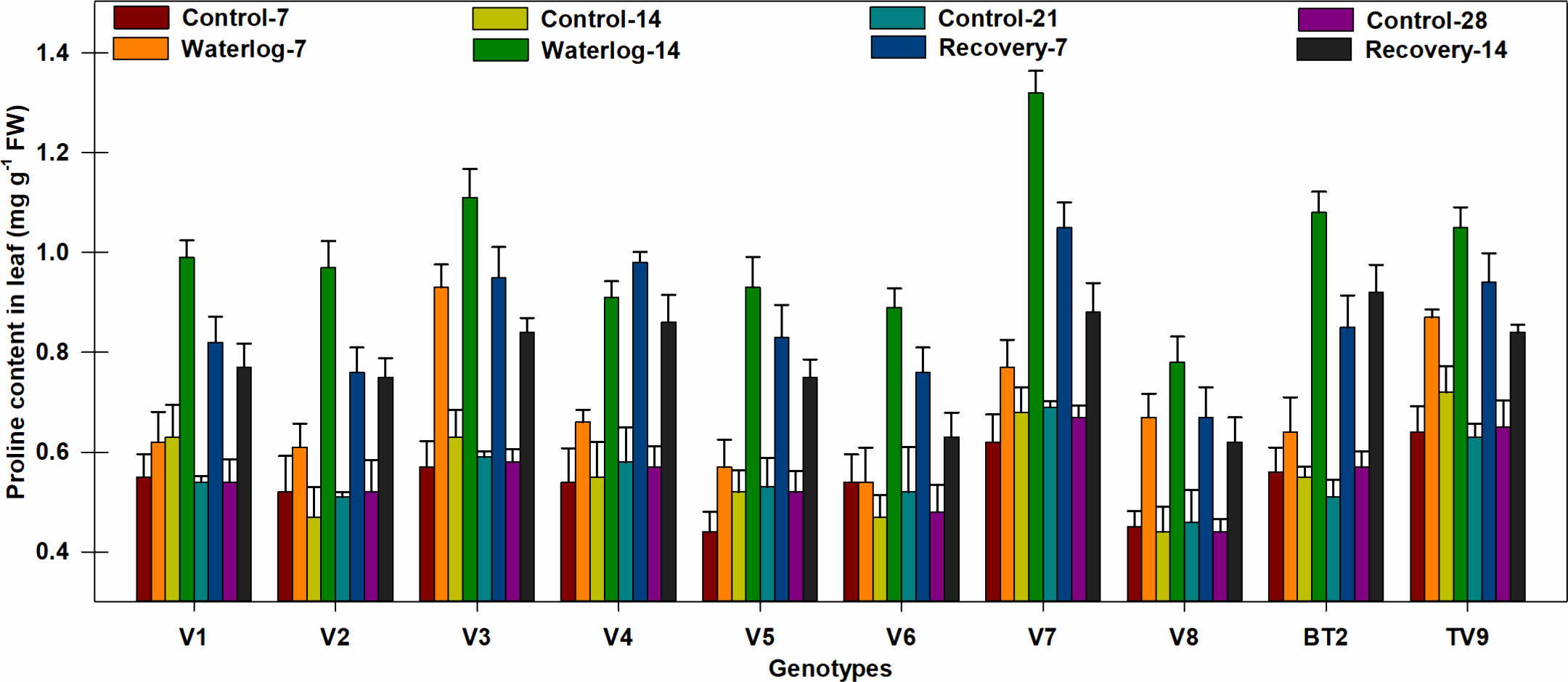

Supplement: S19 Fig — (TIF) [file pone.0354144.s022.tif]

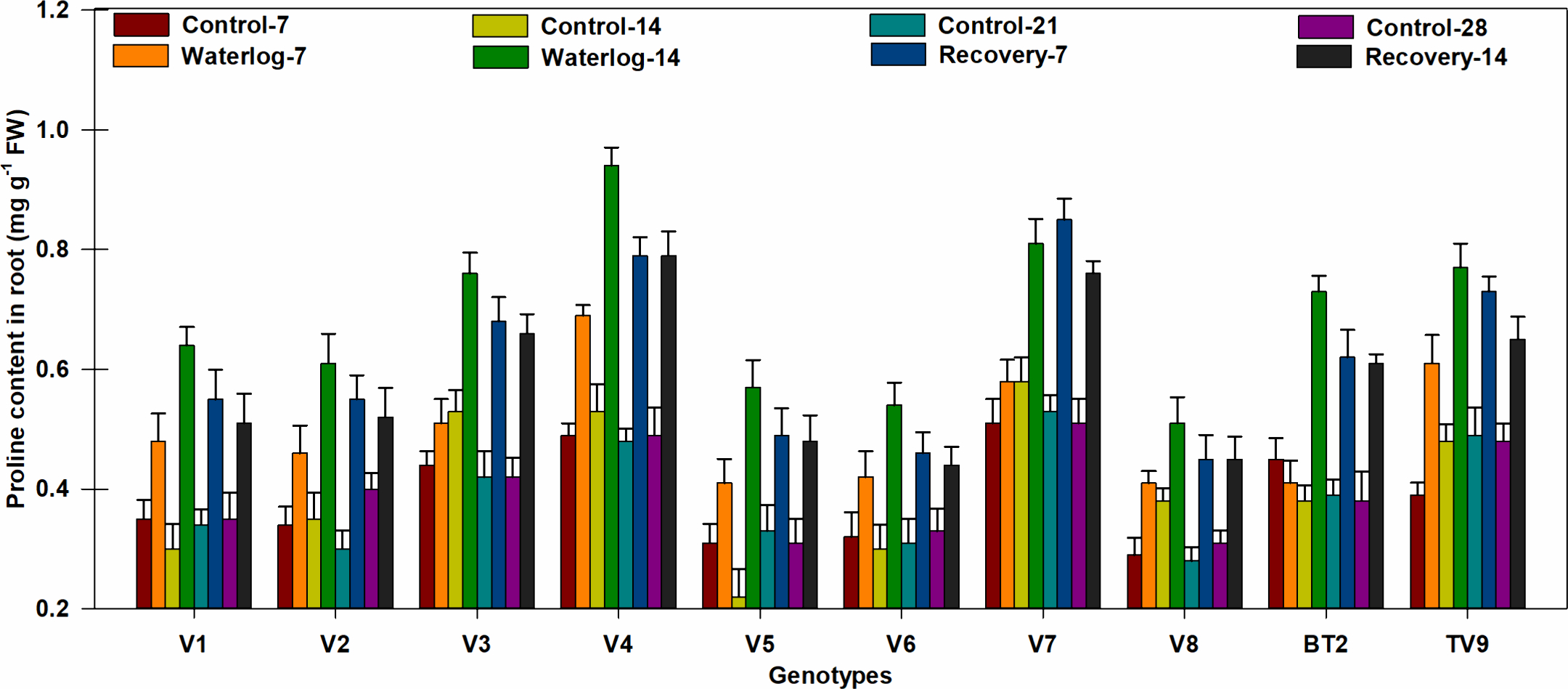

Supplement: S20 Fig — (TIF) [file pone.0354144.s023.tif]

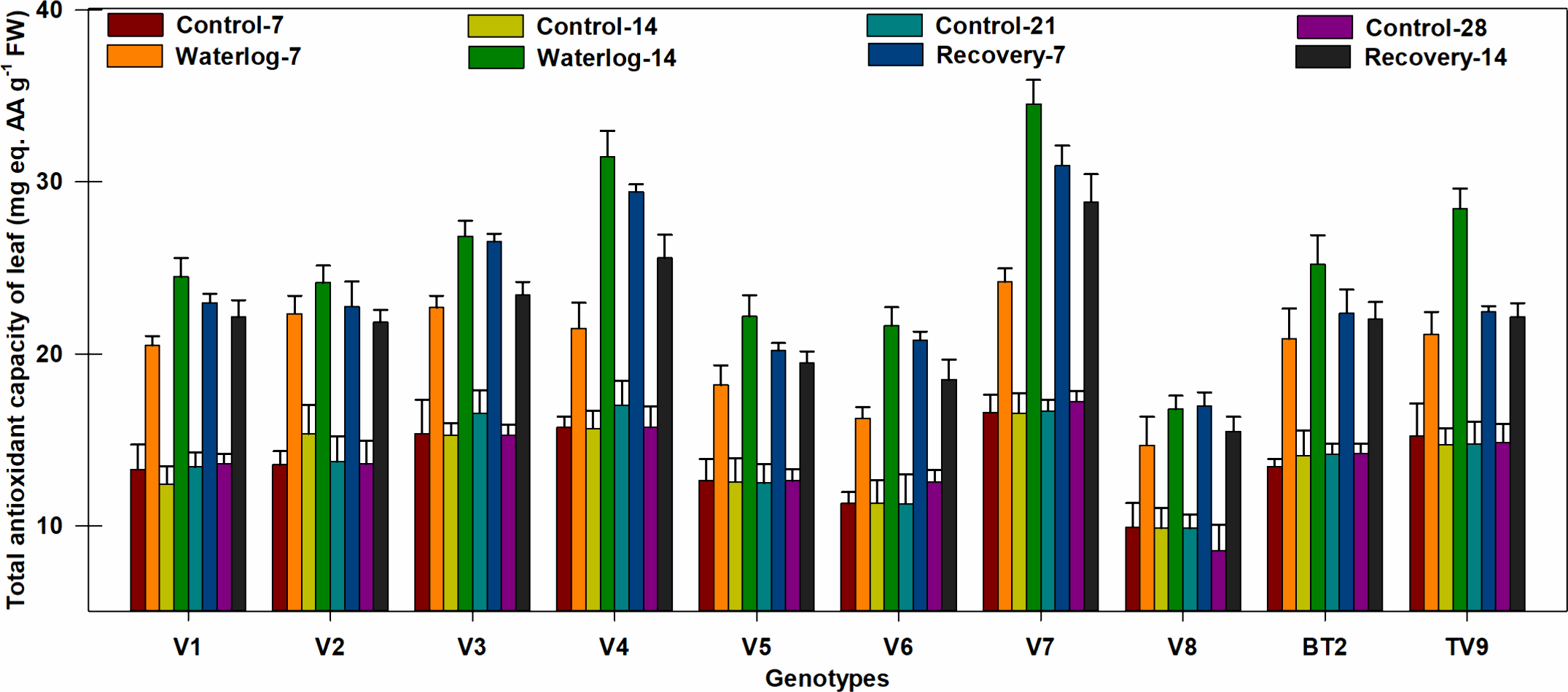

Supplement: S21 Fig — (TIF) [file pone.0354144.s024.tif]

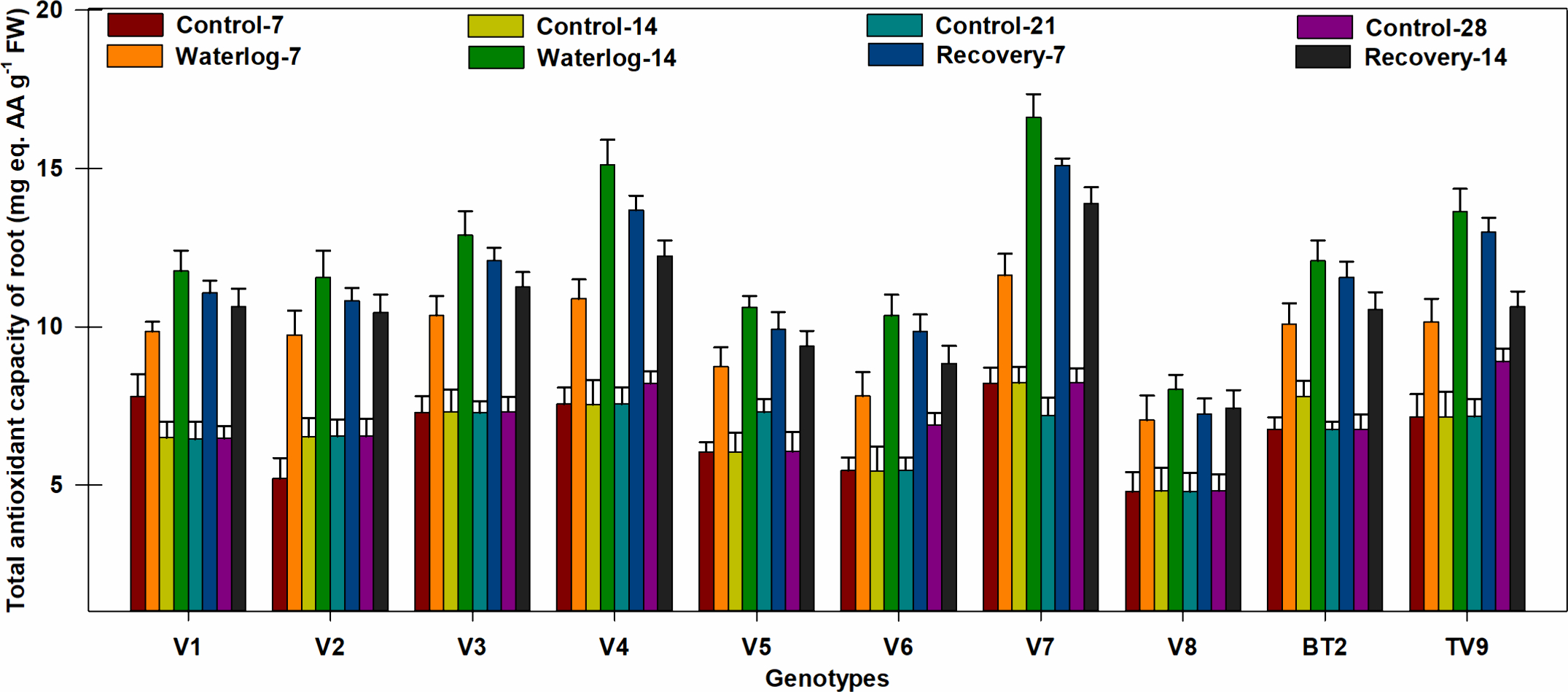

Supplement: S22 Fig — (TIF) [file pone.0354144.s025.tif]

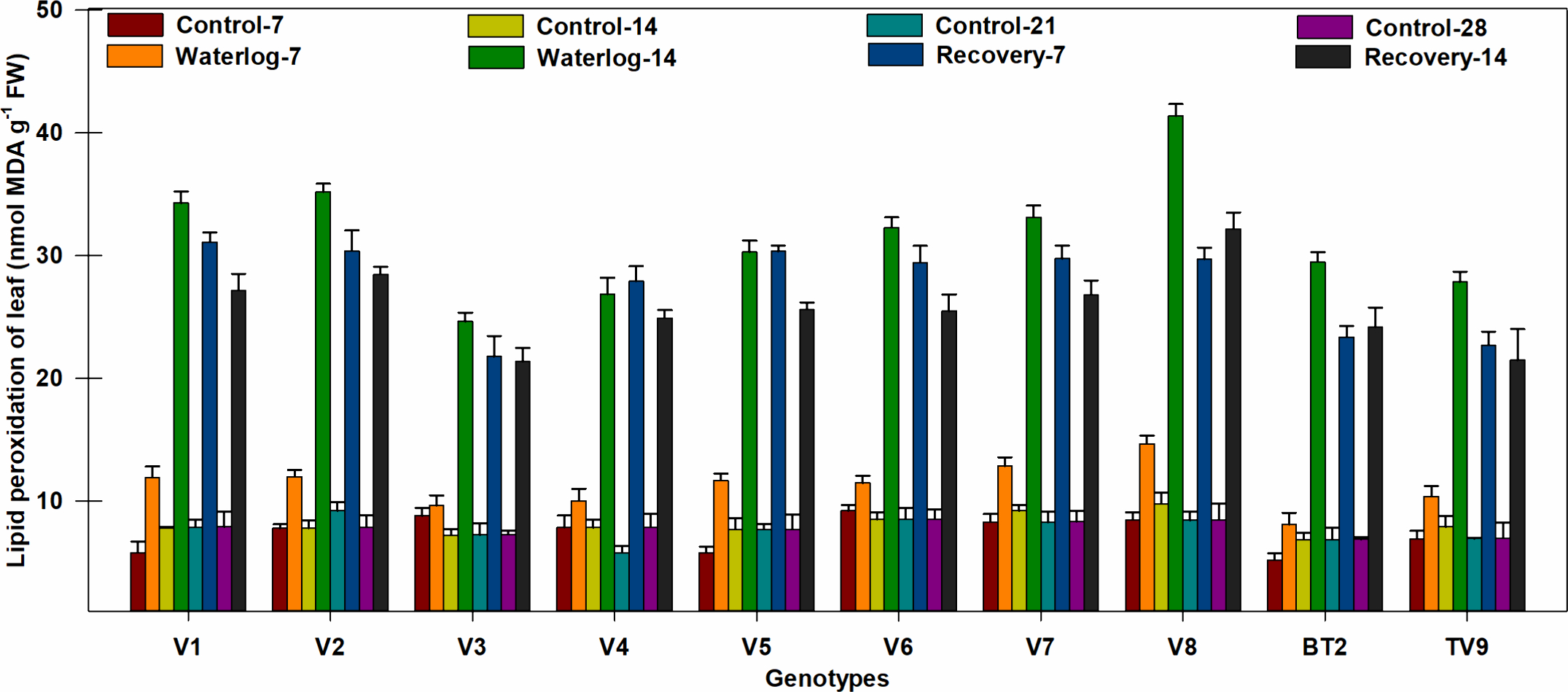

Supplement: S23 Fig — (TIF) [file pone.0354144.s026.tif]

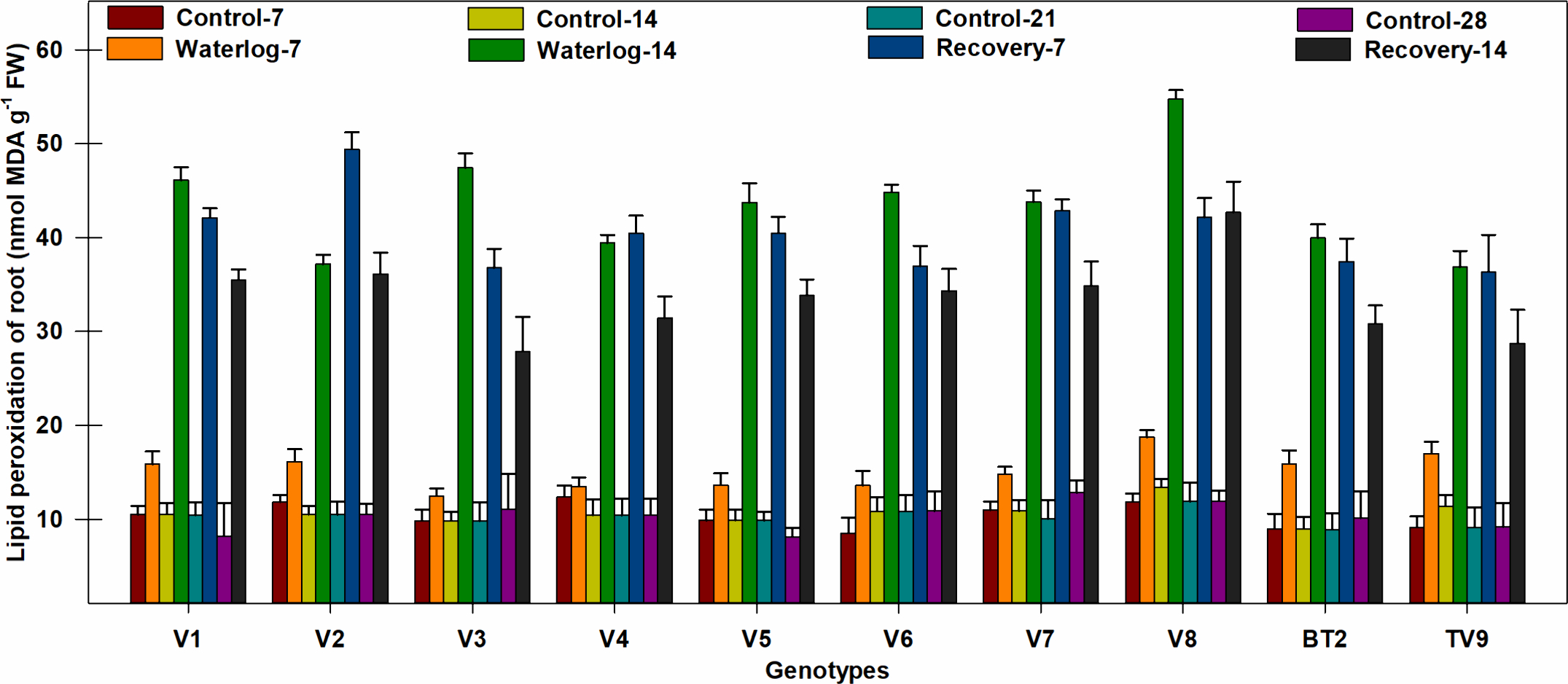

Supplement: S24 Fig — (TIF) [file pone.0354144.s027.tif]

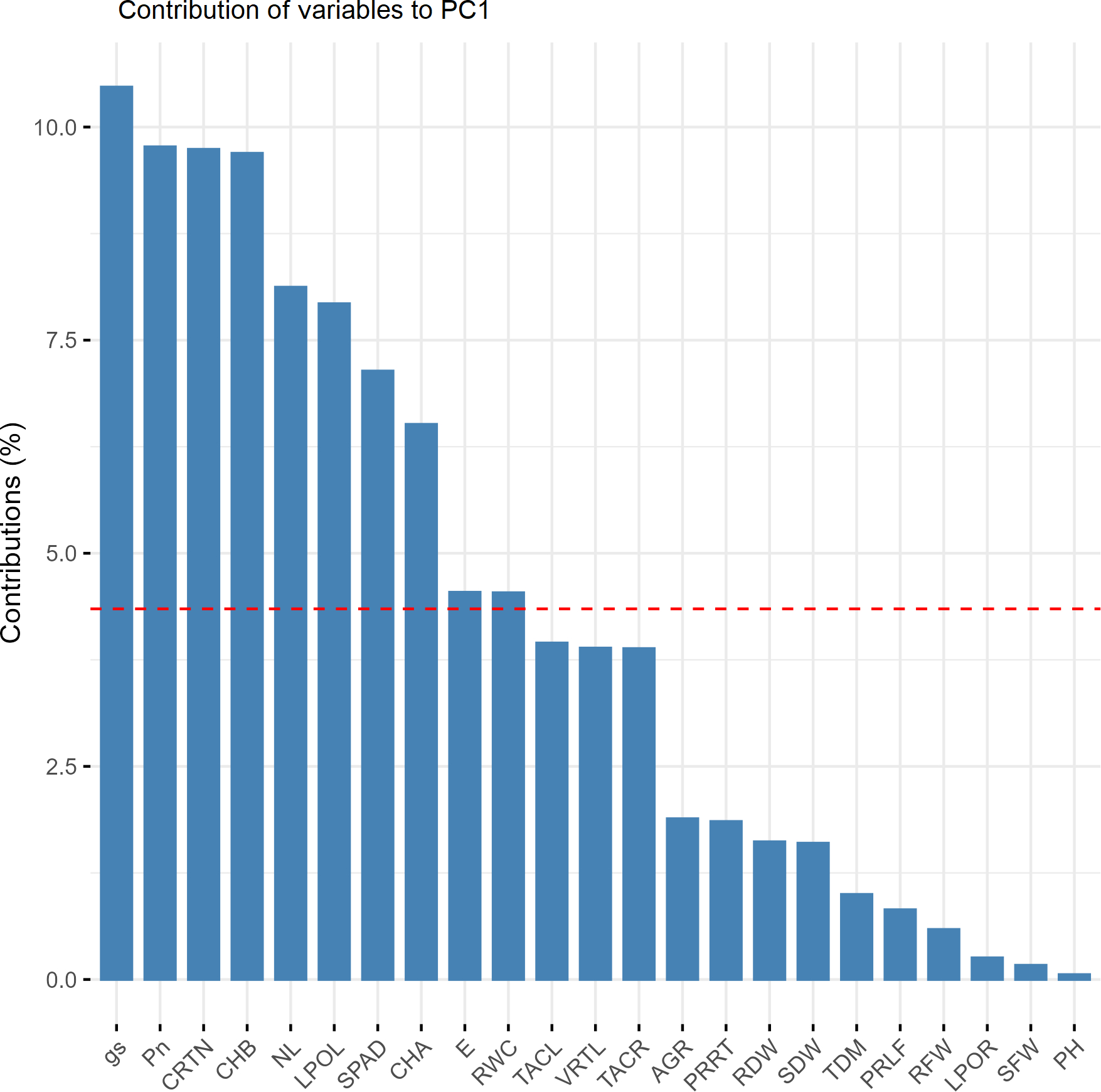

Supplement: S25 Fig — (TIF) [file pone.0354144.s028.tif]

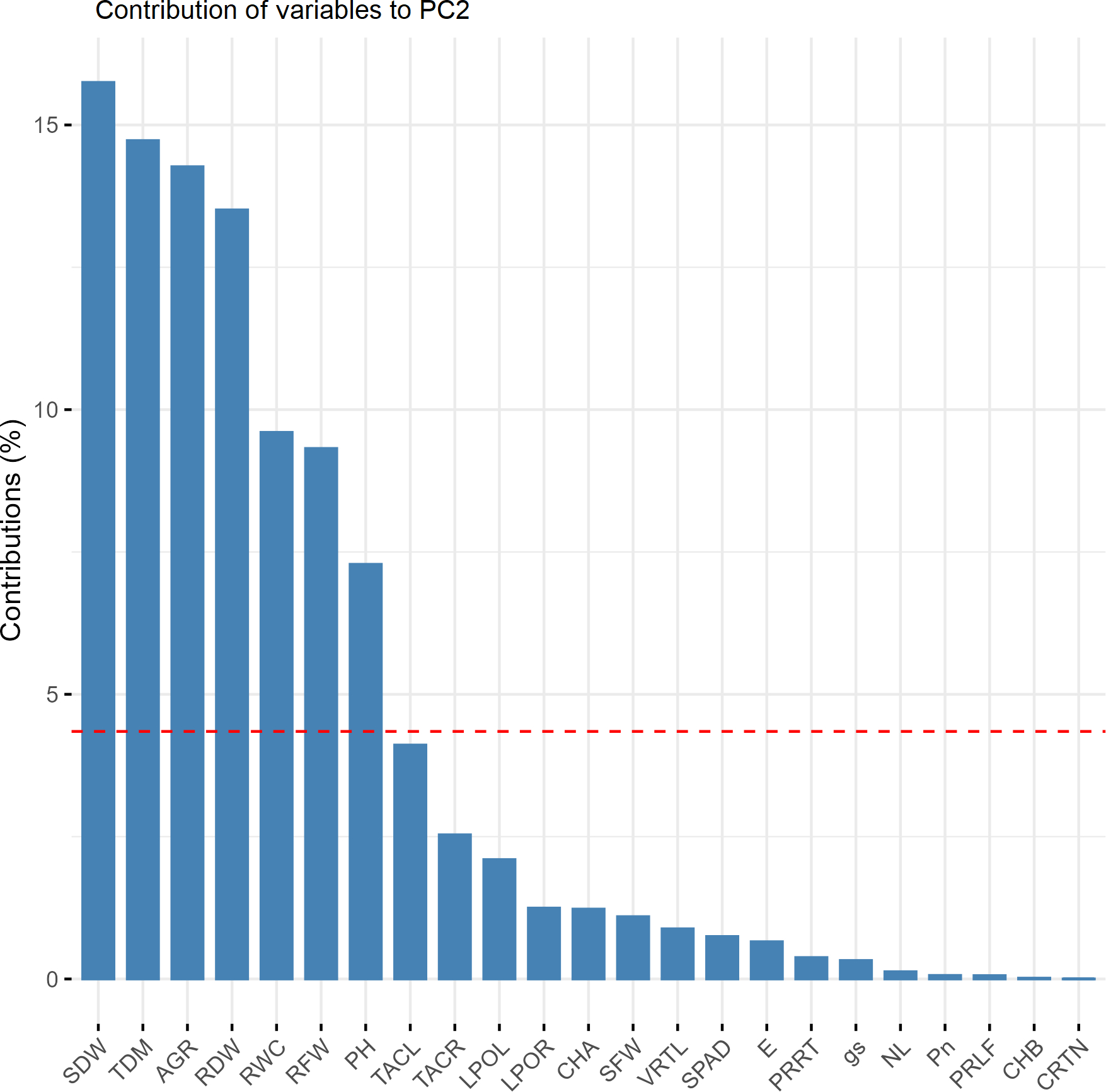

Supplement: S26 Fig — (TIF) [file pone.0354144.s029.tif]

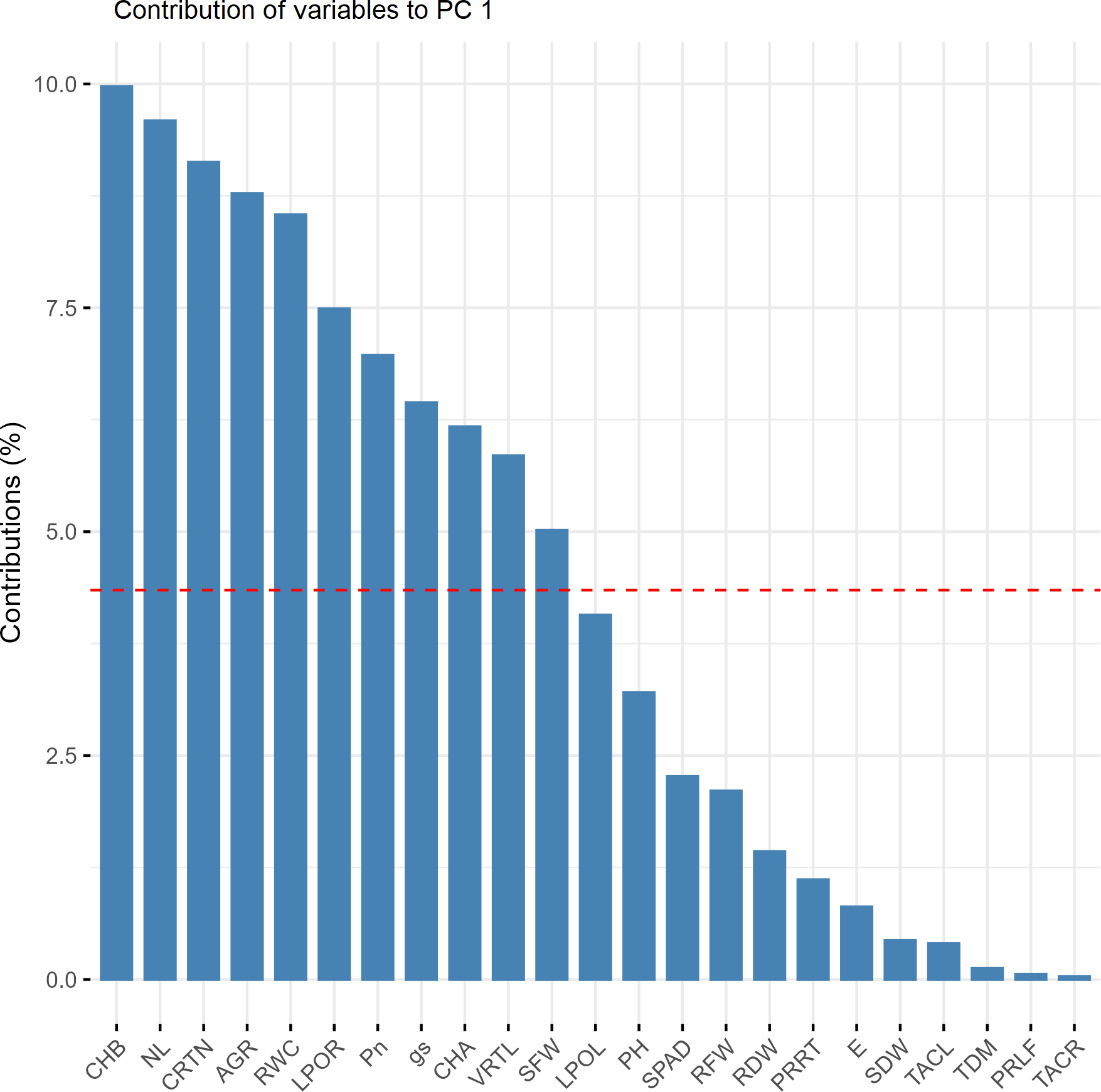

Supplement: S27 Fig — (TIF) [file pone.0354144.s030.tif]

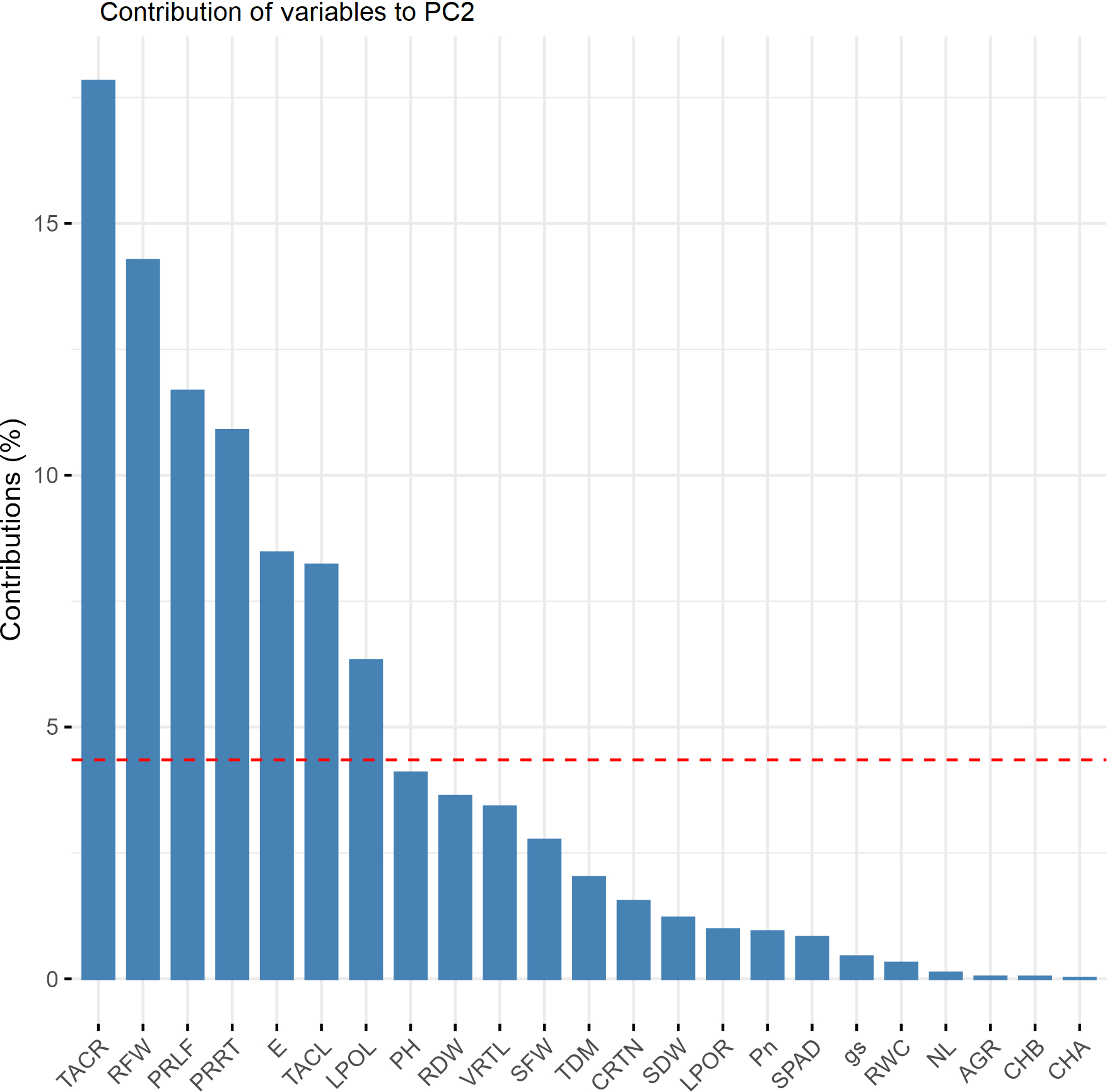

Supplement: S28 Fig — (TIF) [file pone.0354144.s031.tif]
