# Supplementary material for: Assessment of waterlogging tolerance in tea genotypes through morpho-physiological and biochemical profiling
Source: PLoS One. 2026 Jul 20;21(7):e0354144. doi: 10.1371/journal.pone.0354144 (PMC13384526; doi:10.1371/journal.pone.0354144)
Supplement: Attachment — Submitted filename: v1 .pdf [file pone.0354144.s032.pdf]

Abstract

- Clarify how tolerance was quantitatively determined.
- Avoid listing excessive abbreviations.
- State key numerical findings more concisely.

Introduction

- Improve flow and reduce repetition.
- Strengthen gap identification.
- Clearly state novelty compared to previous tea studies.

Materials and Methods

- Clarify experimental unit and replication.
- Provide more detail on environmental conditions (temperature, humidity).
- Specify light intensity during measurements.
- Indicate calibration details of instruments.
- Clarify whether measurements were taken at the same time of day.

Results

- Reduce repetition between text and figures.
- Present key numerical values in tables rather than descriptive narration.
- Ensure figure numbering consistency.
- Improve clarity of clustering interpretation.

Discussion

- Avoid restating results.

- Strengthen mechanistic explanations.
- Compare findings more deeply with previous tea studies.
- Distinguish clearly between stress response and adaptive tolerance.

Figures and Tables

- Improve resolution and readability.
- Shorten overly long figure captions.
- Ensure consistency in abbreviation formatting.
- Verify consistency between text and supplementary figures.

4. Minor Comments

- Check consistency of abbreviations (LPOL vs LPOL/LPOR formatting).
- Correct typographical errors.
- Ensure all supplementary figures are referenced properly.
- Standardize units (e.g., µmol CO_2 m^{-2} s^{-1} formatting).
- Correct repetition in correlation description (e.g., duplicated trait names).

5. Final Recommendation

The study is scientifically relevant and potentially valuable for tea breeding programs. However, major revisions are required to improve statistical rigor, interpretation depth, and clarity of presentation.

Recommendation: Major Revision
