# Supplementary material for: Assessment of waterlogging tolerance in tea genotypes through morpho-physiological and biochemical profiling
Source: PLoS One. 2026 Jul 20;21(7):e0354144. doi: 10.1371/journal.pone.0354144 (PMC13384526; doi:10.1371/journal.pone.0354144)
Supplement: Attachment — Submitted filename: PONE-D-26-00282.pdf [file pone.0354144.s033.pdf]

PLOS One

Assessment of waterlogging tolerance in tea genotypes through morpho-physiological and biochemical profiling

--Manuscript Draft--

**Manuscript Number:**	PONE-D-26-00282
**Article Type:**	Research Article
**Full Title:**	Assessment of waterlogging tolerance in tea genotypes through morpho-physiological and biochemical profiling
**Short Title:**	Assessment of waterlogging tolerance in tea
**Corresponding Author:**	Md. Riyadh Arefin<br>Bangladesh Agricultural University<br>Mymensingh, BANGLADESH
**Keywords:**	Tea; Plant stress; Stress physiology; Waterlogging problem
**Abstract:**	Tea is one of the major economic crops in Bangladesh; however, its productivity is severely constrained by waterlogging stress arising from erratic climatic changes. The lack of tolerant varieties is a critical barrier to sustainable tea cultivation. Therefore, the present study aimed to investigate the integrated morpho-physiological and biochemical responses of tea genotypes under waterlogging stress to identify tolerant genotype(s). The experiment was conducted at the Bangladesh Tea Research Institute from August to December 2024, following a completely randomized design with three replications. Eight pre-screened tea genotypes—P/LAL/08/23, P/LAL/08/62, P/LAL/09/116, P/AFN/11/35, P/AFN/11/46, P/OTI/31, P/AFN/13/90, P/AFN/11/31, with two control clones, BT2 (popular variety) and TV9 (waterlogging tolerant variety), were evaluated by 23 morpho-physiological and biochemical parameters under two treatments: i) control treatment and ii) stress treatment. The stress treatment was established by exposing the tea genotypes to a waterlogged phase of 14 days, with a 14-day recovery phase. All the morphological traits, like root fresh weight (RFW), leaf number (NL), etc., and physiological characters, such as net photosynthesis (Pn), transpiration rate (E), stomatal conductance (gs), relative leaf water content (RWC), absolute growth rate (AGR), etc., were decreased remarkably under stress condition compared to control. Among the biochemical parameters, leaf pigments, like chlorophyll a (CHA), chlorophyll b (CHB), and total carotenoids (CRTN), were also observed to be reduced, while proline content (leaf: PRLF and root: PRRT), total antioxidant activity (leaf: TACL and root: TACR), and lipid peroxidation (leaf: LOPL and root: LOPR) substantially increased under stress treatment. Out of the 23 evaluated traits, only 11 traits—RFW, NL, Pn, E, gs, RWC, AGR, CHA, CHB, CRTN, and LPOL were detected as most influential at both phases of stress treatment. Ultimately, the P/AFN/13/90 genotype was identified as the most potentially tolerant genotype, exhibiting better responses than others under both waterlogging and recovery phases.
**Order of Authors:**	Md. Riyadh Arefin
	Shitosri Mondal
	Md Fazle Rabbi
	Hafsa Tasnim
	Md. Alamgir Hossain, PhD
	Md. Sabibul Haque, PhD
	A.K.M. Golam Sarwar, PhD
**Additional Information:**	
**Question**	**Response**
**Financial Disclosure**<br><br>Enter a financial disclosure statement that	The author(s) received no specific funding for this work.

Powered by Editorial Manager® and ProduXion Manager® from Aries Systems Corporation

describes the sources of funding for the work included in this submission. Review the submission guidelines for detailed requirements. View published research articles from *PLOS ONE* for specific examples.<br><br>This statement is required for submission and **will appear in the published article** if the submission is accepted. Please make sure it is accurate.<br><br>**Funded studies**<br>Enter a statement with the following details:<br>• Initials of the authors who received each award<br>• Grant numbers awarded to each author<br>• The full name of each funder<br>• URL of each funder website<br>• Did the sponsors or funders play any role in the study design, data collection and analysis, decision to publish, or preparation of the manuscript?<br><br>Did you receive funding for this work?	
**Competing Interests**<br><br>Use the instructions below to enter a competing interest statement for this submission. On behalf of all authors, disclose any competing interests that could be perceived to bias this work—acknowledging all financial support and any other relevant financial or non-financial competing interests.<br><br>This statement is required for submission and **will appear in the published article** if the submission is accepted. Please make sure it is accurate and that any funding sources listed in your Funding Information later in the submission form are also declared in your Financial Disclosure statement.<br><br>View published research articles from *PLOS ONE* for specific examples.	The authors have declared that no competing interests exist.

**NO authors have competing interests**<br><br>Enter: *The authors have declared that no competing interests exist.*<br><br>**Authors with competing interests**<br><br>Enter competing interest details beginning with this statement:<br><br>*I have read the journal's policy and the authors of this manuscript have the following competing interests: [insert competing interests here]*<br><br>* typeset	
**Ethics Statement**<br><br>Enter an ethics statement for this submission. This statement is required if the study involved:<br><br>• Human participants<br>• Human specimens or tissue<br>• Vertebrate animals or cephalopods<br>• Vertebrate embryos or tissues<br>• Field research<br><br>Write "N/A" if the submission does not require an ethics statement.<br><br>General guidance is provided below. Consult the submission guidelines for detailed instructions. **Make sure that all information entered here is included in the Methods section of the manuscript.**	The present study was conducted at the Bangladesh Tea Research Institute (BTRI), Sreemangal-3210, Moulvibazar, Bangladesh, with full logistical support provided by the Institute.

Powered by Editorial Manager® and ProduXion Manager® from Aries Systems Corporation

**Format for specific study types**<br><br>**Human Subject Research (involving human participants and/or tissue)**<br>• Give the name of the institutional review board or ethics committee that approved the study<br>• Include the approval number and/or a statement indicating approval of this research<br>• Indicate the form of consent obtained (written/oral) or the reason that consent was not obtained (e.g. the data were analyzed anonymously)<br><br>**Animal Research (involving vertebrate animals, embryos or tissues)**<br>• Provide the name of the Institutional Animal Care and Use Committee (IACUC) or other relevant ethics board that reviewed the study protocol, and indicate whether they approved this research or granted a formal waiver of ethical approval<br>• Include an approval number if one was obtained<br>• If the study involved *non-human primates*, add *additional details* about animal welfare and steps taken to ameliorate suffering<br>• If anesthesia, euthanasia, or any kind of animal sacrifice is part of the study, include briefly which substances and/or methods were applied<br><br>**Field Research**<br><br>Include the following details if this study involves the collection of plant, animal, or other materials from a natural setting:<br>• Field permit number<br>• Name of the institution or relevant body that granted permission	
**Data Availability**<br><br>Authors are required to make all data underlying the findings described fully available, without restriction, and from the time of publication. PLOS allows rare exceptions to address legal and ethical concerns. See the PLOS Data Policy and FAQ for detailed information.	Yes - all data are fully available without restriction

A Data Availability Statement describing where the data can be found is required at submission. Your answers to this question constitute the Data Availability Statement and **will be published in the article**, if accepted.<br><br>**Important:** Stating 'data available on request from the author' is not sufficient. If your data are only available upon request, select 'No' for the first question and explain your exceptional situation in the text box.<br><br>Do the authors confirm that all data underlying the findings described in their manuscript are fully available without restriction?	
**Describe where the data may be found in full sentences. If you are copying our sample text, replace any instances of XXX with the appropriate details.**<br><br>• If the data are **held or will be held in a public repository**, include URLs, accession numbers or DOIs. If this information will only be available after acceptance, indicate this by ticking the box below. For example: *All XXX files are available from the XXX database (accession number(s) XXX, XXX.*).<br>• If the data are all contained **within the manuscript and/or Supporting Information files**, enter the following: *All relevant data are within the manuscript and its Supporting Information files.*<br>• If neither of these applies but you are able to provide **details of access elsewhere**, with or without limitations, please do so. For example:<br><br>*Data cannot be shared publicly because of [XXX]. Data are available from the XXX Institutional Data Access / Ethics Committee (contact via XXX) for researchers who meet the criteria for access to confidential data.*<br><br>*The data underlying the results presented in the study are available from (include the name of the third party*	All relevant data are within the manuscript and its Supporting Information files.

Powered by Editorial Manager® and ProduXion Manager® from Aries Systems Corporation

and contact information or URL).<br>• This text is appropriate if the data are owned by a third party and authors do not have permission to share the data.<br><br>* typeset	
Additional data availability information:	Tick here if the URLs/accession numbers/DOIs will be available only after acceptance of the manuscript for publication so that we can ensure their inclusion before publication.

Manuscript

Click here to access/download;Manuscript;Riyadh et al., 2026.doc

Full title: Assessment of waterlogging tolerance in tea genotypes through morpho-physiological and biochemical profiling

Short title: Assessment of waterlogging tolerance in tea

Md. Riyadh Arefin[1,2]*, Shitosri Mondal[2], Md. Fazle Rabbi[2], Hafsa Tasnim[2], Md. Alamgir Hossain[2], Md. Sabibul Haque[2], A.K.M. Golam Sarwar[2]

[1] Botany Division, Bangladesh Tea Research Institute, Sreemangal, Moulvibazar, Bangladesh.

[2] Department of Crop Botany, Bangladesh Agricultural University, Mymensingh, Bangladesh.

*** Corresponding author:** Md. Riyadh Arefin

Email: riyadh.btri@gmail.com

Abstract

Tea is one of the major economic crops in Bangladesh; however, its productivity is severely constrained by waterlogging stress arising from erratic climatic changes. The lack of tolerant varieties is a critical barrier to sustainable tea cultivation. Therefore, the present study aimed to investigate the integrated morpho-physiological and biochemical responses of tea genotypes under waterlogging stress to identify tolerant genotype(s). The experiment was conducted at the Bangladesh Tea Research Institute from August to December 2024, following a completely randomized design with three replications. Eight pre-screened tea genotypes—P/LAL/08/23, P/LAL/08/62, P/LAL/09/116, P/AFN/11/35, P/AFN/11/46, P/OTI/31, P/AFN/13/90, P/AFN/11/31, with two control clones, BT2 (popular variety) and TV9 (waterlogging tolerant variety), were evaluated by 23 morpho-physiological and biochemical parameters under two treatments: i) control treatment and ii) stress treatment. The stress treatment was established by exposing the tea genotypes to a waterlogged phase of 14 days, with a 14-day recovery phase. All the morphological traits, like root fresh weight (RFW), leaf number (NL), etc., and physiological characters, such as net photosynthesis (*Pn*), transpiration rate (*E*), stomatal conductance (*gs*), relative leaf water content (RWC), absolute growth rate (AGR), etc., were decreased remarkably under stress condition compared to control. Among the biochemical parameters, leaf pigments, like chlorophyll a (CHA), chlorophyll b (CHB), and total carotenoids (CRTN), were also observed to be reduced, while proline content (leaf: PRLF and root: PRRT), total antioxidant activity (leaf: TACL and root: TACR), and lipid peroxidation (leaf: LOPL and root: LOPR) substantially increased under stress treatment. Out of the 23 evaluated traits, only 11 traits—

RFW, NL, *Pn*, *E*, *gs*, RWC, AGR, CHA, CHB, CRTN, and LPOL were detected as most influential at both phases of stress treatment. Ultimately, the P/AFN/13/90 genotype was identified as the most potentially tolerant genotype, exhibiting better responses than others under both waterlogging and recovery phases.

Introduction

Plants face various types of biotic and abiotic stresses throughout their life cycles [1]. Among the abiotic stresses, waterlogging is one of the major stressors affecting growth and development in plants. Nearly 10-16% of worldwide arable lands are affected by waterlogging, which is becoming a serious threat to agricultural productivity and sustainability [2, 3, 4]. Waterlogging is the state of soil when it is partially or completely saturated by water, replacing air spaces of soil for a particular time [5]. There are several causes of the waterlogging stress, but most of the causes are due to the impact of erratic climate change. Natural climatic causes include excessive and unevenly distributed rainfall, increasing sea level, natural calamities, elevation of groundwater, topography, soil properties, etc. [6]. At the same time, man-made reasons comprise ineffective drainage systems, over-irrigation, improper cultural practices, etc. [7].

Tea (*Camellia sinensis* (L.) O. Kuntze) is one of the major beverage crops that has therapeutic, socio-economic, and cultural value worldwide [8]. Currently, tea is growing in 64 countries in different topographical, climatic, and ecological zones, such as hilly areas [9], plain lands [10], tropical to temperate regions [11], etc. Water is essential for tea growth, but it must not be stagnant. To avoid waterlogging or

stagnant water, normally, tea is grown in hilly areas, but the areas between two adjacent hills, which are termed '*kunchi*' [12], suffer from waterlogging due to the percolation of water underneath. Waterlogging caused by heavy rainfall or floods is the reason behind 15-25% annual loss in North-East India [13], 10% less production in West Bengal and Assam [14], and a serious reduction of crop yield in Sri Lanka [15]. Tea is the most used drink in Bangladesh, supporting 1% of the national gross domestic product (GDP) [16], which also suffers from waterlogging in the Sylhet and Chattogram region [17].

Under waterlogged conditions, plants suffer hypoxia or anoxia and are affected anatomically, morphologically, physiologically, and biochemically, hindering plants' growth and development [18, 19]. Waterlogging can be categorized either based on available oxygen (O_2) in soil, *i.e.,* Hypoxia (decreased O_2), and Anoxia (no available O_2) [20] or based on occurrence pattern or duration of waterlogging, *i.e.*, transient (intermittent) and continuous (long term) [21]. In waterlogging stress, roots suffer primarily from insufficient O_2, plants accumulate reactive oxygen species (ROS) and some toxic metabolites resulting from anaerobic respiration, which disrupts gas exchange capacity by stomatal closure with reduced photosynthesis and ultimately reduces crop yield in majority of crops, including tea [22, 23, 24]. ~~Waterlogging is also detrimental to different growth stages depending on crop species, such as at the flowering, squaring, and boll formation stages (fruit positioning, ball number, boll weight, etc.) in cotton [25] as well as the seedling stage in coffee [26] and coconut [27]. Waterlogging in shrubs and tree species causes leaf chlorosis, leaf dropping, deformed roots, fruit dropping, dieback, stunted growth, and even death [28]. Plants show a series of adaptive strategies, including anatomical, morphological, physiological, biochemical, and molecular mechanisms, to withstand waterlogging~~

stress [28, 29]. Tea plants are affected drastically by waterlogging—morphologically through deformation of roots, leaf yellowing (chlorosis), leaf dropping, decreased plant height, low root-shoot weights or dry matters; physiologically through reduced photosynthesis, stomatal conductance, transpiration and leaf water potential; and biochemically by decreased leaf pigments (chlorophyll contents), Phenylalanine ammonia lyase (PAL) activity, wax content, flavonoids and polyphenols [13, 24, 30, 31]. Waterlogging-tolerant tea genotypes exhibit some changes by developing longer roots and aerenchyma tissues, increasing proline content, wax content, polyphenols, flavonoids, synthesizing antioxidants, etc. [13, 24, 30, 31]. A proper drainage system helps to minimize the waterlogging effect in tea estates, but using tolerant plants is more effective and sustainable. Several states have already developed numerous waterlogging-tolerant tea varieties, such as India (TV9, SNT-10, MNPR/51/P2, GNGA/31/P3, GNGA/31/P4, DFLGR/34/P8) and Sri Lanka (TRI 2023) [15, 32, 33]. Although most tea estates in Bangladesh have recently suffered from waterlogging, till now, there are no tolerant tea varieties available for cultivation to overcome this problem [17]. Focusing this existing problem, the current study was conducted with three primary objectives: (i) to investigate the effect of waterlogging stress on tea plant growth, (ii) to identify most contributing parameters against waterlogging, and (iii) to screen the tolerant tea germplasm(s) effectively through morphological, physiological, and biochemical profiling under waterlogging stress, which can be potentially used as commercial varieties or as valuable potential genetic materials for future breeding programs.

Materials and methods

Genotypes and experimental condition

The experiment was conducted at Bangladesh Tea Research Institute, Sreemangal-3210, Moulvibazar (24.352° N, 91.736° E) from August to December 2024. A total of ten tea genotypes were used in the current study. Among these, eight genotypes, such as P/LAL/08/23 (V1), P/LAL/08/62 (V2), P/LAL/09/116 (V3), P/AFN/11/35 (V4), P/AFN/11/46 (V5), P/OTI/31 (V6), P/AFN/13/90 (V7), P/AFN/11/31 (V8) were selected from a previous study, which were collected from waterlogged areas of different tea estates [17]. The remaining two genotypes were used as check varieties: one was the most popular variety in Bangladesh (BT2) [34], and the other was the renowned waterlogging-tolerant variety from India (TV9) [32, 33].

The experiment was established with two treatments (T_1: control treatment, *i.e.*, no stress, and T_2: stress treatment) following a completely randomized design (CRD) with three replications. 12-month-old saplings of each genotype were exposed to waterlogging stress (T_2). The waterlogged conditions were induced by keeping five plants of each genotype in a plastic box (length: 45.72 cm, width: 30.48 cm, and depth: 30.48 cm) and sealing the boxes with polyethylene sheets to facilitate holding the water. Then, fresh water was added to each box, maintaining the water level at five centimeters above the collar region of plants continuously for 14 days (waterlogging phase). After 14 days of waterlogging phase, water in the boxes was drained out, and plants were kept for another 14 days as a recovery phase. At T_1 treatment (control), no stress was applied; instead, they practiced good cultural practices and ideal irrigation when needed. Five plants of each genotype were considered as a single replicate. Therefore, a total of 500 saplings (250 for control and 250 for stress treatments) were maintained throughout the experimental period.

For both the treatments (T_1 and T_2), data were collected with a seven-day interval under 23 parameters of the following morphological, physiological, and biochemical traits. The complete data collection days with each phase of both stress and control treatments are given in Fig 1.

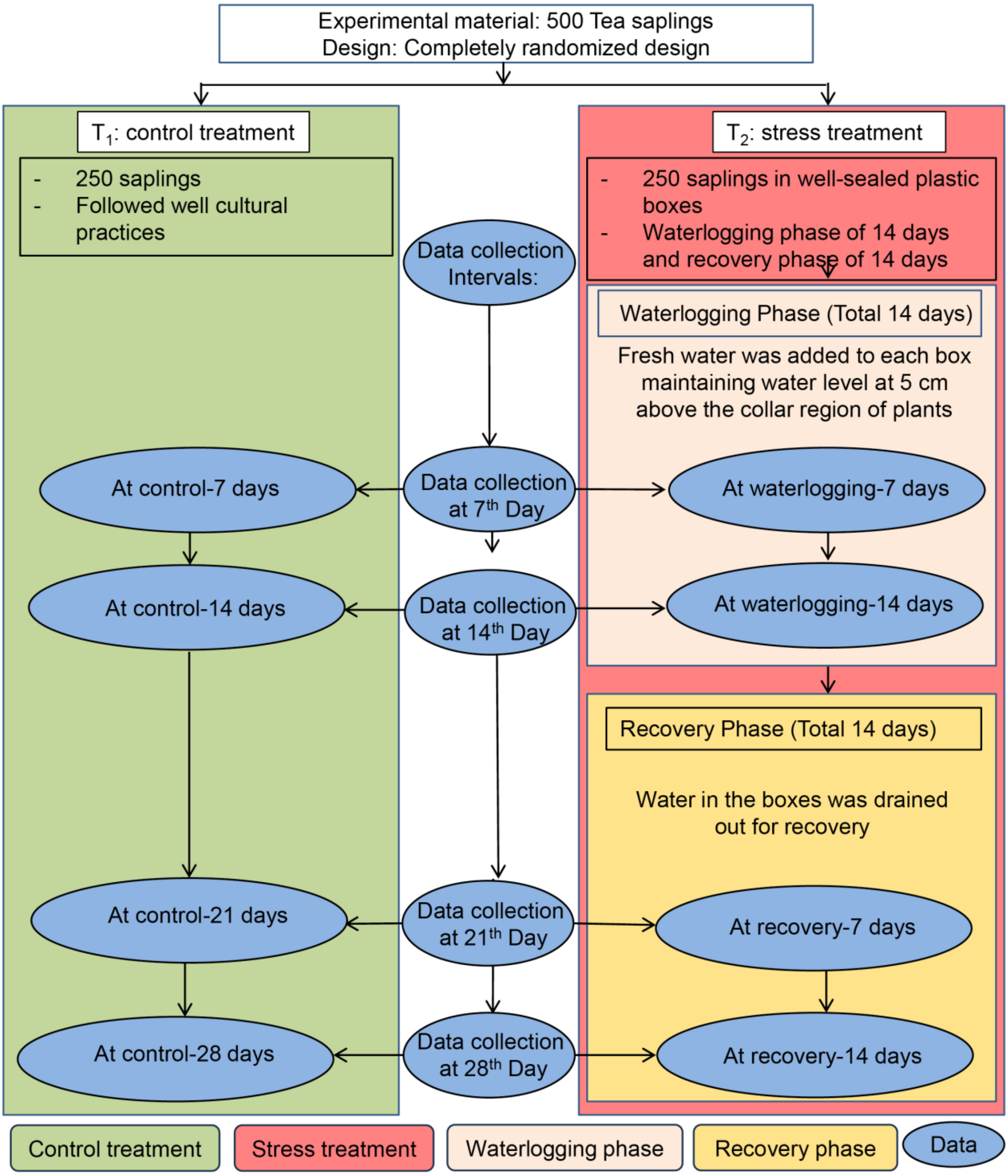

Fig 1. Schematic representation of the experimental methodology for assessing waterlogging tolerance in selected tea genotypes.

Morphological traits

Plant height (PH) was measured in centimeters (cm) by a meter scale from the soil surface of the polybag to the stem apex. Shoot fresh weight (SFW) and root fresh weight (RFW) were recorded by a digital weight scale in grams (g), whereas shoot dry weight (SDW) and root dry weight (RDW) were measured by oven drying of shoots and roots, respectively, at 80°C ± 2°C continuously for 72 hours [35]. Total dry matter (TDM) was calculated by summing the SDW and RDW of respective genotypes. Roots of each plant were carefully washed, and vertical root length (VTRL) was recorded in centimeters (cm) by measuring the longest root. The leaf number of each plant was also recorded and expressed by ~~NL (number of leaves)~~.

Physiological traits

The net photosynthesis (*Pn*), transpiration rate (*E*), stomatal conductance (g_s), relative leaf water content (RWC), absolute growth rate (AGR), and leaf greenness (SPAD) were measured at stress and control environments in all genotypes. The fifth leaf (5th) from the top was tagged for measuring physiological and biochemical traits. The net photosynthesis (*Pn*), transpiration rate (*E*), and stomatal conductance (*gs*) were measured by a portable photosynthesis system (LCi-SD Photosynthetic system, ADC BioScientific Ltd., Herts, UK).

The relative leaf water content (RWC) was determined by taking 0.5 g (FW) mature and disease-free leaves of all genotypes and soaking them in water for 24 hours to obtain turgid weight (TW). Leaves were then dried at 80 ± 2°C for 72 hours, and finally, dry weight (DW) was measured. The RWC (%) was calculated as RWC (%) = {(FW – DW) ÷ (TW – DW)} × 100 formula [36]. The absolute growth rate (AGR) was calculated by AGR = $(W_2 - W_1) \div (T_2 - T_1)$, where $(W_2 - W_1)$ is the difference between the dry weights at T_2 and T_1 [37]. The greenness of leaves (SPAD) was measured by a portable SPAD meter (SPAD-502Plus, Konica Minolta, Osaka, Japan) by averaging three values (at three parts of each leaf), avoiding the midrib region.

Biochemical properties

Leaf pigments like chlorophyll a, chlorophyll b, and total carotenoids were determined by taking 50 mg leaf samples and putting them into 10 ml 80% acetone in the dark for seven days to facilitate the extraction process. The absorbance readings of the extracted solutions were taken at three different wavelengths (470 nm, 646.8 nm, and 663.2 nm) by an ultraviolet-visible (*UV-Vis*) spectrophotometer (LAMBDA 1050+, PerkinElmer, Springfield, United States), and pigments were calculated using the following equations and expressed as mg g^{-1} fresh weight (FW) [38].

Chlorophyll a (CHA) = $12.25\times A_{663.2} - 2.79\times A_{646.8}$

Chlorophyll b (CHB) = $21.50\times A_{646.8} - 5.10\times A_{663.2}$

Total carotenoids (CRTN) = $(1000\times A_{470} - 1.82\times \text{CHA} - 85.02\times \text{CHB}) \div 198$

The leaf (PRLF) and root (PRRT) proline contents of each genotype were measured by ~~Ref.~~ [39] with some slight change [40]. The absorbance was recorded at 520 nm by a similar spectrophotometer and expressed as mg g^{-1} FW. The total antioxidant capacity of leaf (TACL) and root (TACR) was determined by ~~Ref.~~ [41] with some minor changes [40] and expressed as ascorbic acid equivalent (mg eq. AA g^{-1} FW), where the absorbances were taken at 695 nm. Lipid peroxidations in leaf (LPOL) and root (LPOR) were measured by thiobarbituric acid reactive substance (TBARS) assay expressed as malondialdehyde (MDA) content (nmol g^{-1} FW) [42, 43]. The absorbance of prepared samples was measured at 532 nm and 600 nm wavelengths by the same spectrophotometer. The differences between the readings of 532 nm and 600 nm (A_{532} - A_{600}) were estimated to calculate the lipid peroxidation as MDA content with the help of the extinction coefficient of 155 mM^{-1} cm^{-1}.

Statistical analysis of data

The analysis of variance (ANOVA) regarding the parameters was computed by the Minitab software (version 21). The boxplot and correlation matrix graphs were constructed by the *ggplot2* package, where Principal Component Analysis (PCA) was carried out by the *fviz_pca* and *corrplot* packages [44] using RStudio (version 2025.05.1+513 and Released: 05-06-2025) and R (version 4.5.1). The graphs of the percent increase or decrease of each trait in stress condition relative to control were made by SigmaPlot software (Version: 16) developed by Grafiti LLC. The two-way clustering heatmaps with dendrogram and radar plots were graphically created by the software OriginPro (Version: 2025) of OriginLab.

Results

Analysis of variance

Significant variations were observed among traits under different treatments (T_1: control, and T_2: stress) in all genotypes throughout the experiment (S1 Table). Distinct differences were found in all the treatments, while all the genotypes showed notable alternations over the traits except AGR. The maximum number of traits performed significant interaction effects (Genotype × Treatment) except RDW, VRTL, and NL; but the interaction effects were always less than the main two effects (genotypic effect and treatment effect).

Effects of treatments

The overall response of the tea plant under stress conditions (T_2) showed a remarkable variation in morphological performance, physiological functioning, and biochemical stability compared to the control condition (T_1) (Fig 2). Morphological traits (PH, NL, SFW, SDW, RFW, RDW, VRTL, TDM) and physiological traits (AGR, *Pn*, *E*, g_s, RLWC, SPAD) were notably decreased in stress conditions compared to control for all genotypes. Some biochemical traits (CHA, CHB, and CRTN) also decreased, but most of the biochemical traits, such as PRLF, PRRT, TACL, TACR, LPOL, and LPOR, substantially increased in the stress condition compared to the control environment.

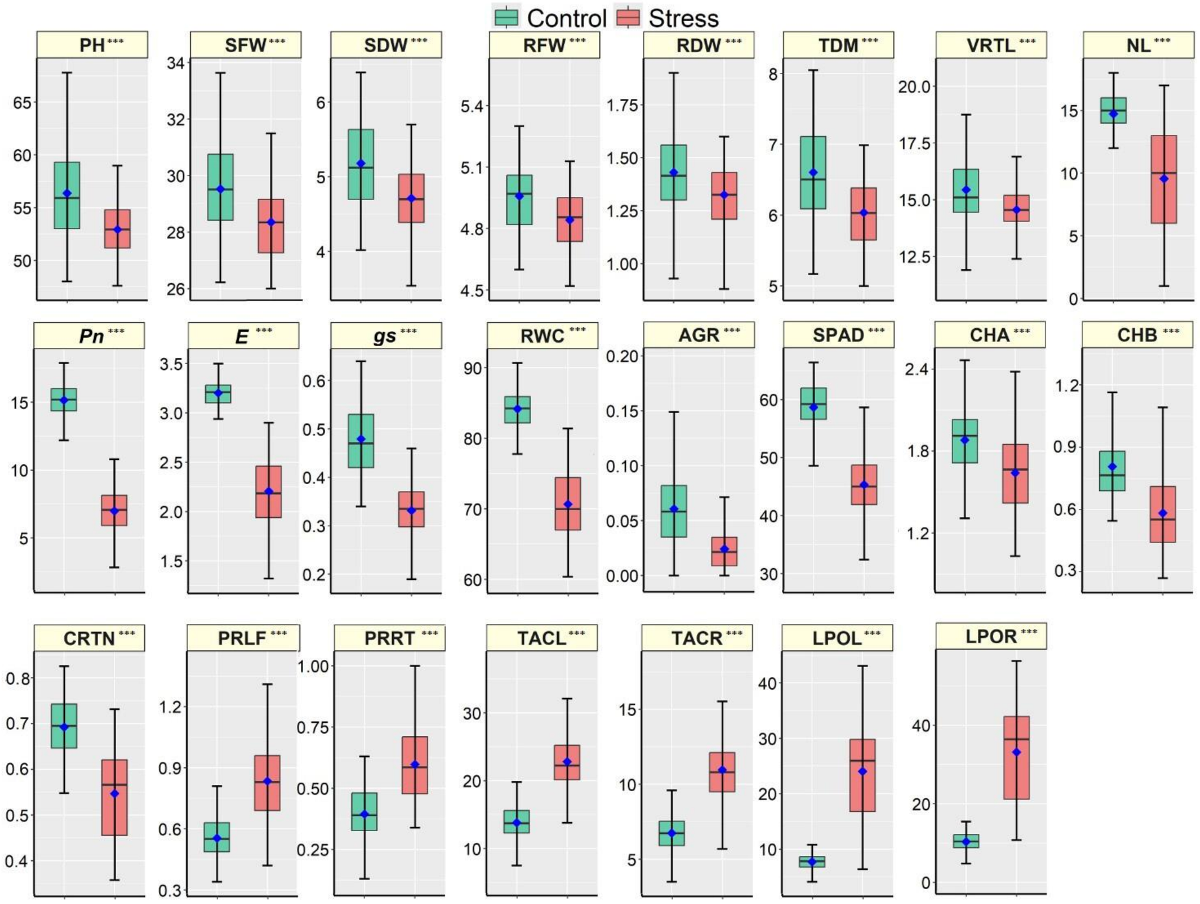

Fig 2. Responses of ten genotypes for 23 traits under control and stress treatments. PH: Plant height (cm), SFW: Shoot fresh weight (g), SDW: Shoot dry weight (g), RFW: Root fresh weight (g), RDW: Root dry weight (g), TDM: Total dry matter (g), VRTL: Vertical root length (cm), NL: Number of leaves per plant, *Pn*: Net photosynthesis (µmol CO_2 m^{-2} s^{-1}), *E*: Transpiration rate (mol H_2O m^{-2} s^{-1}), *gs*: Stomatal conductance (mmol m^{-2} s^{-1}), RWC: Percent relative leaf water content, AGR: Absolute growth rate, SPAD: SPAD value of leaves, CHA: Chlorophyll a (mg g^{-1} FW), CHB: Chlorophyll b (mg g^{-1} FW), CRTN: Total carotenoids (mg g^{-1} FW), PRLF: Proline content in leaf (mg g^{-1} FW), PRRT: Proline content in root (mg g^{-1} FW), TACL: Total antioxidant capacity of leaf (mg eq. AA g^{-1} FW), TACR: Total antioxidant capacity of root (mg eq. AA g^{-1} FW), LPOL: Lipid peroxidation of leaf

(MDA content nmol g^{-1} FW) and LPOR: Lipid peroxidation of root (MDA content nmol g^{-1} FW). The '***' stands for the level of significance at p≤ '0.1%'.

Interaction effects of genotypes and treatments

The percent change (increase or decrease) of all traits under stress conditions compared to the control was discussed below under the following heads (Figs 3-5) to identify the tolerant genotype(s). The responses of genotypes under different treatments were mentioned in S1-S23 Figs for all measured parameters.

Morphological traits

The morphological traits in all tea genotypes were significantly influenced by waterlogging stress. All genotypes showed a substantial reduction in morphological traits compared to the control, and the extent of decline increased with the duration of the stress. The decline became more prominent in the recovery phases due to the stunted growth in stress conditions compared to profuse growth in control (Fig 3). The genotype-specific responses under both control and stress conditions across all the observational intervals were provided in S1-S8 Figs.

Among the evaluated genotypes, V7 exhibited the lowest reduction in plant height (PH) with the decreases of only 1.73% and 8.90% at Waterlog-14 days and Recovery-14 days, respectively, compared to the control (Fig 3A). A comparable trend was observed for shoot fresh weight (SFW), where V7 showed the least decline (2.04%) under Waterlog-14 days, and V4 performed best during Recovery-14

days (3.08% reduction) (Fig 3B). For shoot dry weight (SDW), V2 exhibited greater stability at Waterlog-14 days with 3.48% reduction, whereas V4 maintained the minimum decrease of 9.45% under Recovery-14 days (Fig 3C). The root fresh weight (RFW) was least affected in V5 with the declines of 0.62% and 0.80% at Waterlog-14 days and Recovery-14 days, respectively (Fig 3D). The smallest decrease in root dry weight (RDW) was detected in V3 under Waterlog-14 days (3.60%) and V5 (12.08%) at Recovery-14 days (Fig 3E). Regarding total dry matter (TDM), V2 (4.01%) showed lower decline at Waterlog-14 days, but V4 (10.20%) exhibited the least decrease at Recovery-14 days (Fig 3F). Again, the least decrease in vertical root length (VRTL) was observed in V4 at Waterlog-14 days (1.18%) and in V7 at Recovery-14 days (6.77%) (Fig 3G). In case of number of leaves (NL), V7 consistently showed superior tolerance with lower declines of 13.64% during Waterlog-14 days and 15.56% during Recovery-14 days in comparison to the control condition (Fig 3H).

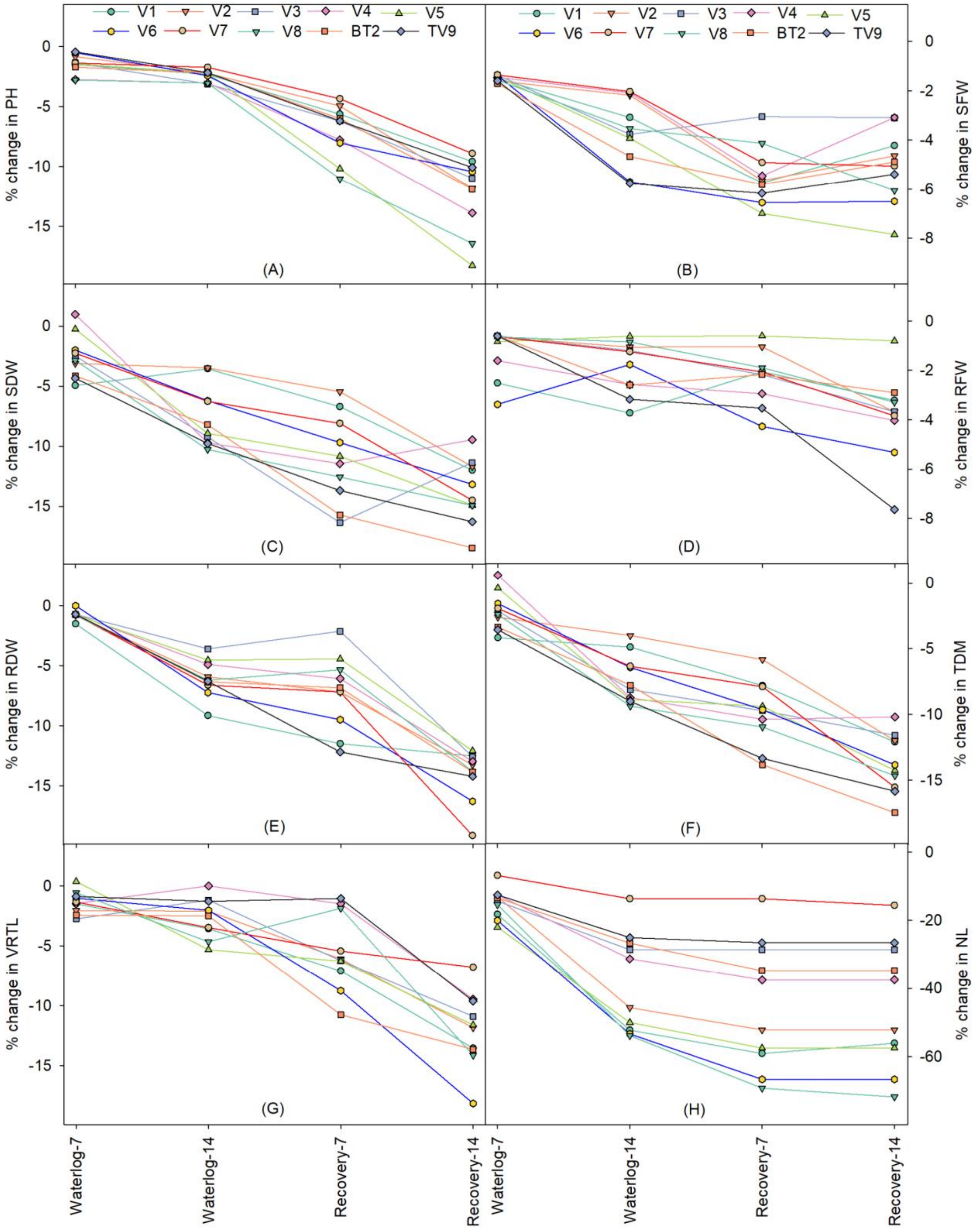

Fig 3. % changes in morphological traits in relation to control at different phases of waterlogging and recovery conditions - (A) PH: Plant height, (B) SFW: Shoot fresh weight, (C) SDW: Shoot dry weight, (D) RFW: Root fresh weight, (E) RDW: Root dry weight, (F) TDM: Total dry matter, (G) VRTL: Vertical root length and (H) NL: Number

of leaves per plant of 10 genotypes under stress condition compared to control condition.

Physiological traits

Waterlogging stress exerted a pronounced effect on the physiological attributes in tea saplings (S9-S14 Fig). At Waterlog-14 days, net photosynthesis (*Pn*) decreased to a minimum of 55.89% and 38.80% at Waterlogging-14 days and Recovery-14 days, respectively, in V7 (Fig 4A). Likely *Pn*, the transpiration rate (*E*) was also impaired by stress conditions, where the least decreases compared to control during Waterlog-14 days and Recovery-14 days were noticed in V6 by 30.77% and 22.19%, respectively (Fig 4B). Again, the least declines in stomatal conductance (*gs*) were observed in V7 at both Waterlog-14 days (32.56%) and Recovery-14 days (17.50%) (Fig 4C). The minimum reduction for relative leaf water content (RWC) was detected in V4 (17.53%) at Waterlog-14 days and in V7 (11.41%) at Recovery-14 days compared to control (Fig 4D). The more stable absolute growth rate (AGR) was noticed in V1 during Waterlog-14 days with a minimum reduction of 29.62% and in V4 during Recovery-14 days (4.56%) (Fig 4E). In terms of SPAD value, V3 demonstrated the lowest decrease of 19.78% at Waterlog-14 days; however, V7 showed comparatively higher tolerance with a minimum decline of 14.96% at Recovery-14 days compared to the control condition (Fig 4F).

Biochemical traits

Distinct biochemical variations were recorded across the treatments (T_1 and T_2) throughout the study (S15-S23 Figs). At waterlog-14 days, Chlorophyll a (CHA) declined least in V4 (5.95%), followed by V7 (9.12%), whereas, Recovery-14 days, V3 (6.85%) and V7 (7.12%) exhibited lower reduction compared to control (Fig 5A). For chlorophyll b (CHB), V7 consistently showed the least decrease at both Waterlog-14 days (25.77%) and Recovery-14 days (16.67%) (Fig 5B). Similarly, total carotenoids (CRTN) decreased minimally in V4 (3.75%) at Waterlog-14 days and in V7 (12.63%) at Recovery-14 days (Fig 5C).

Elevated proline content was observed under stress treatment (T_2) compared with the control (T_1). Leaf proline concentration (PRLF) increased most in V2 at Waterlog-14 days (106.38%) and in BT2 at Recovery-14 days (61.40%) (Fig 5D), while the amount of root proline (PRRT) showed maximum elevation in V5 Waterlog-14 days (159.09%) and in V4 at Recovery-14 days (61.22%) (Fig 5E). Notably, V7 and V4 accumulated the highest leaf proline (1.32 mg g^{-1} FW) and root proline (0.94 mg g^{-1} FW) during Waterlogg-14 days (S18 and S19 Figs).

Total antioxidant capacity (TAC) was found to be increased under stress conditions as a defensive mechanism. Total antioxidant capacity in leaf (TACL) increased most in V7 at Waterlog-14 days (108.83%) and V8 at Recovery-14 days (81.05%) (Fig 5F). In roots, the highest antioxidant capacity (TACR) was observed in V7 at both Waterlog-14 days (101.94%) and Recovery-14 days (68.44%) (Fig 5G). Under severe waterlogging stress, V7 demonstrated the highest TAC in both leaf (34.54 mg eq. AA g^{-1} FW) and root (28.84 mg eq. AA g^{-1} FW) (S20 and S21 Figs).

Elevated lipid peroxidation with disrupted cellular integrity was also detected under stress conditions (T_2). But the least increase of the lipid peroxidation in leaf (LPOL)

was noticed in V3 at Waterlog-14 days (240.65%) and at Recovery-14 days (193.68%) (Fig 5H). Again, in the root, the minimum decline of lipid peroxidation (LPOR) was noticed in TV9 (223.25%) and V3 (151.08%) at Waterlog-14 days and Recovery-14 days, respectively, with the comparison to the control condition (Fig 5I).

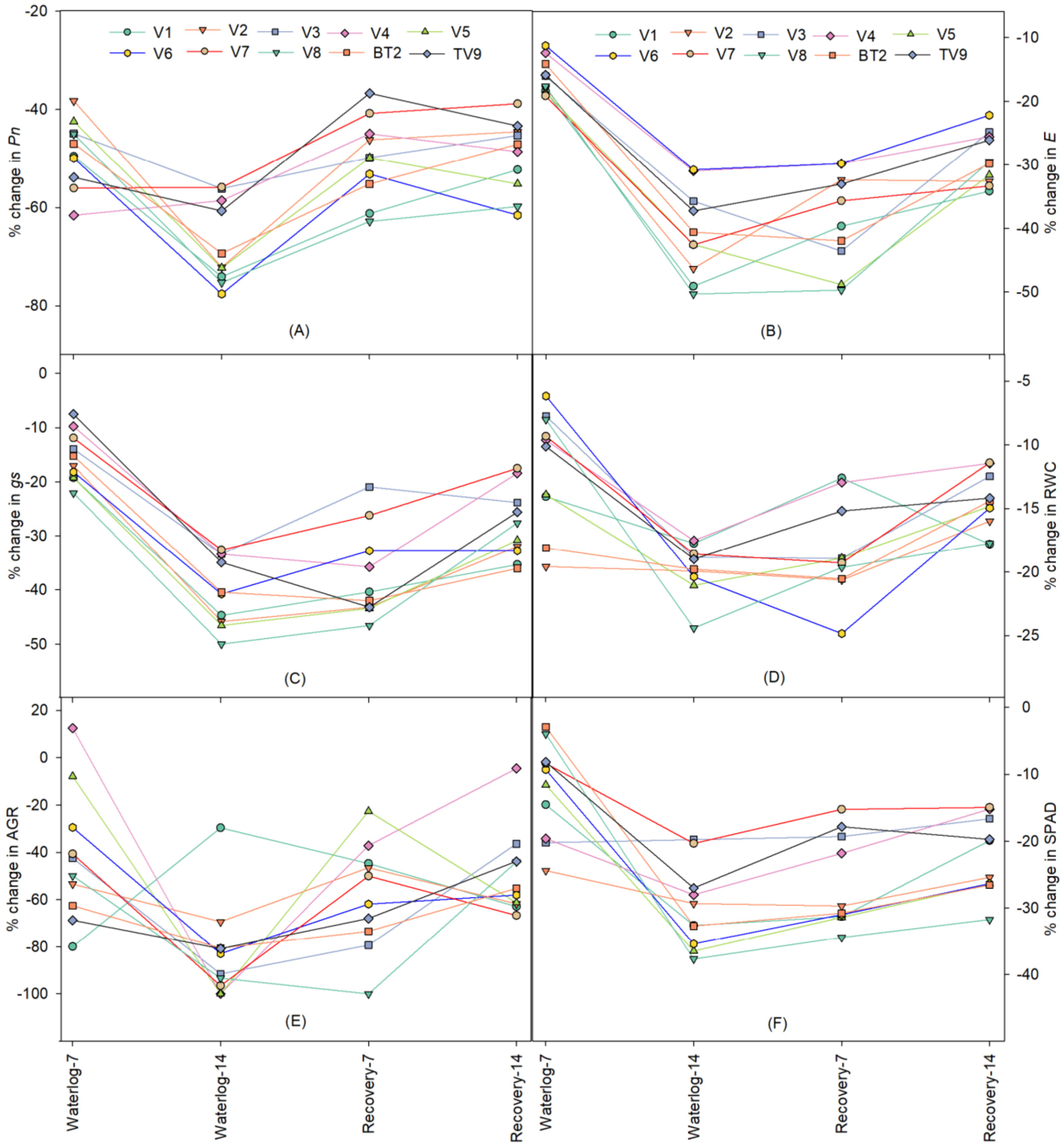

Fig 4. Percent changes in physiological parameters- (A) *Pn*: net photosynthesis, (B) *E*: Transpiration rate, (C) *gs*: Stomatal conductance, (D) RWC: Percent relative leaf

water content, (E) AGR: Absolute growth rate and (F) SPAD: SPAD value of leaves of 10 genotypes under stress treatment in relative to control.

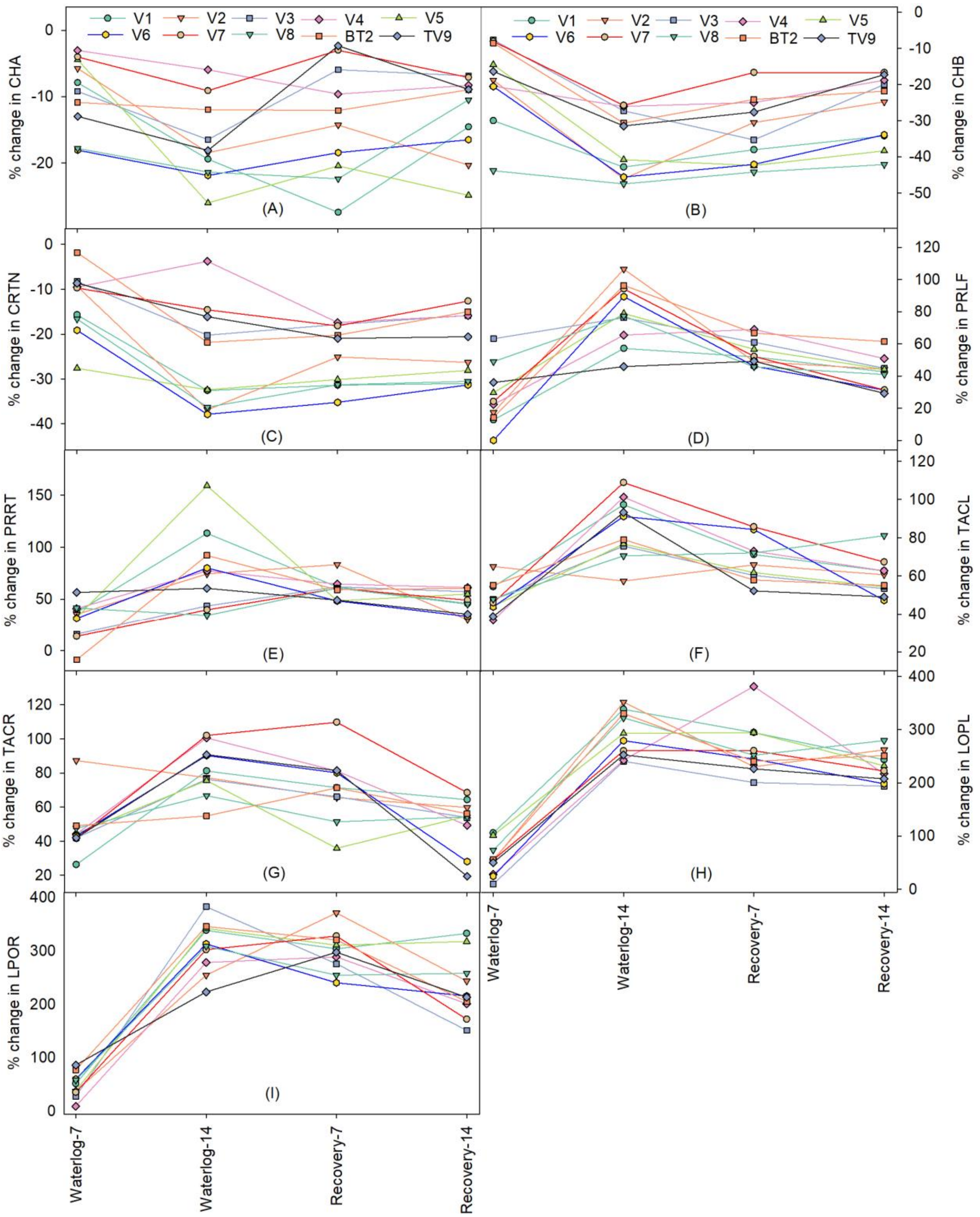

Fig 5. Biochemical changes (%) in- (A) CHA: Chlorophyll a, (B) CHB: Chlorophyll b, (C) CRTN: Total carotenoids, (D) PRLF: Proline content in leaf, (E) PRRT: Proline content in root, (F) TACL: Total antioxidant capacity of leaf, (G) TACR: Total antioxidant capacity of root, (H) LPOL: Lipid peroxidation of leaf and (I) LPOR: Lipid peroxidation of root in ten genotypes under stress condition compared to control condition.

Principal Component Analysis (PCA)

Principal component analysis (PCA) was performed for both waterlogging and recovery phases at 14th days to determine the traits that were more responsible for the tolerance against waterlogging stress based on the per cent changes in the stress condition against control (Tables 1 and 2, Figs 6 and 7, S24-S27 Figs). At Waterlog-14 days, the first seven PCs (PC1 to PC7) possessed eigenvalues greater than one, and these seven PCs cumulatively explained 93.20% of the entire variations (Table 1). The PC1 and PC2 were responsible for 39.16% and 18.65% of total variance, respectively, while cumulatively designated for 57.81% of total variance (Table 1, Fig 6). Some degree of relationship between the traits can also be predicted from the direction of arrows in the biplot (Fig 6). Generally the same direction in a biplot implies positive relationship, like RWC and TACL, TACL and TACR, CHA and SPAD, *gs* and SPAD, *Pn* and CHB, LPOL and PRRT, AGR and TDM, PH and TDM, etc. (Fig 6). Opposite direction of arrows generally is the indication of a negative relationship, such as RDW and TDM, RDW and SDW, AGR and RDW, *E* and LPOL, *gs* and LPOL, *Pn* and LPOL, PRLF and TACL, PRLF and TACR, SPAD and PRRT, LPOL and CRTN (Fig 6C) etc. The genotypes V7, TV9 and

V3, V4 were placed in the 1st and 4th quadrants of the PCA-biplot at Waterlog-14 days, respectively (Fig 6). Traits such as *gs*, *Pn*, CRTN, CHB, NL, LPOL, SPAD, CHA, *E* and RWC highly influenced the PC1, while SDW, TDM, AGR, RDW, RWC, RFW and PH mainly contributed to PC2 at Waterlog-14 days (S24 and S25 Figs).

Table 1. Eigenvalue, percent (%) variance, and cumulatively percent (%) variance of corresponding principal components (PCs) of 10 genotypes for 23 traits under waterlogging phase.

Principal components (PCs)	Eigenvalue	Variance (%)	Cumulatively variance (%)
PC1	9.01	39.16	39.16
PC2	4.29	18.65	57.81
PC3	2.64	11.49	69.30
PC4	1.56	6.78	76.08
PC5	1.43	6.24	82.32
PC6	1.35	5.86	88.18
PC7	1.15	5.02	93.20
PC8	0.82	3.53	96.73
PC9	0.75	3.27	100

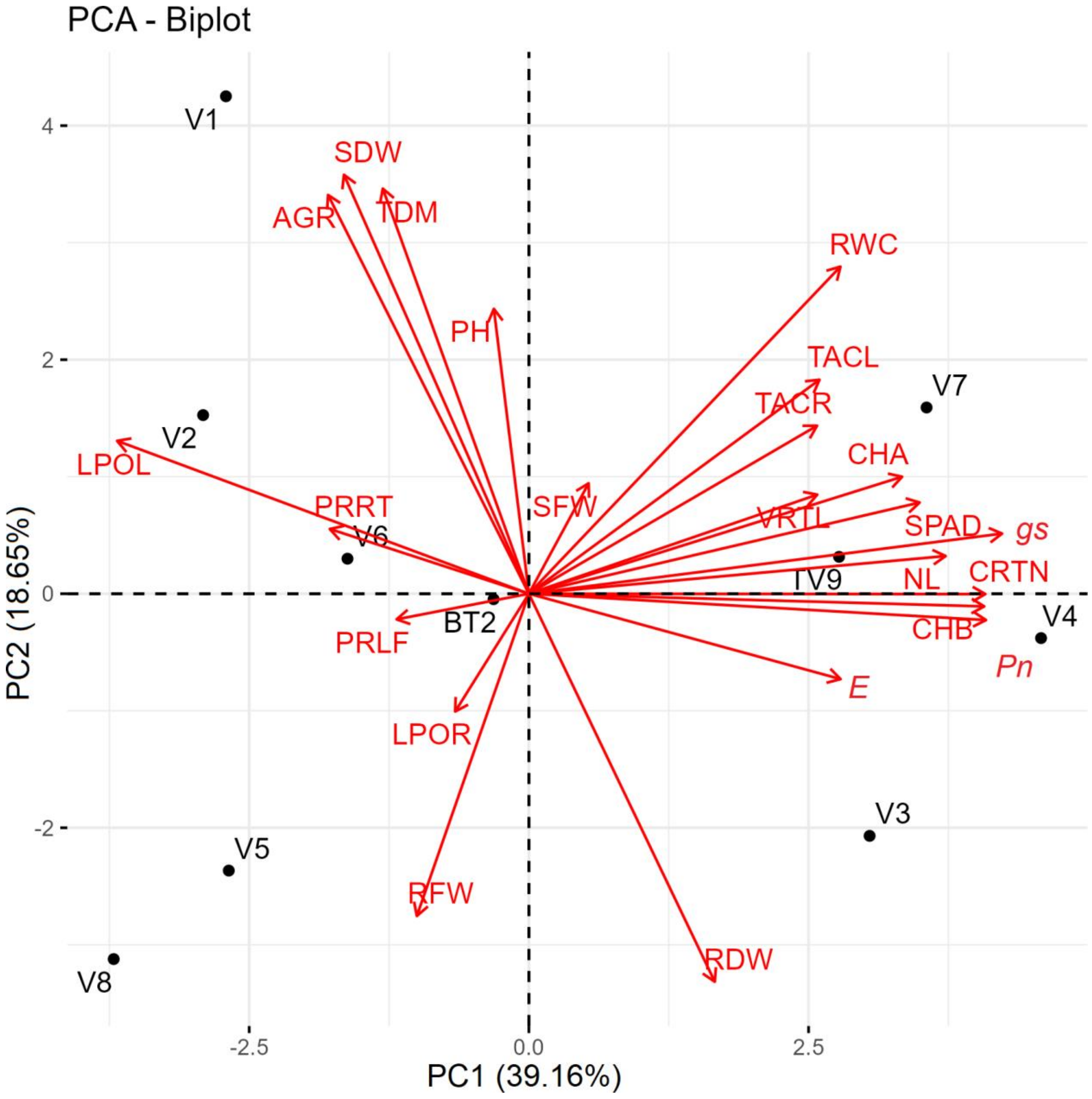

Fig 6. Principal component analysis (PCA)-biplot exhibits the interrelation among the traits and genotypes at the 14th day of the waterlogging phase. PH: Plant height (cm), SFW: Shoot fresh weight (g), SDW: Shoot dry weight (g), RFW: Root fresh weight (g), RDW: Root dry weight (g), TDM: Total dry matter (g), VRTL: Vertical root length (cm), NL: Number of leaves per plant, *Pn*: Net photosynthesis (µmol CO_2 m^{-2} s^{-1}), *E*: Transpiration rate (mol H_2O m^{-2} s^{-1}), *gs*: Stomatal conductance (mmol m^{-2} s-

^{1}), RWC: Percent relative leaf water content, AGR: Absolute growth rate, SPAD: SPAD value of leaves, CHA: Chlorophyll a (mg g^{-1} FW), CHB: Chlorophyll b (mg g^{-1} FW), CRTN: Total carotenoids (mg g^{-1} FW), PRLF: Proline content in leaf (mg g^{-1} FW), PRRT: Proline content in root (mg g^{-1} FW), TACL: Total antioxidant capacity of leaf (mg eq. AA g^{-1} FW), TACR: Total antioxidant capacity of root (mg eq. AA g^{-1} FW), LPOL: Lipid peroxidation of leaf (MDA content nmol g^{-1} FW) and LPOR: Lipid peroxidation of root (MDA content nmol g^{-1} FW).

On the 14th day of the recovery phase, the first six PCs had eigenvalues more than 1, contributing 92.73% of the overall variations (Table 2). The PC1 and PC2 possessed higher eigenvalues of 8.71 and 4.04, respectively, which cumulatively described more than half of the total variance (55.42%) (Table 2, Fig 7). The PC1 contributed 37.86%, meanwhile PC2 contributed 17.56% of the total variation (Table 2, Fig 7). Positive correlations were observed in VRTL and SFW, CRTN and *Pn*, *gs* and *Pn*, RWC and NL, NL and AGR, AGR and RWC, TACR and PRLF, TACR and TACL, LPOL and LPOR, RFW and LPOL, RFW, LPOR, and RFW (Fig 7), etc. On the contrary, negative associations were detected between LPOL and PH, LPOR and PH, *E* and RFW, *gs* and RDW, NL and LPOR, NL and LPOL, RWC and LPOL, RWC and LPOR, CHA and LPOL, CHB and LPOL, CRTN and LPOL, CHA and LPOR, CHB and LPOR, CRTN and LPOR, *Pn* and *E,* etc. (Fig 7). The first quadrant possessed three genotypes like V3, V4, and V7, whereas the fourth quadrant had only one genotype, TV9 (Fig 7). In Recovery-14 days, several traits, such as CHB, NL, CRTN, AGR, RWC, LPOR, *Pn*, *gs*, CHA, VRTL, and SFW affected PC1 mostly, whereas TACR, RFW, PRLF, PRRT, *E*, TACL, and LPOL were the main contributing characters for PC2 (S26 and S27 Figs).

Table 2. Eigenvalue, percent (%) variance, and cumulatively percent (%) variance of corresponding principal components (PCs) of 10 genotypes for 23 traits at Recovery-14 days.

Principal components (PCs)	Eigenvalue	Variance (%)	Cumulatively variance (%)
PC1	8.71	37.86	37.86
PC2	4.04	17.56	55.42
PC3	3.26	14.18	69.6
PC4	2.19	9.53	79.13
PC5	1.66	7.2	86.33
PC6	1.47	6.4	92.73
PC7	0.95	4.13	96.86
PC8	0.54	2.34	99.2
PC9	0.19	0.8	100

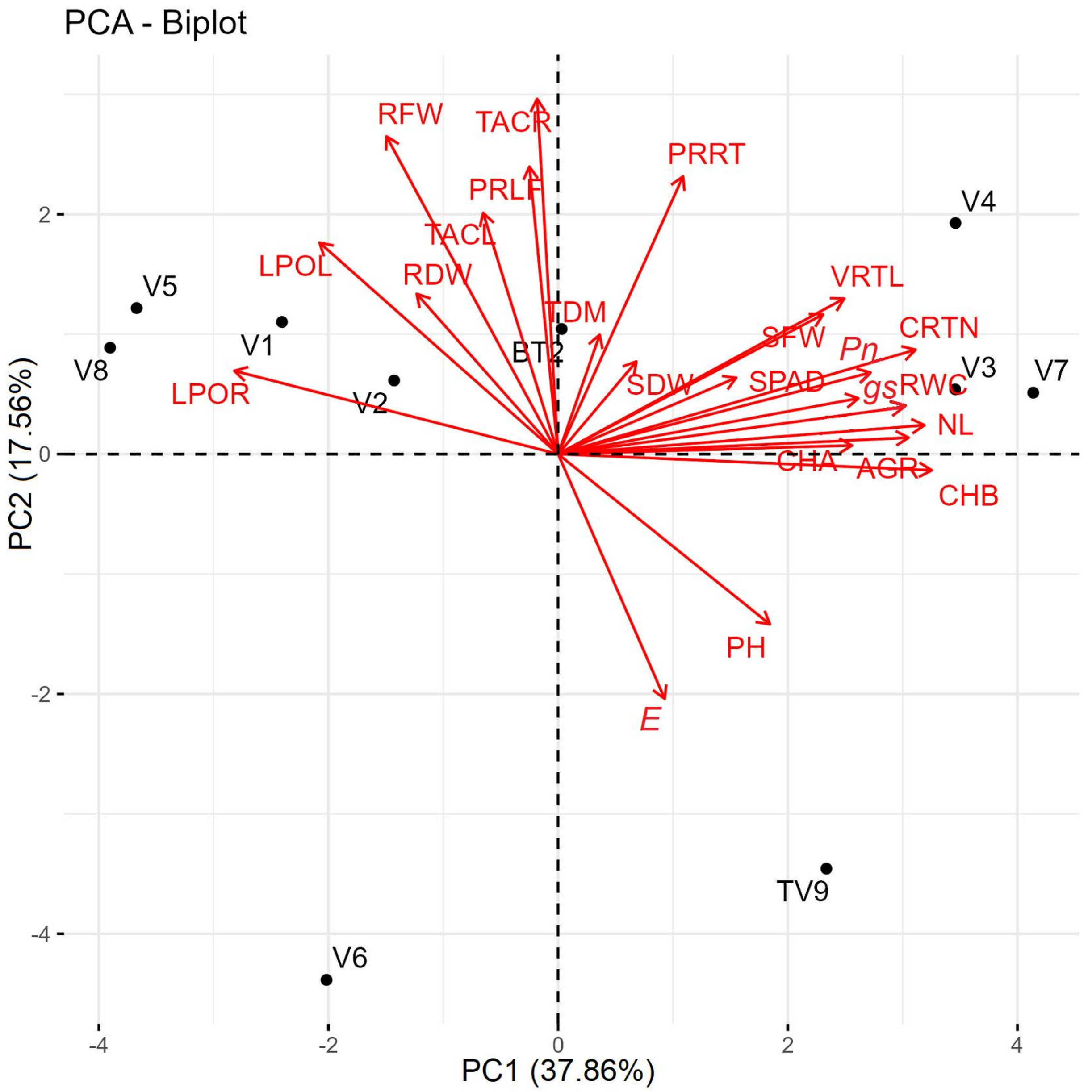

Fig 7. Principal component analysis (PCA)-biplot exhibits the interrelation among the traits and genotypes under recovery phase. PH: Plant height (cm), SFW: Shoot fresh weight (g), SDW: Shoot dry weight (g), RFW: Root fresh weight (g), RDW: Root dry weight (g), TDM: Total dry matter (g), VRTL: Vertical root length (cm), NL: Number of leaves per plant, *Pn*: Net photosynthesis (µmol CO_2 m^{-2} s^{-1}), *E*: Transpiration rate (mol H_2O m^{-2} s^{-1}), *gs*: Stomatal conductance (mmol m^{-2} s^{-1}), RWC: Percent relative leaf water content, AGR: Absolute growth rate, SPAD: SPAD value of leaves, CHA: Chlorophyll a (mg g^{-1} FW), CHB: Chlorophyll b (mg g^{-1} FW), CRTN: Total

carotenoids (mg g^{-1} FW), PRLF: Proline content in leaf (mg g^{-1} FW), PRRT: Proline content in root (mg g^{-1} FW), TACL: Total antioxidant capacity of leaf (mg eq. AA g^{-1} FW), TACR: Total antioxidant capacity of root (mg eq. AA g^{-1} FW), LPOL: Lipid peroxidation of leaf (MDA content nmol g^{-1} FW) and LPOR: Lipid peroxidation of root (MDA content nmol g^{-1} FW).

Correlation Analysis of the Measured Traits

The degree and type of the relationships among the measured traits at stress conditions (both waterlogging and recovery phase) were determined by their percent change relative to the control condition. In waterlogging stress on 14 days, several positive and negative correlations were noticed within the traits (Fig 8). Diversified significant, relationship such as, SDW positively with TDM and AGR; RDW negatively with AGR; TDM positively with AGR; VRTL positively with *E* and *gs*; NL positively with physiological parameters (*Pn*, *gs*, SPAD) and plant pigments (CHA, CHB and CRTN) but negatively with LPOL; *Pn* positively with *gs* and plant pigments (CHA, CHB and CRTN) but negatively with LPOL; *E* positively with *gs* but negatively with LPOL; *gs* positively with RWC and plant pigments but negatively with LPOL; SPAD positively with plant pigments; plant pigments positively with each other; and TACL positively with TACR but negatively with LPOL; TACR negatively with both LPOL were observed on 14th days of waterlogging phase.

In contrast, various sorts of significant associations were also observed among the described traits under the recovery phase (Fig 9). SFW is notably associated with RWC and CHA; SDW strongly positively with TDM; RFW highly relied on TACR;

VRTL positively relied on most of the parameters except *E*, LPOL, and LPOR; NL positively associated with VRTL, several physiological parameters (*Pn*, RWC, AGR), and leaf pigments, while negatively with LPOR; *Pn* positively correlated with RWC and leaf pigments; *gs*, RWC, and AGR positively with each other; leaf pigments correlated with each other but negatively with LPOR.

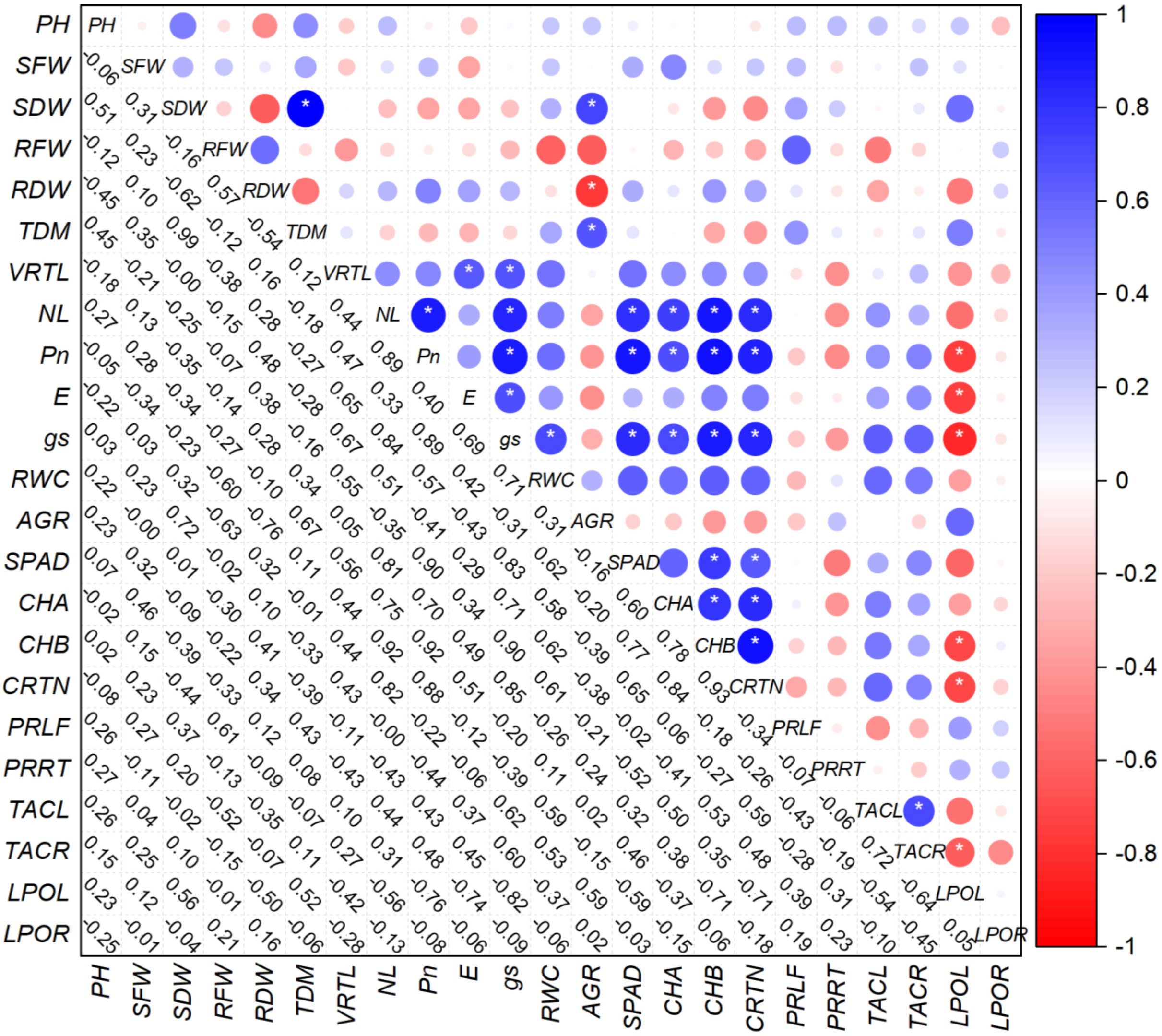

Fig 8. Correlation matrix showing the degree of relationship among the 23 measured traits during the waterlogging phase. The degree of relationship is explained by the color gradient of Pearson’s correlation scale of -1 (deep red color) to 1 (deep blue). PH: Plant height (cm), SFW: Shoot fresh weight (g), SDW: Shoot dry weight (g),

RFW: Root fresh weight (g), RDW: Root dry weight (g), TDM: Total dry matter (g), VRTL: Vertical root length (cm), NL: Number of leaves per plant, *Pn*: Net photosynthesis (µmol CO_2 m^{-2} s^{-1}), *E*: Transpiration rate (mol H_2O m^{-2} s^{-1}), *gs*: Stomatal conductance (mmol m^{-2} s^{-1}), RWC: Percent relative leaf water content, AGR: Absolute growth rate, SPAD: SPAD value of leaves, CHA: Chlorophyll a (mg g^{-1} FW), CHB: Chlorophyll b (mg g^{-1} FW), CRTN: Total carotenoids (mg g^{-1} FW), PRLF: Proline content in leaf (mg g^{-1} FW), PRRT: Proline content in root (mg g^{-1} FW), TACL: Total antioxidant capacity of leaf (mg eq. AA g^{-1} FW), TACR: Total antioxidant capacity of root (mg eq. AA g^{-1} FW), LPOL: Lipid peroxidation of leaf (MDA content nmol g^{-1} FW) and LPOR: Lipid peroxidation of root (MDA content nmol g^{-1} FW). The '*' stands for the level of significance at p≤ '5%'.

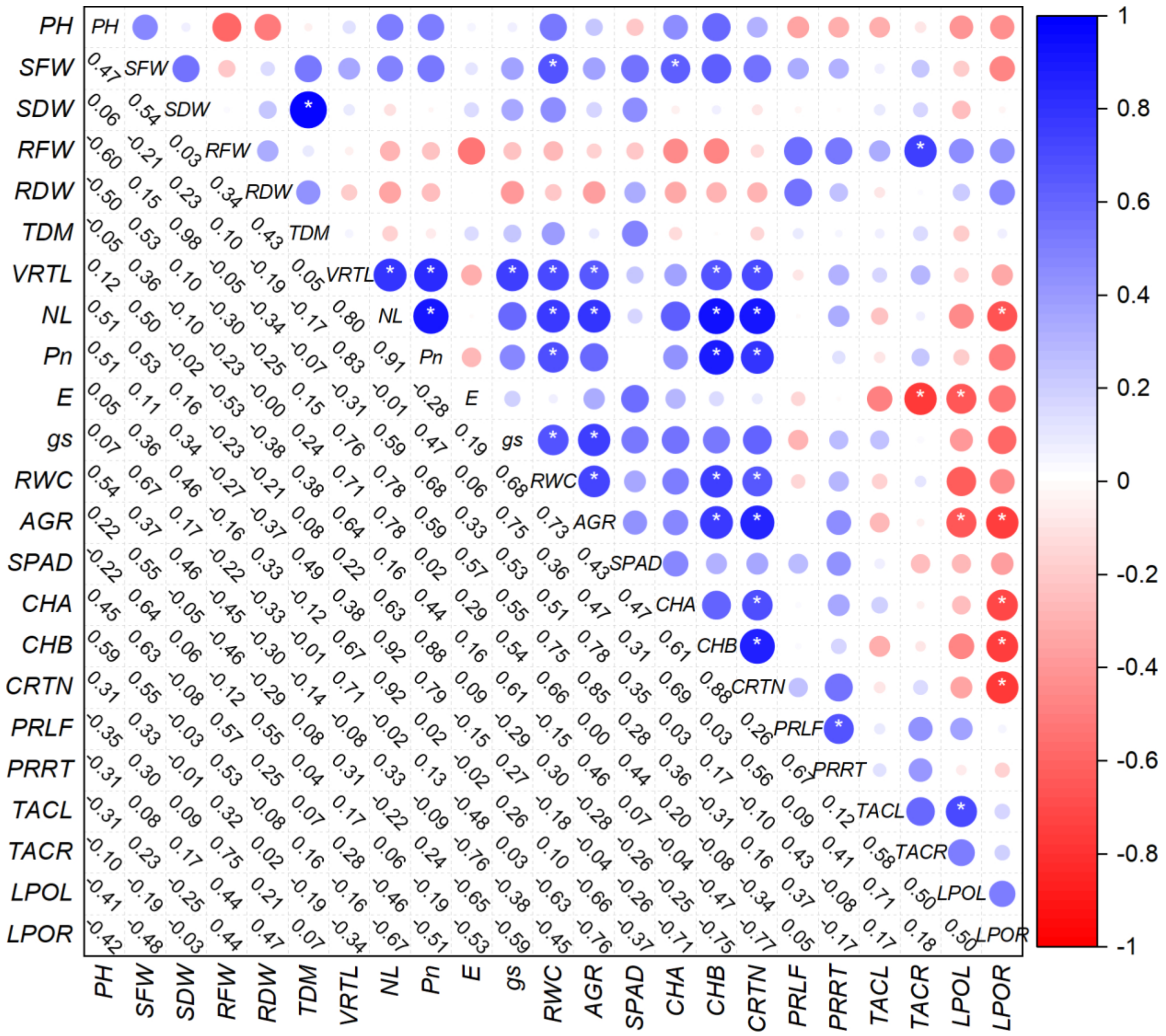

Fig 9. Correlation matrix showing the degree of relationship among the 23 measured traits during the recovery phase. The degree of relationship is explained by the color gradient of Pearson's correlation scale of -1 (deep red color) to 1 (deep blue). PH: Plant height (cm), SFW: Shoot fresh weight (g), SDW: Shoot dry weight (g), RFW: Root fresh weight (g), RDW: Root dry weight (g), TDM: Total dry matter (g), VRTL: Vertical root length (cm), NL: Number of leaves per plant, *Pn*: Net photosynthesis (µmol CO_2 m^{-2} s^{-1}), *E*: Transpiration rate (mol H_2O m^{-2} s^{-1}), *gs*: Stomatal conductance (mmol m^{-2} s^{-1}), RWC: Percent relative leaf water content, AGR: Absolute growth rate, SPAD: SPAD value of leaves, CHA: Chlorophyll a (mg g^{-1} FW), CHB: Chlorophyll b (mg g^{-1} FW), CRTN: Total carotenoids (mg g^{-1} FW), PRLF:

Proline content in leaf (mg g^{-1} FW), PRRT: Proline content in root (mg g^{-1} FW), TACL: Total antioxidant capacity of leaf (mg eq. AA g^{-1} FW), TACR: Total antioxidant capacity of root (mg eq. AA g^{-1} FW), LPOL: Lipid peroxidation of leaf (MDA content nmol g^{-1} FW) and LPOR: Lipid peroxidation of root (MDA content nmol g^{-1} FW). The '*' stands for the level of significance at p≤ '5%'.

Classification of traits and genotypes

All the measured traits and genotypes in the current study were classified into several clusters and groups by performing a two-way hierarchical clustering heatmap with a dendrogram for both the waterlogging phase (Fig 10) and the recovery phase (Fig 11). The heatmaps were prepared by the percent increase or decrease of the traits at 14th days under stress conditions (T_2) compared to the control condition (T_1). After the end of the waterlogging phase, the measured traits were grouped into three groups (G-1, G-2, and G-3) (Fig 10). G-3 consisted of PRRT, PH, LPOL, SDW, TDM, and AGR, while G-2 comprised of PRLF, RFW, RDW, LPOR, SFW, and the rest of the traits placed in G-1. The genotypes were placed into two clusters (C-1 and C-2) based on their performances in the waterlogging phase compared to the control condition (Fig 9). C-1 included the genotypes V7, TV9, V4 and V3 meanwhile rest of the genotypes (BT2, V6, V8, V5, V2, V1) were kept in C-2 cluster.

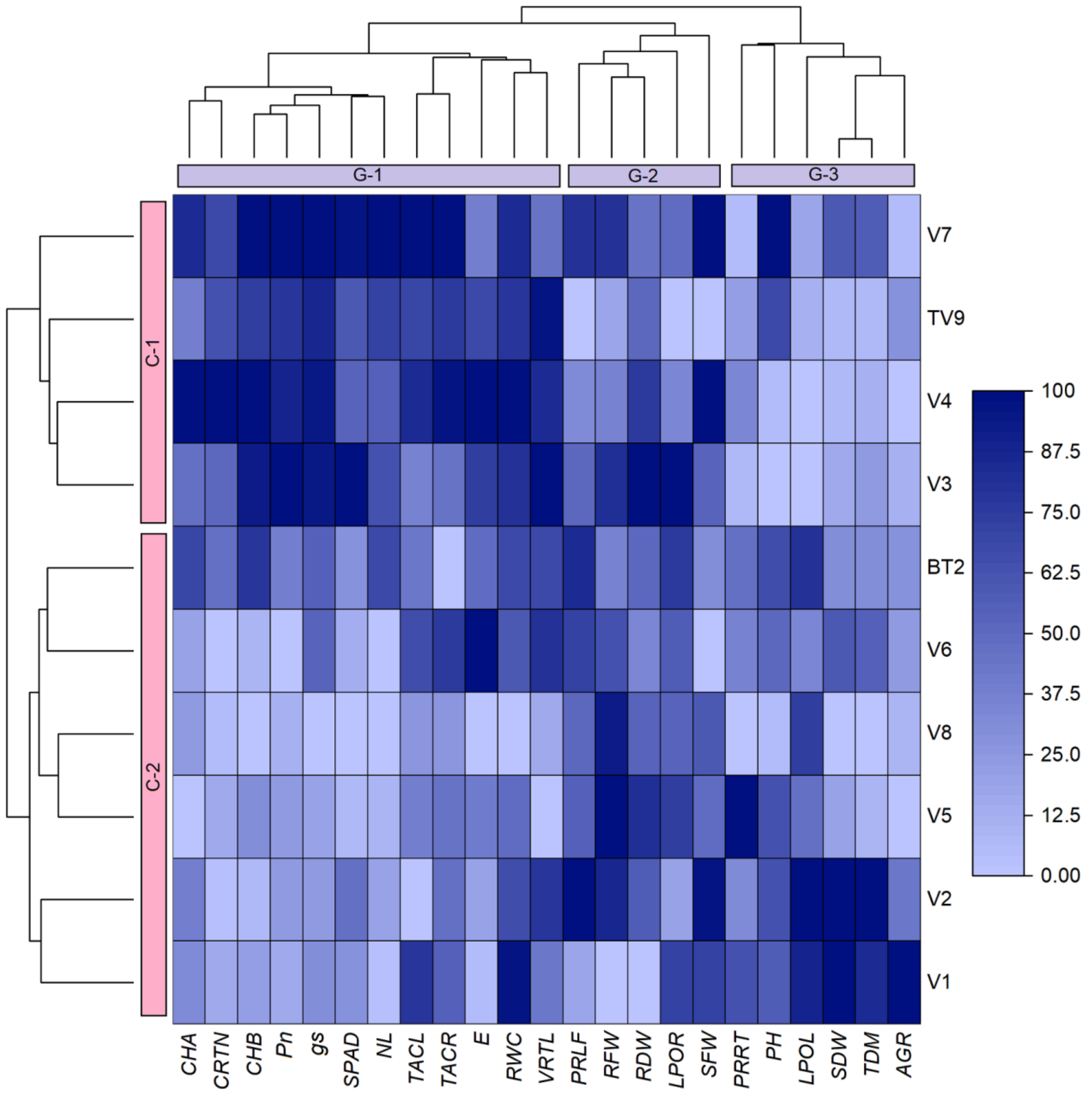

Fig 10. The hierarchical clustering heatmap (two-way) with dendrogram showing the clustering of 23 measured traits and 10 genotypes under the waterlogging phase. The right and left sides of the heatmap illustrate the clusters of the genotypes (C-1 and C-2), while the upper and bottom sides of the heatmap provide the groups of the measured traits (G-1, G-2, and G-3). The performance is demonstrated by a normalized value on a scale of 0 to 100, with a color gradient from light blue to deep blue. PH: Plant height (cm), SFW: Shoot fresh weight (g), SDW: Shoot dry weight (g), RFW: Root fresh weight (g), RDW: Root dry weight (g), TDM: Total dry matter

(g), VRTL: Vertical root length (cm), NL: Number of leaves per plant, *Pn*: Net photosynthesis (µmol CO_2 m^{-2} s^{-1}), *E*: Transpiration rate (mol H_2O m^{-2} s^{-1}), *gs*: Stomatal conductance (mmol m^{-2} s^{-1}), RWC: Percent relative leaf water content, AGR: Absolute growth rate, SPAD: SPAD value of leaves, CHA: Chlorophyll a (mg g^{-1} FW), CHB: Chlorophyll b (mg g^{-1} FW), CRTN: Total carotenoids (mg g^{-1} FW), PRLF: Proline content in leaf (mg g^{-1} FW), PRRT: Proline content in root (mg g^{-1} FW), TACL: Total antioxidant capacity of leaf (mg eq. AA g^{-1} FW), TACR: Total antioxidant capacity of root (mg eq. AA g^{-1} FW), LPOL: Lipid peroxidation of leaf (MDA content nmol g^{-1} FW) and LPOR: Lipid peroxidation of root (MDA content nmol g^{-1} FW).

All the described characters were grouped into three groups at the recovery phase (Fig 11). The highest number (11) of characters were placed into G-1, like CHA, SFW, PH, CHB, *Pn*, NL, VRTL, SPAD, CRTN, RWC, and *gs*. G-2 consisted of only two physiological traits: *E* and AGR, whereas the remaining ten characters were allocated in the G-3 group (Fig 10). The genotypes were also classified into two clusters (C-1 and C-2) at the recovery phase. C-1 consisted of the genotypes V7, V4 and V3, while remaining seven genotypes (TV9, V6, BT2, V8, V5, V2, V1) were placed in C-2.

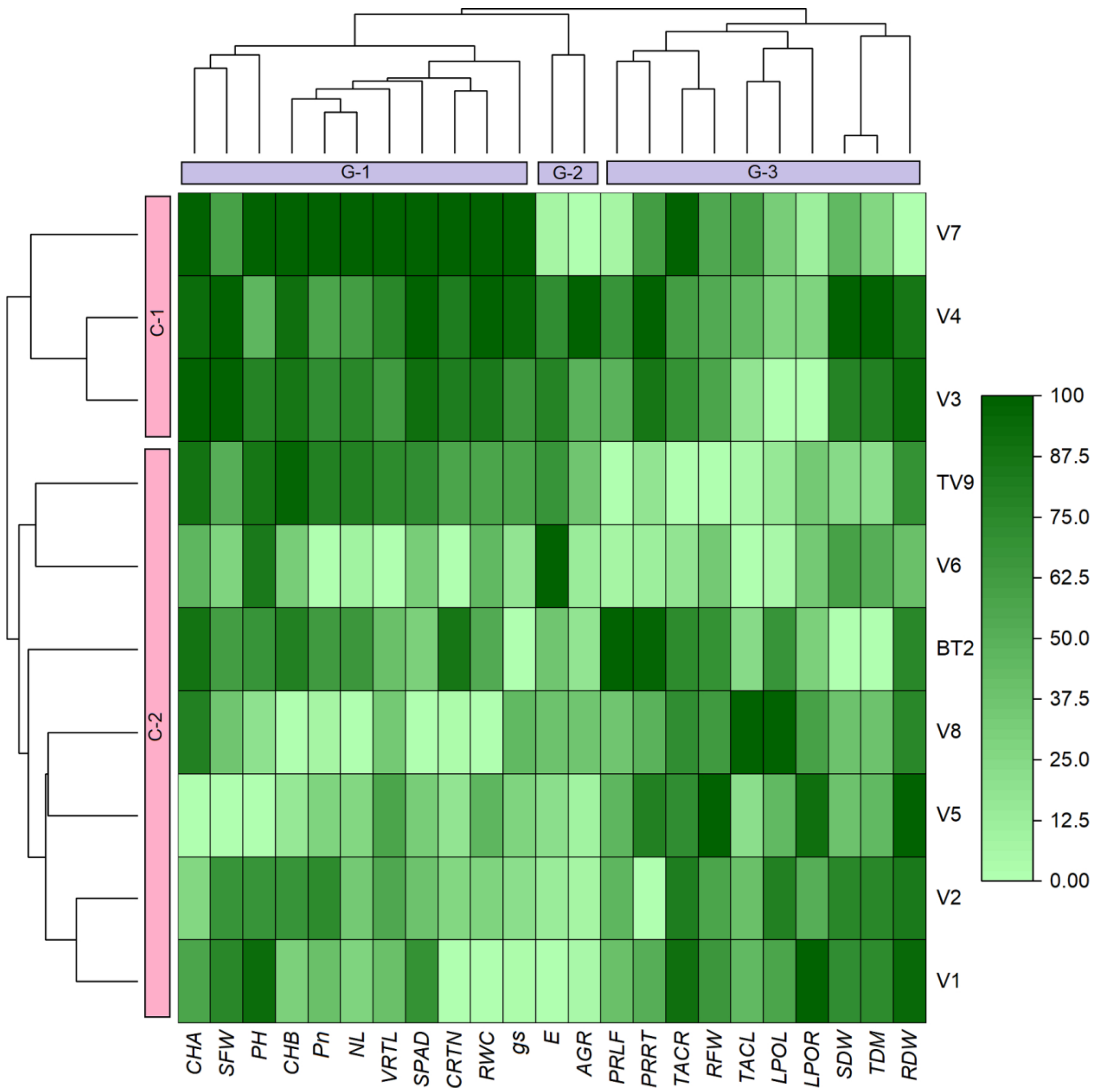

Fig 11. The hierarchical clustering heatmap (two-way) with dendrogram showing the clustering of 23 measured traits and 10 genotypes under the recovery phase. The right and left sides of the heatmap illustrate the clusters of the genotypes (C-1 and C-2), while the upper and bottom sides of the heatmap provide the groups of the measured traits (G-1, G-2, and G-3). The performance is represented by a normalized value on a scale of 0 to 100, with a color gradient from light green to deep green. PH: Plant height (cm), SFW: Shoot fresh weight (g), SDW: Shoot dry

weight (g), RFW: Root fresh weight (g), RDW: Root dry weight (g), TDM: Total dry matter (g), VRTL: Vertical root length (cm), NL: Number of leaves per plant, *Pn*: Net photosynthesis (µmol CO_2 m^{-2} s^{-1}), *E*: Transpiration rate (mol H_2O m^{-2} s^{-1}), *gs*: Stomatal conductance (mmol m^{-2} s^{-1}), RWC: Percent relative leaf water content, AGR: Absolute growth rate, SPAD: SPAD value of leaves, CHA: Chlorophyll a (mg g^{-1} FW), CHB: Chlorophyll b (mg g^{-1} FW), CRTN: Total carotenoids (mg g^{-1} FW), PRLF: Proline content in leaf (mg g^{-1} FW), PRRT: Proline content in root (mg g^{-1} FW), TACL: Total antioxidant capacity of leaf (mg eq. AA g^{-1} FW), TACR: Total antioxidant capacity of root (mg eq. AA g^{-1} FW), LPOL: Lipid peroxidation of leaf (MDA content nmol g^{-1} FW) and LPOR: Lipid peroxidation of root (MDA content nmol g^{-1} FW).

Discussion

~~Tolerance assessment strategies for waterlogging~~

~~Numerous experiments were carried out with different objectives, such as assessing the effects of waterlogging [45], elucidating the adaptive mechanisms [46], and identifying tolerant genotypes in many crops [47, 48]. All the experiments were conducted either under field conditions or controlled artificial environments to investigate the waterlogging effect. Assessment of waterlogging is more challenging in the field due to its complete dependence on rainfall, fluctuating weather conditions, heterogeneous soils, and other uncontrolled factors. Therefore, to obtain more precise and reproducible results, controlled conditions are recommended, and plastic pots are now commonly used in most waterlogging experiments. Artificial~~

waterlogging has been induced by using various plastic pots in *Prunus* species [49], polyvinylchloride pots in cotton [50], plastic tubs in mango [51], double pots in *Phoebe sheareri seedlings* [52], pots covered with polyethylene sheets in sunflower [53], etc. In the current study, numerous plastic boxes (length: 45.72 cm, width: 30.48 cm, and depth: 30.48 cm) were used, each covered with polyethylene sheets to ensure a well-sealed and consistent waterlogged situation for tea saplings.

A waterlogging-induced stress condition, coupled with the post waterlogging recovery phase, provides a comprehensive approach to understanding a plant's resilience and adaptive capabilities during waterlogging. Under an excess moisture situation, plants trigger their tolerance mechanisms to withstand the stress, but in recovery phase, they focus on restoring their original normal state once the stress is alleviated [54]. That's why assessing recovery performance after the stress period is vital to elucidate the sustainability of the tolerance response. Recovery performance studies of different periods after eliminating the waterlogging stress were also observed in different crops, such asbarley [55], coffee [56], avocado [57], banana [58], *Paeonia lactiflora* [59], peanut [60], etc. Tea plants suffer from waterlogging primarily by flooding, which can last for a few hours to several weeks [61]. Tea plants exhibit an immediate response to waterlogging morphologically, physiologically, and biochemically, but the recovery response varies with the genotype, and the effects generally last for a longer period [27]. In the present experiment, tea saplings were kept under both waterlogging and recovery phases to evaluate the responses of genotypes to each trait and to screen the tolerant genotype(s) effectively.

Morphological changes during waterlogging stress

Plants undergo various morphological changes under waterlogged conditions. In the current investigation, the interaction effects (genotype × treatment) of the morphological traits (PH, SFW, SDW, RFW, and TDM) were significant, except for RDW, VRTL, and NL. Interestingly, the genotypic and treatment effects were significant for RDW, VRTL, and NL (S1 Table). Generally, the effects of waterlogging start in the root zone, including limited root growth, varying root length, root discoloration and decay, formation of aerenchyma tissue, development of adventitious roots, anaerobic respiration due to limited oxygen, accumulation of hormones and osmoprotectants, etc. [62, 63]. An insignificant interaction effect between genotypes and treatments on root dry weight (RDW) was observed in soybean under waterlogging stress [64], as well as many other crops [65]. This insignificant effect could be the result of simultaneous changes in waterlogged roots, including root decay, varying root length, and the formation of adventitious roots. Waterlogging also affects the length and plasticity of the root in many crops [66, 67]. In the current study, V4 and V7 showed less reduction in vertical root length during the waterlogging and recovery phases, respectively, compared to the control condition (Figs 3G, 10, and 11). Roots provide nutrients, minerals, etc. to the whole plant, and studies about root systems are very important during waterlogging stress because changes in root morphology have a direct impact on shoot growth parameters, *i.e.,* plant height, biomass accumulation, leaf area, leaf number, etc. [68]. In this investigation, the minimum reduction in plant height (PH) was observed in the V7 genotype during Waterlog-14 days and Recovery-14 days relative to the control (Figs 3A, 10, and 11). A similar trend of decreasing plant height was also observed in many studies [69]. Waterlogging hampers nutrient absorption by the roots; hence, plants suffer from inadequate photosynthates, which disrupts cell

growth, fresh weights, and dry matter accumulation [70]. In the current study, SFW, SDW, RFW, RDW, and TDM decreased with increasing stress treatment duration (Figs 2, 3B-F, 10, and 11). In the case of total dry matter (TDM), V2 and V4 showed lower reductions in waterlogging and recovery phase, respectively (Figs 3F, 10, and 11), and this declining trend in TDM was consistent with many studies [71]. Leaf pigment degradation, reduced physiological activities, and a decline in the leaf number were observed under waterlogging stress [72]. Maximum leaves were observed in TV9 throughout this experiment, but V7 exhibited the lowest reduction [S8 Fig, Figs 3H, 10, and 11], and decreased leaf number was observed in other studies [26].

Fluctuations in physiological traits during waterlogging stress

The physiological parameters (*Pn*, *E*, *gs*, RWC, AGR, and SPAD) had a significant effect throughout the experiment (S1 Table) and were lower in the stress treatment (T_2) than in the control (T_1) (Fig 2). The highest net photosynthesis (*Pn*) was noticed in V1, followed by V7 (S9 Fig), but the least reduction was detected in V7 (waterlogging and recovery phase) (Figs 4A, 10, and 11). V7 also showed less reduction for stomatal conductance (*gs*) (Figs 4C, 10, and 11). Transpiration rate (*E*) declined during stress in all genotypes (S10 Fig), but the decline was low in V6 at both the waterlogging and recovery phases (Figs 4B, 10, and 11). Waterlogging stress impairs root respiration due to oxygen deficiency, thereby hampering nutrient uptake and water transport [73]. This situation stimulates stomatal closure (lower

stomatal conductance) to restrict water content, which in turn prevents the movement of carbon dioxide (CO_2). This limited CO_2 lowers the photosynthetic activity and the transpiration as well [74]. Reduced net photosynthesis, transpiration rate, and stomatal conductance were also noticed in different experiments under waterlogging stress in tea [13, 24, 30]. Waterlogging- tolerant tea cultivar SNT-10 exhibited the least reductions in *Pn*, *E,* and *gs* by 53.6%, 43.8%, and 57.9%, respectively under prolonged waterlogging compared to the other cultivars [13]. Excess moisture stress damages plant roots, and a damaged root system hampers water movement throughout whole plant. This situation creates a water imbalance and reduces cell expansion, which ultimately lowers relative water content (RWC) [75]. The lowest degree of reduction was noticed in V4 (at waterlogging phase) and V7 (at recovery phase) (Figs 4D, 10 and 11) which was consistent with previous many findings [59]. Although the absolute growth rate (AGR) had an insignificant effect on genotypes, but the treatment and interaction effects were significant, indicating that AGR varied significantly across treatments (T_1 and T_2) (S1 Table). The highest AGR was observed in V7 (S12 Fig), but the minimum decline was observed in V1 (at waterlogging phase) and V4 (at recovery phase) (Figs 4E, 10, and 11). The growth rate depends on dry matter accumulation, which decreases under waterlogging stress [76]. Leaf greenness (SPAD indices) was also reduced in the current study (Fig 2), but the minimum decrease was detected in V3 (at the waterlogging phase) and V7 (at the recovery phase) (Figs 4F, 10, and 11). A similar trend was also reported in many studies, and the decreased SPAD value was found due to oxidative stress damages of chloroplast under stress condition [75].

Waterlogging stress and alterations of biochemical parameters

Waterlogging causes numerous biochemical changes in plants. In the current investigation, all biochemical parameters varied significantly among genotypes and treatments (S1 Table). Photosynthetic pigments like chlorophyll a (CHA), chlorophyll b (CHB), total carotenoids (CRTN) were found to be decreased while other parameters, such as proline content in leaf (PRLF) and root (PRRT), total antioxidant capacity of leaf (TACL) and root (TACR), lipid peroxidation in leaf (LPOL) and root (LPOR) were found to be increased when tea plants were subjected to waterlogging stress (Fig 2). Chlorophylls are essential pigments that facilitate photosynthesis by utilizing atmospheric CO_2 and water molecules to convert them into glucose and O_2 in the presence of sunlight [77]. Chlorophyll a and b are the major photosynthetic pigments, where chlorophyll a is responsible for the majority of photosynthesis and chlorophyll b acts as a supporting pigment [78]. Carotenoids are another important group of leaf pigments that protect plants from oxidative stress caused by ROS, such as l superoxide [79]. In this experiment, CHA declined minimally in V4 (at the waterlogging phase) and V3 (at the recovery phase) (Figs 5A, 10, and 11), whereas V7 showed the slightest decrease in CHB (Figs 5B, 10, and 11). The highest amount of CRTN was found in V7 (S17 Fig) but the minimum decrease was observed in V4 (at waterlogging phase) and V7 (at recovery phase) (Figs 5C, 10, and 11). A Decline in leaf pigments under waterlogging has also been reported in many studies, including tea [24, 30, 80, 81]. An Indian tea cultivar, SNT-10, demonstrated superior waterlogging tolerance compared to other cultivars, showing the least decline in CHA (31.4%) and CHB (8.9%) under prolonged and excess soil moisture conditions [13].

Proline, an essential amino acid, acts as an osmolyte that increases in response to waterlogging stress to maintain water balance in plants [82]. Tolerant plants synthesize more proline as a defense mechanism to neutralize the ROS. In this study, V7 accumulated the highest proline content in leaf (at waterlogging phase) (S18 Fig), while V4 synthesized the maximum in root (at recovery phase) (S19 Fig). These findings were similar to those of other studies under waterlogging stress [24, 83]. The highest proline accumulation (3.78 µmol g^{-1} FW) was recorded among 29 tea cultivars after 45 days of prolonged waterlogging [30]. Antioxidants play vital roles in counteracting oxidative stress caused by ROS and free radicals under excess moisture stress [84]. In the current investigation, V7 showed the greatest percentage increase in total antioxidant capacity under waterlogging compared to the control, both in leaf and roots (Figs 5F, 5G, 10, and 11). Higher total antioxidant activity in tolerant species was also noticed in different stress conditions in various experiments [36, 40, 83]. Under stress condition, cell membrane degrades disrupting cellular integrity and making the cell more permeable, thereby unbalancing water-nutrient transport [85]. The level of malondialdehyde (MDA) increases in waterlogging stress, a key indicator of cellular damage, and the genotype with the least increase of MDA appears to be more tolerant [46, 85]. The least increase of MDA in leaf (LPOL) was observed in V3 (Figs 5H, 10, and 11), but in the case of root (LPOR), MDA was found to be increased minimally in TV9 (at waterlogging phase) and V3 (at recovery phase) (Figs 5I, 10, and 11). Significant changes in MDA contents were observed in *Musa acuminata,* ranging from 23 to 25 nmol g^{-1} FW after 14 days of waterlogging phase [86].

Degree of relationship and identification of major traits under waterlogging stress

The types and degrees of relationship among the measured traits were explained by PC analysis (Figs 6 and 7) and a correlation matrix (Figs 8 and 9). PH was positively correlated with SFW and SDW, but negatively correlated with RFW and RDW, reflecting the alternate growth pattern between shoot and root systems, as supported by previous studies [87, 88]. Under waterlogging phase, PH positively associated with PRLF, PRRT, TACL, TACR, LPOL, whereas the relationship became negative during recovery conditions. This trend indicates that the accumulation of proline, antioxidants, and MDA increased under waterlogged conditions but declined upon recovery, while PH continued to increase over time.

Pn, *E,* and *gs* were strongly interrelated, and also positively related with VRTL, NL, RWC, SPAD, CHA, CHB, CRTN, etc. Consistent with earlier studies, waterlogging and other abiotic stresses significantly impair several physiological traits, such as *Pn*, *E*, *gs*, RWC, and SPAD. [13, 24, 30, 89]. Under waterlogged conditions, TDM negatively correlated with *Pn*, *E*, *gs* but showed a positive association during recovery, as reported by others [18, 71]. Under excess moisture conditions, proline synthesis and total antioxidant activity increase remarkably, especially in tolerant tea cultivars and other crops [13, 24, 30, 84]. The accumulation of MDA is also observed under waterlogging, but less accumulation is an important indicator of tolerant genotypes [85, 86]. In the current experiment, PRLF, PRRT, TACL, TACR, LPOL, and

LPOR increased with increasing stress and declined upon recovery, which were correlated with other morpho-physiological parameters.

Principal component analysis (PCA) is a useful statistical tool used in many studies to identify the most important traits or genotypes among the variables, reducing the amplitude of the variables [90, 91]. PC1 and PC2 combinedly contribute 57.81% in the waterlogging phase and 55.42% in the recovery condition (Tables 1 and 2, Figs 6 and 7) of the total variance. During the waterlogging phase, 16 measured parameters out of 23, like PH, SDW, RFW, RDW, TDM, NL, *Pn*, *E*, *gs*, RWC, AGR, SPAD, CHA, CHB, CRTN, and LPOL were responsible for the variation of PC1, and PC2 (S24 and S25 Figs). Again, 18 characters—SFW, RFW, VRTL, NL, *Pn*, *E*, *gs*, RWC, AGR, CHA, CHB, CRTN, PRLF, PRRT, TACL, TACR, LPOL, and LPOR were the main contributing traits in the recovery phase (S26 and S27 Figs). Notably, 11 traits *i.e.,* RFW, NL, *Pn*, *E*, *gs*, RWC, AGR, CHA, CHB, CRTN, and LPOL were in common to both phases, suggesting that these parameters are the most influential indicators of waterlogging response in tea (Fig 12). Similar traits like *Pn*, *E*, *gs,* CHA, and CHB have also been recommended by previous studies for assessing waterlogging tolerance in tea [13, 24, 30].

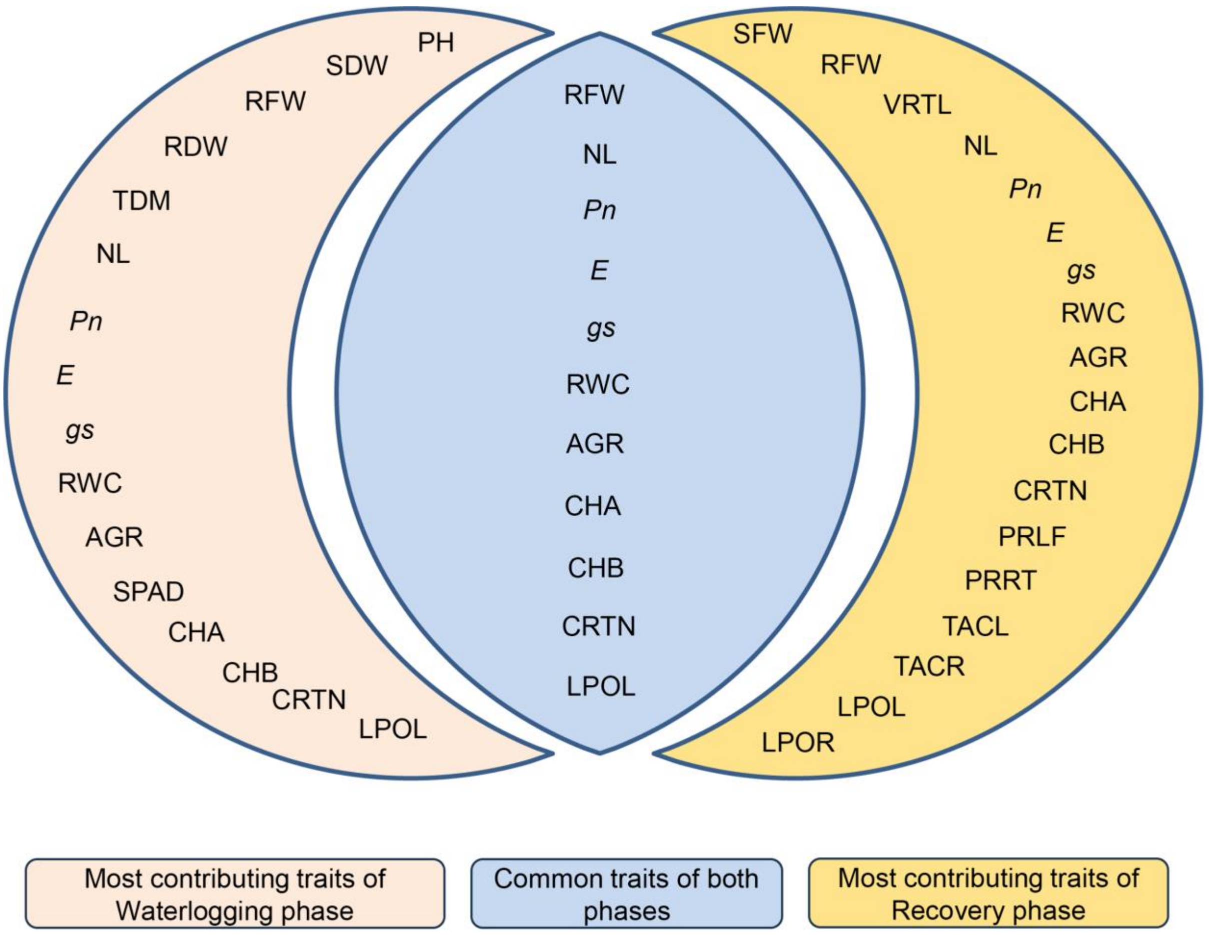

Fig 12. Venn diagram showing the most contributing traits under waterlogging and recovery phases. The left and right fragments of the diagram indicated the traits mostly related to waterlogging and recovery phases, respectively, while the overlapping (middle) fragment in the center represented the common contributors under both phases. PH: Plant height (cm), SFW: Shoot fresh weight (g), SDW: Shoot dry weight (g), RFW: Root fresh weight (g), RDW: Root dry weight (g), TDM: Total dry matter (g), VRTL: Vertical root length (cm), NL: Number of leaves per plant, *Pn*: Net photosynthesis (µmol CO_2 m^{-2} s^{-1}), *E*: Transpiration rate (mol H_2O m^{-2} s^{-1}), *gs*: Stomatal conductance (mmol m^{-2} s^{-1}), RWC: Percent relative leaf water content, AGR: Absolute growth rate, SPAD: SPAD value of leaves, CHA: Chlorophyll a (mg g^{-1} FW), CHB: Chlorophyll b (mg g^{-1} FW), CRTN: Total carotenoids (mg g^{-1} FW), PRLF: Proline content in leaf (mg g^{-1} FW), PRRT: Proline content in root (mg g^{-1} FW), TACL:

Total antioxidant capacity of leaf (mg eq. AA g^{-1} FW), TACR: Total antioxidant capacity of root (mg eq. AA g^{-1} FW), LPOL: Lipid peroxidation of leaf (MDA content nmol g^{-1} FW) and LPOR: Lipid peroxidation of root (MDA content nmol g^{-1} FW).

Screening of tolerant tea genotypes for waterlogging stress

In the present investigation, waterlogging-tolerant tea genotypes were identified using PC analysis (Figs 6 and 7) and hierarchical clustering heatmaps (Figs 10 and 11). PCA helps to identify the promising genotypes by decreasing the magnitudes of the variables [90, 91], whereas a heatmap visualizes the responses of genotypes and groups them accordingly [36, 40, 85, 90]. In the waterlogging phase, the genotypes were grouped into two clusters (C-1 and C-2) (Fig 10). It can be easily noticed that the C-1 cluster which comprised of V7, TV9, V4, and V3 genotypes, exhibited superior responses in waterlogging stress compared to the control for maximum traits (Fig 13). Based on the clustering dendrogram, the performance hierarchy within this cluster was V7 > TV9 > V4 > V3. The same grouping pattern is also observed in PCA-biplot (Fig 6), where V7, TV9, V4, and V3 genotypes were kept on the same right side of the biplot (1st and 4th quadrant). Although V4 and V3 genotypes performed well but they did not surpass the control variety, TV9. On the other hand, the genotypes in C-2 showed a weaker response to waterlogging with a sequence of (BT2 > V6 > V8 > V5 > V2 > V1 which also placed in similar left side (2nd and 3rd quadrants) of PCA-biplot (Fig 6). Therefore, it was observed that only

one genotype (V7) was identified as most superior in the waterlogging phase, whereas V1 was the weakest one.

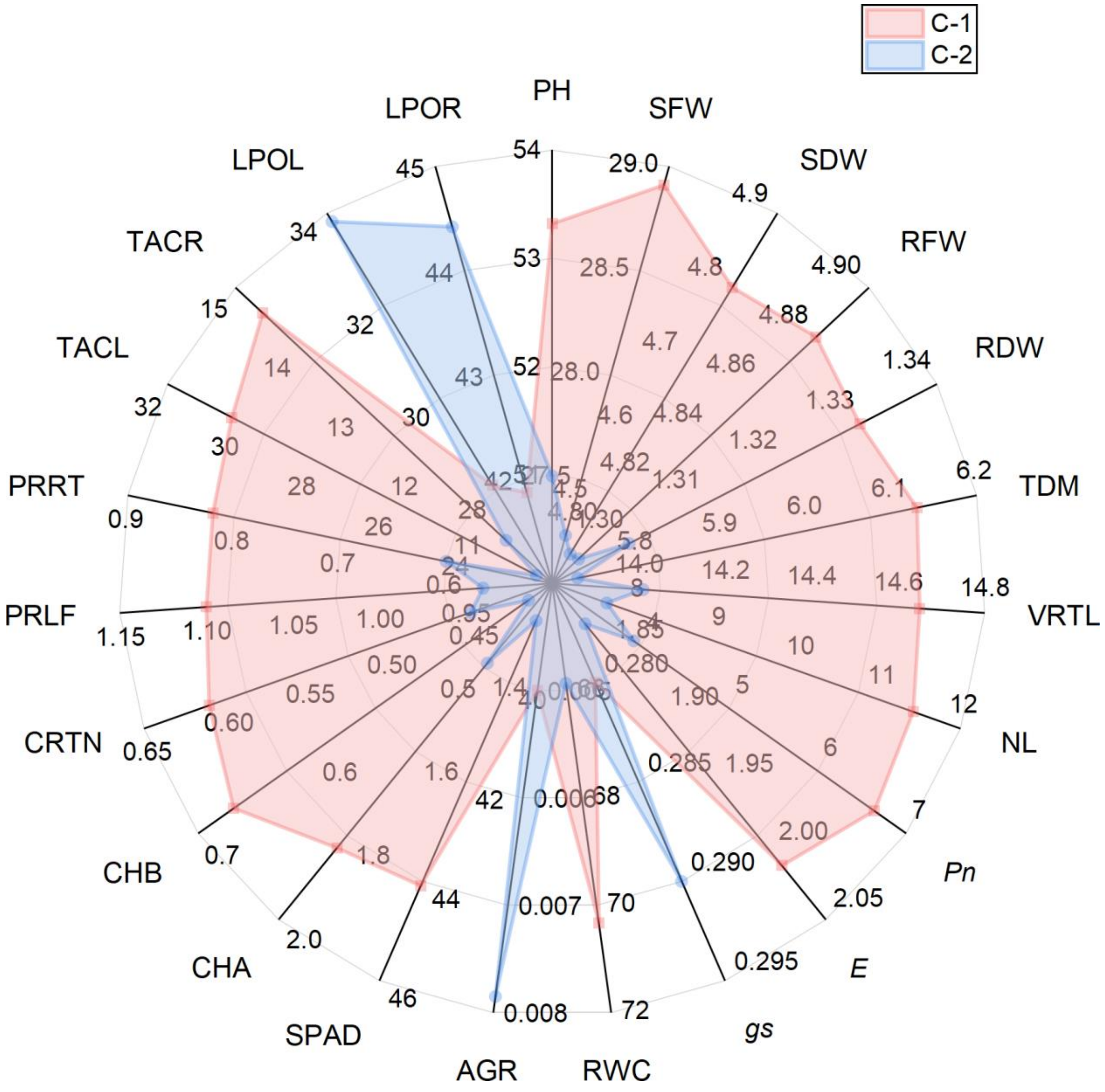

Fig 13. Comparative responses of two different clusters (C-1 and C-2) during the waterlogging phase. The radar plot demonstrated variation in morphological, physiological and biochemical traits, where the pink and blue shaded regions represented the responses of clusters C-1 and C-2, respectively. PH: Plant height (cm), SFW: Shoot fresh weight (g), SDW: Shoot dry weight (g), RFW: Root fresh weight (g), RDW: Root dry weight (g), TDM: Total dry matter (g), VRTL: Vertical root

length (cm), NL: Number of leaves per plant, *Pn*: Net photosynthesis (μmol CO_2 m^{-2} s^{-1}), *E*: Transpiration rate (mol H_2O m^{-2} s^{-1}), *gs*: Stomatal conductance (mmol m^{-2} s^{-1}), RWC: Percent relative leaf water content, AGR: Absolute growth rate, SPAD: SPAD value of leaves, CHA: Chlorophyll a (mg g^{-1} FW), CHB: Chlorophyll b (mg g^{-1} FW), CRTN: Total carotenoids (mg g^{-1} FW), PRLF: Proline content in leaf (mg g^{-1} FW), PRRT: Proline content in root (mg g^{-1} FW), TACL: Total antioxidant capacity of leaf (mg eq. AA g^{-1} FW), TACR: Total antioxidant capacity of root (mg eq. AA g^{-1} FW), LPOL: Lipid peroxidation of leaf (MDA content nmol g^{-1} FW) and LPOR: Lipid peroxidation of root (MDA content nmol g^{-1} FW).

Again, in recovery phase, the genotypes are also categorized in cluster-1 (C-1) and cluster-2 (C-2) (Fig 11). C-1 comprised the genotypes that recovered most effectively (Fig 14), and the order of recovery performance was V7 > V4 > V3, which also placed in a same (1st) quadrant of PCA-biplot (Fig 7). The cluster C-2 consisted of seven genotypes with a sequence of TV9 > V6 > BT2 > V8 > V5 > V2 > V1, which exhibited relatively poor recovery compared to control conditions. Here, interestingly, TV9 which was one of the best performers in waterlogging phase (Fig 10), shifted to C-2 in recovery (Fig 11), suggesting a lower recovery potential. Consistently, V1 remained most susceptible genotype in both waterlogging and recovery phases. Considering the overall responses under both the waterlogging and recovery phases, V7 (P/AFN/13/90) was identified as the most tolerant, demonstrating superior stress endurance and rapid recovery than other genotypes.

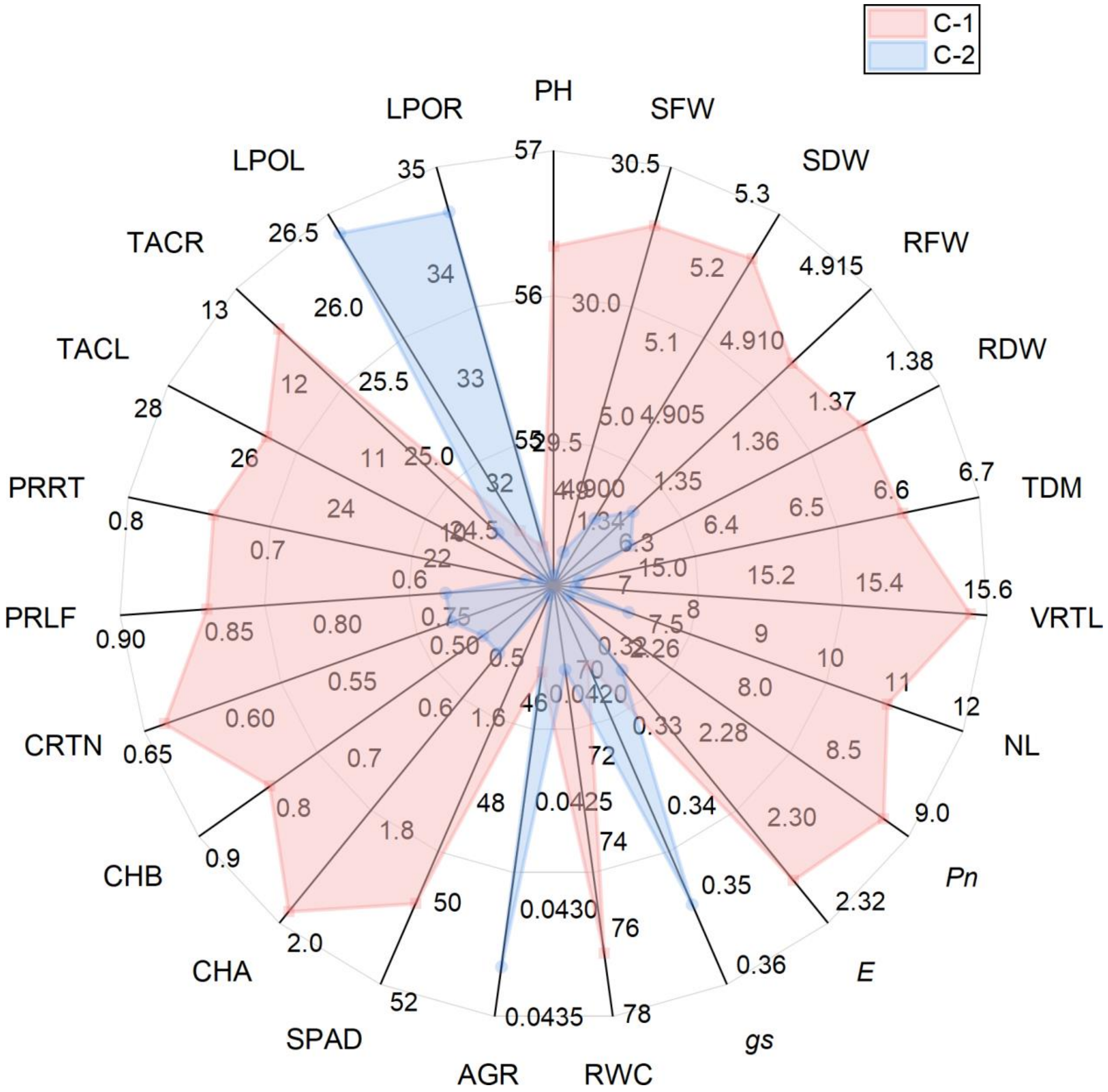

Fig 14. Comparative responses of tea genotype clusters (C-1 and C-2) during the recovery phase. The pink and blue areas indicated the responses of C-1 and C-2, respectively. PH: Plant height (cm), SFW: Shoot fresh weight (g), SDW: Shoot dry weight (g), RFW: Root fresh weight (g), RDW: Root dry weight (g), TDM: Total dry matter (g), VRTL: Vertical root length (cm), NL: Number of leaves per plant, *Pn*: Net photosynthesis (µmol CO_2 m^{-2} s^{-1}), *E*: Transpiration rate (mol H_2O m^{-2} s^{-1}), *gs*: Stomatal conductance (mmol m^{-2} s^{-1}), RWC: Percent relative leaf water content, AGR: Absolute growth rate, SPAD: SPAD value of leaves, CHA: Chlorophyll a (mg

g^{-1} FW), CHB: Chlorophyll b (mg g^{-1} FW), CRTN: Total carotenoids (mg g^{-1} FW), PRLF: Proline content in leaf (mg g^{-1} FW), PRRT: Proline content in root (mg g^{-1} FW), TACL: Total antioxidant capacity of leaf (mg eq. AA g^{-1} FW), TACR: Total antioxidant capacity of root (mg eq. AA g^{-1} FW), LPOL: Lipid peroxidation of leaf (MDA content nmol g^{-1} FW) and LPOR: Lipid peroxidation of root (MDA content nmol g^{-1} FW).

The genotype V7 (P/AFN/13/90) exhibited superior tolerance against waterlogging through coordinated morpho-physiological and biochemical adjustments. It maintained relatively higher and more stable photosynthesis, transpiration, stomatal conductance, and chlorophyll content than other genotypes providing higher energy during stress condition. Increased proline and antioxidant activity helped to maintain osmotic balance and neutralize ROS; meanwhile, lower MDA referred to as higher membrane integrity. Moreover, sustained and higher root growth, relative leaf water content, leaf number with overall growth rate indicated the superior adaptive nature of V7. Collectively these responses of V7 also enabled the rapid recovery after waterlogging stress and a hypothetical tolerance mechanism pathway are demonstrated by Fig 15. This waterlogging tolerance pathway is in line with the findings of previous several studies, which also reported the enhanced photosynthetic efficiency with morphological adjustment and efficient biochemical changes [92, 93].

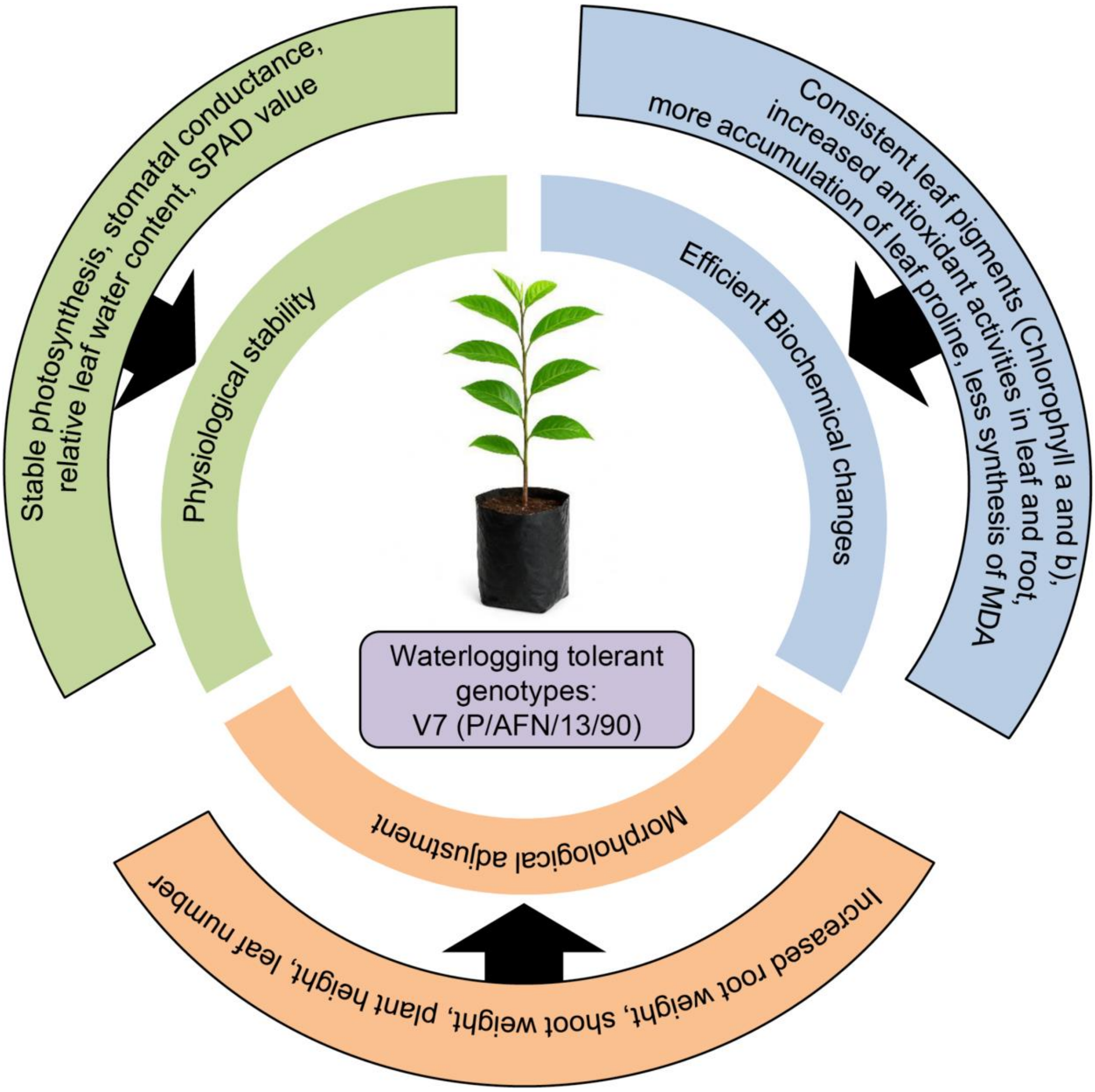

Fig 15. Generalized tolerance pathways of waterlogging tolerant tea genotype V7 (P/AFN/13/90).

Conclusion

The tea plant showed a significant response waterlogging stress, and evaluating tea genotypes for tolerance was more effective when both the waterlogging and

recovery phases were considered together. All the morpho-physiological traits and some biochemical parameters were decreased, while proline content, total antioxidant activity, and lipid peroxidation were increased notably under stress conditions. Among the measured traits, root fresh weight, number of leaves, net photosynthesis, transpiration rate, stomatal conductance, percent relative leaf water content, absolute growth rate, leaf pigments (chlorophyll a, chlorophyll b and total carotenoids), and leaf lipid peroxidation were identified as the most effective stress indicator traits for the assessment of waterlogging tolerance of tea. Finally, only one tea genotype, V7 (P/AFN/13/90) was identified as the most promising and potentially effective against waterlogging stress. However, further genetic characterization and field trial are required to validate the results under variable environmental conditions.

References

1. Pal KK, Islam MA, Uddin MN, Haque MS, Hossain MA. Culm reserves and its remobilization to grain for minimization of grain yield loss in drought-resistant wheat cultivars. Res Crops. 2022;23(1): 26–32. doi: 10.31830/2348-7542.2022.005

2. Donat MG, Lowry AL, Alexander LV, O’Gorman PA, Maher N. More extreme precipitation in the world’s dry and wet regions. Nat Clim Change. 2016;6(5): 508–13. doi: 10.1038/nclimate2941

3. Ahsan N, Lee DG, Lee SH, Lee KW, Bahk JD, Lee BH. A proteomic screen and identification of waterlogging-regulated proteins in tomato roots. Plant Soil. 2007;295(1-2): 37–51. doi: 10.1007/s11104-007-9258-9

4. Shabala S. Physiological and cellular aspects of phytotoxicity tolerance in plants: the role of membrane transporters and implications for crop breeding for waterlogging tolerance. New Phytol. 2011;190(2): 289–98. doi: 10.1111/j.1469-8137.2010.03575.x

5. Kreuzwieser J, Rennenberg H. Molecular and physiological responses of trees to waterlogging stress. Plant Cell Environ. 2014;37(10): 2245–59. doi: 10.1111/pce.12310

6. Sundgren TK, Uhlen AK, Lillemo M, Briese C, Wojciechowski T. Rapid seedling establishment and a narrow root stele promotes waterlogging tolerance in spring wheat. J Plant Physiol. 2018;227: 45–55. doi: 10.1016/j.jplph.2018.04.010

7. Patra A, Singh RP, Singh BK, Kundu MS, Kumar G, Mukherjee S. Waterlogged soils: causes, challenges and management strategies. In: Menon S, Maurya AK, Patra D, Jayswal D, editors. Futuristic Trends in Agriculture Engineering & Food Sciences. Karnataka, India: Iterative International Publishers; 2024. p. 49.58. Available from: https://iipseries.org/assets/docupload/rsl2024C33123AFF12E5DC.pdf

8. Arefin MR, Hossain MI, Rahaman MRU. Determination of green tea recovery percentage and its relation to different weather parameters. Asian Plant Res J. 2020;48–56. doi: 10.9734/aprj/2020/v5i430115

9. Wen B, Ren S, Zhang Y, Duan Y, Shen J, Zhu X, et al. Effects of geographic locations and topographical factors on secondary metabolites distribution in green tea at a regional scale. Food Control. 2020;110: 106979. doi: 10.1016/j.foodcont.2019.106979

10. Hossan MS, Afjal MI, Hasan MF, Hanif MA. Assessment of land dynamics transformation into tea plantations using support vector machine. Trees For People. 2024;18: 100703. doi: 10.1016/j.tfp.2024.100703

11. Zohora KFT, Arefin mr. Tea and tea product diversification: a review. Turk J Agric Food Sci Technol. 2022;10(12): 2334–53. doi: 10.24925/turjaf.v10i12.2334-2353.5280

12. Hossain MS, Uddin R, Barua P, Yasin M, Mamun MSA, Hoque MM. Effects of plant age, topography and processing system on the biochemical traits and quality of tea. Bangladesh J Bot. 2021;50(3): 633–9. doi: 10.3329/bjb.v50i3.55843

13. Borah D, Deka DK, Lahon T, Baruah U, Barman TS. Water logging tolerant clone for cultivation in marginal land. Sci Cult. 2011;77(9-10): 419–24.

14. Ghosal S. Tea production down by more than 10% due to heavy rain, flooding in Assam and West Bengal. The Economic Times. 2020 Jul 20 [Cited 2025 June 1]; Available from: https://economictimes.indiatimes.com/news/economy/agriculture/tea-production-down-by-more-than-10-due-to-heavy-rain-flooding-in-assam-and-west-bengal/articleshow/77069830.cms?from=mdr

15. Mahindapala KGJP, Jayarathna SPAPK. Impact of floods on productivity in tea smallholdings: a case study on lands affected by 2017 floods in Kaluganga River Basin in Ratnapura District of Sri Lanka. Sri Lan J Tea Sci. 2019;84(1-2): 31–43. doi: 10.4038/sljts.v84i1-2.9

16. Raza SMS. Prospects and challenges of tea industry in Bangladesh. Cost Manag. 2019;47(2): 31–5. Available from: http://www.icmab.org.bd/wp-content/uploads/2019/12/5.Prospects.pdf

17. Arefin MR, Haque MS, Sarwar AKMG. Characterization and genetic diversity of tea (*Camellia sinensis* (L.) O. Kuntze) genotypes for waterlogging tolerance. Phyton-Int J Exp Bot. 2024;93(12): 3411–42. doi: 10.32604/phyton.2024.058893

18. Manghwar H, Hussain A, Alam I, Khoso MA, Ali Q, Liu F. Waterlogging stress in plants: unraveling the mechanisms and impacts on growth, development, and productivity. Environ Exp Bot. 2024;224: 105824–4. doi: 10.1016/j.envexpbot.2024.105824

19. Pan J, Sharif R, Xu X, Chen X. Mechanisms of waterlogging tolerance in plants: research progress and prospects. Front Plant Sci. 2021;11. doi: 10.3389/fpls.2020.627331

20. Morales-Olmedo M, Ortiz M, Sellés G. Effects of transient soil waterlogging and its importance for rootstock selection. Chil J Agric Res. 2015;75(Suppl. 1): 45–56. doi: 10.4067/s0718-58392015000300006

21. Zahra N, Hafeez MB, Shaukat K, Wahid A, Hussain S, Naseer R, et al. Hypoxia and anoxia stress: plant responses and tolerance mechanisms. J Agron Crop Sci. 2021;207(2): 249–84. doi: 10.1111/jac.12471

22. Zhang Y, Li Y, Liang T, Yuan Y, Li Z, Xu S, et al. Field-grown cotton shows genotypic variation in agronomic and physiological responses to waterlogging. Field Crops Res. 2023;302: 109067. doi: 10.1016/j.fcr.2023.109067

23. Azrai M, Efendi R, Muliadi A, Aqil M, Suwarti N, Zainuddin B, et al. Genotype by environment interaction on tropical maize hybrids under normal irrigation and waterlogging conditions. Front Sustain Food Syst. 2022;6. doi: 10.3389/fsufs.2022.913211

24. Barman TS, Baruah U, Deka DK, Borah D, Lahon T, Saikia JK. Selection and evaluation of waterlogging tolerant tea genotypes for plantation in marginal land. Two Bud. 2011;58:33–8.

25. Wang X, Deng Z, Zhang W, Meng Z, Chang X, Lv M. Effect of waterlogging duration at different growth stages on the growth, yield and quality of cotton. PLOS ONE. 2017;12(1): e0169029. doi: 10.1371/journal.pone.0169029

26. León-Burgos AF, Unigarro CA, Balaguera-López HE. Soil waterlogging conditions affect growth, water status, and chlorophyll “a” fluorescence in coffee plants (*Coffea arabica* L.). Agronomy. 2022;12(6): 1270. doi: 10.3390/agronomy12061270

27. Medeiros WJF de, Oliveira FÍF de, Lacerda CF de, Sousa CHC de, Cavalcante LF, Silva ARA da, et al. Isolated and combined effects of soil salinity and waterlogging in seedlings of “Green Dwarf” coconut. Semin Ciênc Agrár. 2018;39(4): 1459. doi: 10.5433/1679-0359.2018v39n4p1459

28. Topali C, Antonopoulou C, Chatzissavvidis C. Effect of waterlogging on growth and productivity of fruit crops. Horticulturae. 2024;10(6): 623–3. doi: 10.3390/horticulturae10060623

29. Voesenek LACJ, Colmer TD, Pierik R, Millenaar FF, Peeters AJM. How plants cope with complete submergence. New Phytol. 2006;170(2): 213–26. doi: 10.1111/j.1469-8137.2006.01692.x

30. Deka DK, Borah D, Lahon T, Baruah U, Handique AK, Barman TS. Selection of waterlogging tolerant tea (*Camellia sinensis* L.) genotypes. J Plant Crops. 2011;39(1): 203–9.

31. Jeyaramraja PR, Pius PK, Kumar RR, Jayakumar D. Soil moisture stress-induced alterations in bio constituents determining tea quality. J Sci Food Agric. 2003;83(12): 1187–91. doi: 10.1002/jsfa.1440

32. Das S, Das S, Hazarika M. Breeding of the tea plant (*Camellia sinensis*) in India. In: Chen L, Apostolides Z, Chen ZM, editors. Advanced topics in science and technology in China. China: Zhejiang University Press and Springer; 2012. p. 69–124. doi: 10.1007/978-3-642-31878-8_3

33. Mondal TK. Breeding and biotechnology of tea and its wild species. New Delhi, India: Springer; 2014. doi: 10.1007/978-81-322-1704-6

34. BTRI: Bangladesh Tea Research Institute. Annual report 2023. Bangladesh Tea Research Institute, Sreemangal-3210, Moulvibazar; 2024 p. 80–1. Available from: https://btri.portal.gov.bd/sites/default/files/files/btri.portal.gov.bd/page/faf5cd0b_edd1_4ec4_b5a3_e3aecbc23edb/2024-10-15-03-39-f465d52092e439d1f3f33fb384e85fea.pdf

35. Voyard A, Ciuraru R, Lafouge F, Decuq C, Fortineau A, Loubet B, et al. Emissions of volatile organic compounds from aboveground and belowground parts of rapeseed (*Brassica napus* L.) and tomato (*Solanum lycopersicum* L.). Sci Total Environ. 2024;955: 177081. doi: 10.1016/j.scitotenv.2024.177081

36. Haque MS, Hossain KS, Baroi A, Alamery S, Attia KA, Hafez YM, et al. Foliar application of abscisic acid and glycine betaine induces tolerance to water scarcity in wheat. Plant Growth Regul. 2024;104: 1209–25. doi: 10.1007/s10725-024-01190-6

37. Hunt R. Absolute growth rates. In: Basic growth analysis. Dordrecht: Springer; 1990. p. 17–24. doi: 10.1007/978-94-010-9117-6_2

38. Lichtenthaler HK. Chlorophylls and carotenoids: Pigments of photosynthetic biomembranes. In: Packer L, Douce R, editors. Methods in enzymology. Academic Press; 1987. p. 350–82. doi: 10.1016/0076-6879(87)48036-1

39. Carillo P, Kyriacou MC, El-Nakhel C, Pannico A, dell'Aversana E, D'Amelia L, et al. Sensory and functional quality characterization of protected designation of origin "Piennolo del Vesuvio" cherry tomato landraces from Campania-Italy. Food Chem. 2019;292: 166–75. doi: 10.1016/j.foodchem.2019.04.056

40. Baroi A, Ritu SA, Khan U, Uddin MN, Hossain MA, Haque MS. Abscisic acid and glycine betaine-mediated seed and root priming enhance seedling growth and antioxidative defense in wheat under drought. Heliyon. 2024;10(9): e30598–8. doi: 10.1016/j.heliyon.2024.e30598

41. Prieto P, Pineda M, Aguilar M. Spectrophotometric quantitation of antioxidant capacity through the formation of a phosphomolybdenum complex: specific application to the determination of Vitamin E. Anal Biochem. 1999;269(2): 337–41. doi: 10.1006/abio.1999.4019

42. Heath RL, Packer L. Photoperoxidation in isolated chloroplasts. I. Kinetics and stoichiometry of fatty acid peroxidation. Arch Biochem Biophys. 1968;125(1): 189–98. doi: 10.1016/0003-9861(68)90654-1

43. Fielding JL, Hall JL. A Biochemical and cytochemical study of peroxidase activity in roots of *Pisum sativum*. J Exp Bot. 1978;29(4): 983–91. doi: 10.1093/jxb/29.4.983

44. Sarwar AKMG, Haque MS, Haque ME, Hossain MA, Azam MG, Uddin MN, et al. Agro-morphological characterization and genetic dissection of Linseed (*Linum*

usitatissimum L.) genotypes. Phyton-Int J Exp Bot. 2022;91(7): 1–23. doi: 10.32604/phyton.2022.021069

45. Zhang Y, Chen X, Geng S, Zhang X. A review of soil waterlogging impacts, mechanisms, and adaptive strategies. Front Plant Sci. 2025;16. doi: 10.3389/fpls.2025.1545912

46. Zhang Y, Liu G, Dong H, Li C. Waterlogging stress in cotton: damage, adaptability, alleviation strategies, and mechanisms. Crop J. 2021;9(2): 257–70. doi: 10.1016/j.cj.2020.08.005

47. Ye H, Song L, Chen H, Valliyodan B, Cheng P, Ali L, et al. A major natural genetic variation associated with root system architecture and plasticity improves waterlogging tolerance and yield in soybean. Plant Cell Environ. 2018;41: 2169–82. doi: 10.1111/pce.13190

48. Kuroha T, Nagai K, Gamuyao R, Wang DR, Furuta T, Nakamori M, et al. Ethylene-gibberellin signaling underlies adaptation of rice to periodic flooding. Science. 2018;361(6398): 181–6. doi: 10.1126/science.aat1577

49. Wiström B, Emilsson T, Sjöman H, Levinsson A. Experimental evaluation of waterlogging and drought tolerance of essential *Prunus* species in central Europe. For Ecol Manag. 2023;537: 120904. doi: 10.1016/j.foreco.2023.120904

50. Beegum S, Truong V, Bheemanahalli R, Brand D, Reddy V, Reddy KR. Developing functional relationships between waterlogging and cotton growth and physiology-towards waterlogging modeling. Front Plant Sci. 2023;14: 1174682. doi: 10.3389/fpls.2023.1174682

51. Larson KD, Schaffer B, Davies FS. Mango responses to flooding in limestone soil. Proceedings of the Florida State Horticultural Society. 1991;104: 33–9. Available from: https://journals.flvc.org/fshs/article/download/104742/100634

52. Shi F, Pan Z, Dai P, Shen Y, Lu Y, Han B. Effect of waterlogging stress on leaf anatomical structure and ultrastructure of *Phoebe sheareri* seedlings. Forests. 2023;14(7): 1294–4. doi: 10.3390/f14071294

53. Islam MN, Bell RW, Barrett-Lennard EG, Maniruzzaman M. Growth and yield responses of sunflower to drainage in waterlogged saline soil are caused by changes in plant-water relations and ion concentrations in leaves. Plant Soil. 2022;479(1-2): 679–97. doi: 10.1007/s11104-022-05560-9

54. Tsai KJ, Lin C, Ting CY, Shih MC. Ethylene-regulated glutamate dehydrogenase fine-tunes metabolism during anoxia-reoxygenation. Plant Physiol. 2016;172(3): 1548–62. doi: 10.1104/pp.16.00985

55. Langan P, Cavel E, Henchy J, Bernád V, Ruel P, O'Dea K, et al. Evaluating waterlogging stress response and recovery in barley (*Hordeum vulgare* L.): an image-based phenotyping approach. Plant Methods. 2024;20(1). doi: 10.1186/s13007-024-01256-6

56. Silveira HR de O, Santos M de O, Alves JD, Souza KRD de, Andrade CA, Alves RGM. Growth effects of water excess on coffee seedlings (*Coffea arabica* L.). Acta Sci Agron. 2014;36(2): 211–1. doi: 10.4025/actasciagron.v36i2.17557

57. Lin SY, Chen PA, Zhuang BW. The stomatal conductance and Fv/Fm as the indicators of stress tolerance of avocado seedlings under short-term waterlogging. Agronomy. 2022;12(5): 1084–4. doi: 10.3390/agronomy12051084

58. Lima JD, Passos M, Rozane DE, Modenese H, Gomes EN, Baldinotti S. Recovery of banana seedlings from waterlogging stress with pseudostem residue. Revista Mexicana Re. Mex Cienc Agric. 2024;15(6): e3581–1. doi: 10.29312/remexca.v15i6.3581

59. Zhang Q, Zhang D, Xie A, Xu J, Li X, Li Y, et al. Study on the response and recovery characteristics of different herbaceous peony (*Paeonia lactiflora* Pall.) varieties to waterlogging stress. Am J Plant Sci. 2021;12(09): 1361–79. doi: 10.1016/B978-0-12-394807-6.00083-6

60. Zeng R, Cao J, Li X, Wang X, Wang Y, Yao S, et al. Waterlogging tolerance and recovery capability screening in peanut: a comparative analysis of waterlogging effects on physiological traits and yield. PeerJ. 2022;10: e12741. doi: 10.7717/peerj.12741

61. Yadav LS. Rehabilitation of flood eroded tea land-a field study in North-East India. Int J Innov Res Sci Eng Technol. 2016;5(6): 24–9. Available from: https://www.ijirset.com/upload/2016/nerist/5%20flood2014.pdf

62. Nikolić A, Božić M, Delić N, Marković K, Milivojević M, Čamdžija Z, et al. The early growth of maize under waterlogging stress, as measured by growth, biochemical, and molecular characteristics. Biology. 2025;14(2): 111–1. doi: 10.3390/biology14020111

63. Gowdra VMG, Lalitha BS, Halli HM, Senthamil E, Negi P, Jayadeva HM, et al. Root growth, yield and stress tolerance of soybean to transient waterlogging under different climatic regimes. Sci Rep. 2025;15(1). doi: 10.1038/s41598-025-91780-9

64. Krisnawati A, Nuryati, Adie MM. Agronomic tolerance changes of soybean genotypes under waterlogged condition. In: IOP Conference Series Earth and Environmental Science. IOP Publishing; 2021;648: 012046–6. doi: 10.1088/1755-1315/648/1/012046

65. Yang WC, Lin KH, Wu CW, Chang YJ, Chang YS. Effects of waterlogging with different water resources on plant growth and tolerance capacity of four herbaceous flowers in a Bioretention Basin. Water. 2020;12(6):1619. doi: 10.3390/w12061619

66. Fujita S, Noguchi K, Tange T. Different waterlogging depths affect spatial distribution of fine root growth for *Pinus thunbergii* seedlings. Front Plant Sci. 2021;12. doi: 10.3389/fpls.2021.614764

67. Ostonen I, Püttsepp Ü, Biel C, Alberton O, Bakker MR, Lõhmus K, et al. Specific root length as an indicator of environmental change. Plant Biosyst. 2007;141(3): 426–42. doi: 10.1080/11263500701626069

68. Hayashi T, Yoshida T, Fujii K, Mitsuya S, Tsuji T, Okada Y, et al. Maintained root length density contributes to the waterlogging tolerance in common wheat (*Triticum aestivum* L.). Field Crops Res. 2013;152: 27–35. doi: 10.1016/j.fcr.2013.03.020

69. Jahan MA, Ahmed F. Selection of mungbean genotypes against waterlogging stress. Bangladesh Agron J. 2023;26(1): 40–7. doi: 10.3329/baj.v26i1.69757

70. Zhang Y, Song X, Yang G, Li Z, Lu H, Kong X, et al. Physiological and molecular adjustment of cotton to waterlogging at peak-flowering in relation to growth and yield. Field Crops Res. 2015;179: 164–72. doi: 10.1016/j.fcr.2015.05.001

71. Zeng R, Chen L, Wang X, Cao J, Li X, Xu X, et al. Effect of waterlogging stress on dry matter accumulation, photosynthesis characteristics, yield, and yield

components in three different ecotypes of peanut (*Arachis hypogaea* L.). Agronomy. 2020;10(9): 1244. doi: 10.3390/agronomy10091244

72. Najeeb U, Bange MP, Atwell BJ, Tan DKY. Low incident light combined with partial waterlogging impairs photosynthesis and imposes a yield penalty in cotton. J Agron Crop Sci. 2016;202(4): 331–41. doi: 10.1111/jac.12164

73. Elzenga JTM, van Veen H. Waterlogging and plant nutrient uptake. In: Mancuso S, Shabala S, editors. Waterlogging signaling and tolerance in plants. Berlin, Heidelberg: Springer; 2010. p. 23–35. doi: 10.1007/978-3-642-10305-6_2

74. Else MA, Coupland D, Dutton L, Jackson MB. Decreased root hydraulic conductivity reduces leaf water potential, initiates stomatal closure and slows leaf expansion in flooded plants of castor oil (*Ricinus communis*) despite diminished delivery of ABA from the roots to shoots in xylem sap. Physiol Plant. 2008;111(1): 46–54. doi: 10.1034/j.1399-3054.2001.1110107.x

75. Sathi KS, Masud AAC, Falguni MR, Ahmed N, Rahman K, Hasanuzzaman M. Screening of soybean genotypes for waterlogging stress tolerance and understanding the physiological mechanisms. Adv Agric. 2022; 2022:1–14. doi: 10.1155/2022/5544665

76. Arduini I, Baldanzi M, Pampana S. Reduced growth and nitrogen uptake during waterlogging at tillering permanently affect yield components in late sown oats. Front Plant Sci. 2019;10: 1087. doi: 10.3389/fpls.2019.01087

77. Mandal R, Dutta G. From photosynthesis to biosensing: Chlorophyll proves to be a versatile molecule. Sens Int. 2020;1(1): 100058. doi: 10.1016/j.sintl.2020.100058

78. Tanaka R, Tanaka A. Chlorophyll cycle regulates the construction and destruction of the light-harvesting complexes. Biochim Biophys Acta. 2011;1807(8): 968–76. doi: 10.1016/j.bbabio.2011.01.002

79. Swapnil P, Meena M, Singh SK, Dhuldhaj UP, Harish, Marwal A. Vital roles of carotenoids in plants and humans to deteriorate stress with its structure, biosynthesis, metabolic engineering and functional aspects. Curr Plant Biol. 2021;26: 100203. doi: 10.1016/j.cpb.2021.100203

80. Dethvongsa S, Vu NA, Van TK. Comparison waterlogging tolerance potential of cassava. IOP Conf Ser Earth Environ Sci. 2021;707(1): 012002. doi: 10.1088/1755-1315/707/1/012002

81. Shibly N, Islam R, Hasan M, Bari N, Ahmed J. Evaluation of yield and yield-related traits for waterlogging tolerance in mungbean genotypes using multivariate techniques. J Agric Sci (Belgr). 2020;65(2): 99–120. doi: 10.2298/JAS2002099S

82. Molla MR, Rohman MM, Islam MR, Hasanuzzaman M, Hassan L. Screening and evaluation of chili (*Capsicum annuum* L.) genotypes for waterlogging tolerance at seedling stage. BIOCELL. 2022;46(7): 1613–27. doi: 10.32604/biocell.2022.019243

83. Dalai D, Sardar SS. Tolerance response of sunflower (*Helianthus annuus* L.) cultivar NSSH-1084 to water logging stress. Int J Curr Microbiol Appl Sci. 2021;10(8): 219–33. doi: 10.20546/ijcmas.2021.1008.026

84. Jahan M, Serity SA, Roy TK, Akter K, Rasul S, Depro MJ, et al. Nutrition and Antioxidant potential of three cauliflower (*Brassica oleracea* L. Var. Botrytis) cultivars cultivated in southern part of Bangladesh. Turk J Agric Food Sci Technol. 13(1): 158–68. doi: 10.24925/turjaf.v13i1.158-168.7065

85. Mondal S, Haque MS, Rabbi MF, Tasnim H, Iffat A, Baroi A, et al. Salicylic acid and methyl jasmonate mitigate cadmium toxicity in spinach (*Spinacea oleracea* L.) by modulating metal translocation, growth, photo-physiology and redox regulation. J Plant Nutr Soil Sci. 2025;25(2): 2846. doi: 10.1007/s42729-025-02302-5

86. Teoh EY, Teo CH, Baharum NA, Pua TL, Tan BC. Waterlogging stress induces antioxidant defense responses, aerenchyma formation and alters metabolisms of banana plants. Plants. 2022;11(15): 2052. doi: 10.3390/plants11152052

87. Shabnam R, Iqbal MT. Understanding phosphorus dynamics on wheat plant under split-root system in alkaline soil. Braz J Sci Technol. 2016;3(1). doi: 10.1186/s40552-016-0031-6

88. Chen X, Zhu Y, Ding Y, Pan R, Shen W, Yu X, et al. The relationship between characteristics of root morphology and grain filling in wheat under drought stress. PeerJ . 2021;9: e12015. doi: 10.7717/peerj.12015

89. Rokhmah DN, Astutik D, H Supriadi H. Cultivation technology for drought stress mitigation in tea plants: a review. IOP Conf Ser Earth Environ Sci. 2022;1038(1): 012015. doi: 10.1088/1755-1315/1038/1/012015

90. Mohi-Ud-Din M, Hossain MA, Rohman MM, Uddin MN, Haque MS, Ahmed JU, et al. Multivariate analysis of morpho-physiological traits reveals differential drought tolerance potential of bread wheat genotypes at the seedling stage. Plants. 2021;10(5): 879. doi: 10.3390/plants10050879

91. Brereton R. Principal component analysis: Standardisation. J Chemom. 2025;39(1). doi: 10.1002/cem.3607

92. 94.Arefin MR, Hossain MI, Aziz MA, Haque MS, Sarwar AKMG. Tea (*Camellia sinensis* (L.) O Kuntze) under waterlogging stress: A comprehensive review on adaptation mechanisms and crop improvement opportunities. Plant Sci Today. 2025; doi: https://doi.org/10.14719/pst.8794

93. Nguyen VL, Luu HN, Hong T, Nguyen VL, Chu DH, Bertero D, et al. Genotype by environment interaction across water regimes in relation to cropping season response of quinoa (*Chenopodium quinoa*). PLOS ONE. 2024;19(10): e0309777–7. doi: 10.1371/journal.pone.0309777

List of Supporting data

- S1 Table. Analysis of variances (ANOVA) of 23 measured traits in 10 tea genotypes for waterlogging tolerance.
- S1 Fig. Variations in plant height (cm) of 10 genotypes in different treatments.
- S2 Fig. Variations in shoot fresh weight (g) of 10 genotypes in different treatments.
- S3 Fig. Variations in shoot dry weight (g) of 10 genotypes in different treatments.
- S4 Fig. Variations in root fresh weight (g) of 10 genotypes in different treatments.
- S5 Fig. Variations in root dry weight (g) of 10 genotypes in different treatments.
- S6 Fig. Variations in total dry matter (g) of 10 genotypes in different treatments.
- S7 Fig. Variations in vertical root length (cm) of 10 genotypes in different treatments.
- S8 Fig. Variations in number of leaves per plant of 10 genotypes in different treatments.
- S9 Fig. Variations in net photosynthesis (μmol CO_2 m^{-2} s^{-1}) of 10 genotypes in different treatments.

- S10 Fig. Variations in transpiration rate (mol H_2O m^{-2} s^{-1}) of 10 genotypes in different treatments.
- S11 Fig. Variations in stomatal conductance (mmol m^{-2} s^{-1}) of 10 genotypes in different treatments.
- S12 Fig. Variations in relative leaf water content (%) of 10 genotypes in different treatments.
- S13 Fig. Variations in absolute growth rate of 10 genotypes in different treatments.
- S14 Fig. Variations in SPAD value of leaves of 10 genotypes in different treatments.
- S15 Fig. Variations in chlorophyll a (mg g^{-1} FW) of 10 genotypes in different treatments.
- S16 Fig. Variations in chlorophyll b (mg g^{-1} FW) of 10 genotypes in different treatments.
- S17 Fig. Variations in total carotenoids (mg g^{-1} FW) of 10 genotypes in different treatments.
- S18 Fig. Variations in proline content in leaf (mg g^{-1} FW) of 10 genotypes in different treatments.
- S19 Fig. Variations in proline content in root (mg g^{-1} FW) of 10 genotypes in different treatments.
- S20 Fig. Variations in total antioxidant capacity of leaf (mg eq. AA g^{-1} FW) of 10 genotypes in different treatments.

- S21 Fig. Variations in total antioxidant capacity of root (mg eq. AA g^{-1} FW) of 10 genotypes in different treatments.
- S22 Fig. Variations in lipid peroxidation of leaf (MDA content nmol g^{-1} FW) of 10 genotypes in different treatments.
- S23 Fig. Variations in lipid peroxidation of root (MDA content nmol g^{-1} FW) of 10 genotypes in different treatments.
- S24 Fig. Contribution of the variables to PC1 under waterlogging phase.
- S25 Fig. Contribution of the variables to PC2 under waterlogging phase.
- S26 Fig. Contribution of the variables to PC1 under recovery phase.
- S27 Fig. Contribution of the variables to PC2 under recovery phase.

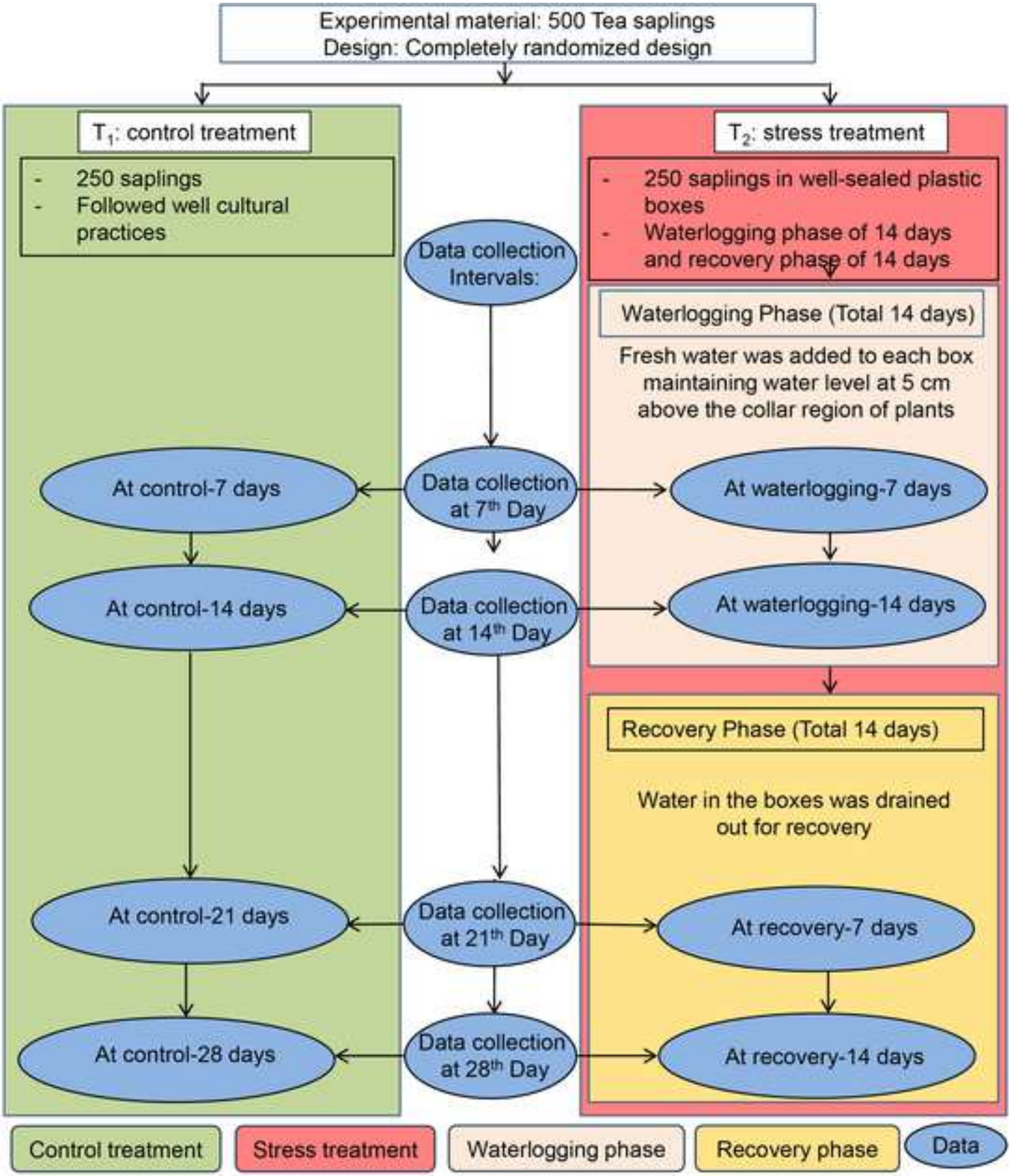
Experimental material: 500 Tea saplings
Design: Completely randomized design
T1: control treatment
- 250 saplings
- Followed well cultural practices
T2: stress treatment
- 250 saplings in well-sealed plastic boxes
- Waterlogging phase of 14 days and recovery phase of 14 days
Data collection Intervals:
Waterlogging Phase (Total 14 days)
Fresh water was added to each box maintaining water level at 5 cm above the collar region of plants
At control-7 days
Data collection at 7th Day
At waterlogging-7 days
At control-14 days
Data collection at 14th Day
At waterlogging-14 days
Recovery Phase (Total 14 days)
Water in the boxes was drained out for recovery
At control-21 days
Data collection at 21th Day
At recovery-7 days
At control-28 days
Data collection at 28th Day
At recovery-14 days
Control treatment
Stress treatment
Waterlogging phase
Recovery phase
Data

Figure2

Click here to access/download;Figure;Fig2.tif

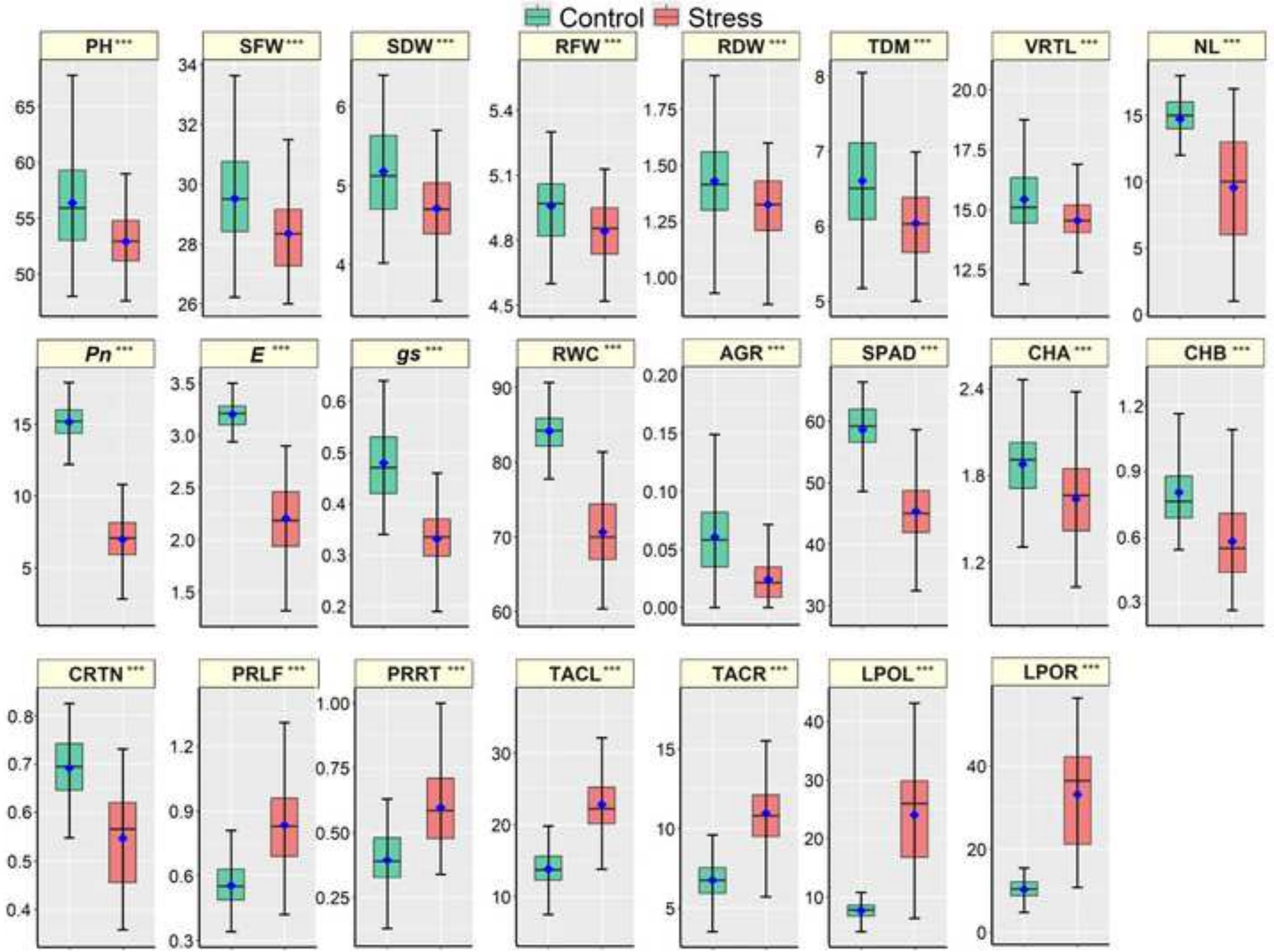

Figure3

Click here to access/download;Figure;Fig3.tif

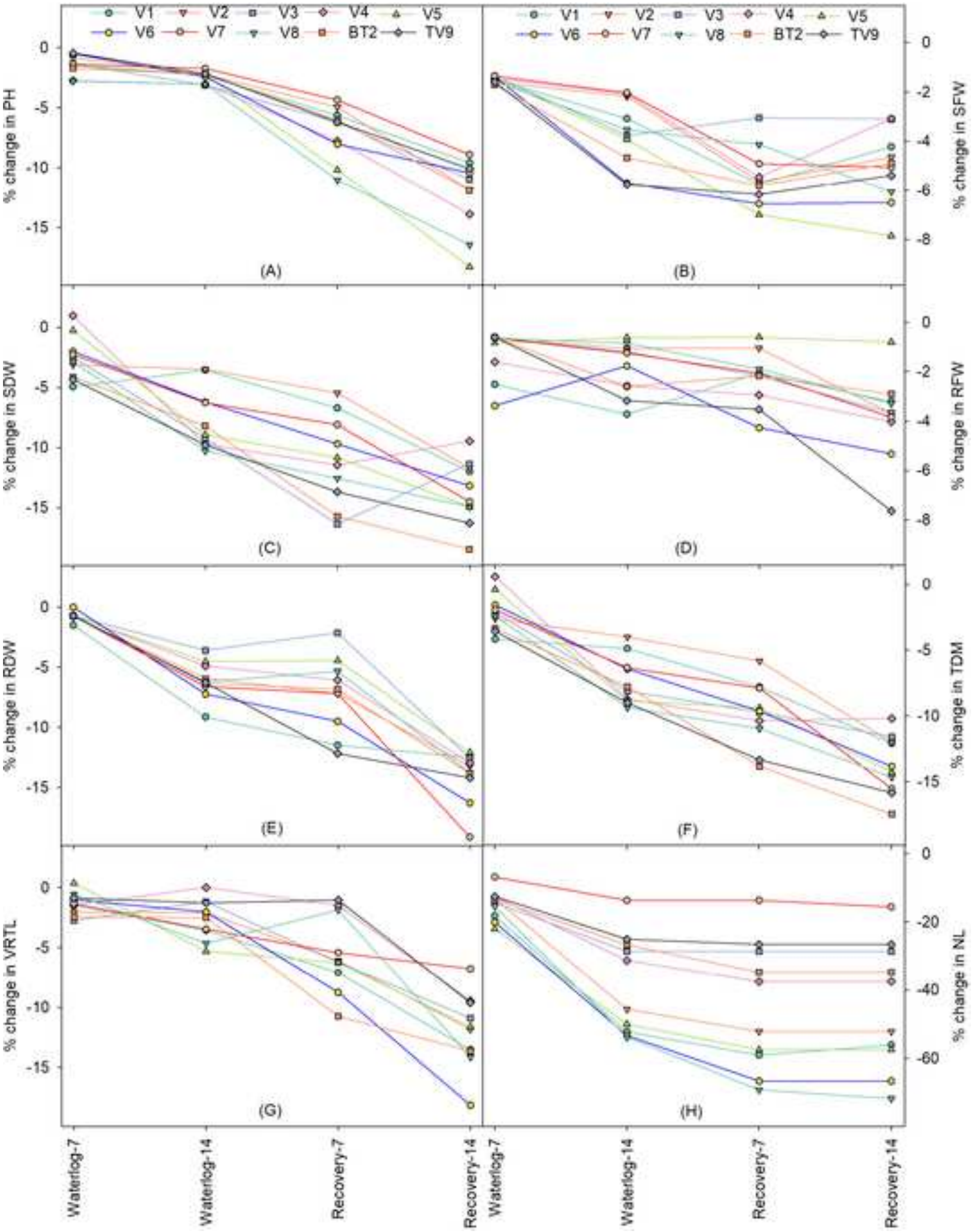

Figure4 Click here to access/download;Figure;Fig4.tif

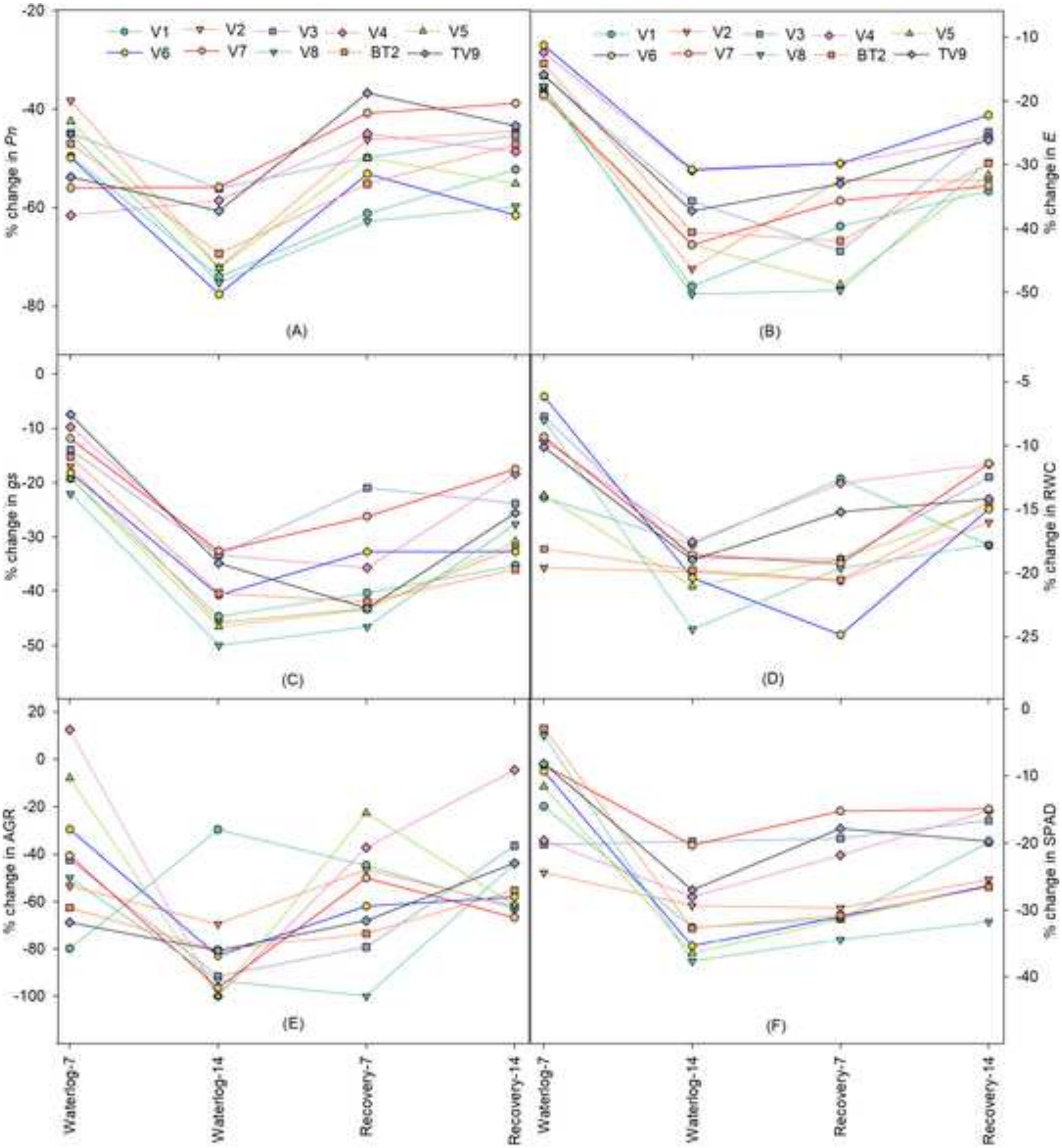

Figure5

Click here to access/download;Figure;Fig5.tif

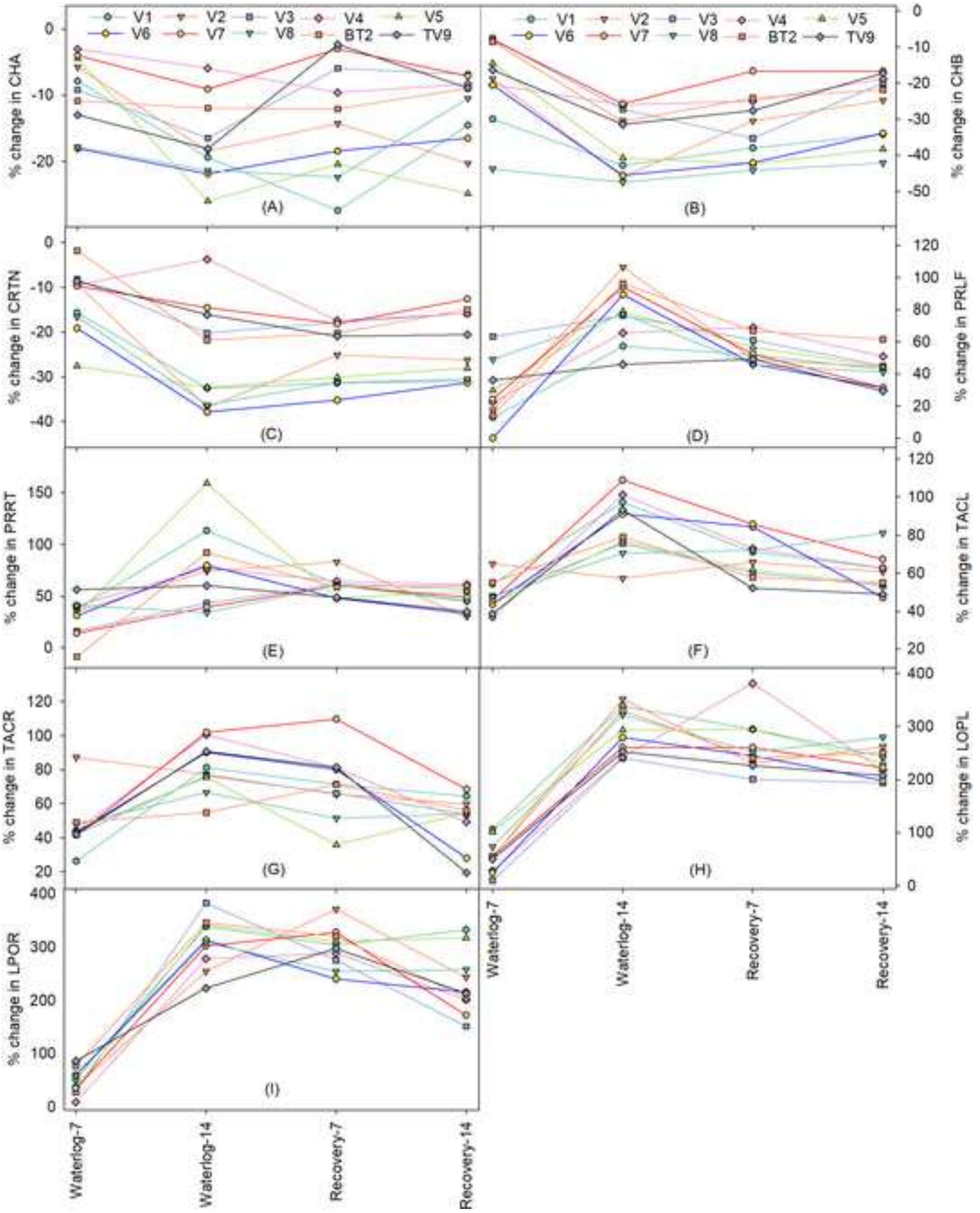

 Click here to access/download;Figure;Fig6.tif

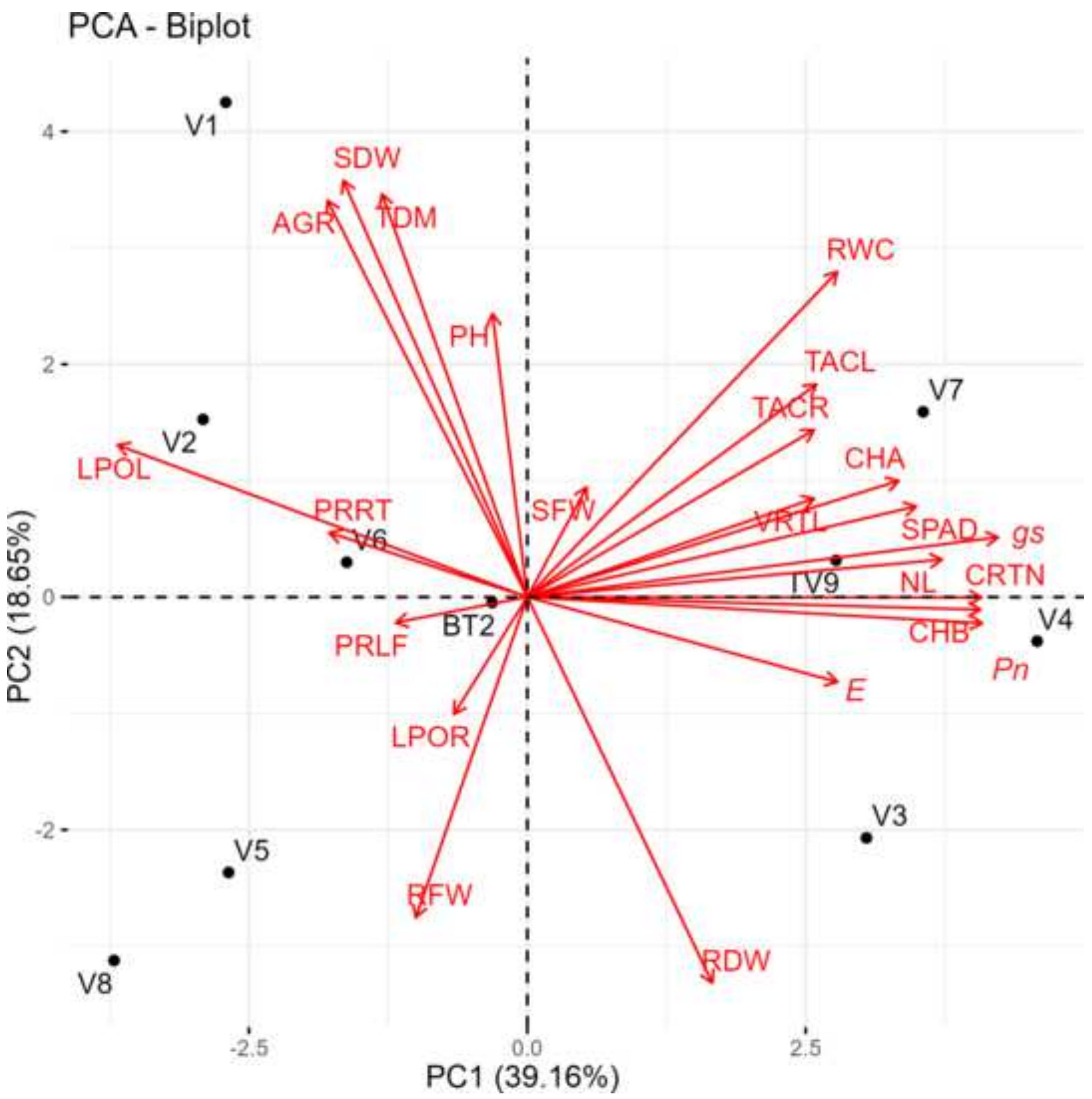

Figure7

Click here to access/download;Figure;Fig7.tif

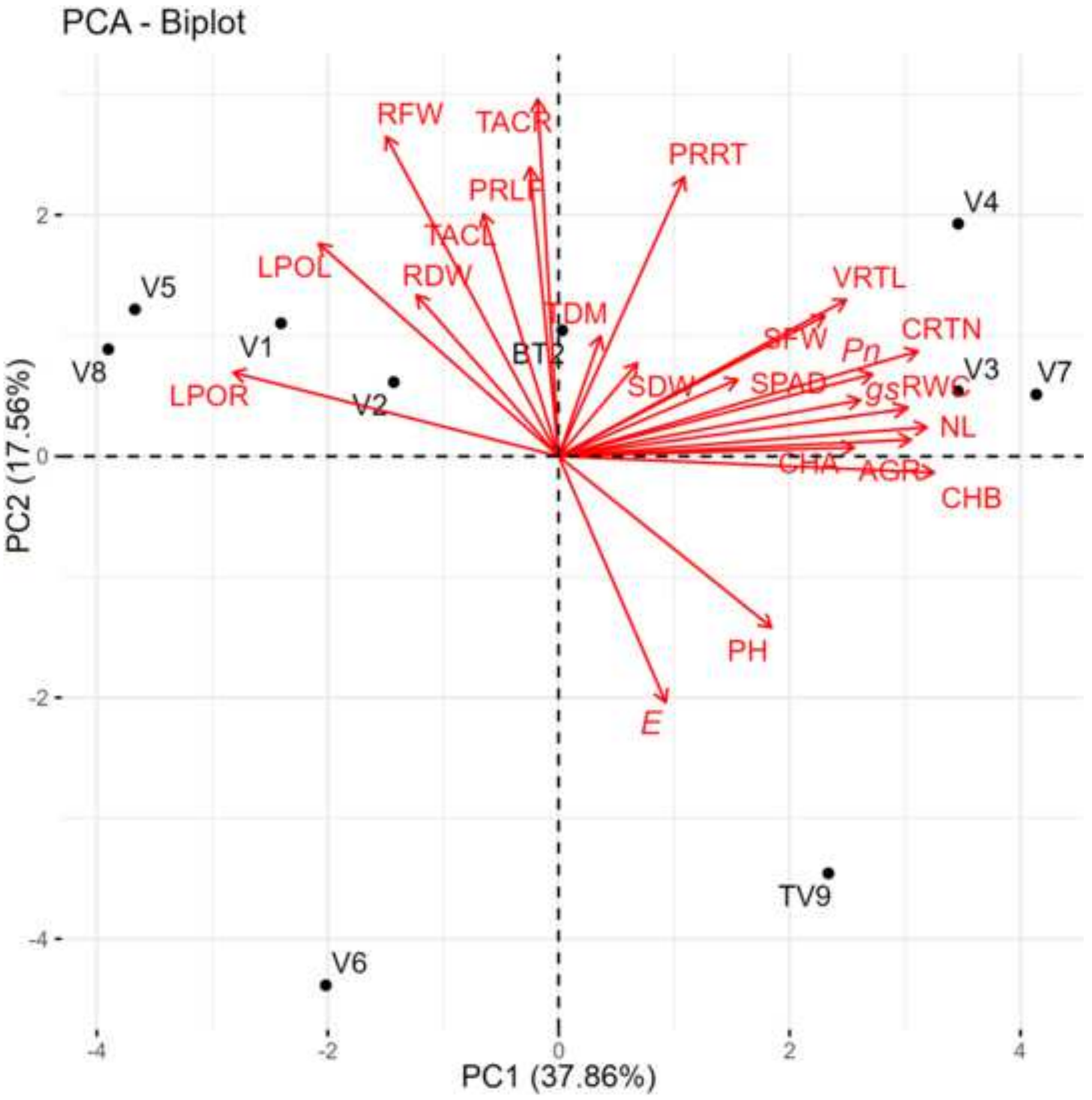

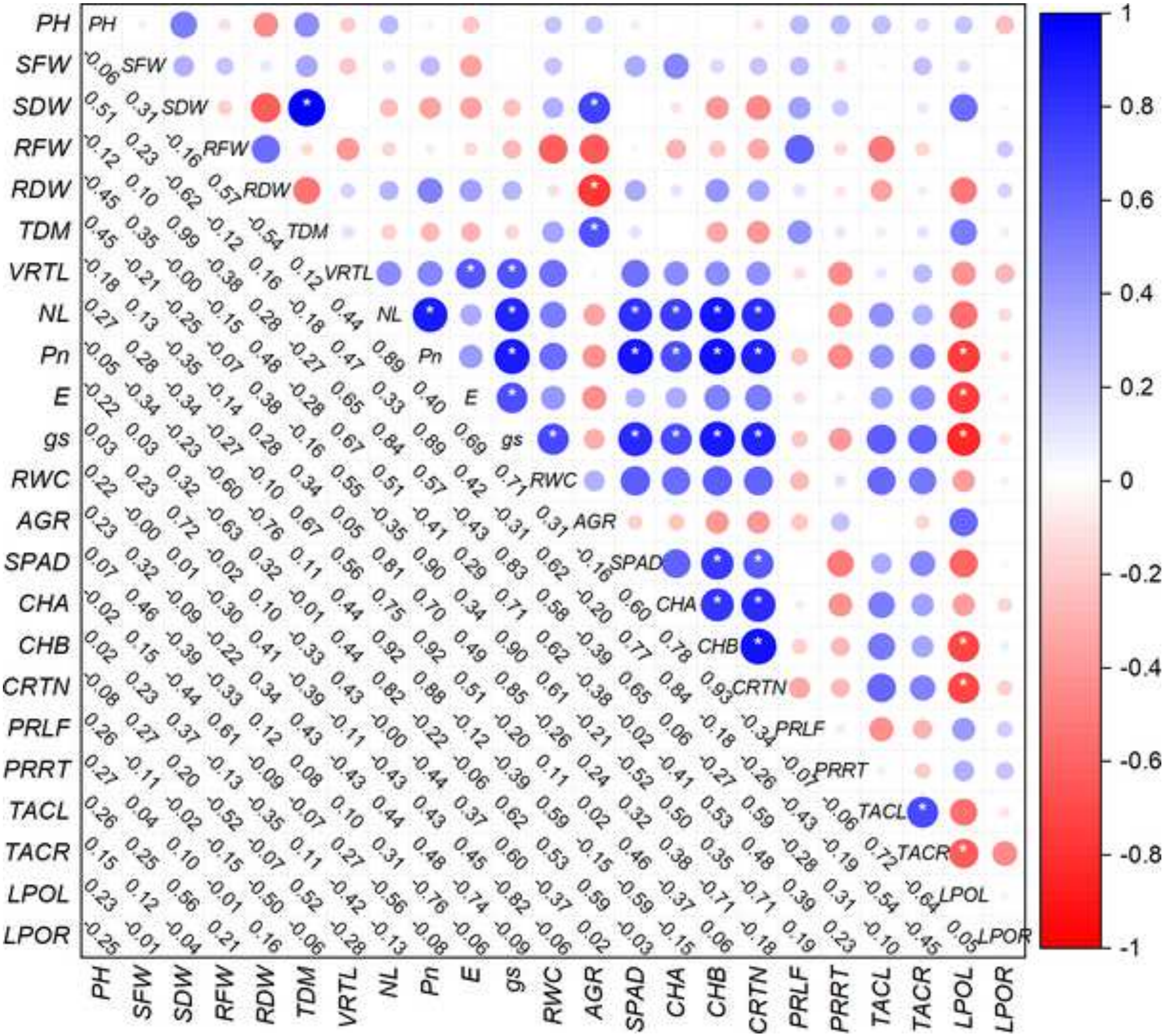
PH
SFW
SDW
RFW
RDW
TDM
VRTL
NL
Pn
E
gs
RWC
AGR
SPAD
CHA
CHB
CRTN
PRLF
PRRT
TACL
TACR
LPOL
LPOR
1
0.8
0.6
0.4
0.2
0
-0.2
-0.4
-0.6
-0.8
-1

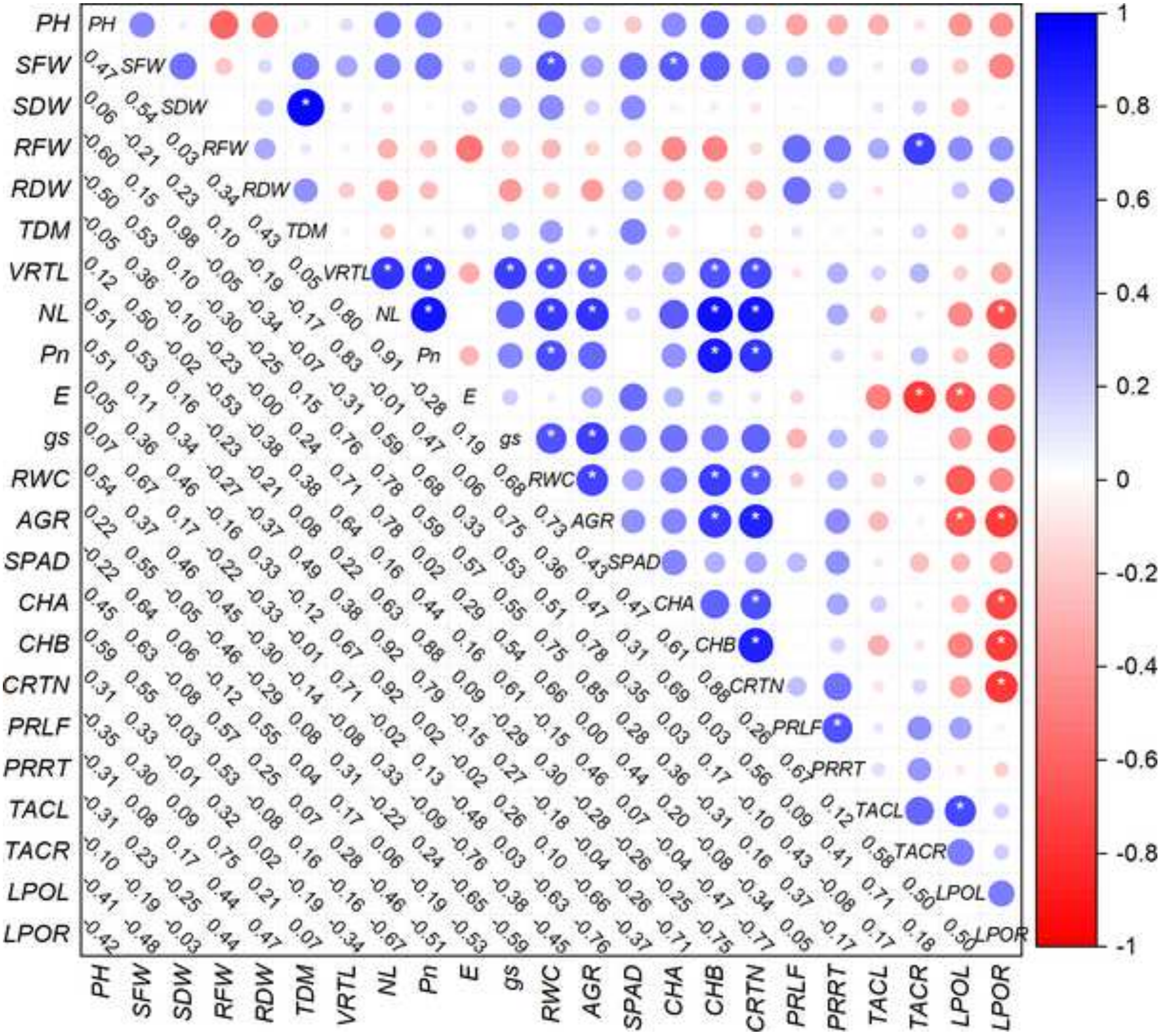
PH SFW SDW RFW RDW TDM VRTL NL Pn E gs RWC AGR SPAD CHA CHB CRTN PRLF PRRT TACL TACR LPOL LPOR
0.47
0.06 0.54
-0.60 -0.21 0.03
-0.50 0.15 0.23 0.34
-0.05 0.53 0.98 0.10 0.43
0.12 0.36 0.10 -0.05 -0.19 0.05
0.51 0.50 -0.10 -0.30 -0.34 -0.17 0.80
0.51 0.53 -0.02 -0.23 -0.25 -0.07 0.83 0.91
0.05 0.11 0.16 -0.53 -0.00 0.15 -0.31 -0.01 -0.28
0.07 0.36 0.34 -0.23 -0.38 0.24 0.76 0.59 0.47 0.19
0.54 0.67 0.46 -0.27 -0.21 0.38 0.71 0.78 0.68 0.06 0.68
0.22 0.37 0.17 -0.16 -0.37 0.08 0.64 0.78 0.59 0.33 0.75 0.73
0.22 0.55 0.46 -0.22 0.33 0.49 0.22 0.16 0.02 0.57 0.53 0.36 0.43
0.45 0.64 -0.05 -0.45 -0.33 -0.12 0.38 0.63 0.44 0.29 0.55 0.51 0.47 0.47
0.59 0.63 0.06 -0.46 -0.30 -0.01 0.67 0.92 0.88 0.16 0.54 0.75 0.78 0.31 0.61
0.31 0.55 -0.08 -0.12 -0.29 -0.14 0.71 0.92 0.79 0.09 0.61 0.66 0.85 0.35 0.69 0.88
-0.35 0.33 -0.03 0.57 0.55 0.08 -0.08 -0.02 0.02 -0.15 -0.29 -0.15 0.00 0.28 0.03 0.03 0.26
-0.31 0.30 -0.01 0.53 0.25 0.04 0.31 0.33 0.13 -0.02 0.27 0.30 0.46 0.44 0.36 0.17 0.56 0.67
-0.31 0.08 0.09 0.32 -0.08 0.07 0.17 -0.22 -0.09 -0.48 0.26 -0.18 -0.28 0.07 0.20 -0.31 -0.10 0.09 0.12
-0.10 0.23 0.17 0.75 0.02 0.16 0.28 0.06 0.24 -0.76 0.03 0.10 -0.04 -0.26 -0.04 -0.08 0.16 0.43 0.41 0.58
-0.41 -0.19 -0.25 0.44 0.21 -0.19 -0.16 -0.46 -0.19 -0.65 -0.38 -0.63 -0.66 -0.26 -0.25 -0.47 -0.34 0.37 -0.08 0.71 0.50
-0.42 -0.48 -0.03 0.44 0.47 0.07 -0.34 -0.67 -0.51 -0.53 -0.59 -0.45 -0.76 -0.37 -0.71 -0.75 -0.77 0.05 -0.17 0.17 0.18 0.50
1 0.8 0.6 0.4 0.2 0 -0.2 -0.4 -0.6 -0.8 -1

Figure10

Click here to access/download;Figure;Fig10.tif

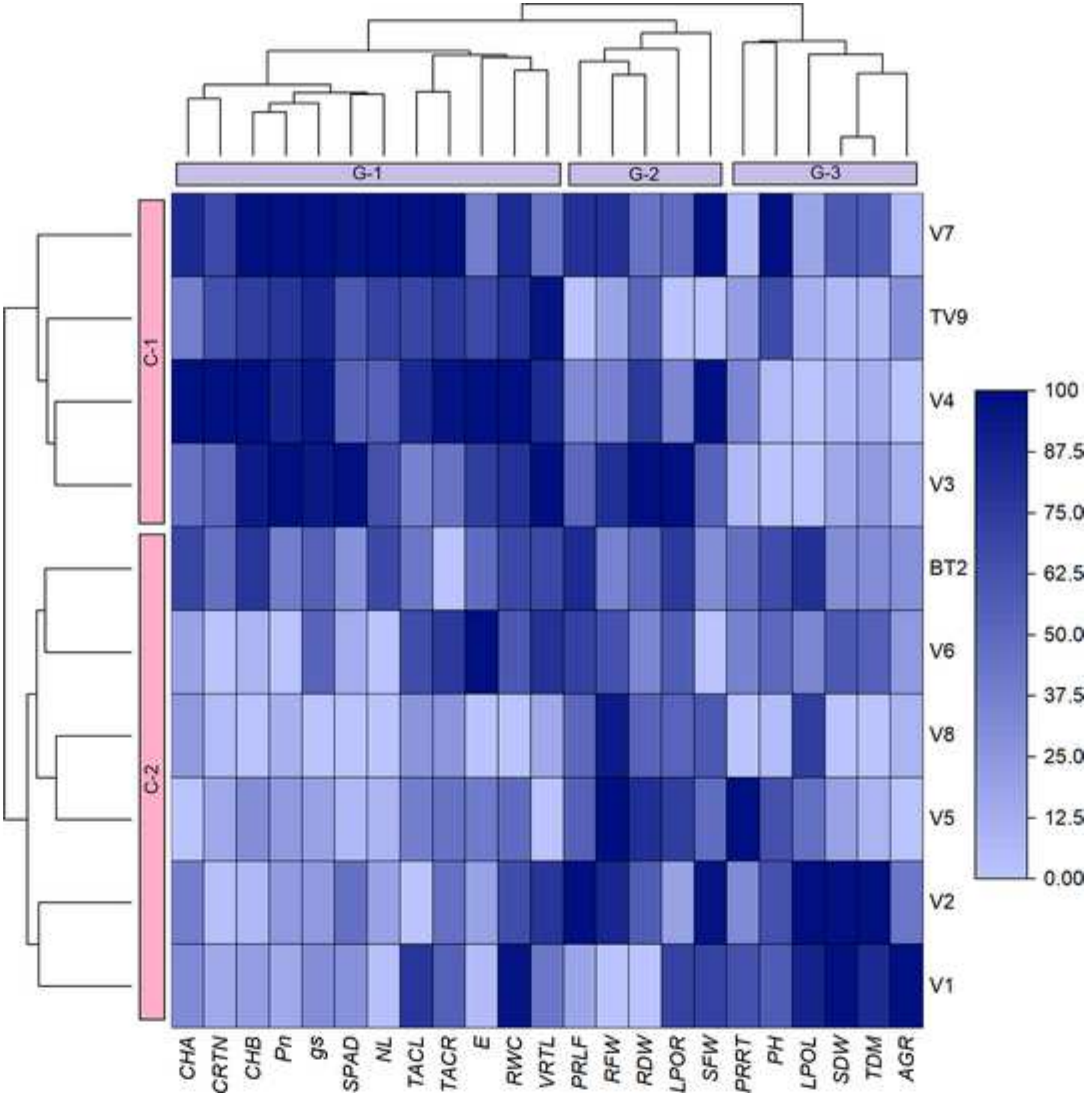

Figure11

Click here to access/download;Figure;Fig11.tif

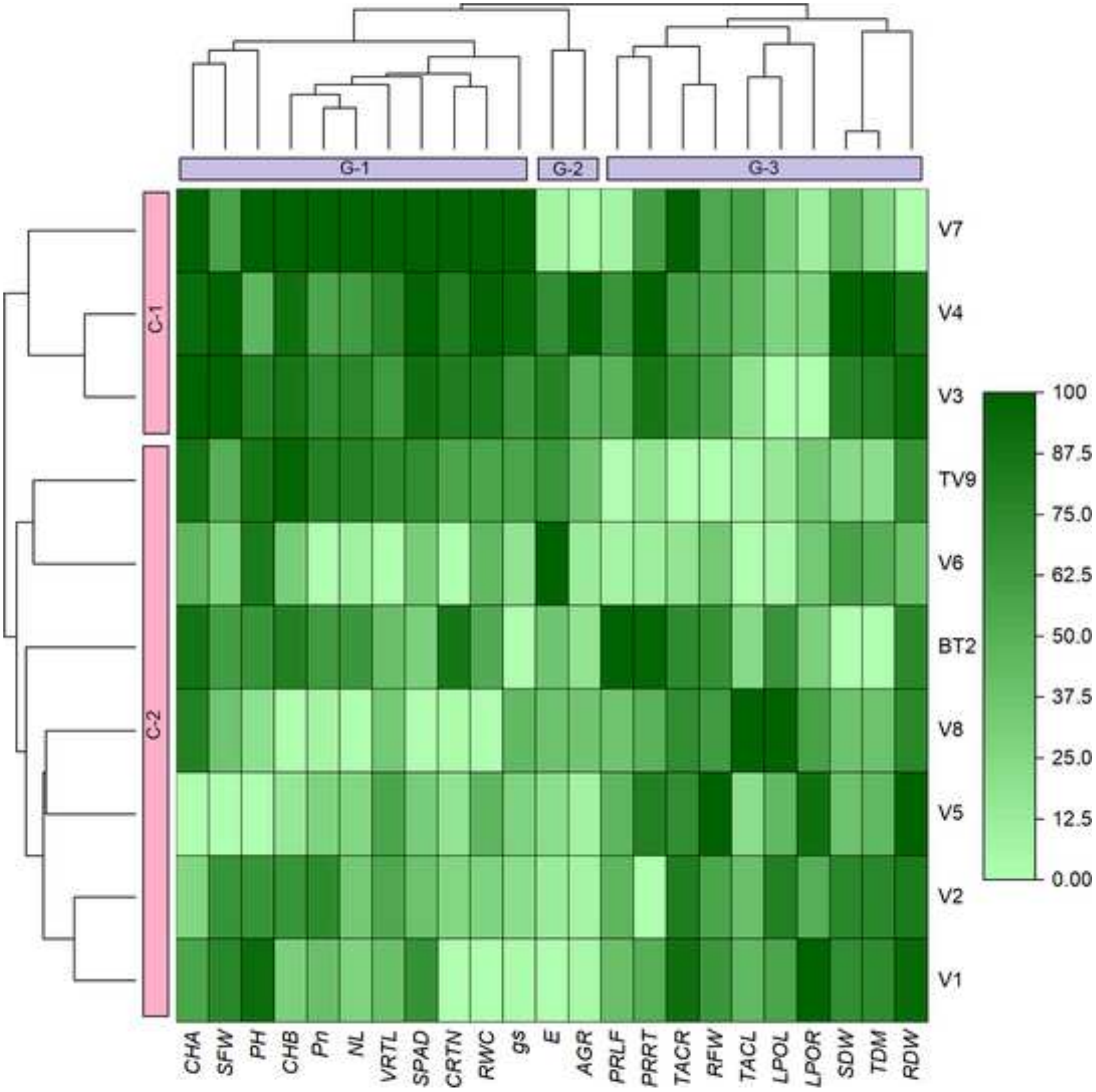

Figure12 Click here to access/download;Figure;Fig12.tif

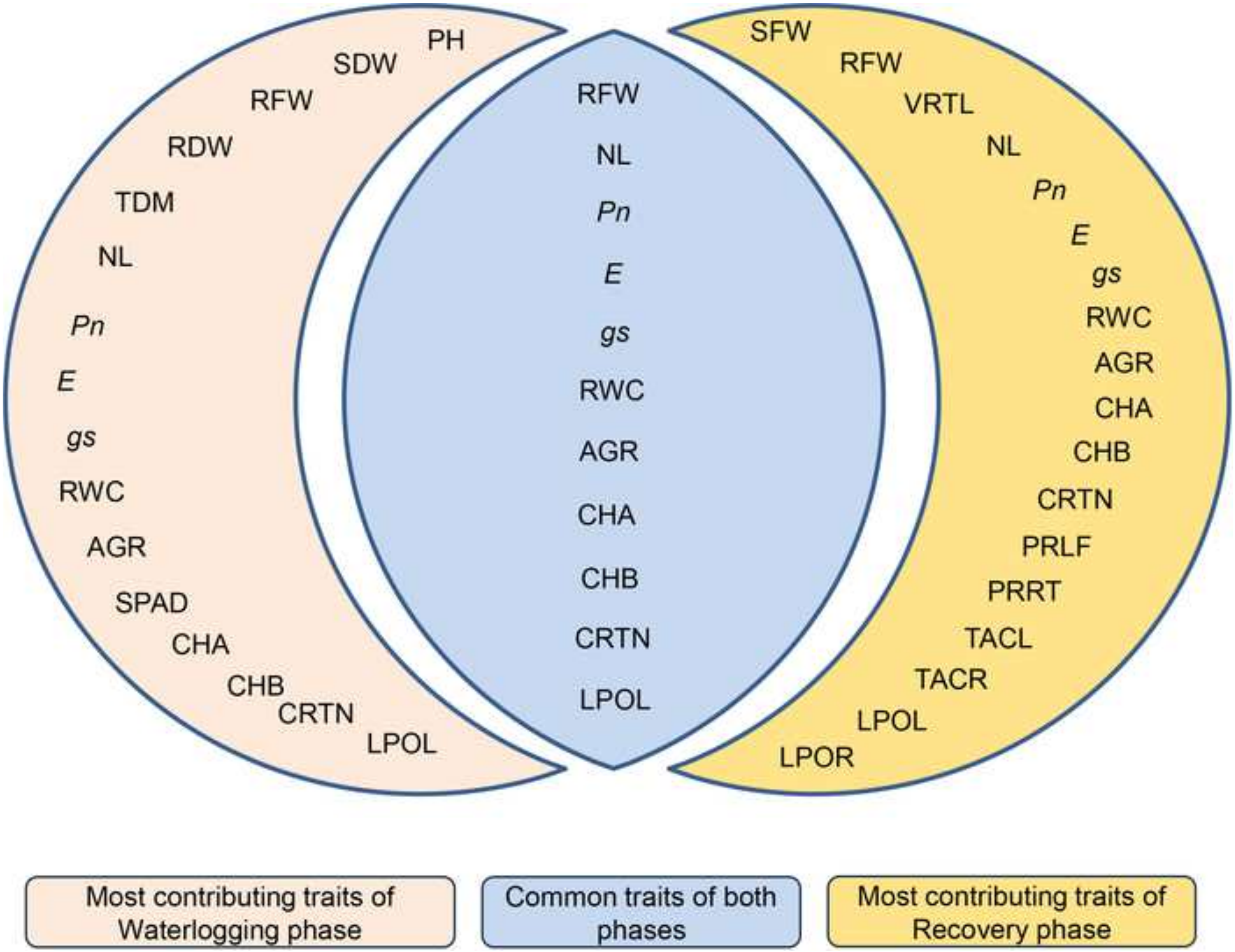

Figure13

Click here to access/download;Figure;Fig13.tif

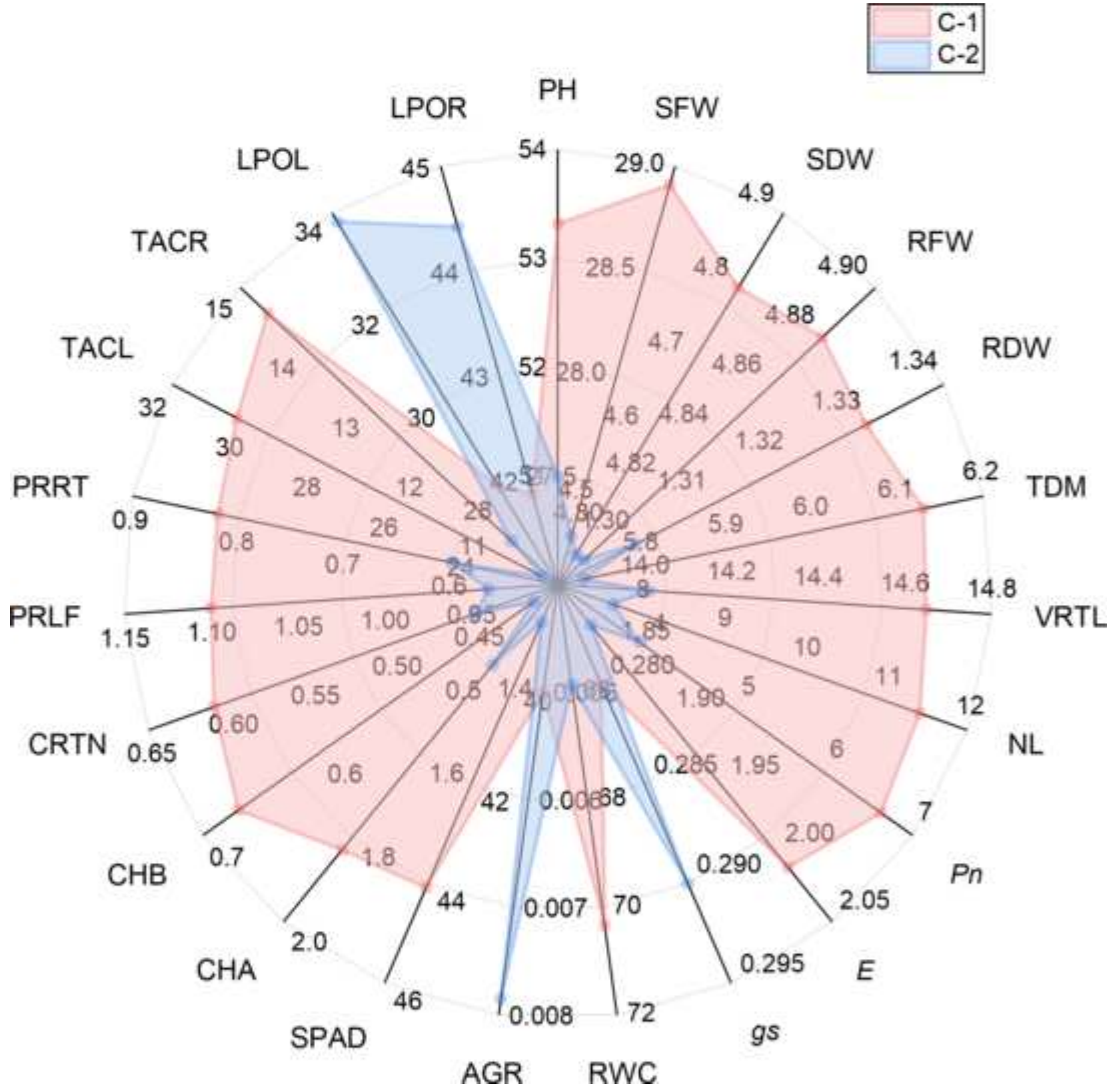

Figure14

Click here to access/download;Figure;Fig14.tif

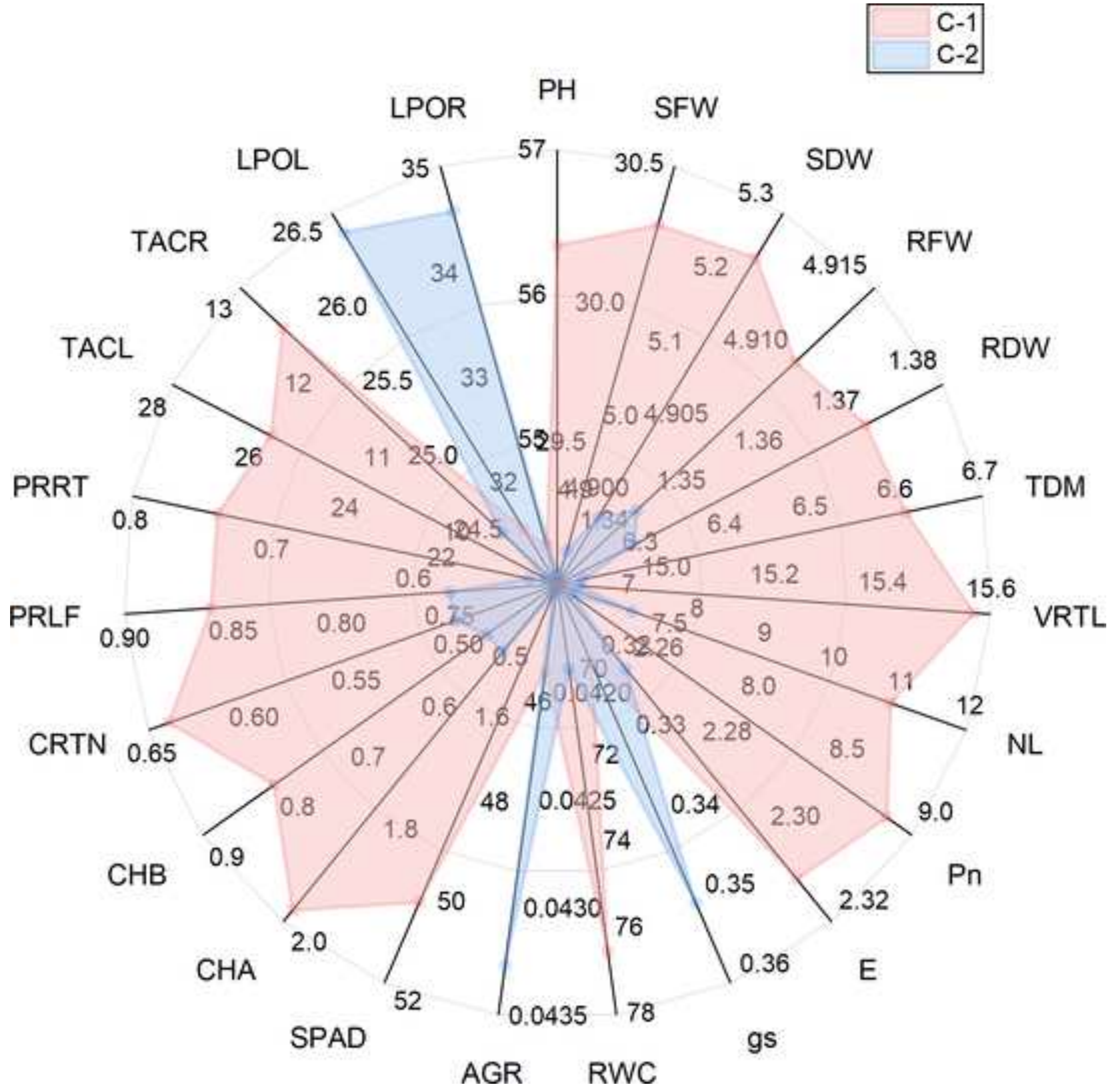

Figure15

Click here to access/download;Figure;Fig15.tif

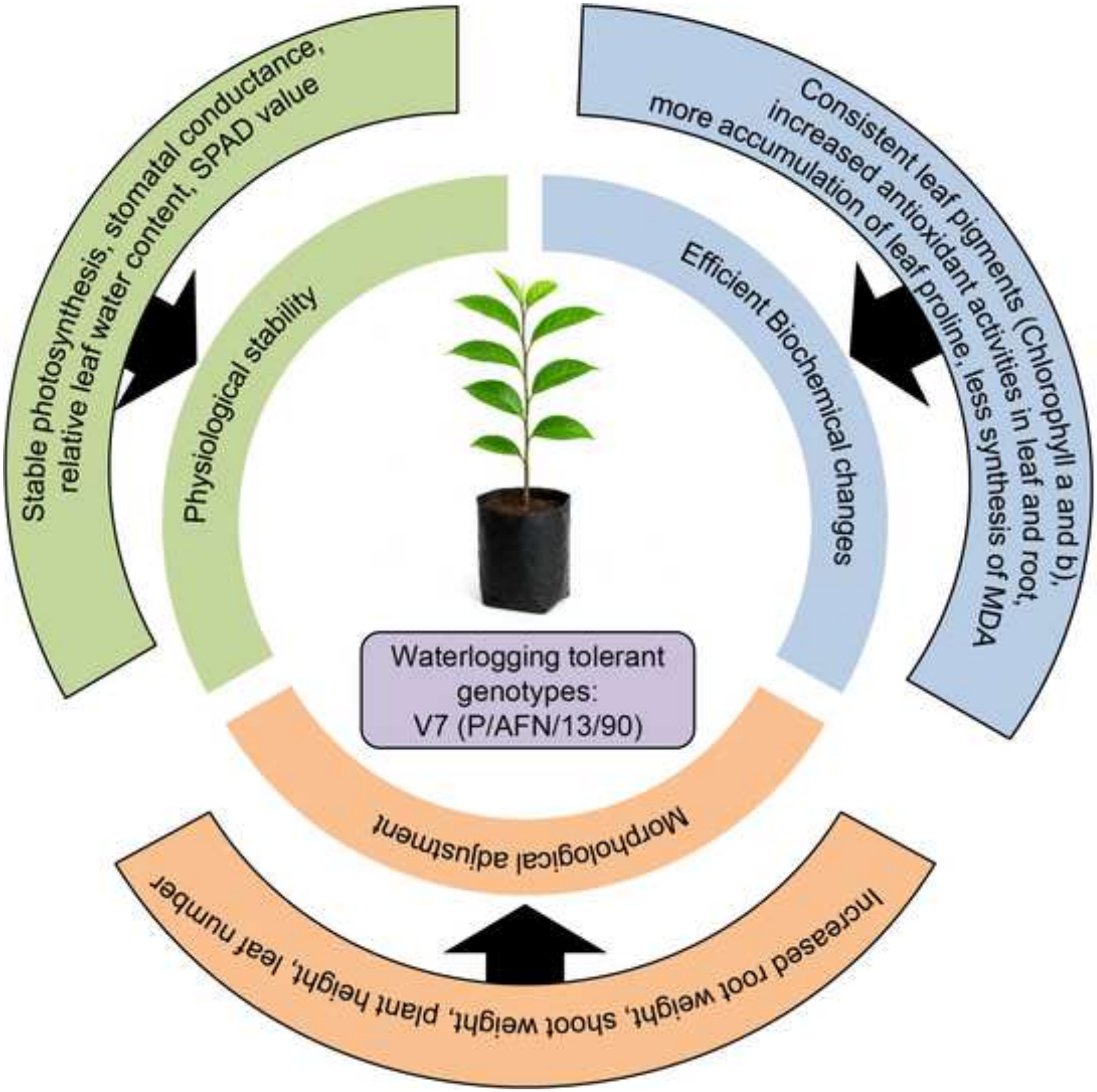

Supporting Table1

Click here to access/download
Supporting Information
S1 Table.docx

Supporting Figure1

Click here to access/download
Supporting Information
S1 Fig.tif

Supporting Figure2

Click here to access/download
Supporting Information
S2 Fig.tif

Supporting Figure3

Click here to access/download
Supporting Information
S3 Fig.tif

Supporting Figure4

Click here to access/download
Supporting Information
S4 Fig.tif

Supporting Figure5

Click here to access/download
Supporting Information
S5 Fig.tif

Supporting Figure6

Click here to access/download
Supporting Information
S6 Fig.tif

Supporting Figure7

Click here to access/download
Supporting Information
S7 Fig.tif

Supporting Figure8

Click here to access/download
Supporting Information
S8 Fig.tif

Supporting Figure9

Click here to access/download
Supporting Information
S9 Fig.tif

Supporting Figure10

Click here to access/download
Supporting Information
S10 Fig.tif

Supporting Figure11

Click here to access/download
Supporting Information
S11 Fig.tif

Supporting Figure12

Click here to access/download
Supporting Information
S12 Fig.tif

Supporting Figure13

Click here to access/download
Supporting Information
S13 Fig.tif

Supporting Figure14

Click here to access/download
Supporting Information
S14 Fig.tif

Supporting Figure15

Click here to access/download
Supporting Information
S15 Fig.tif

Supporting Figure16

Click here to access/download
Supporting Information
S16 Fig.tif

Supporting Figure17

Click here to access/download
Supporting Information
S17 Fig.tif

Supporting Figure18

Click here to access/download
Supporting Information
S18 Fig.tif

Supporting Figure19

Click here to access/download
Supporting Information
S19 Fig.tif

Supporting Figure20

Click here to access/download
Supporting Information
S20 Fig.tif

Supporting Figure21

Click here to access/download
Supporting Information
S21 Fig.tif

Supporting Figure22

Click here to access/download
Supporting Information
S22 Fig.tif

Supporting Figure23

Click here to access/download
Supporting Information
S23 Fig.tif

Supporting Figure24

Click here to access/download
Supporting Information
S24 Fig.tif

Supporting Figure25

Click here to access/download
Supporting Information
S25 Fig.tif

Supporting Figure26

Click here to access/download

Supporting Information

S26 Fig.tif

Supporting Figure27

Click here to access/download
Supporting Information
S27 Fig.tif
