# Supplementary material for: Assessment of waterlogging tolerance in tea genotypes through morpho-physiological and biochemical profiling
Source: PLoS One. 2026 Jul 20;21(7):e0354144. doi: 10.1371/journal.pone.0354144 (PMC13384526; doi:10.1371/journal.pone.0354144)
Supplement: Attachment — Submitted filename: Response to Reviewers.doc [file pone.0354144.s036.doc]

**Response to comments of academic editor**

Comments: (i) clarification and improvement of the experimental design and statistical analysis, including justification of the analytical approach and definition of experimental units,

Authors’ response: Thank you for this valuable comment. We have revised the manuscript to clarify the experimental design and statistical analysis. The analytical approach is now better justified, and the experimental units are explicitly defined in the *Materials and Methods* section.

Comments: (ii) more detailed and transparent methodological descriptions to ensure reproducibility,

Authors’ response: Thank you for this helpful comment. We have revised the *Materials and Methods* section to provide more detailed and transparent descriptions of the experimental procedures and statistical analyses. Specifically, we clarified the experimental design, and provided a stronger justification for the selected analytical approach. Additional information on data collection, treatment structure, replication, and statistical procedures has also been included to ensure that the study can be accurately interpreted.

Comments: (iii) improvement in the presentation and interpretation of results, avoiding redundancy and ensuring consistency across figures and tables,

Authors’ response: Thank you for this valuable comment. We have revised the *Results* section to improve the presentation. Redundant descriptions have been removed. We also carefully reviewed all figures and tables to ensure consistency in data presentation.

Comments: (iv) a more critical and mechanistic discussion supported by relevant and up-to-date literature.

Authors’ response: Thank you for this constructive comment. We have substantially revised the Discussion section to provide a more critical and mechanistic interpretation of the findings. We have also incorporated relevant and recent literature to support the interpretations, and to compare our findings with previous studies.

**Response to comments of Reviewer #1:**

Abstract

Comments: Clarify how tolerance was quantitatively determined.

Authors’ response: We would like to express our sincere thankfulness for your appreciation and efforts spent reviewing this paper, which will undoubtedly improve the manuscript's exposure and quality. We have carefully considered your suggestions and corrected them accordingly.

The tolerance was quantitatively determined by stress tolerance indices which has been included in abstract (L- 26, 27 of *MS* without track changes).

Comments: Avoid listing excessive abbreviations.

Authors’ response: We have carefully revised the abstract and reduced the number of abbreviations to only those that are essential.

Comments: State key numerical findings more concisely.

Authors’ response: We have revised the abstract to present the main numerical results in a more concise manner, emphasizing only the most important values.

Introduction

Comments: Improve flow and reduce repetition.

Authors’ response: Thank you for this constructive comment. Repetitive statements and redundant descriptions have been removed, and several sentences have been reorganized to ensure more logical presentation.

Comments: Strengthen gap identification.

Authors’ response: We have strengthened the identification of the research gap and included briefly (L: 107 - 120 of *MS* without track changes).

Comments: Clearly state novelty compared to previous tea studies.

Authors’ response: This study integrates both stress tolerance and post-waterlogging recovery responses by systematically evaluating the relative contributions of key adaptive traits under Bangladesh conditions, thereby providing a comprehensive framework for identifying waterlogging-tolerant tea genotypes. We have clarified the novelty of the study by distinguishing it from previous tea research and clearly emphasizing its unique objectives, and contributions (L: 107 - 120 of *MS* without track changes).

Materials and Methods

Comments: Clarify experimental unit and replication.

Authors’ response: We have revised the Materials and Methods section to clearly define the experimental unit and provide detailed information on the replication scheme used in the study (L: 130 - 134 of *MS* without track changes).

Comments: Provide more detail on environmental conditions (temperature, humidity).

Authors’ response: We have included the detailed environmental conditions, including temperature and relative humidity, which are now provided in Supplementary Figure S1 (L: 189 - 195 of *MS* without track changes)..

Comments: Specify light intensity during measurements.

Authors’ response: Thank you for the comment. We have revised the manuscript to specify the light intensity used during gas-exchange measurements, and this information has been included in the Materials and Methods section (L: 120 - 123 of *MS* without track changes).

Comments: Indicate calibration details of instruments.

Authors’ response: We have included instrument calibration details, and the corresponding calibration curves have been incorporated into the revised version (L: 249- 282 of *MS* without track changes).

Comments: Clarify whether measurements were taken at the same time of day.

Authors’ response: We have clarified in the revised manuscript that measurements of each interval were conducted at the same of day (L: 181 - 182 of *MS* without track changes).

Results

Comments: Reduce repetition between text and figures.

Authors’ response: We have corrected the manuscript to minimize repetition between the text and figures

Comments: Present key numerical values in tables rather than descriptive narration.

Authors’ response: We have revised the manuscript by presenting key numerical values in tables/ figures and reducing extensive descriptive narration to improve clarity and facilitate data interpretation.

Comments: Ensure figure numbering consistency.

Authors’ response: We have carefully checked and revised the manuscript to ensure consistent and correct figure numbering throughout the text and figure captions.

Comments: Improve clarity of clustering interpretation.

Authors’ response: We have improved the clarity of the clustering interpretation by providing a more detailed explanation of cluster formation, relationships among groups, and their mechanisms under different conditions (L: 767 - 809 of *MS* without track changes).

Discussion

Comments: Avoid restating results.

Authors’ response: We have avoided unnecessary restatement of results and focused the discussion on the interpretation, significance, and implications of the findings.

Comments: Strengthen mechanistic explanations.

Authors’ response: We have strengthened the mechanistic explanations by incorporating relevant morpho-physiological and biochemical processes and supporting them with appropriate literature references.

Comments: Compare findings more deeply with previous tea studies.

Authors’ response: Thank you for the suggestion. Since studies on waterlogging effects in tea are very limited, we have compared our findings with the few available tea-related reports and supplemented the discussion with relevant evidence from other abiotic stress studies in tea to provide broader physiological context.

Comments: Distinguish clearly between stress response and adaptive tolerance.

Authors’ response: The response and tolerance were discussed together from an integrated morpho-physiological and biochemical point of view during waterlogging and the recovery phase.

Figures and Tables

Comments: Improve resolution and readability.

Authors’ response: We have revised all figures according to the journal's guidelines, improving their resolution, readability, and overall presentation quality.

Comments: Shorten overly long figure captions.

Authors’ response: We have shortened some figure captions by retaining only essential information while ensuring that the figures remain clear, informative, and self-explanatory.

Comments: Ensure consistency in abbreviation formatting.

Authors’ response: We have carefully reviewed the manuscript and standardized the formatting of all abbreviations to ensure consistency throughout the text, tables, and figures.

Comments: Verify consistency between text and supplementary figures.

Authors’ response: We have thoroughly cross-checked the manuscript and supplementary figures to ensure consistency in the presentation, labeling, and interpretation of the data throughout the revised version.

4. Minor Comments

Comments: Check consistency of abbreviations (LPOL vs LPOL/LPOR formatting).

Authors’ response: We have carefully reviewed and corrected the abbreviation formatting throughout the manuscript and related terms in the text, tables, figures, and supplementary materials.

Comments: Correct typographical errors.

Authors’ response: Thank you for the comment. We have carefully proofread the entire manuscript and corrected typographical, grammatical, and formatting errors throughout the revised version.

Comments: Ensure all supplementary figures are referenced properly.

Authors’ response: We have carefully cited supplementary figures and referenced in the text where necessary.

Comments: Standardize units (e.g., µmol CO₂ m⁻² s⁻¹ formatting).

Authors’ response: We have revised the manuscript to standardize the formatting of all units,

Comments: Correct repetition in correlation description (e.g., duplicated trait names).

Authors’ response: We have carefully reviewed the correlation analysis section and corrected the repeated trait names and other redundant descriptions to improve clarity and accuracy.

5. Final Recommendation

Comments: The study is scientifically relevant and potentially valuable for tea breeding programs. However, major revisions are required to improve statistical rigor, interpretation depth, and clarity of presentation.

Authors’ response: Thank you for your valuable suggestions. We have incorporated the queries in the revised manuscripts.

*Thanks again for your valuable time and please check the revised manuscript and we made the corrections/improvements in track changed mode.*

**Response to comments of Reviewer #2:**

Comments: 1. Abstract was long, delete or change the parts as I suggest in the reviewed manuscript (lines 25-27, 31-32).

Authors’ response: We wish to express our sincere gratitude for taking the time to review this manuscript. We appreciate your valuable feedback which will certainly improve the quality of the manuscript. We have respectfully considered your valuable comments and revised the manuscript accordingly.

We have revised the Abstract by deleting and modifying the indicated lines.

Comments: 2. Keywords were absent, add them.

Authors’ response: We have added appropriate keywords to the revised manuscript to improve its indexing, discoverability, and alignment with the journal’s requirements.

Comments: 3. Move lines 74-83 as I suggest in the reviewed manuscript.

Authors’ response: We have revised the manuscript accordingly and moved the content from Lines 74–83 to the suggested location to improve the logical flow.

Comments: 4. Delete lines 83-91 as I suggest in the reviewed manuscript.

Authors’ response: We have revised the manuscript accordingly and deleted the text in Lines 83–91 as recommended.

Comments: 5. Line 126: “You must design and analyze the data using a randomized complete block design (RCBD) rather than CRD if the experiment conducted in outdoor. CRD is usually used to design the experiments that the condition of the experiment totally controlled, such as in laboratory or protected agriculture in controlled greenhouses. Therefore, write about the condition of the experiment site.”

Authors’ response: Thank you for your valuable comments. The justification for the CRD experiment has provided with the condition of the experimental site (L: 189 - 198 of *MS* without track changes).

Comments: 6. Lines 128-134: “Write the temperature and humidity of the experiment site because the intensity of waterlogging is directly related to temperature.”

Authors’ response: The mean temperature and humidity of the experimental site are provided in supplementary figure (S1 figure) (L: 193 - 195 of *MS* without track changes).

Comments: 7. Line 156: Change to “number of leaves (NL)”

Authors’ response: The correction has been done.

Comments: 8. Lines 170-171: “What are FW, DW, TW, W1, and W2? They must be explained.”

Authors’ response: The abbreviations with explanation are provided in L: 222 - 231 of *MS* without track changes.

Comments: 9. Line 174: “Three readings per leaf are few for SPAD. It was better to have five or more.”

Authors’ response: Thank you for your valuable suggestion. This will be included in our future research as a valuable direction.

Comments: 10. It is better to italicize the symbol letters of chlorophylls “a” and “b” throughout the manuscript, text, tables, and figures to distinguish them from ordinary letters.

Authors’ response: Thank you for the valuable suggestion. We have revised the manuscript to italicize the symbols of chlorophyll ‘a’ and chlorophyll ‘b’ consistently throughout the manuscript.

Comments: 11. Line 178: “Were the samples dry or fresh? How were they ground”? - Authors’ response: The samples used for analysis were fresh, and the extraction procedures have been clearly described in the *Materials and Methods* section of the revised manuscript (L: 237 - 245 of *MS* without track changes).

Comments: 12. Line 179: “According to which reference? To extract chlorophyll, usually, the ground leaf samples are left overnight.”

Authors’ response: Thank you for the comment. We have clarified the chlorophyll extraction procedure in the revised manuscript and included the appropriate reference supporting the method used. The extraction protocol, including sample preparation and extraction duration, is now described in greater detail in the *Materials and Methods* section (L: 237 - 245 of *MS* without track changes).

Comments: 13. Line 202: “Were you conducting a comparison among means? If you, write the post hoc test. I strongly recommend displaying the results analytically rather than descriptively.”

Authors’ response: We have performed post hoc test. In the figures (S2-S24), lettering was not provided because of overcrowding of data, and it also reduced the readability of figures drastically. The LSD values are now incorporated in ANOVA (S1 table).

Comments: 14. Lines 223-231: “What is the aim of this description and its Figure? It looks like a summary of the results, so this must be the end of the Result section rather than at the beginning.”

Authors’ response: The figure mainly showed the difference between waterlogging stress and control conditions, using descriptive statistics.

Comments: 15. Lines 248-251: “This introduction is not needed (delete). This makes your results long and cumbersome for the readers.”

Authors’ response: Thank you for the comment. This introduction has been removed.

Comments: 16. Delete lines 352-355.

Authors’ response: Thank you for the comment. These lines have been removed.

Comments: 17. Lines 355-356: “Refer to this part of the PCA that was under waterlogging.”

Authors’ response: The correction has been done accordingly.

Comments: 18. Delete Table 1 (lines 369-372).

Authors’ response: Thank you for the comment. This table has been moved to supplementary.

Comments: 19. Delete lines 388-391.

Authors’ response: Thank you for the comment. These marked lines have been removed.

Comments: 20. Lines 391-392: “Refer to this part of the PCA under the recovery phase.”

Authors’ response: Thank you for the suggestion. The correction has been done accordingly.

Comments: 21. Delete Table 2 (lines 405-408).

Authors’ response: Thank you for the comment. This table has been moved to supplementary.

Comments: 22. Lines 488-490: “You should start with description of G-1, G-2, and G-3 rather than start with G-3.”

Authors’ response: Thank you for the suggestion. The corrections have made according to your comment.

Comments: 23. Delete lines 542-573, this part was not needed. This subsection is a literature review mixed with materials and methods rather than a discussion of the achieved results in this study. In the Discussion section, the authors must answer the "how" and "why" of their obtained results.”

Authors’ response: Thank you for the valuable suggestion. We have revised the Discussion section accordingly by moving the contents.

Comments: 24. Find the comments one by one in the reviewed manuscript.

Authors’ response: Thank you for the comment. We have carefully reviewed the manuscript and addressed all comments individually.

*Thanks again for your valuable time and please check the revised manuscript and we made the corrections/improvements in track changed mode*.

**Response to comments of Reviewer #3:**

Comments: A core part of the work is the analysis of variance. I find a strong misconception that experimental design also uses the method of projecting the results of the analysis of variance, which does not allow for a general understanding of statistical trends.

Authors’ response: The ANOVA has now been revised and corrected using an appropriate statistical model, and the corresponding explanations have been clarified in the revised manuscript to ensure accurate interpretation of the experimental data. The factors are included. The analytical justification was provided in *Materials and methods* section. Thanks again for your valuable time and please check the revised manuscript and we made the corrections/improvements in track changed mode.

**Response to comments of Reviewer #4:**

Comments: The manuscript is interesting, and the results could help in obtaining waterlogged genotypes for genetic improvement programs. However, it is very long (over 60 pages) and contains an excessive number of figures. I never found Table 1, which deals with the analysis of variance.

Authors’ response: Thank you for the comment. We have revised the manuscript to improve its conciseness by reducing redundant text. In addition, several tables have been moved to the Supplementary Materials, to reduce the overall length and improve readability. The analysis of variance (ANOVA) table was provided in the supplementary section (S1 table).

Comments: In the materials and methods section, the origin of the plants is not described, either from seed production or vegetatively. How and under what conditions were the plants produced (substrate used, greenhouse conditions, plant management, etc.) is not explained.

Authors’ response: We have revised the Materials and Methods section to provide detailed information on the origin of the plant materials, including their mode of propagation, as well as the conditions under which they were produced and maintained. Details regarding the environmental conditions, and plant management practices have now been included (L: 140 - 160 of *MS* without track changes).

Comments: It is also unclear how the plants were established (bare-rooted, in some type of container, or in bags) inside the plastic boxes. It is important to monitor the oxygen level of the waterlogged water during the experiment, as this is a significant factor (hypoxia or anoxia).

Authors’ response: The establishment procedure of the plant is explained in detailed in the revised Materials and Methods section (L: 161 - 183 of *MS* without track changes). Though dissolved oxygen was not measured during the present experiment. We agree that these measurements would provide additional insight into the waterlogging environment, and therefore, dissolved oxygen will be included in our future experiments.

Comments: The number of plants used to evaluate the waterlogged treatment, which was destructive, is not mentioned, nor is the number of plants that remained to evaluate recovery. The experimental design could have been better as a completely randomized factorial design, since only two factors (genotype and waterlogging treatment) were analyzed.

Authors’ response: Thank you for the valuable comment. We have revised the *Materials and Methods* section to clearly specify the number of plants used for destructive sampling during the waterlogging phase and the number of plants retained for recovery assessment (L: 172 - 179 of *MS* without track changes). We have also clarified the experimental design and statistical analysis employed in the study, including the factors considered and their structure, to improve transparency and reproducibility (L: 189- 198 of *MS* without track changes).

Comments: There were experimental units of 5 plants (one replicate) and three replicates were used, 15 plants per 10 genotypes = 150, in two treatments, for a total of 300 plants. Why is 500 mentioned?

Authors’ response: It was a typographical mistake and clarified with sufficient information about the plant’s number (L: 172 - 179 of *MS* without track changes)

Comments: It is worthwhile to include the statistical model used for data analysis. Mention whether a normality test was performed on the data

Authors’ response: We have performed a normality test prior to ANOVA and this information was stated in *Statistical analysis* section of *Material and Methods* (L: 285 - 287 of *MS* without track changes)

Comments: The results are well described, but further discussion of the findings is needed. It is not necessary to discuss the method used to establish the waterlogging treatments; focus on the discussion of the results.

Authors’ response: The *Discussion* section has been revised to place greater emphasis on interpreting and highlighting the biological significance of the results, including the physiological and biochemical responses of the tea genotypes under waterlogging and recovery conditions.

Comments: Finally, I believe that 14 days of treatment and recovery is insufficient, and the plants were not stressed enough to generate better results.

Authors’ response: The 14-day waterlogging and 14-day recovery periods used in the present study were chosen based on preliminary observations in nursery conditions without causing complete sapling mortality. The extended stress and recovery durations would strengthen the observations and conclusions and would be included in our future experiments as a research direction.

*Thanks again for your valuable time and please check the revised manuscript and we made the corrections/improvements in track changed mode.*

**Response to comments of Reviewer #5:**

Abstract

Comments: The abstract presents the general aim and main findings clearly; however, it is overly descriptive and contains several vague expressions such as “remarkably”, “substantially”, and “better responses”. These statements should be supported with key numerical values.

Authors’ response: We appreciate your valuable comments and express our heartiest gratitude for handling this manuscript. We have revised the abstract accordingly, as recommended.

Comments: The authors state that 11 traits were the most influential, but the statistical basis for this selection is not clearly explained in the abstract. The criterion should be briefly stated, for example PCA contribution, correlation strength, or consistency across stress and recovery phases.

Authors’ response: We have revised the abstract by including the statistical basis for the quantification of tolerance as well as selection of influential traits.

Comments: There is an inconsistency in abbreviations: lipid peroxidation is written as both LOPL and LPOL. This should be standardized throughout the manuscript. -revised

Authors’ response: We have revised the manuscript accordingly. We used LOPL for leaf, and LPOR for root proline contents.

Introduction

Comments: The introduction provides sufficient background on waterlogging stress and its relevance to tea production. However, the novelty of the study is not sharply defined. The authors should explain more clearly how this work differs from previous tea waterlogging screening studies and why these specific eight genotypes were selected.

Authors’ response: This study integrates both stress tolerance and post-waterlogging recovery responses by systematically evaluating the relative contributions of key adaptive traits under Bangladesh conditions, thereby providing a comprehensive framework for identifying waterlogging-tolerant tea genotypes. We have clarified the novelty of the study by distinguishing it from previous tea research and clearly emphasizing its unique objectives, and contributions (L: 107 - 120 of *MS* without track changes). We also mentioned the sources of these specific eight genotypes in *Materials and Method*s section (L: 137 - 145 of *MS* without track changes).

Comments: The Bangladesh-specific problem is mentioned, but the production-level impact, affected regions, and practical breeding relevance are not sufficiently developed. A stronger justification is needed for why identifying tolerant genotypes at the sapling stage is agronomically meaningful.

Authors’ response: The worldwide affected regions by waterlogging, including Bangladesh, with their impacts have been included in the *Introduction* section (L: 81 - 87 of *MS* without track changes). Screening at the sapling stage enables rapid, cost-effective, and large-scale evaluation of breeding materials before field trials. This justification for using saplings has been provided in the *Materials and Methods* section (L: 135 - 137 of *MS* without track changes).

Comments: The objectives are clear, but they are broad. The authors should refine them into testable objectives linked directly to the measured traits and statistical analyses.

Authors’ response: The objectives have now been refined to be more specific, measurable, and directly linked to the evaluated morphological, physiological, and biochemical traits, as well as to the statistical analyses conducted in the study (L: 120 - 125 of *MS* without track changes).

Comments: I recommend supporting the first sentence (“Plants face various types of biotic and abiotic stresses throughout their life cycles”) with more recent and relevant literature (e.g., https://doi.org/10.1186/s44399-025-00024-8; https://doi.org/10.1186/s12870-025-07964-y). Similarly, the second sentence regarding waterlogging as a major abiotic stress should also be supported with an appropriate and up-to-date reference (e.g., Toprak, S., & Coşkun, Ö. F. (2024). Effect of gibberellic acid on waterlogging stress in melon. Proceedings of the VI International Agricultural, Biological, Life Science Conference (AGBIOL 2024), 358.).

Authors’ response: Thank you for your valuable suggestions. We have cited current and up-to-date references as your suggestions in *Introduction* and *Discussions* [*Reference* no 2 and 3].

Materials and methods

Comments: The experimental design requires clarification. The authors state that the study followed a CRD with three replications and that five plants were considered one replicate, but it is not fully clear whether the experimental unit was the box or the individual plant. This is important because five plants in the same box may not represent independent biological replicates.

Authors’ response: Thank you for the important comment. We have clarified the experimental design in the revised manuscript. Each plant was individually grown in a separate polybag and was considered an individual experimental unit. During the waterlogging treatment, polybag-grown plants belonging to the same genotype and replication were placed in a common plastic box solely to maintain a uniform waterlogging condition and optimize resource use. Since all plants were raised individually and maintained under a uniform growth environment, the use of shared boxes did not alter the independence of the experimental units.

Comments: The waterlogging treatment is described as maintaining water 5 cm above the collar region for 14 days, followed by 14 days of recovery. However, the authors do not report whether dissolved oxygen, soil/redox status, temperature, light conditions, or water renewal were monitored. These details are necessary to confirm that the imposed stress was consistent among genotypes.

Authors’ response: We have missed the parameter, dissolved oxygen. These measurements would provide additional insight into the waterlogged environment; therefore, dissolved oxygen will be included in our future experiments for a more comprehensive evaluation of stress conditions.

Comments: The control treatment is described only as “good cultural practices and ideal irrigation”. This is too vague. The authors should define irrigation frequency, substrate moisture status, pot/polybag size, growth medium, drainage condition, and nutrient management.

Authors’ response: We have revised the *Materials and Methods* section to provide a detailed description of the control treatment, including irrigation frequency, polybag specifications, growth medium composition, and nutrient management practices (L: 150 - 160 of *MS* without track changes)..

Comments: The biochemical methods are insufficiently detailed. The phrase “with some slight change/minor changes” is not acceptable unless the exact modifications are described. The proline, antioxidant capacity, and TBARS protocols should include extraction volume, reagent composition, incubation conditions, calibration/standard curve details, and calculation formulas.

Authors’ response: The detailed protocols were mentioned in the revised manuscript as per you recommendation (L: 249 - 282 of *MS* without track changes).

Comments: The statistical analysis is incomplete. The model structure, factors, interaction terms, mean separation test, significance thresholds, data transformation, and assumptions of ANOVA should be reported. Since repeated measurements were taken at 7-day intervals, the authors should justify why a repeated-measures or mixed model was not used.

Authors’ response: The statistical analysis is now well stated with ANOVA model, least significant test, normality test etc. (L: 285 - 298 of *MS* without track changes). In current study, mixed model was not used due to following the destructive methods (L: 181 - 183 of *MS* without track changes) of sampling analysis.

Results

Comments: The results contain many numerical comparisons, but the presentation is often repetitive. The authors should focus on statistically supported genotype × treatment differences rather than listing the “best” genotype for each trait.

Authors’ response: The results section is now well written. Interaction effects were placed in supplement figures. Results mainly focused on relative changes in stress conditions compared to control and stress tolerance indices, because these are the key determinants for quantifying stress tolerance.

Comments: The statement that V7 was the most tolerant genotype needs stronger quantitative support. Some traits identify other genotypes as superior, for example V5 for root fresh weight, V4 for several recovery traits, V3 for lipid peroxidation, and V6 for transpiration. Therefore, a clear tolerance index or ranking system should be provided.

Authors’ response: Stress tolerance indices (STI) were calculated for each trait. To identify the best genotype(s), clustering heatmaps were constructed using STI values for each parameter during both the waterlogging and recovery phases.

Comments: The PCA interpretation is not fully convincing. With only ten genotypes and 23 traits, PCA may be unstable and potentially over-interpreted. The authors should justify the use of PCA under this sample size and avoid making strong biological conclusions only from biplot positions.

Authors’ response: Genotype identification was not based only on PCA; clustering heatmaps were also considered together to support the interpretation and identification of potential waterlogging-tolerant genotypes. This clarification has now been incorporated into the revised *Results* and *Discussion* sections.

Comments: The correlation analysis is descriptive and does not sufficiently distinguish biologically meaningful correlations from statistical artifacts caused by many variables and small genotype number. Multiple-testing correction or at least a cautious interpretation is needed.

Authors’ response: In this study, the correlation analysis was used primarily as an exploratory tool to identify possible relationships among physiological, biochemical, and growth traits. To strengthen the interpretation of these associations, principal component analysis (PCA) was additionally performed, which also helped to identify the most influential traits. The PCA results supported the trends observed in the correlation analysis. We have now avoided repeated interpretation of the correlation results.

Comments: Figures are informative but crowded. Several figures contain too many abbreviations and small labels, reducing readability. The authors should improve figure resolution, simplify captions, and move excessive abbreviation lists to a separate note or supplementary table.

Authors’ response: The figures have now been uploaded in accordance with the journal's recommended resolution and font-size requirements to improve overall readability. The abbreviations provided after each figure and table were intentionally included to help readers immediately understand the abbreviated terms without repeatedly referring to separate sections. However, to improve figure clarity and reduce overcrowding, we have revised figures, discarded several tables, and reduced unnecessary abbreviations wherever possible in the revised manuscript.

Discussion

Comments: The discussion should be more analytical. At present, it appears to restate the results rather than explaining the physiological basis of tolerance. The authors should better connect V7 tolerance with maintenance of photosynthesis, stomatal conductance, RWC, pigment stability, antioxidant capacity, and lower oxidative damage.

Authors’ response: The waterlogging tolerance mechanism of genotype V7 (P/AFN/13/90) during both the stress and recovery phases has been discussed in more detail by integrating morpho-physiological and biochemical responses. Furthermore, a hypothetical mechanistic pathway illustrating the coordinated tolerance and recovery responses of V7 under waterlogging conditions has also been included in the manuscript to provide a clearer conceptual understanding of the observed adaptive mechanisms (L: 825 - 841 of *MS* without track changes).

Comments: The discussion should address contradictory findings. For example, if V7 is the most tolerant genotype, the authors should explain why other genotypes performed better for some root, dry matter, transpiration, or lipid peroxidation traits.

Authors’ response: We agree that interpretation based solely on absolute trait values may sometimes be misleading under stress conditions. In several cases, some genotypes exhibited higher values for individual traits; however, these traits often declined more severely under waterlogging stress compared with the control condition. Therefore, the identification of tolerant genotypes in the present study was primarily based on Stress Tolerance Index (STI) values, which more effectively capture the relative stability and maintenance of traits under stress. In addition, STI values were evaluated at both the end of the waterlogging period and after the recovery phase to assess stress tolerance. Although V7 (P/AFN/13/90) did not always exhibit the highest absolute values for all parameters, it showed greater stability across a broad range of physiological, biochemical, and growth traits during both the stress and recovery phases.

Comments: The role of recovery phase is not sufficiently discussed. Recovery performance is highly important in waterlogging tolerance, and the authors should distinguish between genotypes that tolerate stress during flooding and those that recover better after drainage.

Authors’ response: We have now enlarged our discussion regarding the recovery effect under each head of parameters like, Morphological, Physiological and Biochemical traits under *Discussion* section. We have also identified genotypes that recover faster (L: 797 - 809 of *MS* without track changes) and those that can withstand waterlogging (L: 767 - 779 of *MS* without track changes).

Comments: Important mechanistic traits are missing. Since waterlogging tolerance in tea is often associated with root adaptation and aerenchyma formation, the lack of anatomical/root structural observations should be acknowledged as a limitation.

Authors’ response: Thank you for this valuable suggestion. We acknowledge that important mechanistic traits related to waterlogging tolerance, particularly root anatomical adaptations such as aerenchyma formation and adventitious root development, were not evaluated in the present study. In the current work, our primary focus was on the comparative assessment of physiological, biochemical, and growth responses in tea genotypes under waterlogging stress and during recovery. We agree that the inclusion of such mechanistic analyses would provide deeper insights into the tolerance mechanisms of tea genotypes. Accordingly, this limitation has now been acknowledged in the revised manuscript, and the need for future studies incorporating root anatomical investigations has been highlighted.

Comments: The authors should avoid overclaiming commercial applicability. Since the experiment was conducted under controlled sapling conditions, field validation under naturally waterlogged tea estate conditions is necessary before recommending P/AFN/13/90 as a commercial tolerant genotype.

Authors’ response: We have revised the manuscript to avoid overclaiming its commercial potential and to clarify that further genetic characterization and extensive field evaluations under naturally waterlogged conditions are required before confirming its commercial applicability and waterlogging tolerance.

*Thanks again for your valuable time and please check the revised manuscript and we made the corrections/improvements in track changed mode.*
