# Supplementary material for: Assessment of waterlogging tolerance in tea genotypes through morpho-physiological and biochemical profiling
Source: PLoS One. 2026 Jul 20;21(7):e0354144. doi: 10.1371/journal.pone.0354144 (PMC13384526; doi:10.1371/journal.pone.0354144)
Supplement: Peer review history

# Peer review: Decision Letter 0

**DOI:** 10.1371/journal.pone.0354144.r001

**Authors:** Ömer Faruk Coşkun

27 Apr 2026

PONE-D-26-00282

Assessment of waterlogging tolerance in tea genotypes through morpho-physiological and biochemical profiling

PLOS One

Dear Dr. Arefin,

Thank you for submitting your manuscript to PLOS ONE. After careful consideration, we feel that it has merit but does not fully meet PLOS ONE’s publication criteria as it currently stands. Therefore, we invite you to submit a revised version of the manuscript that addresses the points raised during the review process.

==============================

Based on the reviewer reports and my evaluation, the manuscript requires substantial revision before it can be considered for publication.

The main issues that must be addressed for acceptance are:

(i) clarification and improvement of the experimental design and statistical analysis, including justification of the analytical approach and definition of experimental units,

(ii) more detailed and transparent methodological descriptions to ensure reproducibility,

(iii) improvement in the presentation and interpretation of results, avoiding redundancy and ensuring consistency across figures and tables,

(iv) a more critical and mechanistic discussion supported by relevant and up-to-date literature.

While some reviewers considered the study technically sound, others raised concerns particularly regarding the statistical approach and interpretation of results. These issues must be carefully resolved.

Additional improvements in language clarity, figure readability, and manuscript structure are recommended.

A detailed, point-by-point response to all reviewer comments is required.

==============================

Please submit your revised manuscript by Jun 11 2026 11:59PM. If you will need more time than this to complete your revisions, please reply to this message or contact the journal office at plosone@plos.org. When you're ready to submit your revision, log on to https://www.editorialmanager.com/pone/ and select the 'Submissions Needing Revision' folder to locate your manuscript file.

Please include the following items when submitting your revised manuscript:

- A letter that responds to each point raised by the academic editor and reviewer(s). You should upload this letter as a separate file labeled 'Response to Reviewers'.
- A marked-up copy of your manuscript that highlights changes made to the original version. You should upload this as a separate file labeled 'Revised Manuscript with Track Changes'.
- An unmarked version of your revised paper without tracked changes. You should upload this as a separate file labeled 'Manuscript'.

If you would like to make changes to your financial disclosure, please include your updated statement in your cover letter. Guidelines for resubmitting your figure files are available below the reviewer comments at the end of this letter.

If applicable, we recommend that you deposit your laboratory protocols in protocols.io to enhance the reproducibility of your results. Protocols.io assigns your protocol its own identifier (DOI) so that it can be cited independently in the future. For instructions see: https://journals.plos.org/plosone/s/submission-guidelines#loc-laboratory-protocols. Additionally, PLOS ONE offers an option for publishing peer-reviewed Lab Protocol articles, which describe protocols hosted on protocols.io. Read more information on sharing protocols at https://plos.org/protocols?utm_medium=editorial-email&utm_source=authorletters&utm_campaign=protocols.

As the corresponding author, your ORCID iD is verified in the submission system and will appear in the published article. PLOS supports the use of ORCID, and we encourage all coauthors to register for an ORCID iD and use it as well. Please encourage your coauthors to verify their ORCID iD within the submission system before final acceptance, as unverified ORCID iDs will not appear in the published article. *Only*  the individual author can complete the verification step; PLOS staff *cannot*  verify ORCID iDs on behalf of authors.

We look forward to receiving your revised manuscript.

Kind regards,

Ömer Faruk Coşkun, Ph.D

Academic Editor

PLOS One

Journal requirements:

When submitting your revision, we need you to address these additional requirements.

1. Please ensure that your manuscript meets PLOS ONE's style requirements, including those for file naming. The PLOS ONE style templates can be found at

https://journals.plos.org/plosone/s/file?id=wjVg/PLOSOne_formatting_sample_main_body.pdf and

https://journals.plos.org/plosone/s/file?id=ba62/PLOSOne_formatting_sample_title_authors_affiliations.pdf

2. When completing the data availability statement of the submission form, you indicated that you will make your data available on acceptance. We strongly recommend all authors decide on a data sharing plan before acceptance, as the process can be lengthy and hold up publication timelines. Please note that, though access restrictions are acceptable now, your entire data will need to be made freely accessible if your manuscript is accepted for publication. This policy applies to all data except where public deposition would breach compliance with the protocol approved by your research ethics board. If you are unable to adhere to our open data policy, please kindly revise your statement to explain your reasoning and we will seek the editor's input on an exemption. Please be assured that, once you have provided your new statement, the assessment of your exemption will not hold up the peer review process.

3. Please remove your figures from within your manuscript file, leaving only the individual TIFF/EPS image files, uploaded separately. These will be automatically included in the reviewers’ PDF.

If the reviewer comments include a recommendation to cite specific previously published works, please review and evaluate these publications to determine whether they are relevant and should be cited. There is no requirement to cite these works unless the editor has indicated otherwise.

Additional Editor Comments:

The manuscript addresses a relevant topic in plant stress physiology; however, all reviewers consistently identified substantial issues that need to be addressed before the manuscript can be considered for publication. These concerns mainly relate to the clarity and robustness of the methodology, the presentation and interpretation of the results, and the depth of the discussion in relation to current literature.

Given the consistent recommendations from all reviewers, a major revision is required. The authors are encouraged to carefully address each reviewer comment in a point-by-point manner and to substantially improve the scientific quality and clarity of the manuscript.

[Note: HTML markup is below. Please do not edit.]

Reviewers' comments:

Reviewer's Responses to Questions

**Comments to the Author**

1. Is the manuscript technically sound, and do the data support the conclusions?

The manuscript must describe a technically sound piece of scientific research with data that supports the conclusions. Experiments must have been conducted rigorously, with appropriate controls, replication, and sample sizes. The conclusions must be drawn appropriately based on the data presented.

Reviewer #1: Yes

Reviewer #2: Yes

Reviewer #3: Partly

Reviewer #4: Partly

Reviewer #5: Yes

**********

2. Has the statistical analysis been performed appropriately and rigorously?

Reviewer #1: Yes

Reviewer #2: Yes

Reviewer #3: No

Reviewer #4: No

Reviewer #5: Yes

**********

3. Have the authors made all data underlying the findings in their manuscript fully available?

The PLOS Data policy requires authors to make all data underlying the findings described in their manuscript fully available without restriction, with rare exception (please refer to the Data Availability Statement in the manuscript PDF file). The data should be provided as part of the manuscript or its supporting information, or deposited to a public repository. For example, in addition to summary statistics, the data points behind means, medians and variance measures should be available. If there are restrictions on publicly sharing data—e.g. participant privacy or use of data from a third party—those must be specified.

Reviewer #1: Yes

Reviewer #2: Yes

Reviewer #3: Yes

Reviewer #4: No

Reviewer #5: Yes

**********

4. Is the manuscript presented in an intelligible fashion and written in standard English?

PLOS ONE does not copyedit accepted manuscripts, so the language in submitted articles must be clear, correct, and unambiguous. Any typographical or grammatical errors should be corrected at revision, so please note any specific errors here.

Reviewer #1: Yes

Reviewer #2: Yes

Reviewer #3: Yes

Reviewer #4: Yes

Reviewer #5: Yes

**********

5. Review Comments to the Author

Please use the space provided to explain your answers to the questions above. You may also include additional comments for the author, including concerns about dual publication, research ethics, or publication ethics. (Please upload your review as an attachment if it exceeds 20,000 characters)

Reviewer #1: Abstract

Clarify how tolerance was quantitatively determined.

Avoid listing excessive abbreviations.

State key numerical findings more concisely.

Introduction

Improve flow and reduce repetition.

Strengthen gap identification.

Clearly state novelty compared to previous tea studies.

Materials and Methods

Clarify experimental unit and replication.

Provide more detail on environmental conditions (temperature, humidity).

Specify light intensity during measurements.

Indicate calibration details of instruments.

Clarify whether measurements were taken at the same time of day.

Results

Reduce repetition between text and figures.

Present key numerical values in tables rather than descriptive narration.

Ensure figure numbering consistency.

Improve clarity of clustering interpretation.

Discussion

Avoid restating results.

Strengthen mechanistic explanations.

Compare findings more deeply with previous tea studies.

Distinguish clearly between stress response and adaptive tolerance.

Figures and Tables

Improve resolution and readability.

Shorten overly long figure captions.

Ensure consistency in abbreviation formatting.

Verify consistency between text and supplementary figures.

4. Minor Comments

Check consistency of abbreviations (LPOL vs LPOL/LPOR formatting).

Correct typographical errors.

Ensure all supplementary figures are referenced properly.

Standardize units (e.g., µmol CO₂ m⁻² s⁻¹ formatting).

Correct repetition in correlation description (e.g., duplicated trait names).

5. Final Recommendation

The study is scientifically relevant and potentially valuable for tea breeding programs. However, major revisions are required to improve statistical rigor, interpretation depth, and clarity of presentation.

Reviewer #2: 1. Abstract was long, delete or change the parts as I suggest in the reviewed manuscript (lines 25-27, 31-32).

2. Keywords were absent, add them.

3. Move lines 74-83 as I suggest in the reviewed manuscript.

4. Delete lines 83-91 as I suggest in the reviewed manuscript.

5. Line 126: “You must design and analyze the data using a randomized complete block design (RCBD) rather than CRD if the experiment conducted in outdoor. CRD is usually used to design the experiments that the condition of the experiment totally controlled, such as in laboratory or protected agriculture in controlled greenhouses. Therefore, write about the condition of the experiment site.”

6. Lines 128-134: “Write the temperature and humidity of the experiment site because the intensity of waterlogging is directly related to temperature.”

7. Line 156: Change to “number of leaves (NL)”

8. Lines 170-171: “What are FW, DW, TW, W1, and W2? They must be explained.”

9. Line 174: “Three readings per leaf are few for SPAD. It was better to have five or more.”

10. It is better to italicize the symbol letters of chlorophylls “a” and “b” throughout the manuscript, text, tables, and figures to distinguish them from ordinary letters.

11. Line 178: “Were the samples dry or fresh? How were they ground”?

12. Line 179: “According to which reference? To extract chlorophyll, usually, the ground leaf samples are left overnight.”

13. Line 202: “Were you conducting a comparison among means? If you, write the post hoc test. I strongly recommend displaying the results analytically rather than descriptively.”

14. Lines 223-231: “What is the aim of this description and its Figure? It looks like a summary of the results, so this must be the end of the Result section rather than at the beginning.”

15. Lines 248-251: “This introduction is not needed (delete). This makes your results long and cumbersome for the readers.”

16. Delete lines 352-355.

17. Lines 355-356: “Refer to this part of the PCA that was under waterlogging.”

18. Delete Table 1 (lines 369-372).

19. Delete lines 388-391.

20. Lines 391-392: “Refer to this part of the PCA under the recovery phase.”

21. Delete Table 2 (lines 405-408).

22. Lines 488-490: “You should start with description of G-1, G-2, and G-3 rather than start with G-3.”

23. Delete lines 542-573, “his part was not needed. This subsection is a literature review mixed with materials and methods rather than a discussion of the achieved results in this study. In the Discussion section, the authors must answer the "how" and "why" of their obtained results.”

24. Find the comments one by one in the reviewed manuscript.

Reviewer #3: A core part of the work is the analysis of variance. I find a strong misconception that experimental design also uses the method of projecting the results of the analysis of variance, which does not allow for a general understanding of statistical trends.

Reviewer #4: The manuscript is interesting, and the results could help in obtaining waterlogged genotypes for genetic improvement programs. However, it is very long (over 60 pages) and contains an excessive number of figures. I never found Table 1, which deals with the analysis of variance.

In the materials and methods section, the origin of the plants is not described, either from seed production or vegetatively. How and under what conditions were the plants produced (substrate used, greenhouse conditions, plant management, etc.) is not explained.

It is also unclear how the plants were established (bare-rooted, in some type of container, or in bags) inside the plastic boxes. It is important to monitor the oxygen level of the waterlogged water during the experiment, as this is a significant factor (hypoxia or anoxia).

The number of plants used to evaluate the waterlogged treatment, which was destructive, is not mentioned, nor is the number of plants that remained to evaluate recovery. The experimental design could have been better as a completely randomized factorial design, since only two factors (genotype and waterlogging treatment) were analyzed.

There were experimental units of 5 plants (one replicate) and three replicates were used, 15 plants per 10 genotypes = 150, in two treatments, for a total of 300 plants. Why is 500 mentioned?

It is worthwhile to include the statistical model used for data analysis. Mention whether a normality test was performed on the data.

The results are well described, but further discussion of the findings is needed. It is not necessary to discuss the method used to establish the waterlogging treatments; focus on the discussion of the results.

Finally, I believe that 14 days of treatment and recovery is insufficient, and the plants were not stressed enough to generate better results.

Reviewer #5: Abstract

• The abstract presents the general aim and main findings clearly; however, it is overly descriptive and contains several vague expressions such as “remarkably”, “substantially”, and “better responses”. These statements should be supported with key numerical values.

• The authors state that 11 traits were the most influential, but the statistical basis for this selection is not clearly explained in the abstract. The criterion should be briefly stated, for example PCA contribution, correlation strength, or consistency across stress and recovery phases.

• There is an inconsistency in abbreviations: lipid peroxidation is written as both LOPL and LPOL. This should be standardized throughout the manuscript.

Introduction

• The introduction provides sufficient background on waterlogging stress and its relevance to tea production. However, the novelty of the study is not sharply defined. The authors should explain more clearly how this work differs from previous tea waterlogging screening studies and why these specific eight genotypes were selected.

• The Bangladesh-specific problem is mentioned, but the production-level impact, affected regions, and practical breeding relevance are not sufficiently developed. A stronger justification is needed for why identifying tolerant genotypes at the sapling stage is agronomically meaningful.

• The objectives are clear, but they are broad. The authors should refine them into testable objectives linked directly to the measured traits and statistical analyses.

• I recommend supporting the first sentence (“Plants face various types of biotic and abiotic stresses throughout their life cycles”) with more recent and relevant literature (e.g., https://doi.org/10.1186/s44399-025-00024-8; https://doi.org/10.1186/s12870-025-07964-y). Similarly, the second sentence regarding waterlogging as a major abiotic stress should also be supported with an appropriate and up-to-date reference (e.g., Toprak, S., & Coşkun, Ö. F. (2024). Effect of gibberellic acid on waterlogging stress in melon. Proceedings of the VI International Agricultural, Biological, Life Science Conference (AGBIOL 2024), 358.).

Materials and methods

• The experimental design requires clarification. The authors state that the study followed a CRD with three replications and that five plants were considered one replicate, but it is not fully clear whether the experimental unit was the box or the individual plant. This is important because five plants in the same box may not represent independent biological replicates.

• The waterlogging treatment is described as maintaining water 5 cm above the collar region for 14 days, followed by 14 days of recovery. However, the authors do not report whether dissolved oxygen, soil/redox status, temperature, light conditions, or water renewal were monitored. These details are necessary to confirm that the imposed stress was consistent among genotypes.

• The control treatment is described only as “good cultural practices and ideal irrigation”. This is too vague. The authors should define irrigation frequency, substrate moisture status, pot/polybag size, growth medium, drainage condition, and nutrient management.

• The biochemical methods are insufficiently detailed. The phrase “with some slight change/minor changes” is not acceptable unless the exact modifications are described. The proline, antioxidant capacity, and TBARS protocols should include extraction volume, reagent composition, incubation conditions, calibration/standard curve details, and calculation formulas.

• The statistical analysis is incomplete. The model structure, factors, interaction terms, mean separation test, significance thresholds, data transformation, and assumptions of ANOVA should be reported. Since repeated measurements were taken at 7-day intervals, the authors should justify why a repeated-measures or mixed model was not used.

Results

• The results contain many numerical comparisons, but the presentation is often repetitive. The authors should focus on statistically supported genotype × treatment differences rather than listing the “best” genotype for each trait.

• The statement that V7 was the most tolerant genotype needs stronger quantitative support. Some traits identify other genotypes as superior, for example V5 for root fresh weight, V4 for several recovery traits, V3 for lipid peroxidation, and V6 for transpiration. Therefore, a clear tolerance index or ranking system should be provided.

• The PCA interpretation is not fully convincing. With only ten genotypes and 23 traits, PCA may be unstable and potentially over-interpreted. The authors should justify the use of PCA under this sample size and avoid making strong biological conclusions only from biplot positions.

• The correlation analysis is descriptive and does not sufficiently distinguish biologically meaningful correlations from statistical artifacts caused by many variables and small genotype number. Multiple-testing correction or at least a cautious interpretation is needed.

• Figures are informative but crowded. Several figures contain too many abbreviations and small labels, reducing readability. The authors should improve figure resolution, simplify captions, and move excessive abbreviation lists to a separate note or supplementary table.

Discussion

• The discussion should be more analytical. At present, it appears to restate the results rather than explaining the physiological basis of tolerance. The authors should better connect V7 tolerance with maintenance of photosynthesis, stomatal conductance, RWC, pigment stability, antioxidant capacity, and lower oxidative damage.

• The discussion should address contradictory findings. For example, if V7 is the most tolerant genotype, the authors should explain why other genotypes performed better for some root, dry matter, transpiration, or lipid peroxidation traits.

• The role of recovery phase is not sufficiently discussed. Recovery performance is highly important in waterlogging tolerance, and the authors should distinguish between genotypes that tolerate stress during flooding and those that recover better after drainage.

• Important mechanistic traits are missing. Since waterlogging tolerance in tea is often associated with root adaptation and aerenchyma formation, the lack of anatomical/root structural observations should be acknowledged as a limitation.

• The authors should avoid overclaiming commercial applicability. Since the experiment was conducted under controlled sapling conditions, field validation under naturally waterlogged tea estate conditions is necessary before recommending P/AFN/13/90 as a commercial tolerant genotype.

**********

6. PLOS authors have the option to publish the peer review history of their article (what does this mean?). If published, this will include your full peer review and any attached files.

If you choose “no”, your identity will remain anonymous but your review may still be made public.

**Do you want your identity to be public for this peer review?**  For information about this choice, including consent withdrawal, please see our Privacy Policy.

Reviewer #1: No

Reviewer #2: No

Reviewer #3: No

Reviewer #4: No

Reviewer #5: No

**********

[NOTE: If reviewer comments were submitted as an attachment file, they will be attached to this email and accessible via the submission site. Please log into your account, locate the manuscript record, and check for the action link "View Attachments". If this link does not appear, there are no attachment files.]

To ensure your figures meet our technical requirements, please review our figure guidelines: https://journals.plos.org/plosone/s/figures

You may also use PLOS’s free figure tool, NAAS, to help you prepare publication quality figures: https://journals.plos.org/plosone/s/figures#loc-tools-for-figure-preparation.

NAAS will assess whether your figures meet our technical requirements by comparing each figure against our figure specifications.

Attachment Submitted filename: v1 .pdf

Attachment Submitted filename: PONE-D-26-00282.pdf

Attachment Submitted filename: Riyadh et al., 2026.doc

# Author comment: Author response to Decision Letter 1

**DOI:** 10.1371/journal.pone.0354144.r002

10 Jun 2026

Response to comments of academic editor

Comments: (i) clarification and improvement of the experimental design and statistical analysis, including justification of the analytical approach and definition of experimental units,

Authors’ response: Thank you for this valuable comment. We have revised the manuscript to clarify the experimental design and statistical analysis. The analytical approach is now better justified, and the experimental units are explicitly defined in the Materials and Methods section.

Comments: (ii) more detailed and transparent methodological descriptions to ensure reproducibility,

Authors’ response: Thank you for this helpful comment. We have revised the Materials and Methods section to provide more detailed and transparent descriptions of the experimental procedures and statistical analyses. Specifically, we clarified the experimental design, and provided a stronger justification for the selected analytical approach. Additional information on data collection, treatment structure, replication, and statistical procedures has also been included to ensure that the study can be accurately interpreted.

Comments: (iii) improvement in the presentation and interpretation of results, avoiding redundancy and ensuring consistency across figures and tables,

Authors’ response: Thank you for this valuable comment. We have revised the Results section to improve the presentation. Redundant descriptions have been removed. We also carefully reviewed all figures and tables to ensure consistency in data presentation.

Comments: (iv) a more critical and mechanistic discussion supported by relevant and up-to-date literature.

Authors’ response: Thank you for this constructive comment. We have substantially revised the Discussion section to provide a more critical and mechanistic interpretation of the findings. We have also incorporated relevant and recent literature to support the interpretations, and to compare our findings with previous studies.

Response to comments of Reviewer #1:

Abstract

Comments: Clarify how tolerance was quantitatively determined.

Authors’ response: We would like to express our sincere thankfulness for your appreciation and efforts spent reviewing this paper, which will undoubtedly improve the manuscript's exposure and quality. We have carefully considered your suggestions and corrected them accordingly.

The tolerance was quantitatively determined by stress tolerance indices which has been included in abstract (L- 26, 27 of MS without track changes).

Comments: Avoid listing excessive abbreviations.

Authors’ response: We have carefully revised the abstract and reduced the number of abbreviations to only those that are essential.

Comments: State key numerical findings more concisely.

Authors’ response: We have revised the abstract to present the main numerical results in a more concise manner, emphasizing only the most important values.

Introduction

Comments: Improve flow and reduce repetition.

Authors’ response: Thank you for this constructive comment. Repetitive statements and redundant descriptions have been removed, and several sentences have been reorganized to ensure more logical presentation.

Comments: Strengthen gap identification.

Authors’ response: We have strengthened the identification of the research gap and included briefly (L: 107 - 120 of MS without track changes).

Comments: Clearly state novelty compared to previous tea studies.

Authors’ response: This study integrates both stress tolerance and post-waterlogging recovery responses by systematically evaluating the relative contributions of key adaptive traits under Bangladesh conditions, thereby providing a comprehensive framework for identifying waterlogging-tolerant tea genotypes. We have clarified the novelty of the study by distinguishing it from previous tea research and clearly emphasizing its unique objectives, and contributions (L: 107 - 120 of MS without track changes).

Materials and Methods

Comments: Clarify experimental unit and replication.

Authors’ response: We have revised the Materials and Methods section to clearly define the experimental unit and provide detailed information on the replication scheme used in the study (L: 130 - 134 of MS without track changes).

Comments: Provide more detail on environmental conditions (temperature, humidity).

Authors’ response: We have included the detailed environmental conditions, including temperature and relative humidity, which are now provided in Supplementary Figure S1 (L: 189 - 195 of MS without track changes)..

Comments: Specify light intensity during measurements.

Authors’ response: Thank you for the comment. We have revised the manuscript to specify the light intensity used during gas-exchange measurements, and this information has been included in the Materials and Methods section (L: 120 - 123 of MS without track changes).

Comments: Indicate calibration details of instruments.

Authors’ response: We have included instrument calibration details, and the corresponding calibration curves have been incorporated into the revised version (L: 249- 282 of MS without track changes).

Comments: Clarify whether measurements were taken at the same time of day.

Authors’ response: We have clarified in the revised manuscript that measurements of each interval were conducted at the same of day (L: 181 - 182 of MS without track changes).

Results

Comments: Reduce repetition between text and figures.

Authors’ response: We have corrected the manuscript to minimize repetition between the text and figures

Comments: Present key numerical values in tables rather than descriptive narration.

Authors’ response: We have revised the manuscript by presenting key numerical values in tables/ figures and reducing extensive descriptive narration to improve clarity and facilitate data interpretation.

Comments: Ensure figure numbering consistency.

Authors’ response: We have carefully checked and revised the manuscript to ensure consistent and correct figure numbering throughout the text and figure captions.

Comments: Improve clarity of clustering interpretation.

Authors’ response: We have improved the clarity of the clustering interpretation by providing a more detailed explanation of cluster formation, relationships among groups, and their mechanisms under different conditions (L: 767 - 809 of MS without track changes).

Discussion

Comments: Avoid restating results.

Authors’ response: We have avoided unnecessary restatement of results and focused the discussion on the interpretation, significance, and implications of the findings.

Comments: Strengthen mechanistic explanations.

Authors’ response: We have strengthened the mechanistic explanations by incorporating relevant morpho-physiological and biochemical processes and supporting them with appropriate literature references.

Comments: Compare findings more deeply with previous tea studies.

Authors’ response: Thank you for the suggestion. Since studies on waterlogging effects in tea are very limited, we have compared our findings with the few available tea-related reports and supplemented the discussion with relevant evidence from other abiotic stress studies in tea to provide broader physiological context.

Comments: Distinguish clearly between stress response and adaptive tolerance.

Authors’ response: The response and tolerance were discussed together from an integrated morpho-physiological and biochemical point of view during waterlogging and the recovery phase.

Figures and Tables

Comments: Improve resolution and readability.

Authors’ response: We have revised all figures according to the journal's guidelines, improving their resolution, readability, and overall presentation quality.

Comments: Shorten overly long figure captions.

Authors’ response: We have shortened some figure captions by retaining only essential information while ensuring that the figures remain clear, informative, and self-explanatory.

Comments: Ensure consistency in abbreviation formatting.

Authors’ response: We have carefully reviewed the manuscript and standardized the formatting of all abbreviations to ensure consistency throughout the text, tables, and figures.

Comments: Verify consistency between text and supplementary figures.

Authors’ response: We have thoroughly cross-checked the manuscript and supplementary figures to ensure consistency in the presentation, labeling, and interpretation of the data throughout the revised version.

4. Minor Comments

Comments: Check consistency of abbreviations (LPOL vs LPOL/LPOR formatting).

Authors’ response: We have carefully reviewed and corrected the abbreviation formatting throughout the manuscript and related terms in the text, tables, figures, and supplementary materials.

Comments: Correct typographical errors.

Authors’ response: Thank you for the comment. We have carefully proofread the entire manuscript and corrected typographical, grammatical, and formatting errors throughout the revised version.

Comments: Ensure all supplementary figures are referenced properly.

Authors’ response: We have carefully cited supplementary figures and referenced in the text where necessary.

Comments: Standardize units (e.g., µmol CO₂ m⁻² s⁻¹ formatting).

Authors’ response: We have revised the manuscript to standardize the formatting of all units,

Comments: Correct repetition in correlation description (e.g., duplicated trait names).

Authors’ response: We have carefully reviewed the correlation analysis section and corrected the repeated trait names and other redundant descriptions to improve clarity and accuracy.

5. Final Recommendation

Comments: The study is scientifically relevant and potentially valuable for tea breeding programs. However, major revisions are required to improve statistical rigor, interpretation depth, and clarity of presentation.

Authors’ response: Thank you for your valuable suggestions. We have incorporated the queries in the revised manuscripts.

Thanks again for your valuable time and please check the revised manuscript and we made the corrections/improvements in track changed mode.

Response to comments of Reviewer #2:

Comments: 1. Abstract was long, delete or change the parts as I suggest in the reviewed manuscript (lines 25-27, 31-32).

Authors’ response: We wish to express our sincere gratitude for taking the time to review this manuscript. We appreciate your valuable feedback which will certainly improve the quality of the manuscript. We have respectfully considered your valuable comments and revised the manuscript accordingly.

We have revised the Abstract by deleting and modifying the indicated lines.

Comments: 2. Keywords were absent, add them.

Authors’ response: We have added appropriate keywords to the revised manuscript to improve its indexing, discoverability, and alignment with the journal’s requirements.

Comments: 3. Move lines 74-83 as I suggest in the reviewed manuscript.

Authors’ response: We have revised the manuscript accordingly and moved the content from Lines 74–83 to the suggested location to improve the logical flow.

Comments: 4. Delete lines 83-91 as I suggest in the reviewed manuscript.

Authors’ response: We have revised the manuscript accordingly and deleted the text in Lines 83–91 as recommended.

Comments: 5. Line 126: “You must design and analyze the data using a randomized complete block design (RCBD) rather than CRD if the experiment conducted in outdoor. CRD is usually used to design the experiments that the condition of the experiment totally controlled, such as in laboratory or protected agriculture in controlled greenhouses. Therefore, write about the condition of the experiment site.”

Authors’ response: Thank you for your valuable comments. The justification for the CRD experiment has provided with the condition of the experimental site (L: 189 - 198 of MS without track changes).

Comments: 6. Lines 128-134: “Write the temperature and humidity of the experiment site because the intensity of waterlogging is directly related to temperature.”

Authors’ response: The mean temperature and humidity of the experimental site are provided in supplementary figure (S1 figure) (L: 193 - 195 of MS without track changes).

Comments: 7. Line 156: Change to “number of leaves (NL)”

Authors’ response: The correction has been done.

Comments: 8. Lines 170-171: “What are FW, DW, TW, W1, and W2? They must be explained.”

Authors’ response: The abbreviations with explanation are provided in L: 222 - 231 of MS without track changes.

Comments: 9. Line 174: “Three readings per leaf are few for SPAD. It was better to have five or more.”

Authors’ response: Thank you for your valuable suggestion. This will be included in our future research as a valuable direction.

Comments: 10. It is better to italicize the symbol letters of chlorophylls “a” and “b” throughout the manuscript, text, tables, and figures to distinguish them from ordinary letters.

Authors’ response: Thank you for the valuable suggestion. We have revised the manuscript to italicize the symbols of chlorophyll ‘a’ and chlorophyll ‘b’ consistently throughout the manuscript.

Comments: 11. Line 178: “Were the samples dry or fresh? How were they ground”? - Authors’ response: The samples used for analysis were fresh, and the extraction procedures have been clearly described in the Materials and Methods section of the revised manuscript (L: 237 - 245 of MS without track changes).

Comments: 12. Line 179: “According to which reference? To extract chlorophyll, usually, the ground leaf samples are left overnight.”

Authors’ response: Thank you for the comment. We have clarified the chlorophyll extraction procedure in the revised manuscript and included the appropriate reference supporting the method used. The extraction protocol, including sample preparation and extraction duration, is now described in greater detail in the Materials and Methods section (L: 237 - 245 of MS without track changes).

Comments: 13. Line 202: “Were you conducting a comparison among means? If you, write the post hoc test. I strongly recommend displaying the results analytically rather than descriptively.”

Authors’ response: We have performed post hoc test. In the figures (S2-S24), lettering was not provided because of overcrowding of data, and it also reduced the readability of figures drastically. The LSD values are now incorporated in ANOVA (S1 table).

Comments: 14. Lines 223-231: “What is the aim of this description and its Figure? It looks like a summary of the results, so this must be the end of the Result section rather than at the beginning.”

Authors’ response: The figure mainly showed the difference between waterlogging stress and control conditions, using descriptive statistics.

Comments: 15. Lines 248-251: “This introduction is not needed (delete). This makes your results long and cumbersome for the readers.”

Authors’ response: Thank you for the comment. This introduction has been removed.

Comments: 16. Delete lines 352-355.

Authors’ response: Thank you for the comment. These lines have been removed.

Comments: 17. Lines 355-356: “Refer to this part of the PCA that was under waterlogging.”

Authors’ response: The correction has been done accordingly.

Comments: 18. Delete Table 1 (lines 369-372).

Authors’ response: Thank you for the comment. This table has been moved to supplementary.

Comments: 19. Delete lines 388-391.

Authors’ response: Thank you for the comment. These marked lines have been removed.

Comments: 20. Lines 391-392: “Refer to this part of the PCA under the recovery phase.”

Authors’ response: Thank you for the suggestion. The correction has been done accordingly.

Comments: 21. Delete Table 2 (lines 405-408).

Authors’ response: Thank you for the comment. This table has been moved to supplementary.

Comments: 22. Lines 488-490: “You should start with description of G-1, G-2, and G-3 rather than start with G-3.”

Authors’ response: Thank you for the suggestion. The corrections have made according to your comment.

Comments: 23. Delete lines 542-573, this part was not needed. This subsection is a literature review mixed with materials and methods

Attachment Submitted filename: Response to Reviewers.doc

# Peer review: Decision Letter 1

**DOI:** 10.1371/journal.pone.0354144.r003

**Authors:** Ömer Faruk Coşkun

5 Jul 2026

Assessment of waterlogging tolerance in tea genotypes through morpho-physiological and biochemical profiling

PONE-D-26-00282R1

Dear Dr. Arefin,

We’re pleased to inform you that your manuscript has been judged scientifically suitable for publication and will be formally accepted for publication once it meets all outstanding technical requirements.

Within one week, you’ll receive an e-mail detailing the required amendments. When these have been addressed, you’ll receive a formal acceptance letter and your manuscript will be scheduled for publication.

An invoice will be generated when your article is formally accepted. Please note, if your institution has a publishing partnership with PLOS and your article meets the relevant criteria, all or part of your publication costs will be covered. Please make sure your user information is up-to-date by logging into Editorial Manager at Editorial Manager® and clicking the ‘Update My Information' link at the top of the page. For questions related to billing, please contact billing support.

If your institution or institutions have a press office, please notify them about your upcoming paper to help maximize its impact. If they’ll be preparing press materials, please inform our press team as soon as possible -- no later than 48 hours after receiving the formal acceptance. Your manuscript will remain under strict press embargo until 2 pm Eastern Time on the date of publication. For more information, please contact onepress@plos.org.

Kind regards,

Ömer Faruk Coşkun, Ph.D

Academic Editor

PLOS One

Additional Editor Comments (optional):

Thank you for submitting the revised version of your manuscript. The authors have carefully addressed the reviewers' comments, and the manuscript has improved substantially. Based on the revised manuscript, the reviewers' recommendations, and my own evaluation, I believe that the manuscript is now suitable for publication in PLOS ONE. I therefore recommend acceptance.

Reviewers' comments:

Reviewer's Responses to Questions

**Comments to the Author**

1. If the authors have adequately addressed your comments raised in a previous round of review and you feel that this manuscript is now acceptable for publication, you may indicate that here to bypass the “Comments to the Author” section, enter your conflict of interest statement in the “Confidential to Editor” section, and submit your "Accept" recommendation.

Reviewer #2: (No Response)

Reviewer #5: All comments have been addressed

**********

2. Is the manuscript technically sound, and do the data support the conclusions?

The manuscript must describe a technically sound piece of scientific research with data that supports the conclusions. Experiments must have been conducted rigorously, with appropriate controls, replication, and sample sizes. The conclusions must be drawn appropriately based on the data presented.

Reviewer #2: (No Response)

Reviewer #5: Yes

**********

3. Has the statistical analysis been performed appropriately and rigorously?

Reviewer #2: (No Response)

Reviewer #5: Yes

**********

4. Have the authors made all data underlying the findings in their manuscript fully available?

The PLOS Data policy requires authors to make all data underlying the findings described in their manuscript fully available without restriction, with rare exception (please refer to the Data Availability Statement in the manuscript PDF file). The data should be provided as part of the manuscript or its supporting information, or deposited to a public repository. For example, in addition to summary statistics, the data points behind means, medians and variance measures should be available. If there are restrictions on publicly sharing data—e.g. participant privacy or use of data from a third party—those must be specified.

Reviewer #2: (No Response)

Reviewer #5: Yes

**********

5. Is the manuscript presented in an intelligible fashion and written in standard English?

PLOS ONE does not copyedit accepted manuscripts, so the language in submitted articles must be clear, correct, and unambiguous. Any typographical or grammatical errors should be corrected at revision, so please note any specific errors here.

Reviewer #2: (No Response)

Reviewer #5: Yes

**********

6. Review Comments to the Author

Please use the space provided to explain your answers to the questions above. You may also include additional comments for the author, including concerns about dual publication, research ethics, or publication ethics. (Please upload your review as an attachment if it exceeds 20,000 characters)

Reviewer #2: (No Response)

Reviewer #5: After carefully evaluating the revised manuscript and the authors’ responses to the reviewers’ comments, I find that the major concerns raised during the previous review round have been adequately addressed. The manuscript has been substantially improved in terms of methodological clarity, statistical analysis, presentation of results, and discussion of the findings.

The study provides useful information on waterlogging tolerance in tea genotypes and is of potential value for future breeding programs. I have no further major scientific concerns.

I therefore recommend acceptance of the manuscript for publication.

**********

7. PLOS authors have the option to publish the peer review history of their article (what does this mean?). If published, this will include your full peer review and any attached files.

If you choose “no”, your identity will remain anonymous but your review may still be made public.

**Do you want your identity to be public for this peer review?**  For information about this choice, including consent withdrawal, please see our Privacy Policy.

Reviewer #2: No

Reviewer #5: No

**********

# Editor report: Acceptance letter

**DOI:** 10.1371/journal.pone.0354144.r004

**Authors:** Ömer Faruk Coşkun

PONE-D-26-00282R1

PLOS One

Dear Dr. Arefin,

I'm pleased to inform you that your manuscript has been deemed suitable for publication in PLOS One. Congratulations! Your manuscript is now being handed over to our production team.

At this stage, our production department will prepare your paper for publication. This includes ensuring the following:

* All references, tables, and figures are properly cited

* All relevant supporting information is included in the manuscript submission,

* There are no issues that prevent the paper from being properly typeset

You will receive further instructions from the production team, including instructions on how to review your proof when it is ready. Please keep in mind that we are working through a large volume of accepted articles, so please give us a few days to review your paper and let you know the next and final steps.

Lastly, if your institution or institutions have a press office, please let them know about your upcoming paper now to help maximize its impact. If they'll be preparing press materials, please inform our press team within the next 48 hours. Your manuscript will remain under strict press embargo until 2 pm Eastern Time on the date of publication. For more information, please contact onepress@plos.org.

You will receive an invoice from PLOS for your publication fee after your manuscript has reached the completed accept phase. If you receive an email requesting payment before acceptance or for any other service, this may be a phishing scheme. Learn how to identify phishing emails and protect your accounts at https://explore.plos.org/phishing.

If we can help with anything else, please email us at customercare@plos.org.

Thank you for submitting your work to PLOS ONE and supporting open access.

Kind regards,

PLOS ONE Editorial Office Staff

on behalf of

Professor Ömer Faruk Coşkun

Academic Editor

PLOS One
